# Hybrid exosomal nanomotors convert pathological ROS into transport-driving cues to enhance cartilage accumulation in osteoarthritis

**DOI:** 10.1038/s41467-026-76949-8

**Published:** 2026-08-29

**Authors:** Kai Huang, Hui-Zhi Liu, Yuan Liu, Jia-Yan Lyu, Qing-Yi Zhang, Zi-Yuan Feng, Zi-Lin Wang, Hui Dong, Rong Nie, Yi-Wen Zhang, Yue-Qi Zhang, Yi Zeng, Hui-Qi Xie, Zhou Zhou, Bin Shen

**Affiliations:** 1 Department of Orthopedic Surgery and Orthopedic Research Institute, West China Hospital, Sichuan University, Chengdu, China; 2 Stem Cell and Tissue Engineering Research Center, State Key Laboratory of Biotherapy, West China Hospital, Sichuan University, Chengdu, China; 3 Key Laboratory of Drug-Targeting and Drug Delivery System of the Education Ministry and Sichuan Province, Sichuan Engineering Laboratory for Plant-Sourced Drug and Sichuan Research Center for Drug Precision Industrial Technology, West China School of Pharmacy, Sichuan University, Chengdu, China; 4 Hongqiao International Institute of Medicine, Tongren Hospital and State Key Laboratory of Systems Medicine for Cancer, Department of Pharmacology and Chemical Biology, Shanghai Jiao Tong University School of Medicine, Shanghai, China; 5 Department of Urology, West China Hospital, Sichuan University, Chengdu, China

**Keywords:** Trauma, Drug delivery, Mesenchymal stem cells

## Abstract

Osteoarthritis (OA) remains difficult to treat because drug transport within cartilage is restricted by its dense extracellular matrix and rapid joint clearance. Here we show that hybrid exosomal nanomotors (CAP-Mn/140@hyExos) convert pathological reactive oxygen species (ROS) into transport-driving cues to overcome diffusion-limited transport within cartilage. In the oxidative OA microenvironment, manganese dioxide (MnO₂) catalyzes H₂O₂ decomposition, driving ROS-responsive transport, while a cartilage-targeting peptide enhances matrix interaction, together promoting cartilage accumulation. This strategy achieves deeper penetration (~140 μm), greater cellular uptake (~2.5-fold), and prolonged intra-articular retention (~30% remaining at two weeks). Enhanced transport enables efficient intracellular delivery of miR-140 and MnO₂ nanoparticles, attenuating oxidative stress and promoting cartilage regeneration. In a rat OA model, this therapeutic strategy improves gait performance, restores subchondral bone microarchitecture, and suppresses OA progression. These findings establish pathological microenvironments as endogenous drivers of intratissue drug transport, providing a transport-regulated strategy for drug delivery in transport-restricted tissues.

## Abstract (web summary)

Osteoarthritis therapies are limited by poor drug transport within dense cartilage. Here, authors develop hybrid exosomal nanomotors that convert pathological ROS into transport-driving cues, enhancing transport within cartilage and slowing osteoarthritis progression.

## Introduction

Osteoarthritis (OA) is a highly prevalent degenerative joint disease among middle-aged and elderly individuals, characterized by progressive pain, structural deformity, and loss of joint function^1^. Globally, it is estimated that knee OA affects ~15.6% of men and 30.5% of women over the age of 55, imposing a substantial socioeconomic burden^2^. At the pathological level, disruption of cartilage homeostasis drives disease progression, involving excessive matrix degradation and chondrocyte apoptosis, ultimately leading to irreversible structural damage^3,4^. Although joint replacement surgery can restore function in end-stage disease, it remains invasive and associated with potential complications^5^. Pharmacological interventions, by contrast, are largely palliative and fail to halt or reverse disease progression^6,7^.

A major limitation of OA pharmacotherapy is that drug transport within cartilage remains fundamentally inefficient, resulting in inadequate local concentrations even following intra-articular administration^8^. Owing to the avascular nature of cartilage, systemically delivered agents exhibit poor tissue accumulation, while intra-articular injection is constrained by rapid clearance from the joint cavity and restricted movement within the dense extracellular matrix^9–11^. These limitations are further compounded by the fact that chondrocytes occupy only a small fraction of cartilage volume yet are essential for maintaining matrix homeostasis, thereby requiring efficient delivery following tissue penetration^12^. Importantly, these barriers do not act independently but form a hierarchically coupled system in which transport inefficiency at the tissue level directly governs cellular accessibility and therapeutic efficacy^13^. Meanwhile, OA cartilage is characterized by persistent oxidative stress, where excessive accumulation of reactive oxygen species (ROS) contributes to cellular dysfunction and amplifies inflammatory cascades^14,15^. Existing therapeutic strategies primarily focus on scavenging ROS to mitigate damage, yet fail to exploit ROS as a functional regulator of transport behavior within the tissue microenvironment^16,17^. Thus, converting ROS from a pathological mediator into a transport-driving cue offers an approach for actively regulating intratissue transport rather than passive diffusion.

Exosomes, nanoscale extracellular vesicles (30–150 nm), have emerged as promising delivery vehicles due to their intrinsic biocompatibility, low immunogenicity, and capacity to transport bioactive molecules such as proteins, lipids, and nucleic acids^18,19^. Engineering approaches, particularly those enhancing miRNA loading, have significantly improved their therapeutic potential^20,21^. Among these, miR-140 plays a central role in cartilage homeostasis by regulating inflammation, matrix metabolism, and chondrogenic differentiation^22–24^. Our previous work demonstrated that miR-140-enriched exosomes can attenuate OA progression by promoting mitophagy through suppression of CAPN1^25^. However, both native and engineered exosomes remain limited by insufficient movement within the dense cartilage matrix, reflecting the broader constraint of diffusion-dominated transport.

Recent advances in nanomotor technology provide a potential strategy to overcome this limitation by enabling the conversion of energy into autonomous motion^26,27^. In contrast to systems driven by external physical fields or biological carriers^28,29^, chemically powered nanomotors exploit chemical energy transduction to achieve self-propelled motion within complex biological environments, thereby offering improved compatibility with physiological conditions^30^. However, their transport behavior remains largely decoupled from disease-specific microenvironmental cues within pathological tissues. In this context, manganese dioxide nanoparticles (MnO₂ NPs) offer a chemically driven motility module that is inherently responsive to disease-associated biochemical conditions. In ROS-enriched environments, MnO₂ NPs catalyzes the decomposition of H₂O₂, thereby coupling redox reactions with modulation of nanoscale motion^31,32^. This enables pathological ROS to be repurposed as endogenous motility-regulating signals, introducing spatial redistribution toward regions of elevated oxidative stress and enabling environment-adaptive intratissue transport behavior. In addition, incorporation of tissue-specific targeting further refines transport behavior^33,34^. Cartilage-targeting peptides (CAP) bind extracellular matrix components with high affinity, promoting intratissue retention and increasing interactions with chondrocytes^35,36^. Notably, such matrix interactions impose spatial confinement, which couples with active motion to generate biased transport rather than passive diffusion, thereby enhancing both cartilage accumulation and cellular uptake.

**Fig. 1:**
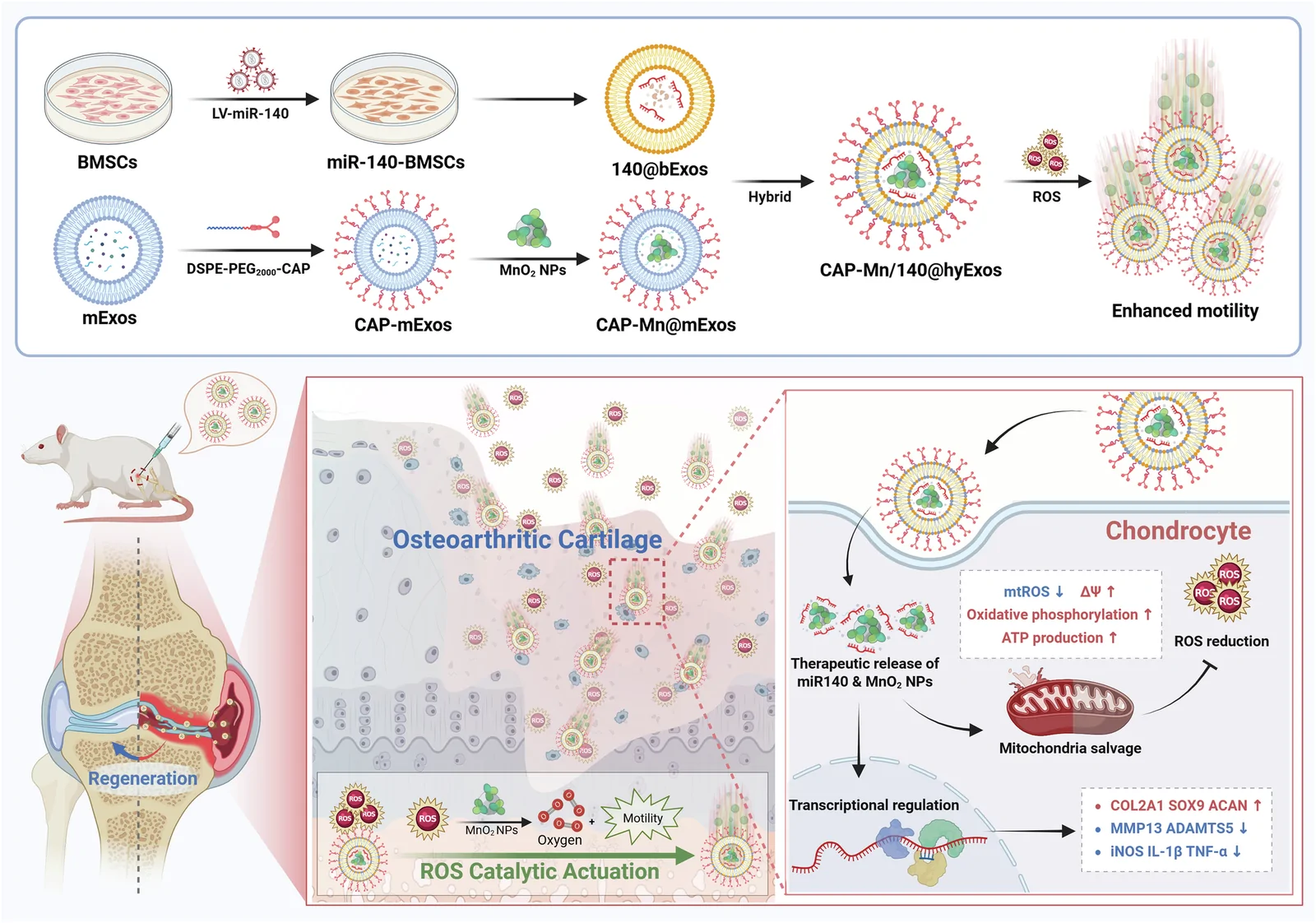
Graphical abstract of hybrid exosomal nanomotors for OA therapy. Hybrid exosomal nanomotors (CAP-Mn/140@hyExos) utilize MnO₂ NPs to catalyze H₂O₂ decomposition within the oxidative osteoarthritic microenvironment, thereby converting pathological ROS into a driving cue that enables ROS-responsive enhancement of stochastic motility and biased intratissue transport through dense cartilage. Coupled with CAP-mediated matrix interactions and confinement, this transport behavior significantly enhances cartilage penetration and cellular uptake, enabling efficient delivery of miR-140 and MnO₂ NPs. The resulting therapeutic effects enhance oxidative detoxification, restore mitochondrial energy metabolism, and suppress inflammatory signaling, collectively promoting cartilage regeneration and functional recovery. Created in BioRender. Huang, K. (https://BioRender.com/nqgl3ax).

Here, we develop a ROS-responsive hybrid exosomal nanomotor system (CAP-Mn/140@hyExos) that integrates reaction-coupled motion, matrix-targeted interactions, and intracellular delivery within a unified platform (Fig. 1). Constructed via membrane fusion, the system incorporates MnO₂ NPs, CAP modification, and therapeutic miR-140 loading. By converting pathological ROS into a motility-regulating cue, this system enables microenvironment-dependent enhancement of intratissue transport, thereby overcoming diffusion-limited delivery and improving both tissue penetration and cellular uptake. This work, therefore, establishes a microenvironment-guided transport strategy in which pathological signals are not merely therapeutic targets but functional regulators of transport behavior in transport-restricted biological tissues.

## Results

### Isolation and Characterization of BMSCs and Chondrocytes

Bone marrow mesenchymal stem cells (BMSCs) were successfully isolated from the bone marrow of Sprague Dawley (SD) rats and expanded under in vitro culture conditions. As shown in Supplementary Fig. 1, the cells exhibited characteristic spindle or polygonal morphology, and no significant alterations in cellular morphology or proliferation capacity were observed during passages. Flow cytometric analysis revealed high expression of stem cell surface markers CD90 and CD73, along with negligible expression of hematopoietic markers CD34 and CD45 (Supplementary Fig. 2), indicating a highly pure BMSC population without detectable contamination. After induction along osteogenic, adipogenic, and chondrogenic lineages, specific staining revealed the formation of mineralized deposits (osteogenesis), intracellular lipid accumulations (adipogenesis), and distinct chondrocytic lacunae (chondrogenesis) (Supplementary Fig. 3), confirming the trilineage differentiation capacity of the isolated BMSCs.

Similarly, chondrocytes were successfully extracted from the articular cartilage of SD rats. The cells displayed a relatively regular polygonal morphology and could be stably expanded and passaged in vitro (Supplementary Fig. 4). Immunofluorescence and Western blot analyses indicated high expression of the anabolic marker COL2A1 and low expression of the catabolic marker MMP13 and inflammatory factor iNOS (Supplementary Fig. 5-6). Additionally, positive staining with Alcian blue and Safranin O confirmed abundant glycosaminoglycan deposition, consistent with typical chondrocyte phenotype maintenance (Supplementary Fig. 7). In contrast, interleukin-1β (IL-1β) stimulation resulted in marked downregulation of COL2A1, accompanied by significant upregulation of MMP13 and iNOS, as evidenced by both immunofluorescence and Western blotting (Supplementary Fig. 5-6). Concomitant reduction in Alcian blue and Safranin O staining intensity further validated the successful establishment of an IL-1β-stimulated inflammatory chondrocyte model mimicking OA-like catabolic phenotype (Supplementary Fig. 7).

### Preparation and characterization of exosomal nanomotors

**Fig. 2:**
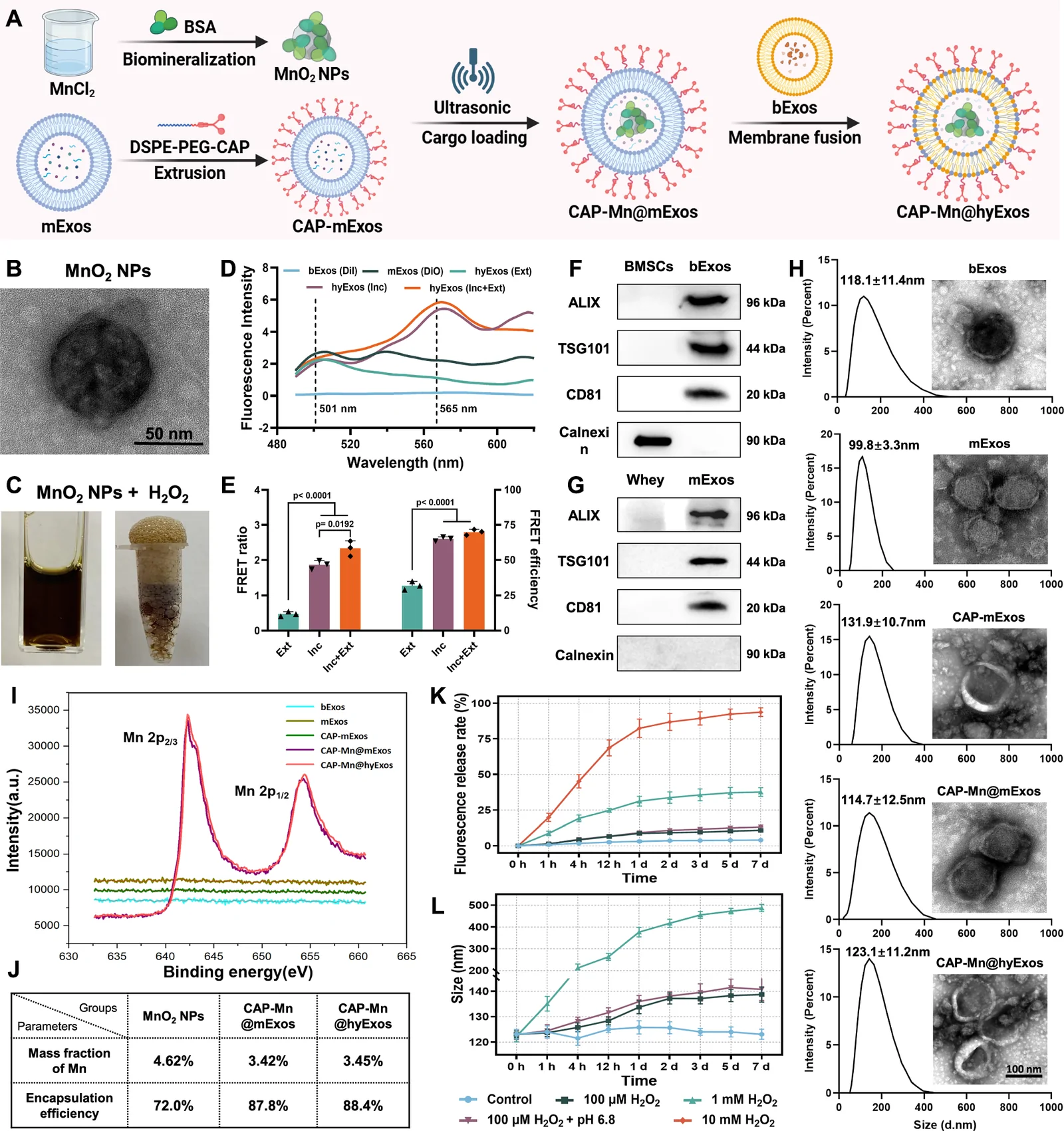
Preparation and characterization of exosomal nanomotors. **A** Schematic illustration of the fabrication procedure of exosomal nanomotors. Created in BioRender. Huang, K. (https://BioRender.com/nqgl3ax). **B** TEM image of MnO₂ NPs, scale bar = 50 nm. **C** Catalytic activity of MnO₂ NPs, showing bubble generation upon addition of H₂O₂. **D** FRET-based analysis of membrane fusion between exosomes using incubation, extrusion, and combined “incubation-extrusion” methods. **E** Quantitative evaluation of the FRET ratio and FRET efficiency (*n* = 3, biological replicates per group). **F**, **G** Western blot analysis of exosomal marker proteins ALIX, TSG101, CD81, and Calnexin. **H** Representative TEM micrographs showing morphology and NTA analysis showing size distribution, scale bar = 100 nm. **I** XPS analysis of manganese chemical states. **J** ICP-OES quantification of manganese content and encapsulation efficiency in different exosomal formulations (*n* = 3, biological replicates per group). **K** Fluorescence release of DiI-labeled CAP-Mn@hyExos over 7 days under OA-relevant microenvironmental conditions (*n* = 3, biological replicates per group). **L** DLS analysis of the hydrodynamic diameter of CAP-Mn@hyExos over 7 days under OA-relevant microenvironmental conditions (*n* = 3, biological replicates per group). Data are presented as the mean ± SD. *P* values are from one-way analysis of variance (ANOVA) with Tukey’s multiple comparisons test (**E**). Data in (**B–D**,** H**,** I**) are representative of three independent experiments with similar results. Source data are provided as a Source Data file.

The fabrication process of the exosomal nanomotors is schematically illustrated in Fig. 2A. (i) Initially, MnO₂ NPs were synthesized through a biomineralization method using bovine serum albumin (BSA)-modified manganese chloride. Transmission electron microscopy (TEM) revealed that the MnO₂ NPs possessed a uniform spherical morphology with an average diameter of ~70 nm (Fig. 2B). These nanoparticles demonstrated catalytic activity toward H₂O₂ decomposition, generating visible gas evolution in the presence of H₂O₂ (Fig. 2C). (ii) Subsequently, a cartilage targeting peptide CAP was covalently conjugated to DSPE-PEG₂₀₀₀-MAL via an maleimide–thiol coupling reaction, yielding DSPE-PEG₂₀₀₀-CAP (Supplementary Fig. 8). Successful conjugation was confirmed by ^1^H nuclear magnetic resonance (^1^H-NMR) and Fourier transform infrared (FT-IR) spectroscopy, which displayed characteristic absorption peaks of CAP alongside preserved signals from the PEG backbone, in contrast to the DSPE-PEG₂₀₀₀-MAL precursor (Supplementary Fig. 9–10). This modification facilitated the incorporation of CAP into the exosomal membrane, thereby conferring cartilage binding capability. Further extrusion of mExos with DSPE-PEG₂₀₀₀-CAP resulted in an ~2 mV increase in zeta potential, suggesting successful surface modification, attributable to the weakly positive charge of CAP (Supplementary Table 1). (iii) MnO₂ NPs were then encapsulated into CAP-mExos via ultrasonication, resulting in CAP-Mn@mExos. Optimization experiments revealed that a MnO₂ NPs-to-mExos number ratio of 1:1 provided optimal particle size distribution and polydispersity index (PDI) (Supplementary Table 2). (iv) Finally, CAP-Mn@mExos were fused with bExos to generate hybrid exosomal nanomotors (CAP-Mn@hyExos). Fusion efficiency was assessed by fluorescence resonance energy transfer (FRET) analysis, in which DiO-labeled mExos and DiI-labeled bExos were mixed at a 1:1 ratio and subjected to either incubation or extrusion, followed by measurement of FRET signals under excitation at 501 and 567 nm. The incubation group exhibited a pronounced increase in FRET signal, indicating effective membrane fusion, but it was accompanied by increased particle size and PDI. In contrast, the extrusion group displayed a more uniform size distribution with a lower PDI, albeit with relatively weaker FRET signals. Based on these observations, a combined “incubation-extrusion” strategy was adopted to balance fusion efficiency and structural uniformity. Quantitative analysis further revealed that this combined approach achieved a FRET efficiency of ~70%, demonstrating efficient and stable membrane fusion between the two exosome populations (Fig. 2D, E and Supplementary Fig. 11).

The obtained exosomal formulations underwent systematic physicochemical characterization. Initially, Western blot analysis confirmed the presence of characteristic exosomal surface markers. Both bExos and mExos exhibited high expression of ALIX, TSG101, and CD81, while showing no detectable expression of Calnexin (Fig. 2F, G), in accordance with the criteria of the International Society for Extracellular Vesicles for exosome identification. Sodium dodecyl sulfate-polyacrylamide gel electrophoresis (SDS-PAGE) analysis further revealed distinct protein banding patterns among the groups, indicating that CAP modification, MnO₂ NPs loading, and membrane fusion procedures preserved the intrinsic protein profile, thereby verifying the structural integrity of the hybrid exosome nanomotors (Supplementary Fig. 12). TEM imaging confirmed the spherical morphology of all formulations, while nanoparticle tracking analysis (NTA) showed a predominant size distribution of 90–150 nm (Fig. 2H). Although CAP modification and MnO₂ loading induced a slight increase in hydrodynamic diameter, colloidal stability was maintained. Cryo-electron microscopy (cryo-EM) further provided direct structural evidence of MnO₂ association, where CAP-Mn@mExos and CAP-Mn@hyExos exhibited distinct intravesicular high electron-density regions that were absent in all control groups (Supplementary Fig. 13). X-ray photoelectron spectroscopy (XPS) further confirmed MnO₂ incorporation, with characteristic *Mn* 2*p*₃/₂ and *Mn* 2*p*₁/₂ peaks at 640 eV and 655 eV observed exclusively in MnO₂-loaded groups (Fig. 2I). In addition, energy-dispersive X-ray spectroscopy (EDS) mapping revealed no resolvable Mn signal above background due to the low loading content and nanoscale constraints; however, an increased nitrogen signal in CAP-Mn@hyExos provided indirect compositional evidence consistent with BSA-mediated hybrid assembly (Supplementary Fig. 14). Quantitative inductively coupled plasma optical emission spectrometry (ICP-OES) analysis showed a Mn mass fraction of 4.62% in MnO₂ NPs, corresponding to an encapsulation efficiency of 72.0%. After exosomal loading, Mn content reached 3.42% and 3.45% in CAP-Mn@mExos and CAP-Mn@hyExos, respectively, with encapsulation efficiencies increased to 87.8% and 88.4% (Fig. 2J). This enhancement is attributed to the stabilizing effect of the exosomal lipid bilayer, which facilitates efficient MnO₂ retention during assembly.

Furthermore, dynamic light scattering (DLS) analysis after 7 days of incubation in simulated synovial fluid revealed negligible changes in particle size and PDI across all groups, indicating good physicochemical stability and structural integrity under physiological conditions (Supplementary Fig. 15). To further evaluate the structural stability of the exosomal nanomotor in OA-relevant microenvironments, its responsiveness under different stimuli was systematically assessed (Fig. 2K, L and Supplementary Fig. 16). In simulated synovial fluid, the system exhibited stability, with consistently low fluorescence leakage and no evident time-dependent variation, confirming favorable colloidal stability under physiological conditions. At low H₂O₂ concentration (100 μM), only mild perturbations were observed, reflected by slight increases in particle size and fluorescence leakage, without detectable structural damage, indicating limited impact of low-level ROS. When H₂O₂ was increased to 1 mM or higher, a pronounced reduction in stability occurred, accompanied by significant fluorescence leakage and marked particle enlargement, suggesting structural perturbation of MnO₂-containing components under strong oxidative conditions. Under combined 100 μM H₂O₂ and mildly acidic conditions (pH 6.8), the system showed moderate and well-tolerated structural changes, with only slight increases in particle size and leakage while maintaining overall dispersity and integrity. These findings indicate that the nanomotor remains structurally stable under OA-like acidic and oxidative microenvironments. Collectively, these comprehensive analyses demonstrate that the engineered exosomal nanomotors exhibit well-defined structure, high payload capacity, and stability, providing a solid foundation for their biomedical applications.

### ROS-responsive motility enables enhanced cartilage penetration

**Fig. 3:**
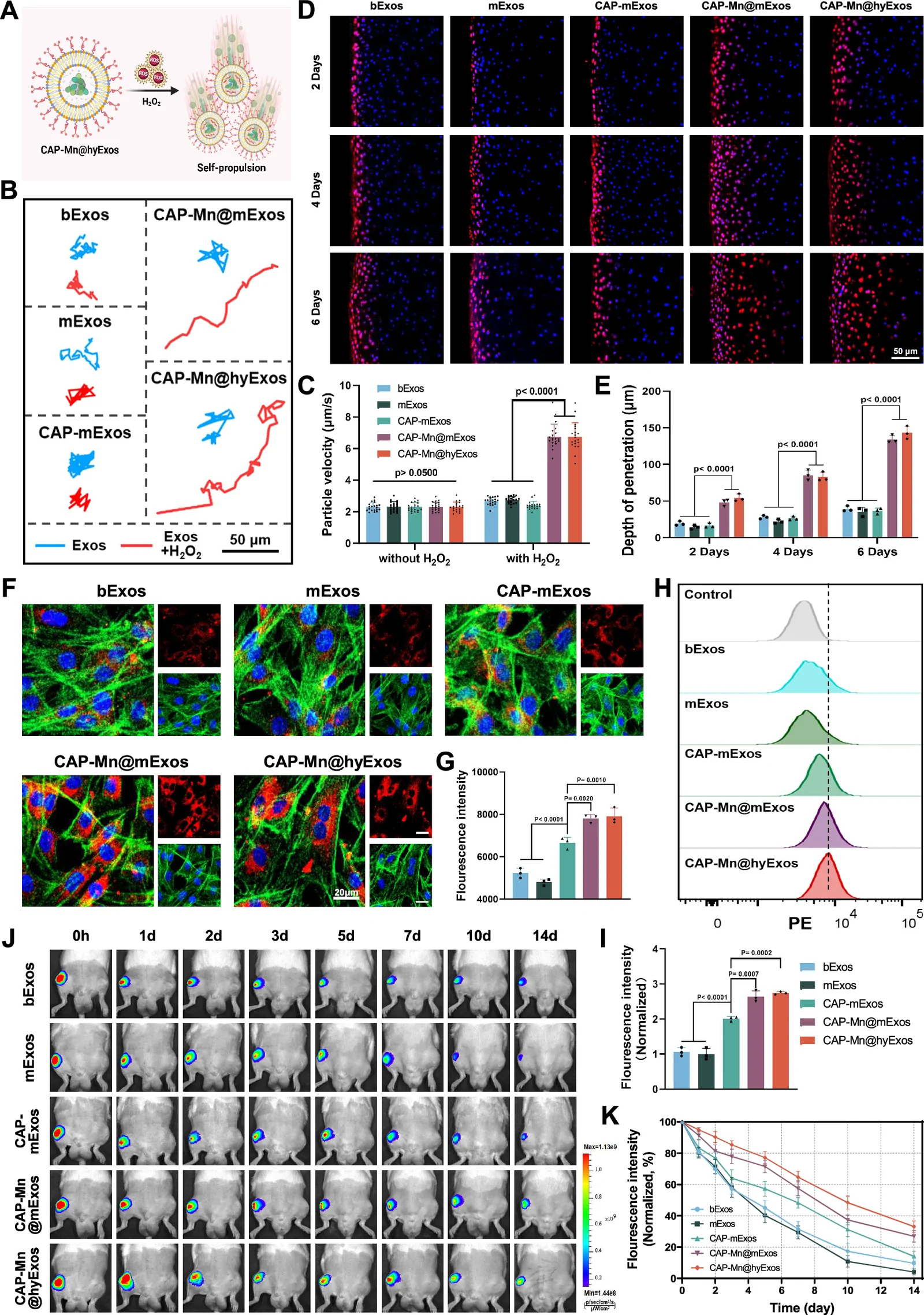
ROS-responsive exosomal nanomotors enable deep cartilage penetration, enhanced cellular uptake, and prolonged joint retention. **A** Schematic illustration of ROS-responsive motion enhancement of exosomal nanomotors in oxidative microenvironments. Created in BioRender. Huang, K. (https://BioRender.com/nqgl3ax). **B**, **C** Representative NTA tracking trajectories of exosomal formulations in the presence or absence of H₂O₂, with quantitative analysis of mean particle velocity (*n* = 20, tracked particles per group). **D**, **E** Confocal microscopy of ex vivo cartilage sections following incubation with DiI-labeled exosomal formulations for 2, 4, and 6 days, with quantitative analysis of fluorescence penetration depth, scale bar = 50 μm (*n* = 3, biological replicates per group). **F**, **G** Confocal micrographs of chondrocytes incubated with DiI-labeled exosomal formulations, with quantitative analysis of intracellular fluorescence intensity, scale bar = 20 μm (*n* = 3, biological replicates per group). **H**, **I** Flow cytometric quantification of exosome internalization by chondrocytes (*n* = 3, biological replicates per group). **J**, **K** IVIS of DiI-labeled exosomes after intra-articular injection into rat OA joints, with relative quantitative analysis of fluorescence retention over time (*n* = 3, biological replicates per group). Data are presented as the mean ± SD. *P* values are from one-way analysis of variance (ANOVA) with Tukey’s multiple comparisons test (**C**, **E**, **G**, **I**). Data in (**B**, **D**, **F**, **H**, **J**) are representative of three independent experiments with similar results. Source data are provided as a Source Data file.

The capacity of exosomal nanomotors to penetrate the dense cartilage matrix is critical for efficient intra-articular drug delivery (Fig. 3A). To characterize their dynamic motility, NTA was performed to record the trajectories of different exosomal formulations in the absence or presence of H₂O₂. In the absence of H₂O₂, all groups exhibited typical Brownian motion with limited displacement. In contrast, exposure to H₂O₂ markedly enhanced the motility of CAP-Mn@mExos and CAP-Mn@hyExos, both of which contain MnO₂, as evidenced by significantly extended trajectories and increased average velocities, indicating enhanced particle movement under an oxidative environment (Fig. 3B, C and Supplementary Fig. 17). To further assess motion behavior under a ROS-enriched microenvironment, a Y-shaped microfluidic chip was established. CAP-Mn@mExos and CAP-Mn@hyExos preferentially accumulated in the H₂O₂-containing terminal chamber, whereas exosomes without MnO₂ (mExos, bExos, and CAP-mExos) showed no discernible directional migration or enrichment in either chamber, indicating no detectable preferential migration under the tested conditions (Supplementary Fig. 18). These findings demonstrate that incorporation of the MnO₂ propulsion module confers ROS-responsive transport behavior, providing a mechanistic basis for enhanced cartilage penetration. The stimulus-selective behavior of the nanomotors was further evaluated using different microenvironmental components, including PBS, urea, glucose, and ROS-related species (H₂O₂ and O₂⁻). CAP-Mn@hyExos exhibited pronounced migration exclusively under ROS-related conditions, with preferential accumulation in the corresponding stimulated chambers, whereas negligible migration was observed under non-ROS conditions. Notably, the response to H₂O₂ was more pronounced than that to O₂⁻, indicating higher responsiveness toward H₂O₂ compared with other tested stimuli (Supplementary Fig. 19).

Based on these observations, cartilage matrix penetration capability was evaluated using an ex vivo femoral head cartilage model under H₂O₂-conditioned culture. As shown in Fig. 3D, mExos, bExos, and CAP-mExos exhibited comparable penetration depths and were largely confined to the superficial cartilage layer, indicating that CAP modification alone was insufficient to substantially enhance deep cartilage penetration. In contrast, incorporation of the MnO₂ component markedly improved cartilage penetration. Both CAP-Mn@mExos and CAP-Mn@hyExos exhibited progressive, time-dependent migration into deeper cartilage regions, with substantial fluorescence accumulation observed after 6 days. Quantitative analysis revealed that both nanomotor groups achieved a penetration depth of ~140 μm at day 6, representing a ~four-fold increase compared with non-motorized exosome groups, thereby confirming their markedly enhanced matrix penetration capability (Fig. 3E). To further distinguish the contribution of CAP-mediated matrix binding, Mn@hyExos lacking CAP modification was included as an additional control. Although Mn@hyExos exhibited substantially improved penetration compared with non-motorized exosomes, CAP-Mn@hyExos consistently achieved greater fluorescence accumulation and deeper penetration within cartilage (Supplementary Fig. 20). These results suggest that ROS-responsive motion is the primary contributor to deep cartilage penetration, whereas CAP-mediated matrix binding further enhances cartilage interaction and local accumulation once transport into cartilage has been achieved. To further characterize the spatial relationship between penetrated exosomes and chondrocytes, Z-stack confocal imaging followed by three-dimensional reconstruction and orthogonal view analysis was performed (Supplementary Fig. 21). Across multiple optical sections, DiI fluorescence remained closely associated with DAPI-positive nuclei throughout the cartilage depth, whereas minimal fluorescence was detected in nucleus-free extracellular matrix regions. While the spatial resolution of confocal microscopy does not allow definitive discrimination between intracellular and pericellular localization, the three-dimensional reconstruction consistently demonstrated close spatial association between exosomal signals and DAPI-positive chondrocytes. These observations are consistent with preferential localization of penetrated exosomes in cell-rich deep cartilage regions rather than broad retention within the extracellular matrix.

In vivo validation further corroborated these findings. Compared with healthy cartilage, OA knee cartilage exhibited structural degeneration accompanied by elevated ROS levels and altered tissue architecture (Supplementary Fig. 22). In vivo distribution analysis showed that mExos, bExos, and CAP-mExos exhibited slightly improved penetration relative to ex vivo results, while their distribution remained predominantly superficial, indicating limited ability to traverse the cartilage matrix barrier. This partial enhancement may be associated with structural changes in osteoarthritic cartilage but remains insufficient for deep tissue delivery. In contrast, CAP-Mn@mExos and CAP-Mn@hyExos showed wider spatial distribution within cartilage tissue and could be detected in deeper regions within 2 days post-injection, demonstrating improved intra-cartilage delivery efficiency compared with controls (Supplementary Fig. 23). These in vivo observations are consistent with the enhanced cartilage penetration conferred by the ROS-responsive propulsion module observed in the ex vivo model. Collectively, these findings suggest that ROS-responsive motion primarily facilitates deep cartilage penetration, whereas the comparison between Mn@hyExos and CAP-Mn@hyExos indicates that CAP-mediated matrix binding further enhances tissue interaction and local accumulation once transport into cartilage has occurred.

### Multiscale evaluation of targeting, retention, and biodistribution

To evaluate the effect of CAP modification on the chondrocyte-targeting capability of exosomes, DiI-labeled exosomal formulations were co-incubated with chondrocytes and quantitatively analyzed by confocal laser scanning microscopy (CLSM) and flow cytometry. Compared with unmodified mExos and bExos, all CAP-modified groups (CAP-mExos, CAP-Mn@mExos, and CAP-Mn@hyExos) exhibited markedly enhanced intracellular fluorescence signals, indicating significantly improved chondrocyte-targeting and uptake efficiency. Notably, intracellular fluorescence in CAP-Mn@mExos and CAP-Mn@hyExos exceeded that of CAP-mExos, suggesting that nanomotor-associated behavior may further contribute to increased cellular uptake (Fig. 3F, G). Consistently, flow cytometry revealed that the mean fluorescence intensity of these two nanomotor groups was ~2.5-fold higher than that of mExos and bExos (Fig. 3H, I), highlighting an additive effect between CAP modification and nanoparticle incorporation on cellular uptake.

To assess intra-articular retention, DiI-labeled exosomes were injected into the knee joints of OA rats and longitudinally monitored using in vivo imaging (IVIS). As shown in Fig. 3J, K, mExos and bExos were rapidly cleared, with fluorescence intensity substantially decreasing within one week. CAP-mExos exhibited moderately prolonged retention. In contrast, both CAP-Mn@mExos and CAP-Mn@hyExos demonstrated significantly extended intra-articular residence, retaining ~30% of initial fluorescence at 2 weeks post-injection. This prolonged intra-articular retention is likely associated with enhanced cartilage association and local tissue retention properties, which collectively reduce exposure to synovial clearance pathways and favor sustained localization within cartilage. Notably, clearance of CAP-Mn@hyExos was accelerated in healthy joints compared with OA joints (Supplementary Fig. 24), indicating that intra-articular retention is influenced by the pathological joint environment.

For biodistribution and metabolic profiling, major organs were harvested 3 days post-administration. Strong fluorescence signals were predominantly observed in the spleen and kidneys, with relatively weak accumulation in the liver, suggesting involvement of splenic and renal-associated clearance pathways, which may be associated with synovial vascular and lymphatic drainage. Among all groups, mExos, bExos, and CAP-mExos exhibited higher organ fluorescence than CAP-Mn@mExos and CAP-Mn@hyExos, consistent with faster systemic clearance in the absence of MnO₂ loading. At 2 weeks, fluorescence signals in major organs were markedly reduced in mExos, bExos, and CAP-mExos groups, whereas detectable signals persisted in CAP-Mn@mExos and CAP-Mn@hyExos, reflecting prolonged joint retention and slower systemic elimination. By 8 weeks, fluorescence was nearly undetectable across all organs, showing progressive systemic clearance (Supplementary Fig. 25). These results were further corroborated by ICP-OES quantification of manganese content, which demonstrated that manganese derived from CAP-Mn@hyExos was primarily distributed in joints, spleen, and kidneys, and gradually decreased over time (Supplementary Fig. 26). Given the brain as a potential target organ for manganese-associated toxicity, it was further systematically evaluated^37,38^. Throughout the observation period, neither fluorescence imaging nor ICP-OES analysis revealed abnormal manganese accumulation in brain tissue (Supplementary Fig. 25, 26). Consistently, histological examination showed no structural abnormalities, inflammatory infiltration, or pathological alterations (Supplementary Fig. 27), indicating negligible neurotoxicity and favorable systemic biosafety.

Collectively, CAP modification together with nanoparticle incorporation improves chondrocyte uptake and intra-articular retention, leading to enhanced joint accumulation and reduced systemic exposure. Importantly, the platform exhibits efficient metabolic clearance and biocompatibility, enabling multiscale optimization of exosome-based delivery from joint residence and cellular uptake to systemic fate.

### Preparation and therapeutic evaluation of 140@bExos

**Fig. 4:**
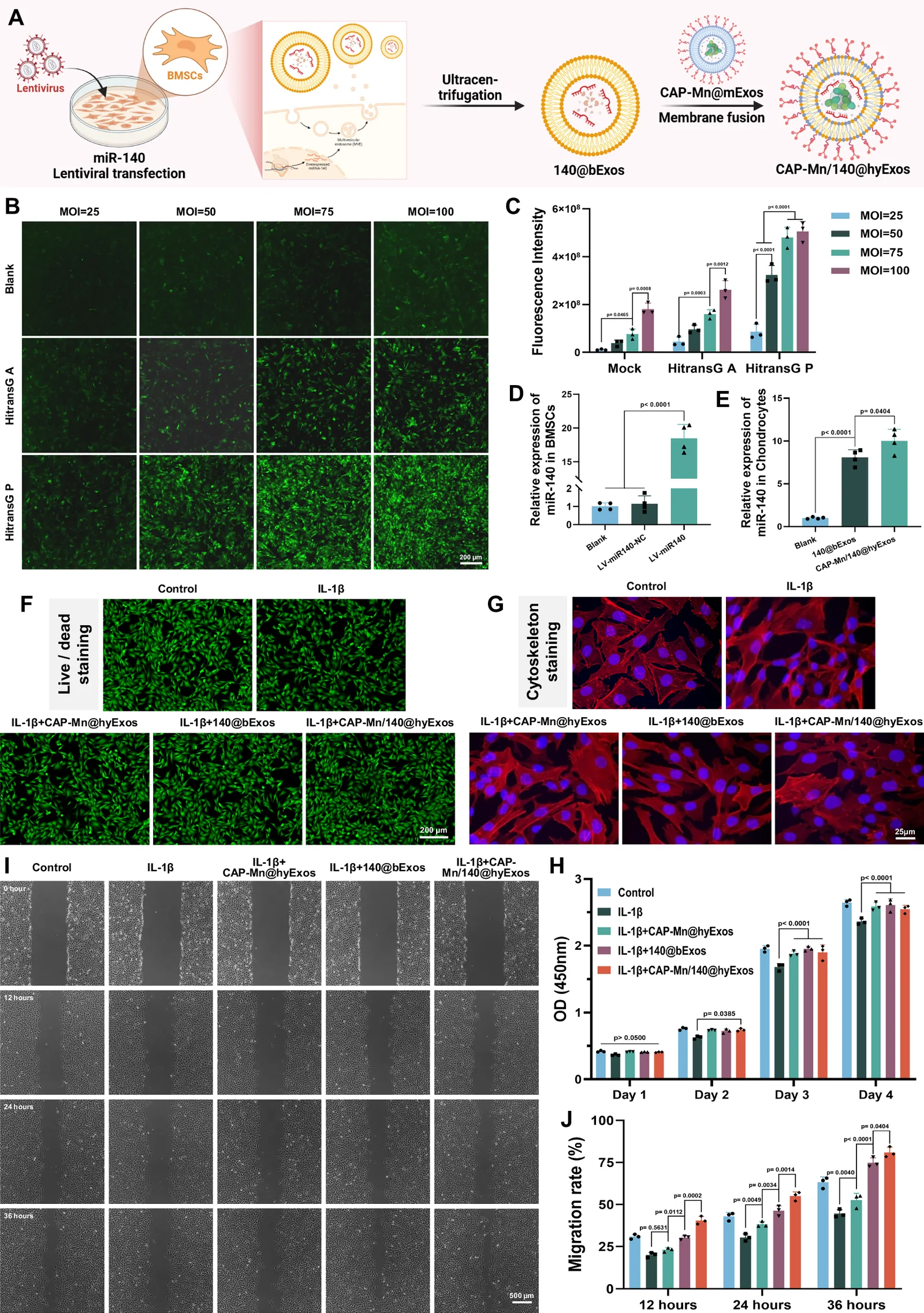
Fabrication of 140@bExos and evaluation of the biocompatibility and bioactivity of exosomal formulations. **A** Schematic illustration depicting the preparation of 140@bExos and the fabrication of CAP-Mn/140@hyExos. Created in BioRender. Huang, K. (https://BioRender.com/nqgl3ax). **B**, **C** Confocal microscopy imaging for optimization of lentiviral transduction conditions, with quantitative analysis of transduction efficiency, scale bar = 200 μm (*n* = 3, biological replicates per group). **D** qPCR analysis of *miR-140* expression levels in BMSCs (*n* = 3, biological replicates per group). **E** qPCR assessment of *miR-140* expression in chondrocytes following co-culture with 140@bExos (n = 3, biological replicates per group). **F** Representative fluorescence images of live/dead staining in OA chondrocytes treated with different exosomal formulations, scale bar = 200 μm. **G** Representative fluorescence images of cytoskeletal staining in OA chondrocytes treated with different exosomal formulations, scale bar = 25 μm. **H** CCK-8 assay assessing proliferation of OA chondrocytes treated with different exosomal formulations (*n* = 3, biological replicates per group). **I**, **J** Scratch wound assay assessing migration of OA chondrocytes treated with various exosomal formulations, with quantitative analysis of migration rate, scale bar = 500 μm (*n* = 3, biological replicates per group). Data are presented as the mean ± SD. *P* values are from one-way analysis of variance (ANOVA) with Tukey’s multiple comparisons test (**C**,** D**,** E**, **H**, **J**). Data in (**B**,** F**, **G**, **I**) are representative of three independent experiments with similar results. Source data are provided as a Source Data file.

BMSCs overexpressing miR-140 were constructed via lentiviral transduction using Lentivirus-miR-140, and 140@bExos were subsequently isolated from these modified cells (Fig. 4A). As shown in Fig. 4B, C, optimal transduction conditions were determined to be a multiplicity of infection of 100 supplemented with Hitrans A transfection reagent, which yielded maximal transduction efficiency. Quantitative polymerase chain reaction (qPCR) analysis confirmed a significant upregulation of *miR-140* expression in Lentivirus-miR-140 transfected BMSCs compared to both naive BMSCs and those transfected with Lentivirus-miR-140-NC (Fig. 4D). Moreover, co-culture of chondrocytes with 140@bExos resulted in a substantial increase in intracellular *miR-140* levels (Fig. 4E).

The therapeutic potential of 140@bExos on OA chondrocytes was evaluated through immunofluorescence, Western blotting, and qPCR. Immunofluorescence analysis revealed that, compared to the normal group, the OA control chondrocytes exhibited significantly reduced COL2A1 expression accompanied by markedly increased MMP13 and iNOS levels. Although mExos treatment produced no significant change in these markers compared to the OA control, bExos administration led to a modest increase in COL2A1 and decrease in MMP13 and iNOS. In contrast, 140@bExos treatment markedly increased COL2A1 expression and reduced MMP13 and iNOS expression (Supplementary Fig. 28). These findings were consistently supported by Western blotting and qPCR analyses across all experimental groups (Supplementary Fig. 29, 30). Additionally, Alcian blue and Safranin O staining revealed weak staining in both OA control and mExos groups, moderate intensity in the bExos group, and the strongest staining in the 140@bExos group (Supplementary Fig. 31). In summary, mExos showed minimal effect on OA chondrocyte phenotype, bExos showed intermediate effects, and 140@bExos exhibited the most pronounced restorative effect on chondrocyte matrix-associated phenotype.

### Exosomal Nanomotors Exhibit Biocompatibility and Bioactivity

Hybrid exosomal nanomotors, designated as CAP-Mn/140@hyExos, were developed via membrane fusion of 140@bExos and CAP-Mn@mExos (Fig. 4A). The biocompatibility of the exosomal nanomotor system was comprehensively assessed through live/dead staining, hemolysis assay, cytoskeletal staining, CCK-8 proliferation assay, and scratch healing assay. Live/dead staining demonstrated negligible cytotoxicity across all exosome groups (Fig. 4F). Corroborating these results, hemolysis assays revealed no detectable hemolytic activity in any exosomal formulation, confirming their biocompatibility (Supplementary Fig. 32). Cytoskeletal staining illustrated that normal chondrocytes maintained a characteristic polygonal morphology, while IL-1β-induced OA chondrocytes adopted an irregular, elongated spindle shape. Intervention with CAP-Mn@hyExos and 140@bExos partially restored cellular morphology, with the CAP-Mn/140@hyExos exhibiting the most substantial recovery toward the normal phenotypic appearance (Fig. 4G). CCK-8 assay demonstrated that treatment with CAP-Mn@hyExos, 140@bExos, and CAP-Mn/140@hyExos significantly enhanced the proliferation of OA chondrocytes compared to the IL-1β-induced OA control group (Fig. 4H). Scratch healing assays further indicated that all exosomal formulations promoted cellular migration to differing degrees. Notably, CAP-Mn@hyExos induced a moderate migratory response, 140@bExos produced an intermediate effect, while CAP-Mn/140@hyExos exhibited the most greatest pro-migratory capability among all tested formulations (Fig. 4I, J).

### Exosomal nanomotors demonstrate antioxidant capacity

**Fig. 5:**
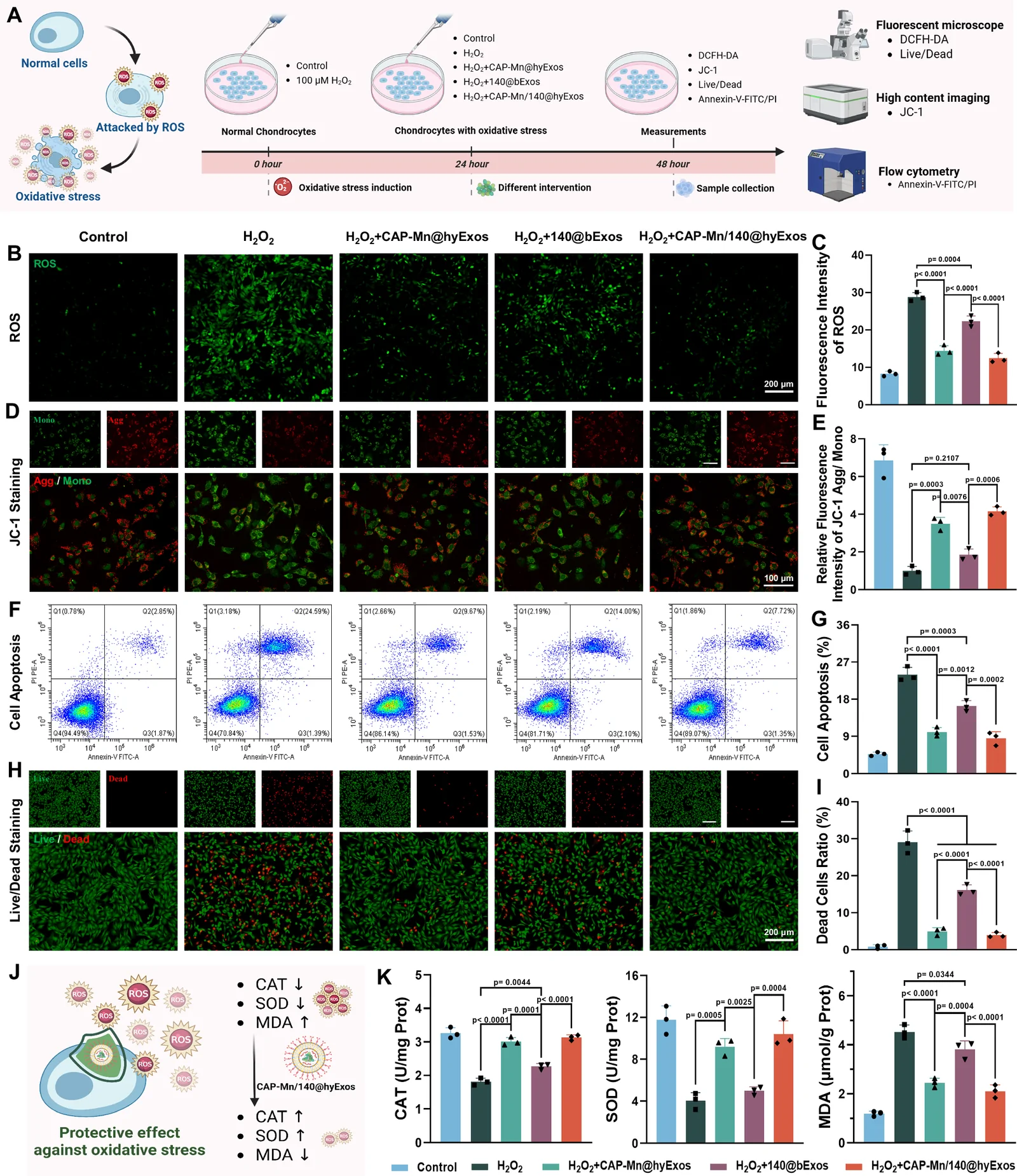
Exosomal nanomotors demonstrate antioxidant capacity. **A** Schematic diagram of the H₂O₂-induced oxidative stress model and corresponding experimental workflow. Created in BioRender. Huang, K. (https://BioRender.com/nqgl3ax). **B**, **C** DCFH-DA staining for intracellular ROS detection in chondrocytes treated with different exosomal formulations, with quantitative analysis, scale bar = 200 μm (*n* = 3, biological replicates per group). **D**,** E** JC-1 staining for evaluation of mitochondrial membrane potential, with relative quantitative analysis, scale bar = 100 μm (*n* = 3, biological replicates per group). **F**, **G** Annexin V/PI staining for apoptosis detection by flow cytometry, with quantitative analysis of apoptotic rates (*n* = 3, biological replicates per group). **H**,** I** Live/dead staining of chondrocytes treated with different exosomal formulations, with quantitative analysis of cell viability, scale bar = 200 μm (*n* = 3, biological replicates per group). **J** Schematic illustration of oxidative stress-associated biomarkers evaluated in chondrocytes. Created in BioRender. Huang, K. (https://BioRender.com/nqgl3ax). **K** Quantitative analysis of intracellular SOD and CAT activities and MDA levels in chondrocytes treated with different exosomal formulations (n = 3, biological replicates per group). Data are presented as the mean ± SD. *P* values are from one-way analysis of variance (ANOVA) with Tukey’s multiple comparisons test (**C**, **E**,** G**,** I**,** K**). Data in (**B**,** D**,** F**,** H**) are representative of three independent experiments with similar results. Source data are provided as a Source Data file.

During the progression of OA, chondrocytes are chronically exposed to elevated ROS, resulting in mitochondrial dysfunction, increased apoptosis, and accelerated extracellular matrix degradation. Exogenous H₂O₂ treatment partially mimics oxidative stress-related pathological features and is therefore widely used to establish oxidative stress models in vitro^39,40^. To determine an appropriate working concentration, chondrocytes were first exposed to increasing concentrations of H₂O₂, and cell viability was evaluated by CCK-8 assay (Supplementary Fig. 33). H₂O₂ treatment induced a concentration-dependent reduction in cell viability. Based on the IC₅₀-guided viability analysis, 100 μM H₂O₂ was selected as the working concentration because it induced a stable oxidative stress state while maintaining sufficient cell viability for subsequent functional evaluation. Accordingly, an H₂O₂-induced oxidative stress model was established using 100 μM H₂O₂ to evaluate the ROS-responsive behavior and antioxidant effects of CAP-Mn/140@hyExos (Fig. 5A).

DCFH-DA staining indicated that H₂O₂ stimulation significantly increased intracellular ROS levels in chondrocytes. While 140@bExos treatment moderately reduced ROS levels, both CAP-Mn@mExos and CAP-Mn/140@hyExos markedly attenuated ROS accumulation, suggesting that MnO₂-containing formulations contribute to attenuation of intracellular ROS levels (Fig. 5B, C). JC-1 staining revealed that H₂O₂ induction caused a pronounced reduction in mitochondrial membrane potential, which was effectively restored following treatment with CAP-Mn@hyExos and CAP-Mn/140@hyExos (Fig. 5D, E). Apoptosis assay further demonstrated that H₂O₂ significantly increased both early and late apoptotic rates. This effect was partially ameliorated by 140@bExos, while CAP-Mn@hyExos and CAP-Mn/140@hyExos interventions substantially reduced total apoptosis rates (Fig. 5F, G). Consistent with these observations, live/dead staining indicated diminished cell viability in the H₂O₂ control group, whereas both CAP-Mn@hyExos and CAP-Mn/140@hyExos significantly increased the proportion of viable cells, confirming improved cellular survival (Fig. 5H, I). Additionally, key oxidative stress-related indicators, including intracellular activities of catalase (CAT), total superoxide dismutase (SOD), and malondialdehyde (MDA) content, were comprehensively evaluated to assess cellular redox status (Fig. 5J). The results revealed that CAP-Mn@hyExos and CAP-Mn/140@hyExos treatments significantly enhanced SOD and CAT activities while reducing MDA levels, indicating attenuation of oxidative stress–associated damage (Fig. 5K). In summary, CAP-Mn@hyExos and CAP-Mn/140@hyExos confer protection against H₂O₂-induced oxidative injury in chondrocytes through modulation of intracellular ROS levels, preservation of mitochondrial function, suppression of apoptosis, and improvement of antioxidant enzyme activity profiles.

### Exosomal nanomotors facilitate functional restoration of OA chondrocytes

**Fig. 6:**
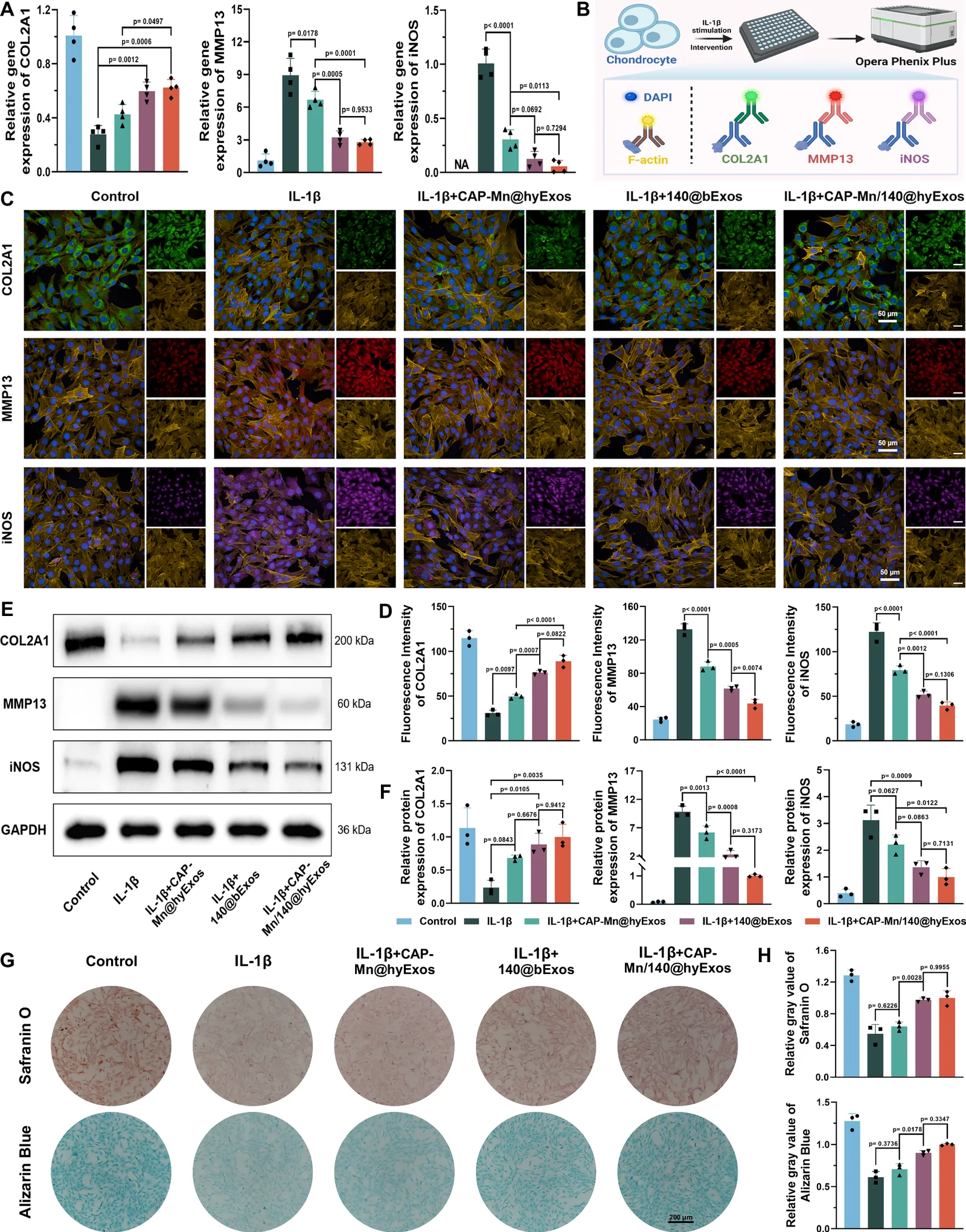
Exosomal nanomotors facilitate functional restoration of OA chondrocytes. **A** qPCR analysis of *COL2a1*, *MMP13*, and *iNOS* gene expression levels (*n* = 4, biological replicates per group). **B**–**D** Schematic illustration and representative immunofluorescence images of COL2A1, MMP13, and iNOS, with corresponding quantitative analysis, scale bar = 50 μm (*n* = 3, biological replicates per group). Created in BioRender. Huang, K. (https://BioRender.com/nqgl3ax). **E**,** F** Western blot analysis of COL2A1, MMP13, and iNOS protein levels, with relative quantitative analysis (*n* = 3, biological replicates per group). **G**, **H** Safranin O and Alcian blue staining of OA chondrocytes treated with different exosomal formulations, with related quantitative analysis, scale bar = 200 μm (*n* = 3, biological replicates per group). Data are presented as the mean ± SD. *P* values are from one-way analysis of variance (ANOVA) with Tukey’s multiple comparisons test (**A**, **D**, **F**,** H**). Data in (**C**,** E**,** G**) are representative of three independent experiments with similar results. Source data are provided as a Source Data file.

Articular cartilage in OA condition exhibits a pronounced decline in anabolic activity, exacerbated catabolism, and excessive inflammatory mediator release. To evaluate the therapeutic potential of CAP-Mn/140@hyExos on OA chondrocytes in vitro, the expression levels of representative markers including COL2A1, MMP13, and iNOS were examined. qPCR analyses demonstrated that CAP-Mn@hyExos treatment moderately enhanced *COL2a1* expression while reducing *MMP13* and *iNOS* levels. In comparison, both 140@bExos and CAP-Mn/140@hyExos interventions induced substantial recovery of *COL2a1* expression and significant inhibition of *MMP13* and *iNOS*, with CAP-Mn/140@hyExos yielding the most pronounced effects on OA-associated gene expression profiles (Fig. 6A). These observations were corroborated by immunofluorescence and Western blot analyses, which confirmed that 140@bExos and CAP-Mn/140@hyExos treatments significantly upregulated COL2A1 at protein levels, while concurrently downregulating MMP13 and iNOS, indicating a coordinated enhancement of extracellular matrix homeostasis and mitigation of inflammatory-related signaling in OA chondrocytes (Fig. 6B–F). Furthermore, Alcian blue and Safranin O staining revealed a substantial reduction in proteoglycan deposition in OA chondrocytes compared to normal controls. In contrast, intervention with 140@bExos and CAP-Mn/140@hyExos markedly intensified staining, indicating enhanced extracellular matrix biosynthesis (Fig. 6G, H). Collectively, these findings demonstrate that CAP-Mn/140@hyExos effectively alleviates pathological alterations and promotes recovery of OA-associated chondrocyte phenotype through combined effects of enhanced miR-140 delivery and microenvironment-responsive transport behavior.

### Exosomal nanomotors ameliorate gait patterns and restore subchondral bone microarchitecture in knee OA rats

**Fig. 7:**
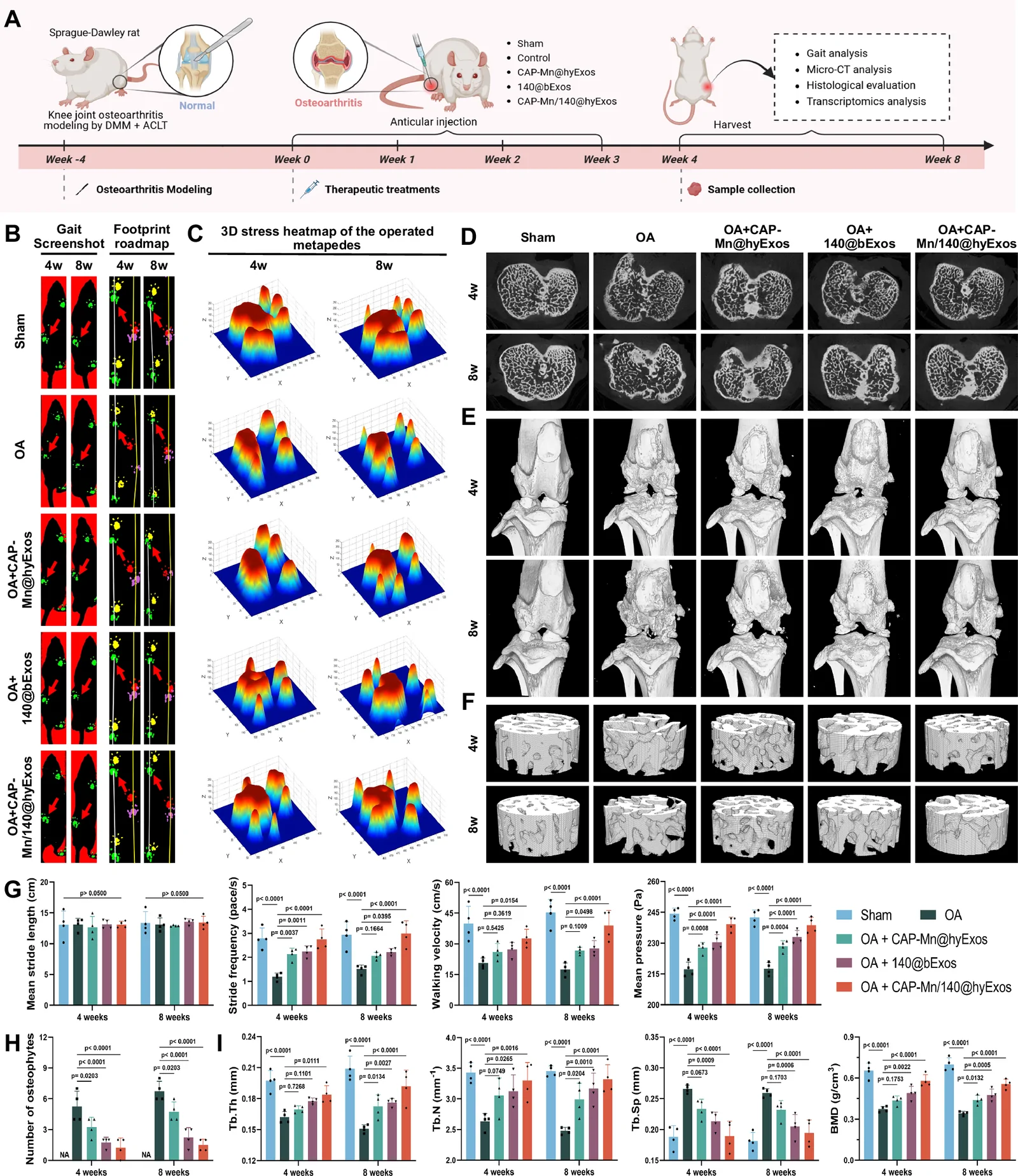
Exosomal nanomotors ameliorate gait patterns and restore subchondral bone microarchitecture in knee OA rats. **A** Schematic timeline of experimental treatment in the OA rat model. Created in BioRender. Huang, K. (https://BioRender.com/nqgl3ax). **B** Representative gait screenshots and footprint roadmap of OA rats (Red arrows indicate the footprints of the operated limb). **C** 3D stress heatmap of the operated metapedes. **D** Horizontal micro-CT view of tibial subchondral bone microstructure. **E**,** H** Three-dimensional reconstructions of the affected knee joint and quantitative analysis of osteophyte number (*n* = 4, biological replicates per group). **F** Three-dimensional morphometric analysis of tibial plateau subchondral bone microarchitecture. **G** Quantitative gait parameter analysis including stride length, frequency, velocity, and plantar pressure (*n* = 4, biological replicates per group). **I** Quantitative micro-CT analysis of tibial subchondral bone for BMD, Tb.N, Tb.Th, and Tb.Sp (*n* = 4, biological replicates per group). Data are presented as the mean ± SD. *P* values are from one-way analysis of variance (ANOVA) with Tukey’s multiple comparisons test (**G**–**I**). Data in (**B**–**F**) are representative of four independent experiments with similar results. Source data are provided as a Source Data file.

Following the confirmation of the therapeutic potential of exosomal nanomotors for OA chondrocytes in vitro, we further evaluated their efficacy in a rat knee OA model (Fig. 7A). Gait analysis represents an essential method for evaluating motor impairments and was used to assess the effects of CAP-Mn/140@hyExos on locomotor performance. No statistically significant intergroup differences were observed in mean stride length. Although both CAP-Mn@hyExos and 140@bExos administration yielded partial improvements in stride frequency and velocity among OA rats, CAP-Mn/140@hyExos treatment produced the most substantial enhancement in these gait parameters. Notably, compared to the Sham group, OA animals demonstrated significantly diminished plantar pressure in the affected limb. Therapeutic intervention with CAP-Mn@hyExos, 140@bExos, and CAP-Mn/140@hyExos significantly improved plantar pressure distribution, with the most pronounced effect observed in the CAP-Mn/140@hyExos group (Fig. 7B, C and G).

Further evaluation via micro-computed tomography (micro-CT) scanning of the affected knee joints revealed subchondral bone fissures accompanied by localized sclerosis in OA specimens, along with sparse and disorganized trabecular architecture (Fig. 7D and Supplementary Fig. 34). Three-dimensional reconstructions demonstrated irregular articular surfaces, significant joint space narrowing, and osteophyte formation with loose bodies. Interventions with CAP-Mn@hyExos, 140@bExos, and CAP-Mn/140@hyExos partially improved subchondral bone structure and trabecular organization, while reducing osteophyte formation and loose body incidence, with CAP-Mn/140@hyExos administration showing the most pronounced structural improvements (Fig. 7E, F and H). Moreover, quantitative analysis of subchondral bone microstructural parameters in the tibial plateau showed that, compared to the Sham group, OA animals exhibited significantly decreased bone mineral density (BMD), trabecular number (Tb.N), and trabecular thickness (Tb.Th), along with increased trabecular separation (Tb.Sp). All therapeutic interventions partially restored BMD, Tb.N, and Tb.Th while reducing Tb.Sp, with the CAP-Mn/140@hyExos group demonstrating the most effective improvement across all measured parameters (Fig. 7I).

### Exosomal nanomotors facilitate cartilage repair and attenuate disease progression in knee OA rats

**Fig. 8:**
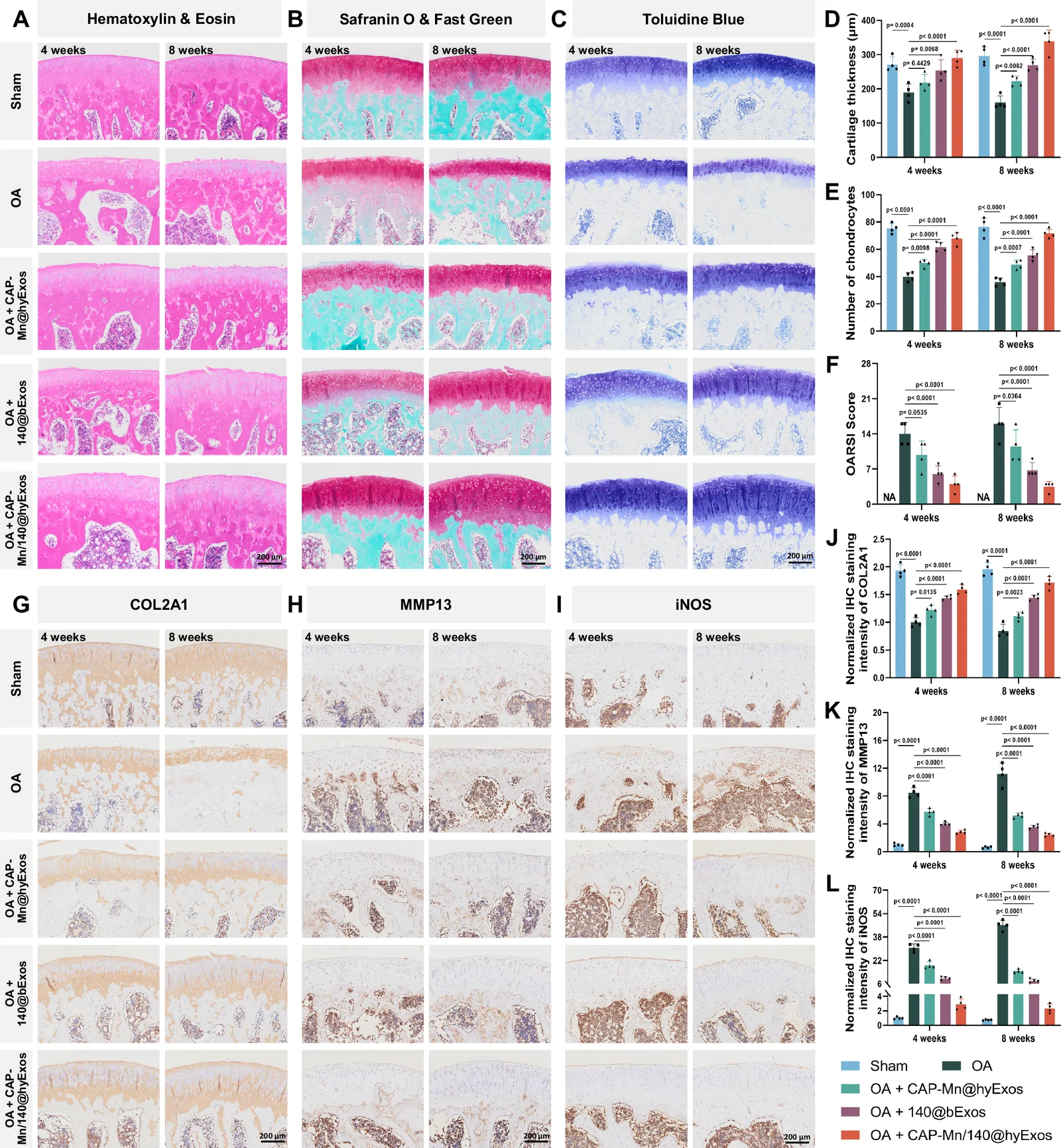
Exosomal nanomotors facilitate cartilage repair and attenuate disease progression in knee OA rats. **A**–**C** Representative histological staining of the affected OA joint treated with different exosomal formulations, including Hematoxylin & Eosin (**A**), Safranin O & Fast Green (**B**), and Toluidine Blue (**C**), scale bar = 200 μm. **D**–**F** Quantitative analysis of cartilage thickness (**D**), chondrocyte number (**E**), and OARSI score (**F**) (*n* = 4, biological replicates per group). **G**–**I** Representative immunohistochemical staining of COL2A1 (**G**), MMP13 (**H**), and iNOS (**I**) in cartilage tissues, scale bar = 200 μm. **J**–**L** Quantitative analysis of immunohistochemical expression of COL2A1 (**J**), MMP13 (**K**), and iNOS (**L**) (*n* = 4, biological replicates per group). Data are presented as the mean ± SD. *P* values are from one-way analysis of variance (ANOVA) with Tukey’s multiple comparisons test (**D**–**E**,** J**–**L**), or non-parametric tests (Kruskal-Wallis followed by two-tailed Mann-Whitney *U* test) (**F**). Data in (**A**–**C**,** G**–**I**) are representative of four independent experiments with similar results. Source data are provided as a Source Data file.

Initial comparative analysis of knee joint gross morphology across experimental groups revealed distinctive morphological features. As depicted in Supplementary Fig. 35, the Sham group presented smooth, intact, and translucent articular cartilage with a glistening surface. In contrast, OA group specimens exhibited pronounced surface irregularity, abnormal discoloration, focal erosion, and subchondral bone exposure. CAP-Mn@hyExos intervention partially restored articular cartilage architecture, while more substantial morphological recovery was observed in both 140@bExos and CAP-Mn/140@hyExos treatment groups, with the latter demonstrating better macroscopic outcomes. Histological staining revealed that OA cartilage manifested surface fissuring, thinning of the cartilage, reduced chondrocyte density, disorganized cellular arrangement, diminished matrix-specific staining, and disruption of the tidemark structure compared to the Sham group. All exosomal interventions attenuated cartilage degeneration and significantly reduced Osteoarthritis Research Society International (OARSI) scores, indicating amelioration of OA pathology. Notably, the CAP-Mn/140@hyExos group exhibited the most substantial therapeutic response, with regenerated cartilage thickness approaching or exceeding that of the Sham group (Fig. 8A–F). Immunohistochemical staining revealed significantly reduced COL2A1 expression alongside elevated MMP13 and iNOS levels in OA cartilage, indicative of enhanced matrix degradation and inflammatory activation. CAP-Mn/140@hyExos treatment effectively restored COL2A1 expression while suppressing MMP13 and iNOS, suggesting recovery toward cartilage homeostasis (Fig. 8G-L).

Finally, systemic biosafety assessment was conducted via Hematoxylin and Eosin (H&E) staining of major organs. No significant alterations in hematological parameters or hepatic and renal function biomarkers were observed across treatment groups (Supplementary Fig. 36). Additionally, histological staining revealed no notable architectural distortions or pathological modifications in vital organs (Supplementary Fig. 37), corroborating the favorable biocompatibility of exosomal nanomotors and supporting their potential for translational application in OA therapy.

### Transcriptomic analysis reveals the underlying mechanisms of exosomal nanomotors in OA treatment

**Fig. 9:**
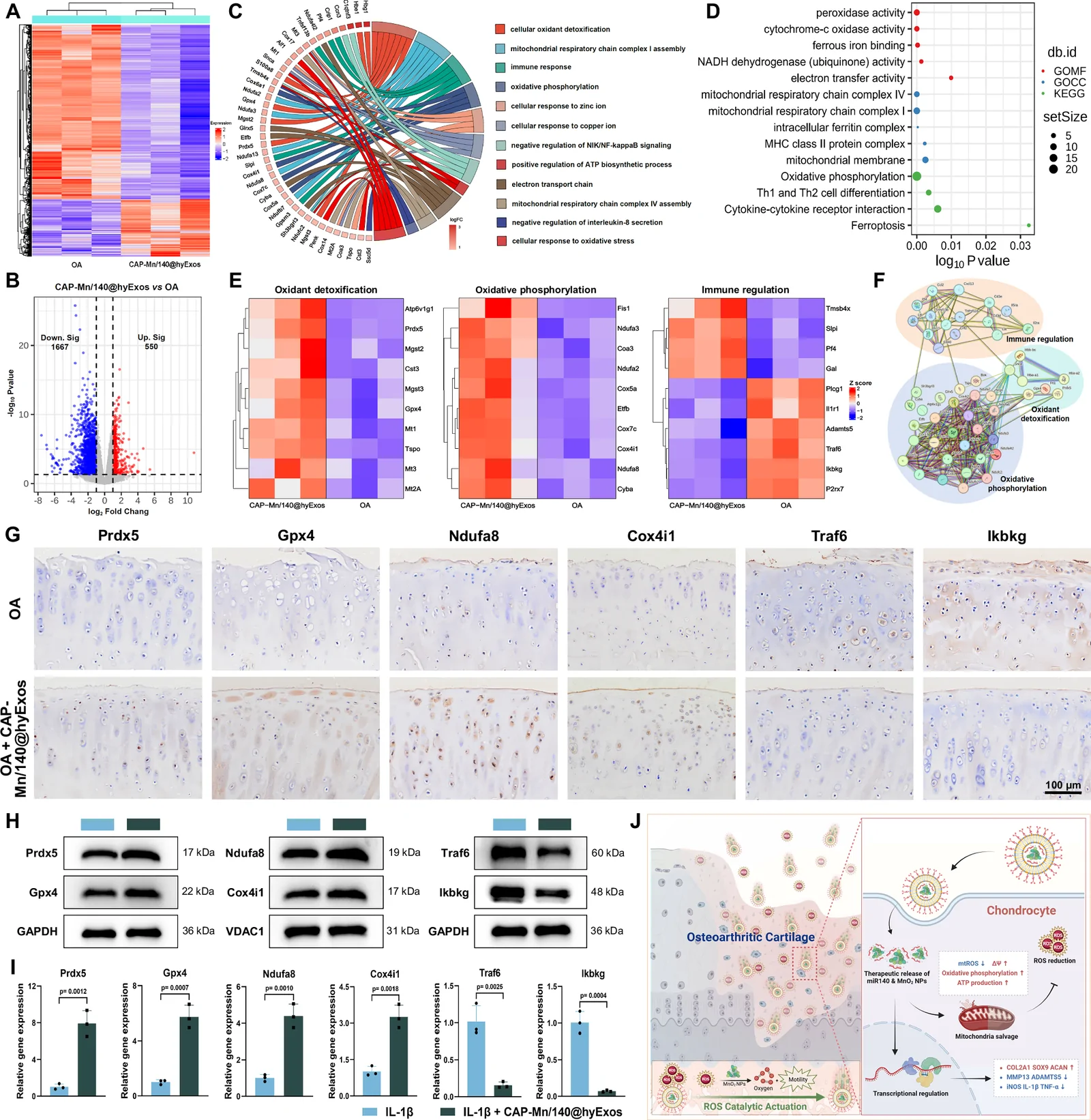
Transcriptomic analysis reveals the underlying mechanisms of exosomal nanomotors in OA treatment. **A** Hierarchical clustering of global gene expression patterns in OA control and CAP-Mn/140@hyExos-treated cartilage. **B** Volcano plot showing DEGs between the two groups, where differential expression analysis was performed using DESeq2 (*P* < 0.05, |log₂FC | > 1). **C** Chord diagram of significantly enriched Biological Process (GO) terms and their associated DEGs. **D** Bubble plot summarizing enriched Molecular Function and Cellular Component (GO) terms and KEGG pathways, where GO and KEGG enrichment analyses were performed using clusterProfiler. **E** Heatmap of selected DEGs involved in pathways related to oxidative detoxification, oxidative phosphorylation, and immune modulation. **F** Protein-protein interaction network of proteins encoded by the selected DEGs. **G** Immunohistochemical analysis of representative proteins corresponding to selected DEGs from major pathways in OA cartilage following CAP-Mn/140@hyExos treatment, scale bar = 100 μm. **H** Western blot analysis of the same proteins in chondrocyte models. **I** qPCR validation of the same genes in chondrocyte models (*n* = 3, biological replicates per group). **J** Schematic illustration of the proposed mechanism of CAP-Mn/140@hyExos in OA therapy. Created in BioRender. Huang, K. (https://BioRender.com/nqgl3ax). Data are presented as mean ± SD. *P* values are from unpaired two-tailed Student’s *t* test (**I**). Data in (**G**, **H**) are representative of three independent experiments with similar results. Source data are provided as a Source Data file.

To elucidate the mechanisms underlying CAP-Mn/140@hyExos-mediated cartilage regeneration and repair in OA, transcriptomic analysis was conducted at 8 weeks post-administration. Initial assessment of data quality via principal component analysis (PCA) and Pearson correlation demonstrated a cumulative variance contribution of 80% for PC1 and PC2 (Supplementary Fig. 38), with intra-group correlation coefficients consistently exceeding R² = 0.95 (Supplementary Fig. 39). These metrics confirm substantial inter-group differentiation and intra-group reproducibility, establishing a solid foundation for subsequent analytical validity. Hierarchical clustering analysis demonstrated distinct segregation of gene expression profiles between CAP-Mn/140@hyExos-treated and OA control cartilage (Fig. 9A). Volcano plot analysis identified 550 upregulated and 1667 downregulated differentially expressed genes (DEGs), suggesting the engagement of an integrated multi-gene regulatory network during cartilage repair and microenvironmental remodeling (Fig. 9B).

Further Gene Ontology (GO) and Kyoto Encyclopedia of Genes and Genomes (KEGG) enrichment analyses were performed on these DEGs. Within the GO biological process category, DEGs exhibited significant enrichment in pathways related to oxidative detoxification (e.g., cellular oxidant detoxification, cellular response to zinc ion, cellular response to copper ion, cellular response to oxidative stress), oxidative phosphorylation (e.g., mitochondrial respiratory chain complex I assembly, oxidative phosphorylation, positive regulation of ATP biosynthetic process, electron transport chain, mitochondrial respiratory chain complex IV assembly), and immune regulation (e.g., immune response, negative regulation of NIK/NF-kappaB signaling, negative regulation of interleukin-8 secretion) (Fig. 9C). Consistent findings were obtained from GO molecular function, cellular component, and KEGG enrichment analyses (Fig. 9D), highlighting the broad regulatory role of CAP-Mn/140@hyExos across multiple pathological and restorative processes. Heatmap visualization affirmed marked differential expression of pivotal genes between groups (Fig. 9E), supporting the enhancement of oxidative detoxification (e.g., Prdx5, Gpx4, Mgst2, Mgst3, Mt1), promotion of mitochondrial oxidative phosphorylation and energy metabolism (e.g., Ndufa8, Cox4i1, Cox5a, Etfb, Cyba), and anti-inflammatory immune regulation (e.g., Traf6, Ikbkg, Slpi, Pf4, Adamts5) upon treatment. Protein-protein interaction network analysis further categorized the DEGs into three functional clusters, including oxidative detoxification, oxidative phosphorylation, and immune modulation, which corroborated the prior enrichment results (Fig. 9F).

To validate these transcriptomic findings, key genes associated with oxidative detoxification, mitochondrial oxidative phosphorylation, and anti-inflammatory immune regulation were chosen and examined at the molecular level (Supplementary Fig. 40). Immunohistochemical analysis showed that, compared with OA controls, CAP-Mn/140@hyExos treatment markedly upregulated Prdx5 and Gpx4, indicating enhanced endogenous antioxidant capacity and mitigation of ROS-induced oxidative stress. Mitochondrial oxidative phosphorylation genes Ndufa8 and Cox4i1 were also significantly restored, reflecting improved energy metabolism and mitochondrial function, thereby supporting cellular metabolic activity and cartilage homeostasis. In parallel, anti-inflammatory regulatory genes Traf6 and Ikbkg were downregulated, demonstrating effective suppression of NIK/NF-κB signaling and inflammatory mediator release, ultimately improving the local immune microenvironment (Fig. 9G). Western blot and qPCR analyses at the cellular level were consistent with immunohistochemistry results, confirming coordinated modulation of these three pathways by CAP-Mn/140@hyExos (Fig. 9H, I). Importantly, these gene expression changes were also observed in deep cartilage layers, further supporting the efficient intratissue distribution and microenvironment-responsive action of the hybrid exosomal nanomotors. Collectively, CAP-Mn/140@hyExos coordinate the enhancement of oxidative detoxification, restoration of mitochondrial energy metabolism, and suppression of inflammatory responses, establishing a multi-pathway synergistic mechanism that preserves chondrocyte homeostasis and promotes functional repair (Fig. 9J). These findings provide a molecular framework for understanding the therapeutic efficacy of exosomal nanomotors in OA.

## Discussion

The therapeutic efficacy of intra-articular drug delivery in OA is constrained by multiple interdependent biological barriers, including rapid joint clearance, the densely packed cartilage extracellular matrix, and inherently limited chondrocyte accessibility^41^. Importantly, these limitations fundamentally arise from diffusion-dominated transport within cartilage, where restricted intratissue movement directly limits cellular access and therapeutic efficacy. Rather than acting independently, these barriers form a hierarchically coupled system in which impaired matrix transport suppresses cellular uptake, while insufficient joint retention further limits effective tissue interactions^13^. As a result, improving a single parameter alone rarely translates into meaningful therapeutic gains, underscoring the necessity of a coordinated strategy that actively regulates transport behavior across multiple biological scales^42^. To address this challenge, we developed a ROS-responsive hybrid exosomal nanomotor system that converts pathological microenvironmental signals to control intratissue transport. Unlike conventional diffusion-driven delivery systems, this platform harnesses elevated ROS levels in osteoarthritic joints as a functional energy source. In particular, MnO₂-mediated catalytic decomposition of H₂O₂ generates oxygen while alleviating oxidative stress, and concomitantly induces reaction-coupled enhancement of particle motion under ROS-rich environments. In this manner, ROS is converted from a pathological byproduct into a transport-driving regulator, enabling enhanced intratissue redistribution beyond diffusion-limited behavior.

In ex vivo cartilage models, this transport reprogramming manifests as markedly enhanced intratissue distribution, with penetration depths reaching ~140 μm, far exceeding those of conventional exosomal systems. Notably, such distribution cannot be fully explained by passive diffusion or size-dependent permeability alone. Instead, it arises from the synergy between ROS-responsive motility enhancement and the inherent deformability of the exosomal lipid bilayer, which together facilitate traversal through the densely crosslinked extracellular matrix^43^. Mechanistically, tissue penetration and targeting are not separable events but emerge from a coupled transport–confinement effect. CAP modification enhances interactions with the cartilage extracellular matrix, resulting in localized spatial confinement that prolongs intratissue residence^44,45^. Under these confined conditions, ROS-driven motion is transformed from stochastic diffusion into biased transport behavior, resulting in preferential accumulation within ROS-enriched diseased cartilage. At the tissue scale, this distribution exhibits a chemotaxis-like spatial redistribution behavior. However, this phenomenon does not rely on classical receptor-mediated long-range gradient sensing or active directional chemotactic signaling^46,47^. Instead, it is better described as a ROS-responsive, motility-biased transport process, arising from the coupling between local redox reactions and spatial confinement within the cartilage extracellular matrix, ultimately resulting in emergent biased intratissue distribution.

This transport-programmed spatial distribution further enhances cellular delivery while coupling intracellular regulation with microenvironmental remodeling. CAP-mediated retention increases both the frequency and duration of nanocarrier-chondrocyte interactions, thereby facilitating endocytosis and improving intracellular delivery efficiency. As a result, miR-140 is more effectively delivered, leading to coordinated regulation of cartilage-associated pathways, as evidenced by increased COL2A1 expression and decreased MMP13 and iNOS levels. In parallel, MnO₂-mediated catalytic decomposition of H₂O₂ reduces oxidative stress while improving local oxygen availability, thereby reshaping the redox and metabolic microenvironment. Transcriptomic analysis reveals coordinated enrichment of pathways associated with oxidative detoxification, oxidative phosphorylation, and immune regulation. Upregulation of oxidative phosphorylation-related genes such as Ndufa8 and Cox4i1 suggests partial restoration of mitochondrial activity, while modulation of immune-related pathways indicates a shift toward a less pro-inflammatory state. Rather than acting independently, these processes are unified under transport-regulated delivery behavior, whereby enhanced distribution and microenvironmental modulation synergistically promote cartilage repair.

More broadly, these findings expand current strategies for intra-articular delivery, which has predominantly relied on prolonging retention or improving formulation stability while leaving transport behavior largely diffusion-dominated^48,49^. In contrast, this work demonstrates that intratissue distribution can be actively regulated through microenvironmental feedback, enabling transport to be controlled rather than passively governed by diffusion. On this basis, we propose a conceptual framework in which pathological signals are not merely therapeutic targets but regulators of delivery behavior. Within this framework, ROS serves a dual role as both pathological mediator and transport-driving cue, while tissue-specific binding not only enhances targeting specificity but also reshapes spatial distribution through confinement effects. By integrating reaction-coupled motion, confinement, and cellular regulation into a unified system, this strategy provides a microenvironment-responsive delivery framework in which distribution is actively regulated rather than diffusion-limited, providing a generalizable design principle for complex tissue-targeted therapies.

Although this study establishes a framework for overcoming multiple biological barriers, several aspects remain to be further refined. The current system still relies on finite intra-articular residence, and sustained retention strategies may further improve long-term efficacy. In addition, while transcriptomic and phenotypic analyses provide converging evidence of therapeutic benefit, the precise coupling mechanisms between ROS-driven transport behavior and downstream cellular and microenvironmental responses remain to be fully elucidated. Finally, long-term biosafety and immunological responses under repeated administration require systematic evaluation to facilitate clinical translation.

In summary, we engineered a ROS-responsive hybrid exosomal nanomotor system (CAP-Mn/140@hyExos) that integrates reaction-coupled motion, matrix-targeted interactions, and intracellular delivery to overcome multiple biological barriers in osteoarthritic cartilage. By converting pathological ROS into transport-driving cues and leveraging CAP-mediated spatial confinement, the platform actively regulates intratissue transport behavior, thereby enhancing cartilage accumulation and overcoming the limitations of passive diffusion within cartilage. The resulting transport-regulated delivery enables coordinated intracellular delivery and microenvironmental remodeling, ultimately alleviating oxidative stress, promoting cartilage regeneration, and slowing OA progression. Collectively, these findings demonstrate that pathological microenvironments can be harnessed as functional regulators of transport behavior rather than merely therapeutic targets. This work establishes a microenvironment-responsive, transport-regulated delivery paradigm for drug delivery in transport-restricted tissues and provides a design principle for microenvironment-responsive drug delivery.

## Methods

### Ethical statement

All animal experiments were conducted in accordance with all relevant ethical regulations. The study protocol was approved by the Animal Ethics Committee of West China Hospital, Sichuan University (Approval No. 20250923002).

### Materials

The principal reagents and commercial sources used in this study are as follows. α-MEM medium, DMEM/F12 (1:1) medium, fetal bovine serum (FBS), penicillin, streptomycin, and trypsin were purchased from Gibco, USA. Collagenase type II and recombinant interleukin-1β (IL-1β) were obtained from Sigma-Aldrich, USA. Skimmed milk powder was purchased from Yili Co., China. Manganese chloride, bovine serum albumin (BSA), potassium bromide (KBr), 1% acetic acid, and hydrogen peroxide (H₂O₂) were purchased from Macklin, China. DSPE-PEG_2000_-MAL and the CAP peptide were obtained from ChinaPeptides, China. Phosphate-buffered saline (PBS), dimethyl sulfoxide (DMSO), deuterated dimethyl sulfoxide (DMSO-d₆), triethylamine, 4% paraformaldehyde, and Triton X-100 were purchased from Solarbio, China. Sodium xanthine, xanthine oxidase, urea, and glucose were obtained from Aladdin, China.

Histological staining kits, including Hematoxylin and Eosin, Safranin O/Fast Green, Toluidine Blue, Alizarin Red S, Oil Red O, and Alcian Blue staining kits, were purchased from Solarbio, China. The Cell Counting Kit-8 (CCK-8) and Live/Dead Cell Staining Kit were obtained from Dojindo, Japan. miR-140 lentivirus was supplied by Genechem, China. The Reactive Oxygen Species (ROS) Assay Kit, Mitochondrial Membrane Potential Assay Kit (JC-1), Annexin V-FITC/PI Apoptosis Detection Kit, and Catalase (CAT), superoxide dismutase (SOD), and malondialdehyde (MDA) assay kits were purchased from Beyotime, China. DAPI, rhodamine-conjugated phalloidin, DiI, and DiO fluorescent dyes were also obtained from Beyotime, China.

Flow cytometry antibodies against CD90, CD73, CD34, and CD45 were purchased from BioLegend, USA. Primary antibodies against ALIX, TSG101, CD81, Calnexin, COL2A1, MMP13, iNOS, Prdx5, Gpx4, Ndufa8, Cox4i1, Traf6, Ikbkg, GAPDH, and VDAC1, used for western blotting, immunofluorescence, and immunohistochemistry, were obtained from Abclonal, China. The RNA Extraction Kit and RNA Reverse Transcription Kit were purchased from Promega, USA. RIPA lysis buffer, protease and phosphatase inhibitor cocktails, BCA Protein Assay Kit, protein loading buffer, and Fast PAGE Gel Preparation Kits were obtained from Epizyme Biotech, China. Polyvinylidene fluoride (PVDF) membranes were purchased from Merck, Germany. Antigen retrieval solution, goat serum, and fluorescence- or horseradish peroxidase (HRP)-conjugated secondary antibodies were obtained from ZSGB-BIO, China. All chemicals and reagents were used as received without further purification.

### Isolation and identification of BMSCs

Three-week-old SD rats were euthanized and surface-sterilized. The femurs and tibiae were aseptically dissected from the lower limbs, and all adherent muscle, fascia, and soft tissues were meticulously removed. Both epiphyses were excised to isolate the diaphyseal segments, which were then transferred into perforated 5 mL EP tubes. Each tube was placed inside a 15 mL centrifuge tube and subjected to centrifugation to harvest the bone marrow. The collected marrow was washed once with α-MEM medium, centrifuged, and then resuspended in α-MEM complete medium supplemented with 10% FBS and 1% penicillin-streptomycin. Primary cultures were maintained at 37 °C in a humidified atmosphere of 5% CO₂. Upon reaching ~80% confluence, the cells were passaged for subsequent experiments.

To verify the stemness of BMSCs, surface antigen analysis and trilineage differentiation assays were performed. For surface antigen analysis, 1×10⁶ third-passage BMSCs were resuspended in phosphate-buffered saline (PBS) and incubated with monoclonal antibodies against CD90, CD73, CD34, and CD45 for 30 min at 4 °C in the dark. Following washing with PBS, the cells were resuspended in 300 μL PBS and analyzed using a flow cytometer (FACScan, Beckman, USA). For trilineage differentiation, BMSCs were induced toward osteogenic, adipogenic, and chondrogenic lineages following established protocols^50^. After induction, Alizarin Red S staining was applied to detect mineralized nodules, Oil Red O staining was used to visualize intracellular lipid accumulation, and Toluidine Blue staining was employed to assess sulfated proteoglycan deposition in the chondrogenic matrix.

### Isolation of chondrocytes and induction of the OA phenotype

One-week-old SD rats were euthanized, and the bilateral lower limbs were aseptically harvested. Following surface disinfection, the tissues were thoroughly rinsed with PBS. Muscular and soft tissues were carefully removed to isolate the articular cartilage from the knee joints. The cartilage specimens were subjected to sequential enzymatic digestion using 0.25% trypsin for 60 min followed by 0.2% type II collagenase for 8 h. The resulting cell suspension was filtered through a 75 μm mesh strainer and centrifuged at 200 g for 3 min to collect cells. After washing with PBS, cells were resuspended in DMEM/F12 (1:1) medium supplemented with 10% FBS and 1% penicillin-streptomycin for primary culture. Upon reaching approximately 80% confluence, cells were passaged for subsequent experiments.

To establish an in vitro OA chondrocyte model, cells were treated with 10 ng/mL IL-1β for 24 h. The induced OA phenotype was evaluated by assessing chondrocyte-associated markers (COL2A1, MMP13, and iNOS) using immunofluorescence staining and Western blotting. For immunofluorescence, cells were fixed, permeabilized, and blocked, followed by incubation with primary and secondary antibodies. Imaging was performed using the Opera Phenix Plus high-content imaging system (PerkinElmer, USA). For Western blotting, cellular proteins were lysed by ultrasonication and quantified using a BCA assay. Equal amounts of protein were separated by SDS-PAGE, transferred to PVDF membranes, blocked with 5% non-fat milk, and incubated overnight at 4 °C with specific primary antibodies. After incubation with secondary antibodies, bands were visualized and normalized to GAPDH using ImageJ software. Additionally, Alcian Blue and Safranin O staining were performed to further assess matrix-associated phenotype changes.

### Isolation and characterization of BMSCs and milk derived exosomes

BMSCs were cultured in vitro until reaching ~80% confluence, after which the medium was replaced with serum-free medium for 24 h to allow exosome secretion. The conditioned medium was collected, and exosomes were isolated via differential ultracentrifugation: 300 g for 10 min to remove cells, 2000 g for 20 min to remove cell debris, 10,000 g for 30 min to remove large vesicles, and 100,000 g for 70 min to collect exosomes. These BMSC-derived exosomes were defined as bExos. For milk-derived exosomes (mExos), fresh skim milk was pre-warmed to 37 °C and treated with 1% acetic acid to precipitate casein. The supernatant was filtered through a 0.22 μm membrane to remove protein aggregates, followed by ultracentrifugation at 174,900 g for 70 min to collect exosomes. The pellets of bExos and mExos were resuspended in PBS, aliquoted, and stored at −80 °C. Protein concentrations were determined using a BCA assay for subsequent experiments.

The isolated exosomes were characterized using TEM for morphology, NTA for size distribution and concentration, and Western blotting for exosomal markers (ALIX, TSG101, CD81) and negative marker Calnexin.

### Synthesis and characterization of DSPE-PEG_2000_-CAP

DSPE-PEG_2000_-MAL was accurately weighed and dissolved in dimethyl sulfoxide (DMSO) to form a homogeneous solution. An equimolar DMSO solution of the CAP peptide was then prepared and added dropwise to the DSPE-PEG_2000_-MAL solution under constant stirring to ensure homogeneous mixing and promote the conjugation reaction. To enhance the amidation efficiency, triethylamine was added as a basic catalyst, and the mixture was stirred at 25 °C for 24 h. The resulting mixture was dialyzed against deionized water using a 3.5 kDa molecular weight cutoff membrane for 48 h to remove unreacted peptides, followed by lyophilization to obtain the DSPE-PEG_2000_-CAP conjugate as a dry powder.

For structural validation, both the initial DSPE-PEG_2000_-MAL and the lyophilized DSPE-PEG_2000_-CAP were separately dissolved in deuterated DMSO (DMSO-d6) and sonicated for 10 min to achieve complete dissolution. Conjugation was confirmed by ¹H-NMR spectroscopy. Furthermore, samples were homogenized with potassium bromide (KBr), compressed into pellets, and subjected to FT-IR spectroscopy to acquire absorption spectra and identify characteristic functional group vibrations.

### Membrane fusion of bExos and mExos

To evaluate the membrane fusion behavior between exosomes derived from different sources, FRET analysis was employed. Briefly, bExos (5 μg/mL) and mExos (5 μg/mL) were fluorescently labeled with 0.5 mg/mL DiI and 1 mg/mL DiO, respectively, and incubated for 40 min in the dark at room temperature. Unbound dyes were removed by ultrafiltration (5,000 g, 5 min), followed by washing with PBS to ensure labeling purity. The labeled exosomes were then mixed at a 1:1 ratio and subjected to membrane fusion via three independent approaches: (1) incubation-assisted fusion, involving gentle shaking at 60 rpm at 37 °C for 12 h; (2) extrusion-assisted fusion, performed by 11 consecutive passes through a 400 nm polycarbonate membrane; and (3) a sequential incubation-extrusion group, in which samples were first incubated under the above conditions for 12 h followed by 11 cycles of extrusion.

Membrane fusion efficiency was quantified using FRET analysis by monitoring the emission of DiI under DiO excitation. Fluorescence intensities of the donor channel (501 nm) and acceptor channel (565 nm) were recorded using a fluorescence spectrophotometer. Membrane fusion efficiency was calculated using the ratio I_501_/ I_565_ and FRET efficiency [I_565_ / (I_501_ + I_565_)], where I_501_ and I_565_ represent fluorescence intensities at the corresponding wavelengths. An increase in FRET efficiency reflects reduced spatial distance between donor and acceptor fluorophores, indicating enhanced membrane fusion between exosomal populations. Meanwhile, the size distribution and PDI of the resulting fusion products were further characterized by NTA.

### Preparation and characterization of nanomotors

Initially, MnO₂ NPs were synthesized through a biomineralization process. Briefly, a 100 mM manganese chloride solution was combined with 10 mg/mL BSA, and the pH was adjusted to 10. The mixture was reacted under continuous stirring at 37 °C for 2 h. The product was then dialyzed to remove residual metal ions, resulting in purified MnO₂ NPs. Subsequently, 10% (w/w) DSPE-PEG_2000_-CAP was incorporated into 1 mg/mL mExos and subjected to 11 cycles of co-extrusion through a 400 nm polycarbonate membrane to achieve surface functionalization, yielding CAP-mExos. MnO₂ NPs were then loaded into CAP-mExos via sonication (100 W, 5 min, pulse mode: 1 s on/1 s off) to produce CAP-Mn@mExos. Finally, membrane fusion between CAP-Mn@mExos and bExos was performed as described above, generating the hybrid exosomal nanomotor CAP-Mn@hyExos.

The constructed exosomal formulations underwent systematic characterization. Initially, SDS-PAGE was used to assess protein profiles. Subsequently, NTA and DLS were employed to determine particle size distribution and zeta potential, respectively. The morphology and ultrastructure of the exosomes were examined by TEM and cryo-EM under complementary imaging conditions. The elemental composition and chemical states were analyzed by XPS. In addition, EDS elemental mapping was performed to evaluate elemental distribution within the hybrid exosomal systems. Building upon these analyses, manganese content in the nanomotors was quantified via ICP-OES, enabling calculation of MnO₂ encapsulation efficiency. To evaluate physical stability, samples were incubated in PBS supplemented with 10% FBS at 37 °C under gentle agitation (60 rpm) for 7 days. Aliquots were collected daily and analyzed by DLS to monitor changes in hydrodynamic diameter and PDI. To further assess stability under OA-relevant microenvironmental conditions, exosomal nanomotors were labeled with DiI and dispersed in PBS containing 10% FBS, H₂O₂ at different concentrations (100 μM, 1 mM, and 10 mM), and acidic conditions (pH 6.8) supplemented with 100 μM H₂O₂. Samples were incubated at 37 °C in the dark under gentle agitation. At predetermined time points (0–7 days), suspensions were subjected to ultrafiltration (100 kDa, 6500 g, 15 min) to separate free dye from encapsulated dye. The filtrates were collected for fluorescence measurement. Total fluorescence (Ftotal) was obtained after complete disruption of vesicles using 1% Triton X-100. The cumulative release was calculated as Release (%) = (Fₜ − F₀) / (F_total_ − F₀) × 100%, where Fₜ is the fluorescence at time t and F₀ is the initial background from the control group. In parallel, DLS measurements were performed to dynamically track changes in particle size and PDI under different conditions, enabling a comprehensive assessment of colloidal stability and structural integrity. Collectively, this multi-faceted analytical approach provided a comprehensive evaluation of the physicochemical properties and successful fabrication of the exosomal nanomotors.

### Evaluation of ROS-responsive motility and cartilage matrix penetration

To evaluate the ROS-responsive motility of the exosomal nanomotors, bExos, mExos, CAP-mExos, CAP-Mn@mExos, and CAP-Mn@hyExos were incubated in the presence or absence of 100 μM H₂O₂. Their motion trajectories in solution were recorded in real time using nanoparticle tracking analysis (NTA). ROS-responsive motion under oxidative stress-like conditions was quantitatively assessed based on displacement and average velocity. To further investigate directional migration, a Y-shaped microfluidic chip was constructed to mimic an H₂O₂-enriched environment. In this system, the central channel was used for injection of nanoplatforms, while the two side branches were filled with Matrigel containing either H₂O₂ or no H₂O₂. Different formulations were introduced into the central channel and allowed to migrate toward the terminal chambers. Fluorescence imaging was performed at 1 h for the terminal chambers. In addition, to evaluate ROS-selective responses, the same Y-shaped microfluidic platform was employed. The central channel was used to inject nanomotors, while the two branches were loaded with Matrigel containing different stimuli, including PBS, urea, glucose, and ROS-related components (H₂O₂ and O₂⁻, generated by the xanthine/xanthine oxidase system). Nanomotors migrated toward the terminal chambers, and fluorescence imaging was performed at 1 h to assess migration behavior under different microenvironmental conditions.

To evaluate cartilage matrix penetration ability, an ex vivo cartilage model was established using femoral head cartilage from SD rats. The tissues were pretreated in H₂O₂-containing medium to mimic an oxidative microenvironment, followed by incubation with DiI-labeled bExos, mExos, CAP-mExos, CAP-Mn@mExos, Mn@hyExos, and CAP-Mn@hyExos. Samples were collected at 2, 4, and 6 days, washed with PBS, and processed for cryo-embedding and sectioning. Nuclei were counterstained with DAPI, and the spatial distribution of exosomes within cartilage tissue was visualized using CLSM. ImageJ was used to quantify fluorescence penetration depth along the tissue axis. The inclusion of Mn@hyExos lacking CAP modification enabled comparison of the respective contributions of ROS-responsive motion and CAP-mediated matrix binding. To further characterize the spatial relationship between exosomes and chondrocytes, Z-stack confocal images were acquired from cartilage sections at multiple optical planes. Three-dimensional reconstruction together with orthogonal view analysis was performed using the microscope software to visualize the spatial distribution of DiI-labeled exosomes relative to DAPI-positive nuclei.

On this basis, in vivo validation was performed in an established OA rat model. ROS levels and spatial distribution in knee cartilage were first examined in healthy and OA rats. Knee joint condyles were harvested, washed with PBS, and processed for cryo-sectioning. ROS staining was performed using a commercial ROS detection kit, followed by DAPI nuclear counterstaining. CLSM was used to visualize ROS distribution in cartilage tissue. Subsequently, DiI-labeled exosomal formulations were intra-articularly injected into OA knees. After 2 days, knee condyles were collected and processed using the same procedures (PBS washing, cryo-embedding, sectioning, and DAPI staining). CLSM was used to observe the spatial distribution of exosomes within cartilage. Based on fluorescence distribution along cartilage depth, the in vivo penetration capability of the nanomotors in OA joints was quantitatively evaluated.

### Evaluation of chondrocyte targeting

Chondrocytes were seeded onto pre-positioned coverslips in 12-well plates at a density of 1 × 10⁵ cells per well and allowed to adhere for 12 h. An OA cellular model was then established by stimulation with 10 ng/mL IL-1β for 24 h. Then, the cells were incubated with various DiI-labeled exosomal formulations for an additional 12 h. Following incubation, the culture medium was aspirated, and cells were gently rinsed three times with PBS. Subsequently, the cells were fixed, permeabilized, and stained with FITC-conjugated phalloidin in the dark for 40 min to specifically visualize F-actin cytoskeletal structures. Following staining, the cells were washed with PBS and counterstained with DAPI for 10 min to visualize nuclei. Coverslips were then mounted, and the intracellular distribution of exosome-derived signal was examined via CLSM to evaluate cellular targeting. In parallel, another set of identically treated cells was collected and subjected to flow cytometry for quantitative analysis of DiI fluorescence intensity, providing further assessment of cellular uptake efficiency and targeting behavior of the various exosomal formulations by chondrocytes.

### Evaluation of in vivo joint retention, biodistribution, and clearance

To systematically assess the intra-articular retention of exosomal nanomotors, an SD rat OA model was established via intra-articular injection of monosodium iodoacetate (MIA). Upon successful model validation, 100 μL of DiI-labeled exosomal formulations were administered into the OA knee joint cavity. Fluorescence imaging of the knee region was conducted at 0, 1, 2, 3, 5, 7, 10, and 14 days post-injection using an IVIS system. Fluorescence intensities were quantitatively analyzed with the integrated IVIS software, and decay profiles were generated to characterize the retention kinetics and intra-articular residence of each formulation. To further evaluate the influence of the OA joint environment on retention behavior, age-matched healthy SD rats were included as a normal control group and subjected to the same procedures.

For biodistribution and clearance analysis, animals were sacrificed at 3 days, 2 weeks, and 8 weeks post-administration. Major organs, including the heart, liver, spleen, lung, kidney, and brain, along with knee joint tissues, were harvested for analysis. Whole-organ fluorescence imaging was first performed using IVIS to provide a qualitative overview of in vivo distribution and tissue accumulation. Subsequently, selected tissues (liver, spleen, kidney, brain, and joint) were digested and analyzed by ICP-OES to quantify Mn content, thereby enabling evaluation of systemic distribution and elimination pathways. Considering the brain as a potential organ of concern for Mn-associated accumulation, brain tissues collected at 8 weeks were subjected to Hematoxylin & Eosin staining and examined under light microscopy for structural alterations, inflammatory infiltration, or histopathological changes. Collectively, these analyses provide a comprehensive evaluation of the in vivo retention, biodistribution, metabolic fate, and biosafety of the exosomal nanomotors.

### Isolation and characterization of miR-140-overexpressing bExos

BMSCs were transfected with Lentivirus-miR-140 in accordance with the manufacturer’s instructions, with optimal transfection conditions identified via fluorescence inverted microscopy (Nikon, Japan). Successfully transfected BMSCs were expanded in vitro, and exosomes overexpressing miR-140 (140@bExos) were subsequently isolated from the culture medium. The overexpression efficiency of *miR-140* in BMSCs was evaluated by qPCR to verify the transfection outcome. To further evaluate the *miR-140* content in exosome-treated chondrocytes, chondrocytes were co-cultured with 140@bExos for 24 h, followed by evaluation of intracellular *miR-140* expression using qPCR. All qPCR procedures were rigorously performed according to the manufacturer’s protocols. Specifically, total RNA was extracted, reverse-transcribed into complementary DNA (cDNA), and amplified on a LightCycler® 96 Real-Time PCR System (Roche, Switzerland). Relative gene expression was then calculated using the 2 ^− ΔΔCq^ method. The sequences of primers used are listed in Supplementary Table 3.

### In vitro biocompatibility evaluation

To systematically assess the biocompatibility of various exosomal formulations in vitro, a series of experiments including live/dead staining, hemolysis assay, cytoskeletal staining, cell proliferation, and scratch healing assays were conducted. Chondrocytes were seeded into culture plates, stimulated with IL-1β, and subsequently co-cultured with different exosome preparations. Live/dead staining was performed in strict accordance with the instructions of the Live/Dead Cell Staining Kit, and results were observed and recorded under a fluorescence inverted microscope. For the hemolysis assay, whole blood was initially collected using heparin sodium anticoagulant tubes. After centrifugation and washing with PBS, red blood cells (RBCs) were isolated and diluted to a 4% RBC suspension. This suspension was mixed 1:1 (v/v) with each exosomal formulation and incubated at 37 °C with gentle shaking (70 rpm) for 2 h. The supernatant absorbance was measured at 545 nm using a microplate reader. PBS and 0.2% Triton X-100 served as negative and positive controls, respectively, with the latter defined as 100% hemolysis. The hemolysis rate was calculated as follows: Hemolysis (%) = (A_sample_ - A_negative_) / (A_positive_ - A_negative_) × 100%. For cytoskeletal staining, cells were fixed, permeabilized, and stained with phalloidin and DAPI to visualize F-actin and nuclei, respectively. Images were acquired using an Opera Phenix Plus high-content imaging system. Cell proliferation was evaluated using a CCK-8 kit. In the scratch healing assay, when chondrocytes in 6-well plates reached over 90% confluence, the medium was replaced with medium containing 2% FBS for overnight starvation. Subsequently, a standardized scratch was introduced using a 1 mL pipette tip, and cells were cultured for another 36 h in conditioned medium supplemented with 2% FBS and the respective exosomal formulations. Scratch images were captured at 0, 12, 24, and 36 h and analyzed using ImageJ 1.8.0. The migration rate was calculated as follows: Migration rate (%) = (S₀ – Sₜ) / S₀ × 100%, where S₀ represents the initial scratch area and Sₜ denotes the scratch area at each time point.

### Evaluation of cellular antioxidant capacity

To establish an in vitro oxidative stress model, chondrocytes were first exposed to different concentrations of H₂O₂ for 24 h, and cell viability was evaluated using a CCK-8 assay to determine an appropriate working concentration based on a dose–response viability curve (IC₅₀-guided selection). Chondrocytes were subsequently treated with the optimal working concentration of H₂O₂ for 24 h and co-cultured with different exosomal formulations. Their antioxidant capacity was comprehensively evaluated through intracellular ROS detection, JC-1 staining, apoptosis assay, live/dead staining, and measurements of oxidative stress-related biomarkers. Intracellular ROS levels were quantified using a Reactive Oxygen Species Assay Kit following the manufacturer’s instructions. Fluorescence signals were observed and imaged using a fluorescence inverted microscope. For JC-1 staining, chondrocytes seeded in 96-well plates were incubated with JC-1 working solution at 37 °C for 20 min, followed by washing with JC-1 staining buffer. Fluorescence of JC-1 aggregates (red) and monomers (green) was visualized using an Opera Phenix Plus high-content imaging system. Apoptosis was analyzed using an Annexin V-FITC/PI Apoptosis Detection Kit according to the manufacturer’s protocol and quantified by flow cytometry. Cell viability was assessed using a Live/Dead Cell Staining Kit. In addition, CAT activity, SOD activity, and MDA levels were measured using commercial assay kits according to the manufacturers’ instructions to comprehensively evaluate cellular oxidative stress status.

### Evaluation of OA chondrocyte repair

An in vitro model of OA was established by treating chondrocytes with 10 ng/mL IL-1β for 24 h. The cells were subsequently co-cultured with various exosomal formulations. The therapeutic effects of each exosomal preparation were evaluated by quantifying expression levels of the anabolic marker COL2A1, the catabolic marker MMP13, and the inflammatory mediator iNOS using qPCR, immunofluorescence, and Western blotting. Primer sequences used in qPCR are provided in Supplementary Table 3. Additionally, Safranin O and Alcian Blue staining were employed to assess glycosaminoglycan deposition, providing further evaluation of extracellular matrix content in chondrocytes.

### Rat knee OA modeling and therapeutic intervention

Eight-week-old male SD rats (weight: 200 ± 20 g) were employed in this study. Only male rats were included to minimize variability associated with sex differences. A knee OA model was established using the “ACLT + DMM” surgical approach. Briefly, under isoflurane inhalation anesthesia, the surgical site was shaved and disinfected. The right knee joint was exposed through a medial parapatellar approach, the patella was laterally dislocated, the anterior cruciate ligament was transected, and the anterior horn of the medial meniscus was resected. The incision was closed layer by layer, and the rats were allowed unrestricted cage activity with free access to food and water. In the sham group, the rats underwent capsulotomy only without ACLT or DMM.

Four weeks post-surgery, except for the Sham group, OA-induced rats were randomly divided into four groups: PBS (Control), CAP-Mn@hyExos, 140@bExos, and CAP-Mn/140@hyExos. Each group received the corresponding formulation via intra-articular injection (100 μL per injection, corresponding to 1 μg μL⁻¹ exosomal protein, i.e., 100 μg per joint) once weekly for four consecutive weeks.

### Gait analysis and micro-CT scanning

At 4 and 8 weeks post-intervention, gait parameters were assessed using an automated animal gait analysis system (VisuGait, China) during unassisted walking. Footprint data were quantitatively analyzed with dedicated software to evaluate gait pattern, stride length, stride frequency, walking velocity and plantar pressure distribution.

Upon completion of gait analysis, animals were euthanized, and knee joint specimens were collected and fixed for ex vivo micro‑CT scanning (NMC‑100, Pinsheng, China). Three-dimensional reconstructions of the joint architecture were generated to assess osteophyte formation and loose bodies. The tibial plateau region was isolated for detailed three-dimensional histomorphometric analysis of subchondral bone microstructure, including BMD, Tb.N, Tb.Th, and Tb.Sp.

### Histological and immunohistochemical evaluation

After micro-CT imaging, peri-articular soft tissues were meticulously dissected to isolate femoral condylar cartilage specimens, which were photographed for gross observation. The samples were decalcified, embedded in paraffin, and serially sectioned along the sagittal plane. Tissue sections were stained with Hematoxylin & Eosin, Safranin O & Fast Green, and Toluidine Blue to evaluate cartilage architecture, proteoglycan content, and cellular distribution. Cartilage integrity was systematically graded and staged using the OARSI scoring system, with quantitative scoring used to assess tissue health status. Immunohistochemical staining was carried out to examine the specific expression patterns of COL2A1, MMP13, and iNOS in cartilage tissues. Semi-quantitative analysis of staining intensity was performed using ImageJ software. Furthermore, systemic biocompatibility of the exosomal nanomotors was evaluated through serum biochemical analysis and histological examination of major organs via Hematoxylin & Eosin staining.

### Transcriptomic sequencing and analysis

To systematically elucidate the transcriptomic regulatory effects of exosomal nanomotors in OA cartilage, knee joint cartilage tissues were harvested from CAP-Mn/140@hyExos-treated and OA control rats at 8 weeks post-intervention. Samples were rapidly dissected under RNase-free conditions and snap-frozen in liquid nitrogen. Total RNA was extracted using the TRIzol method, and only high-quality RNA (A260/A280 = 1.8-2.2) confirmed by integrity and purity assessment was used for downstream analysis. mRNA libraries were constructed using the Hieff NGS® Ultima kit and sequenced on an Illumina NovaSeq 6000 platform to generate 150 bp paired-end reads. Raw reads were processed with fastp to obtain clean reads, which were then aligned to the reference genome using HISAT2. Gene expression levels were quantified using FPKM and HTSeq-count.

Data quality and inter-sample consistency were assessed by PCA, Pearson correlation analysis, and hierarchical clustering. DEGs were identified using DESeq2 (p < 0.05, |log₂FC | > 1) and visualized by volcano plots. GO and KEGG enrichment analyses were performed using clusterProfiler to characterize the functional profiles of DEGs, particularly in oxidative stress, mitochondrial metabolism, and inflammatory response pathways. In parallel, PPI network analysis was conducted to identify functional modules and interaction patterns among key genes, thereby revealing potential molecular regulatory networks associated with exosomal nanomotor-mediated cartilage repair. Key pathway-associated genes were further validated at multiple levels. Protein expression and spatial distribution were first assessed in tissue sections by immunohistochemistry, while at the cellular level, Western blotting and qPCR were performed to verify corresponding protein and transcript-level changes, further supporting the transcriptomic findings across tissue and cellular contexts. Primer sequences used in qPCR are provided in Supplementary Table 3.

### Statistical analysis

All experiments were performed with at least three independent biological replicates to ensure reproducibility. Data are presented as mean ± standard deviation (mean ± SD). Statistical comparisons between two groups were conducted using a two-tailed unpaired or paired Student’s t-test, whereas multiple-group comparisons were analyzed using one-way analysis of variance (ANOVA) followed by Tukey’s post hoc test. For non-continuous data such as OARSI scores that did not follow a normal distribution, non-parametric analysis was performed using a Kruskal-Wallis test followed by two-tailed Mann-Whitney U test. A *P* value < 0.05 was considered statistically significant. All data acquisition and statistical analyses were performed in a blinded manner, and no data were excluded from the analyses. All statistical analyses were carried out using GraphPad Prism software (version 8.0.2).

### Reporting summary

Further information on research design is available in the Nature Portfolio Reporting Summary linked to this article.

## Supplementary information

Supplementary Information

Reporting Summary

Transparent Peer Review file

## Source data

Source Data 1

Source Data 2

## Data Availability

The transcriptome sequencing data generated in this study have been deposited in the Genome Sequence Archive (GSA) of the National Genomics Data Center (NGDC), China National Center for Bioinformation, under accession code CRA035408 (https://ngdc.cncb.ac.cn/gsa/browse/CRA035408). The data supporting the findings of this study are available within the Article, Supplementary Information, or Source Data file. The Source Data file contains the data underlying Figs. 2D, E, K–L; 3C, E, G, I, K; 4C–E, H, J; 5C, E, G, I, K; 6A, D, F, H; 7G–I; 8D–F, J–L; 9I; and Supplementary Figs. 11, 15, 16, 24, 26, 28–32, and 36. Source data are provided with this paper.
