# Supplementary material for: Hybrid exosomal nanomotors convert pathological ROS into transport-driving cues to enhance cartilage accumulation in osteoarthritis
Source: Nat Commun. 2026 Aug 29;17:10310. doi: 10.1038/s41467-026-76949-8 (PMC13619634; doi:10.1038/s41467-026-76949-8)

Supplementary Information

Hybrid exosomal nanomotors convert pathological ROS into transport-driving cues to enhance cartilage accumulation in osteoarthritis

Kai Huang†, Hui-Zhi Liu†, Yuan Liu†, Jia-Yan Lyu Qing-Yi Zhang, Zi-Yuan Feng, Zi-Lin Wang, Hui Dong, Rong Nie, Yi-Wen Zhang, Yue-Qi Zhang, Yi Zeng, Hui-Qi Xie*, Zhou Zhou*, Bin Shen*

Supplementary Table 1 | Particle size, PDI, and Zeta potential of mExos after modification with DSPE-PEG_{2000}-CAP via incubation or extrusion.

Grouping	Partical Size (nm)	PDI	Zeta potential (mV, 0.1×PBS)
mExos	133.57 ± 1.47	0.16 ± 0.02	-11.17 ± 0.45
DSPE-PEG_{2000}-CAP + mExos (Incubation)	123.97 ± 1.56	0.13 ± 0.01	-11.87 ± 0.74
DSPE-PEG_{2000}-CAP + mExos (Extrusion)	132.50 ± 1.48	0.15 ± 0.01	-9.84 ± 0.80

Supplementary Table 2 | Particle size, PDI, and Zeta potential of mExos after ultrasonic loading of MnO_2 NPs at different feed ratios.

Grouping (Particle ratio)	Partical Size (nm)	PDI	Zeta potential (mV, 0.1×PBS)
mExos	118.47 ± 0.54	0.15 ± 0.02	-17.57 ± 1.31
MnO_2 NPs	72.59 ± 0.66	0.25 ± 0.01	-17.78 ± 0.90
mExos: MnO_2 NPs = 1: 4	80.16 ± 1.70	0.31 ± 0.03	——
mExos: MnO_2 NPs = 1: 2	92.36 ± 1.28	0.28 ± 0.03	——
mExos: MnO_2 NPs = 1: 1	106.50 ± 2.58	0.24 ± 0.02	——
mExos: MnO_2 NPs = 2: 1	113.00 ± 2.55	0.28 ± 0.03	——

Supplementary Table 3 | Primer sequences of *miR-140*, *COL2A1*, *MMP13*, *iNOS*, *GAPDH, Prdx5, Gpx4, Ndufa8, Cox4i1, Traf6,* and *Ikbkg* used for qPCR analysis in this study.

Genes	Primer sequences
miR-140 (Stem-loop RT primer)	5'- GTCGTATCCAGTGCAGGGTCCGAGGT ATTCGCACTGGATACGACCTACCA -3'
U6	F: 5'- CTCGCTTCGGCAGCACA -3'<br>R: 5'- AACGCTTCACGAATTTGCGT -3'
COL2a1	F: 5'- GACGCCACGCTCAAGTC -3'<br>R: 5'- TCTCCGCTCTTCCACTCTG -3'
MMP13	F: 5'- CCATCCCGAGACCTCAT -3'<br>R: 5'- GCCTTTTCACCTCTTTTGG -3'
iNOS	F: 5'- TCCCAGCCCAACAACAC -3'<br>R: 5'- TCTCCATTGCCCCAGTTT -3'
GAPDH	F: 5'- CAAGAAGGTGGTGAAGCAG -3'<br>R: 5'- CAAAGGTGGAAGAATGGG -3'
Prdx5	F: 5'- CAGGCAGAAGGCAAGGTT -3'<br>R: 5'- CCGACGATTCCCAAAGAG -3'
Gpx4	F: 5'- TGAAAGTCCAGCCCAAGG -3'<br>R: 5'- CCACGCAGCCGTTCTTA -3'
Ndufa8	F: 5'- TTCCGGCAGATCAAGAG -3'<br>R: 5'- TCCAACACACATTCGTCAA -3'
Cox4i1	F: 5'- CGTGACTACCCCTTGCCT -3'<br>R: 5'- CCCACCACTGTCTTCCACT -3'
Traf6	F: 5'- GAGTGCCCCATCTGCTT -3'<br>R: 5'- CTTGTGACCTGCATCCCT -3'
Ikbkg	F: 5'- TTGAGGATATGAGGAAGCG -3'<br>R: 5'- GCCAAATGAAAGGAGTGG -3'

Supplementary Fig. 1 | Morphological characteristics of the primary and passaged BMSCs, scale bar = 200 μm. Data are representative of three independent experiments with similar results.

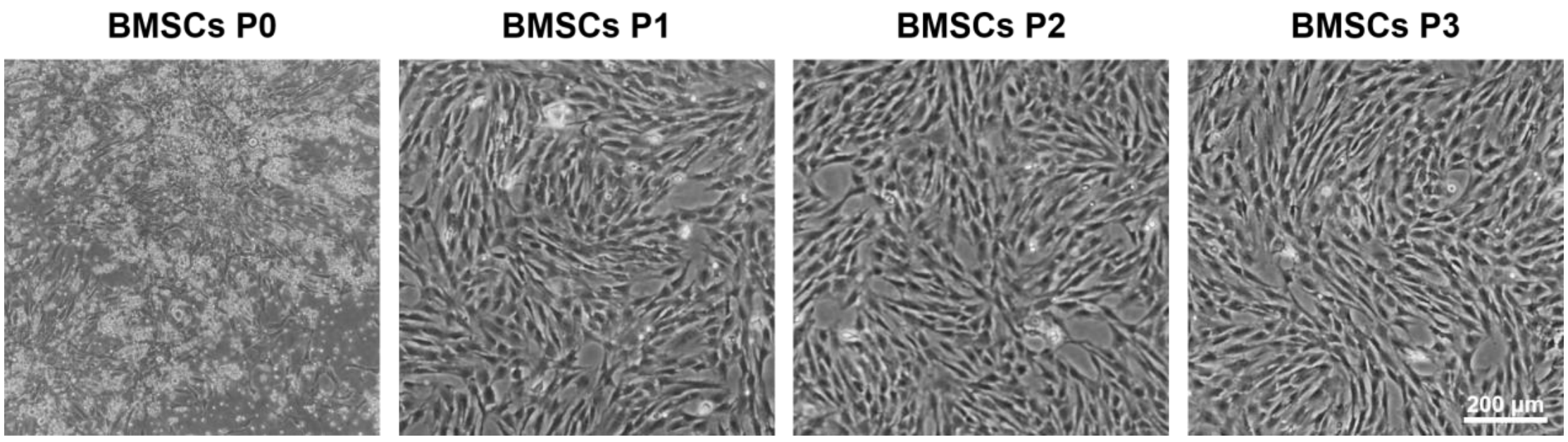

Supplementary Fig. 2 | Flow cytometric analysis of BMSC surface markers, showing high expression of CD90 and CD73, and negative for CD34 and CD45. Data are representative of three independent experiments with similar results.

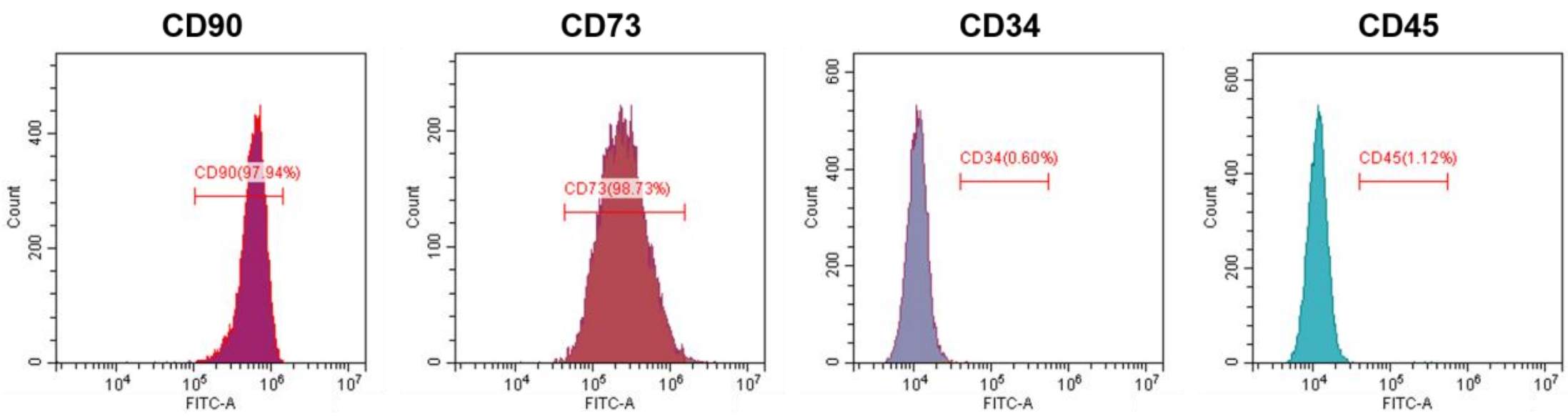

Supplementary Fig. 3 | Trilineage differentiation of BMSCs, showing osteogenic mineralized nodules (scale bar = 200 μm), adipogenic lipid droplets (scale bar = 100 μm), and chondrogenic lacunae (scale bar = 200 μm). Data are representative of three independent experiments with similar results.

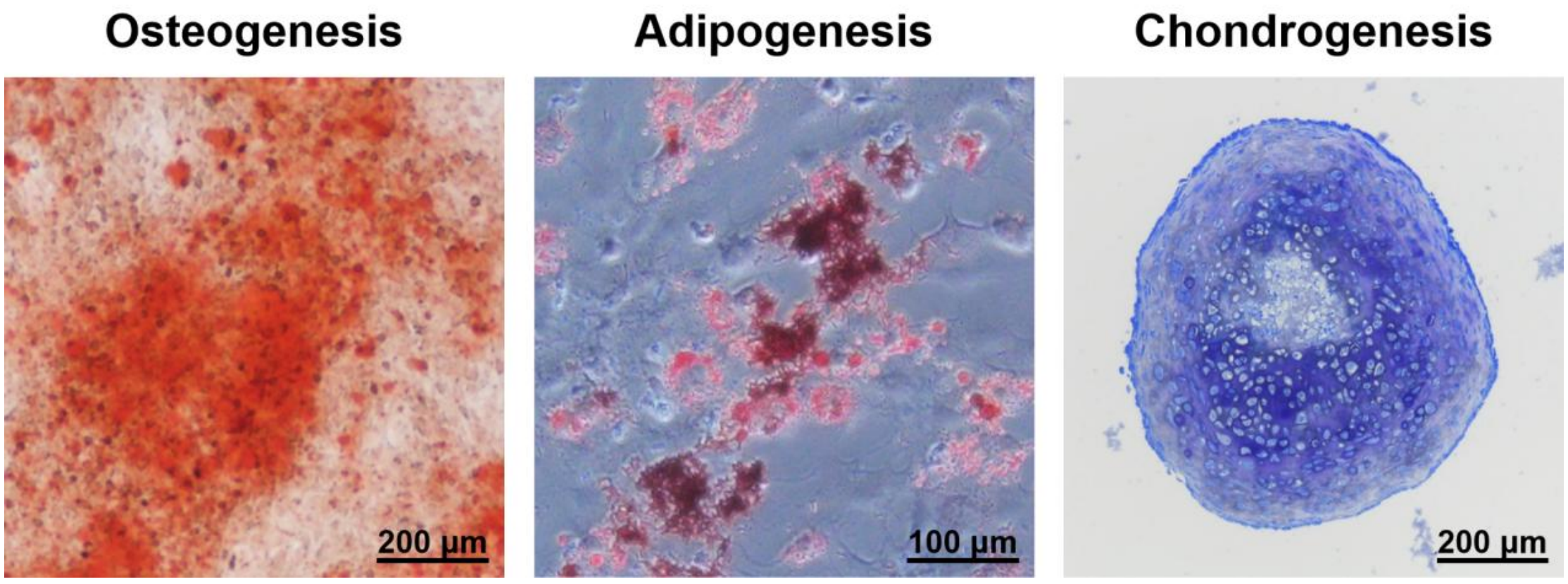

Supplementary Fig. 4 | Morphological characteristics of the primary and passaged chondrocytes, scale bar = 200 μm. Data are representative of three independent experiments with similar results.

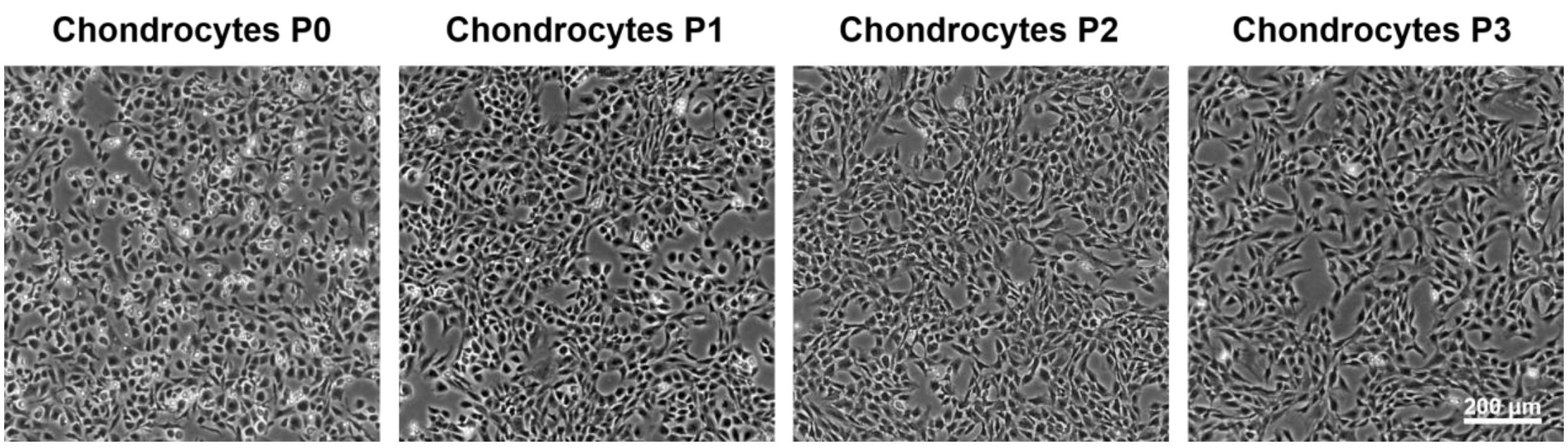

Supplementary Fig. 5 | Immunofluorescence analysis of COL2A1, MMP13, and iNOS expression in chondrocytes treated with or without 10 ng/mL IL-1β, scale bar = 50 μm. Data are representative of three independent experiments with similar results.

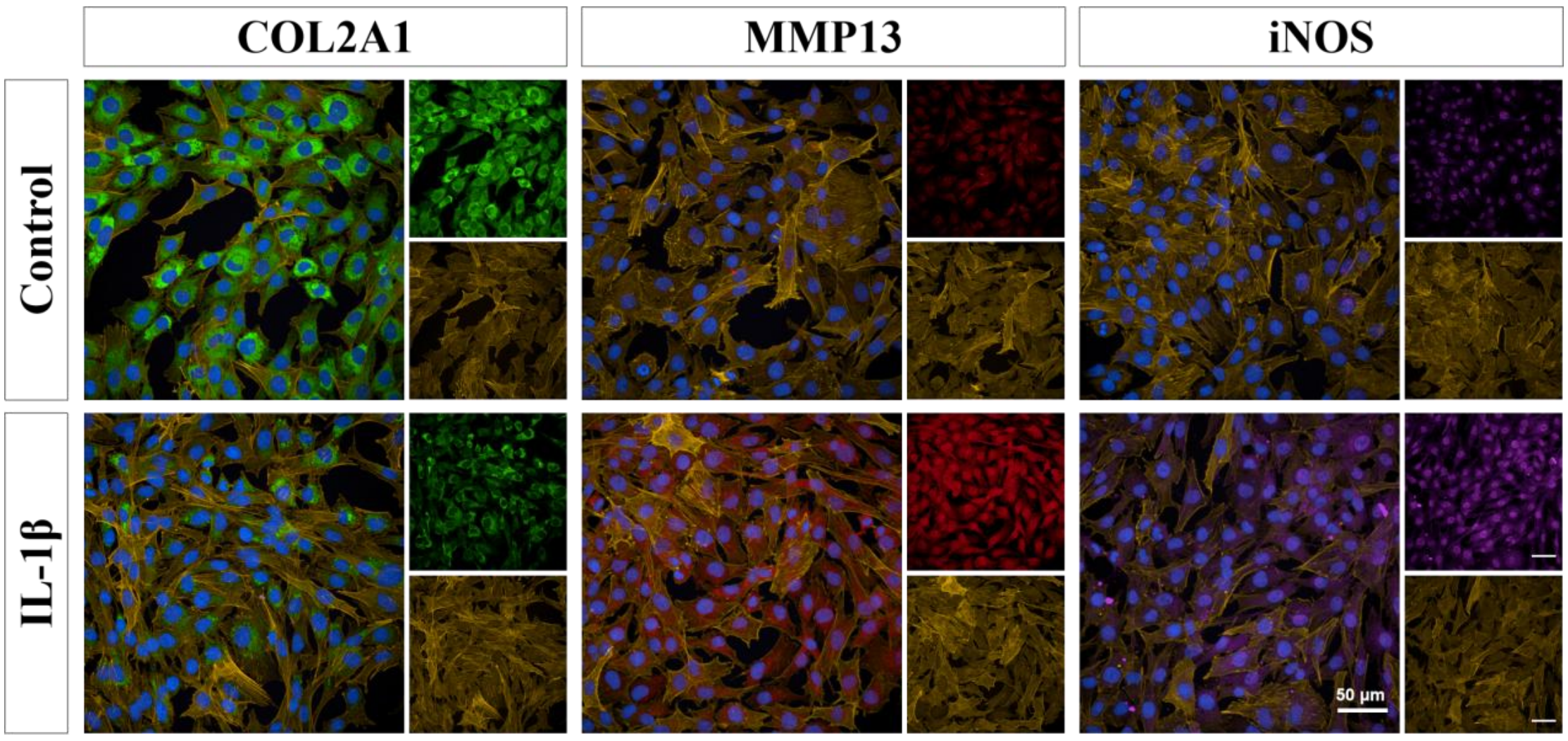

Supplementary Fig. 6 | Western blot analysis of COL2A1, MMP13, and iNOS expression in chondrocytes treated with or without 10 ng/mL IL-1β. Data are representative of three independent experiments with similar results.

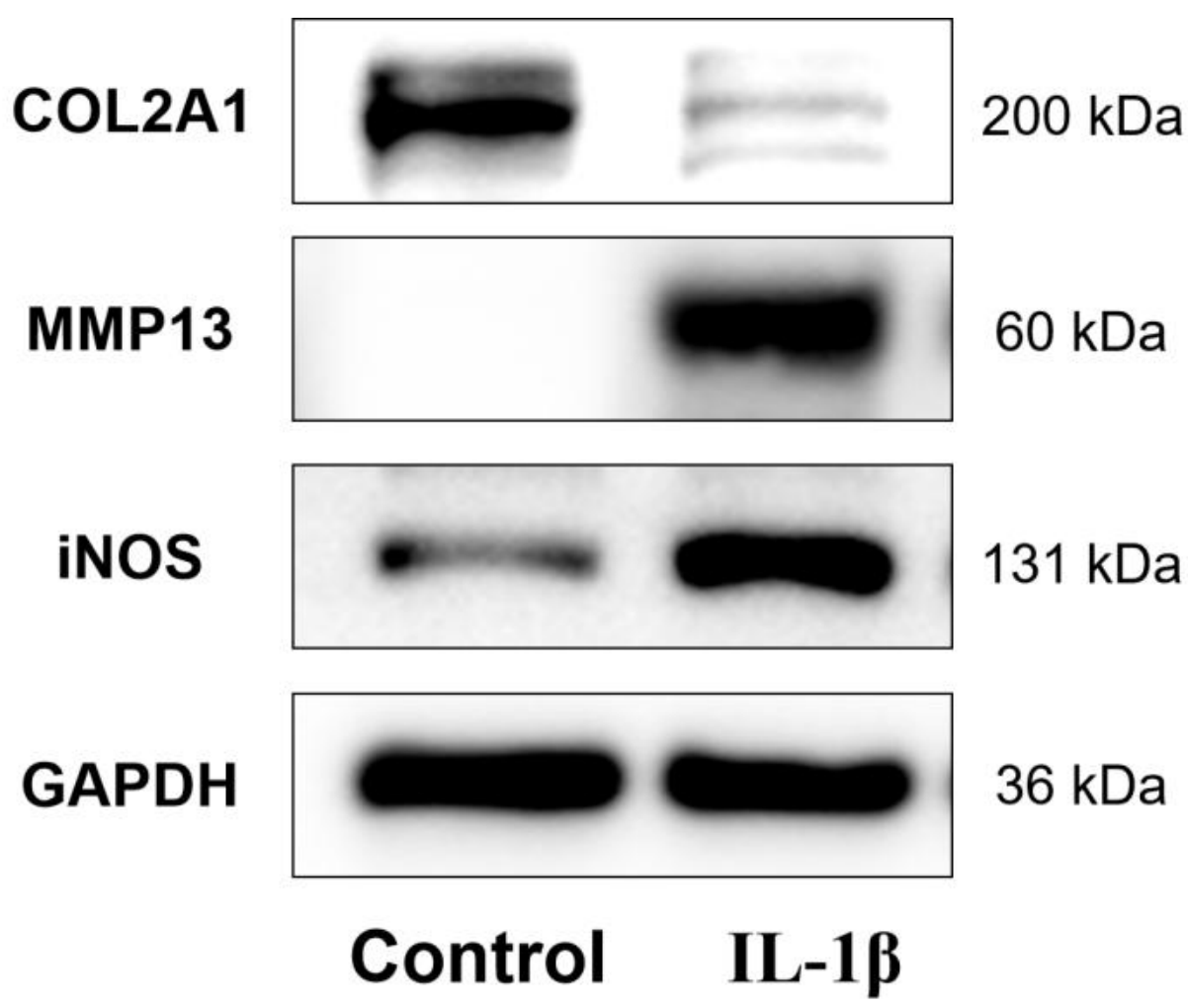

Supplementary Fig. 7 | Safranin O and Alcian Blue staining of chondrocytes treated with or without 10 ng/mL IL-1β, scale bars = 200 μm. Data are representative of three independent experiments with similar results.

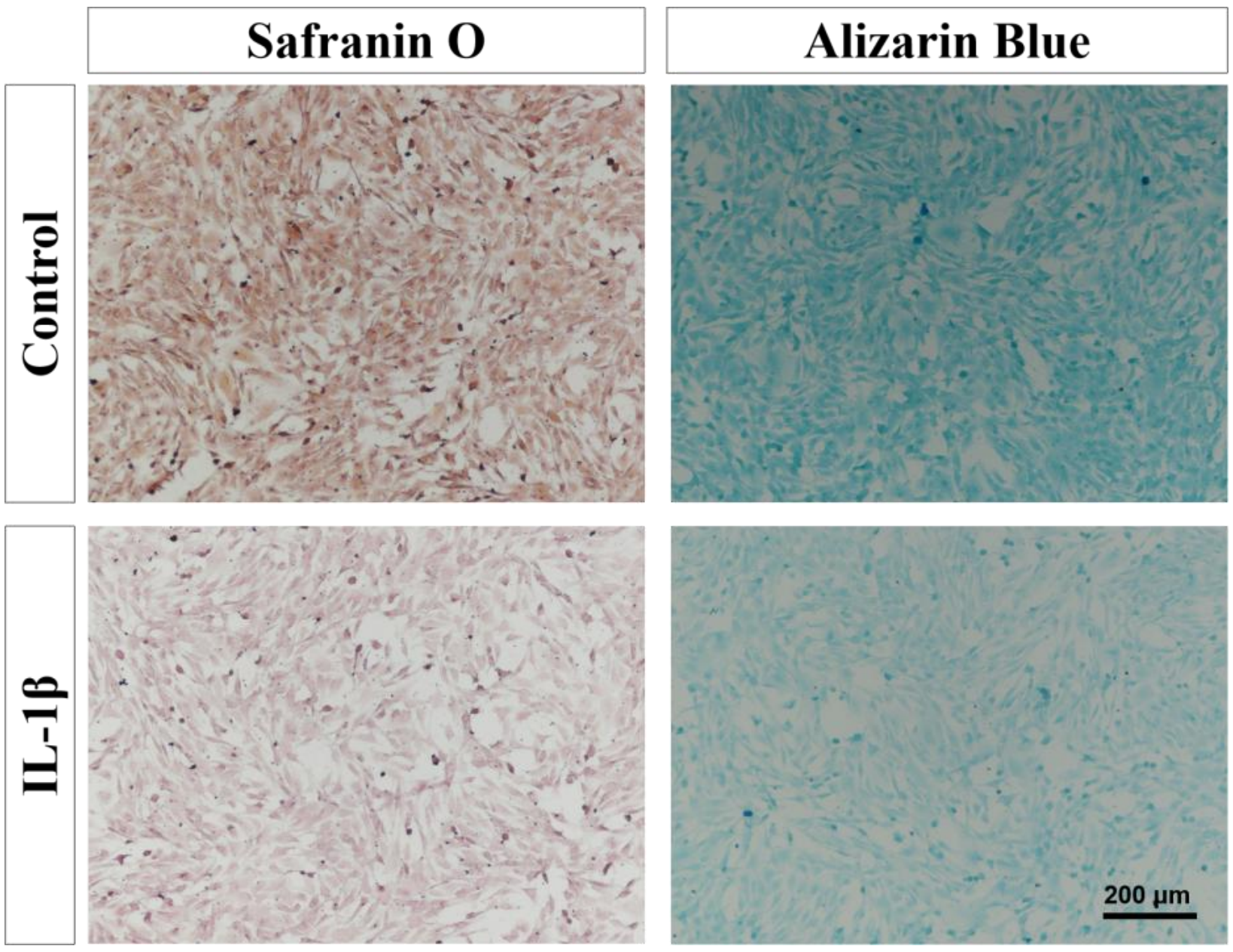

Supplementary Fig. 8 | Schematic illustration of the synthesis of DSPE-PEG_{2000}-CAP. CAP was covalently conjugated to the terminal maleimide group of DSPE-PEG_{2000}-MAL via a thiol-maleimide click reaction to yield the targeted lipid-polymer conjugate.

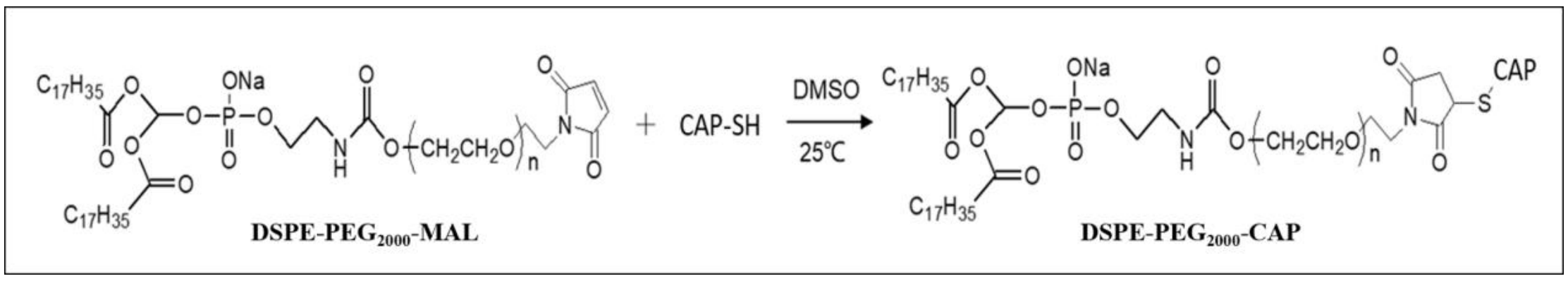

Supplementary Fig. 9 | ^{1}H-NMR characterization of DSPE-PEG2000-CAP. The spectrum shows characteristic proton signals of CAP alongside the preserved DSPE-PEG2000-MAL backbone, confirming successful conjugation. Data are representative of three independent experiments with similar results.

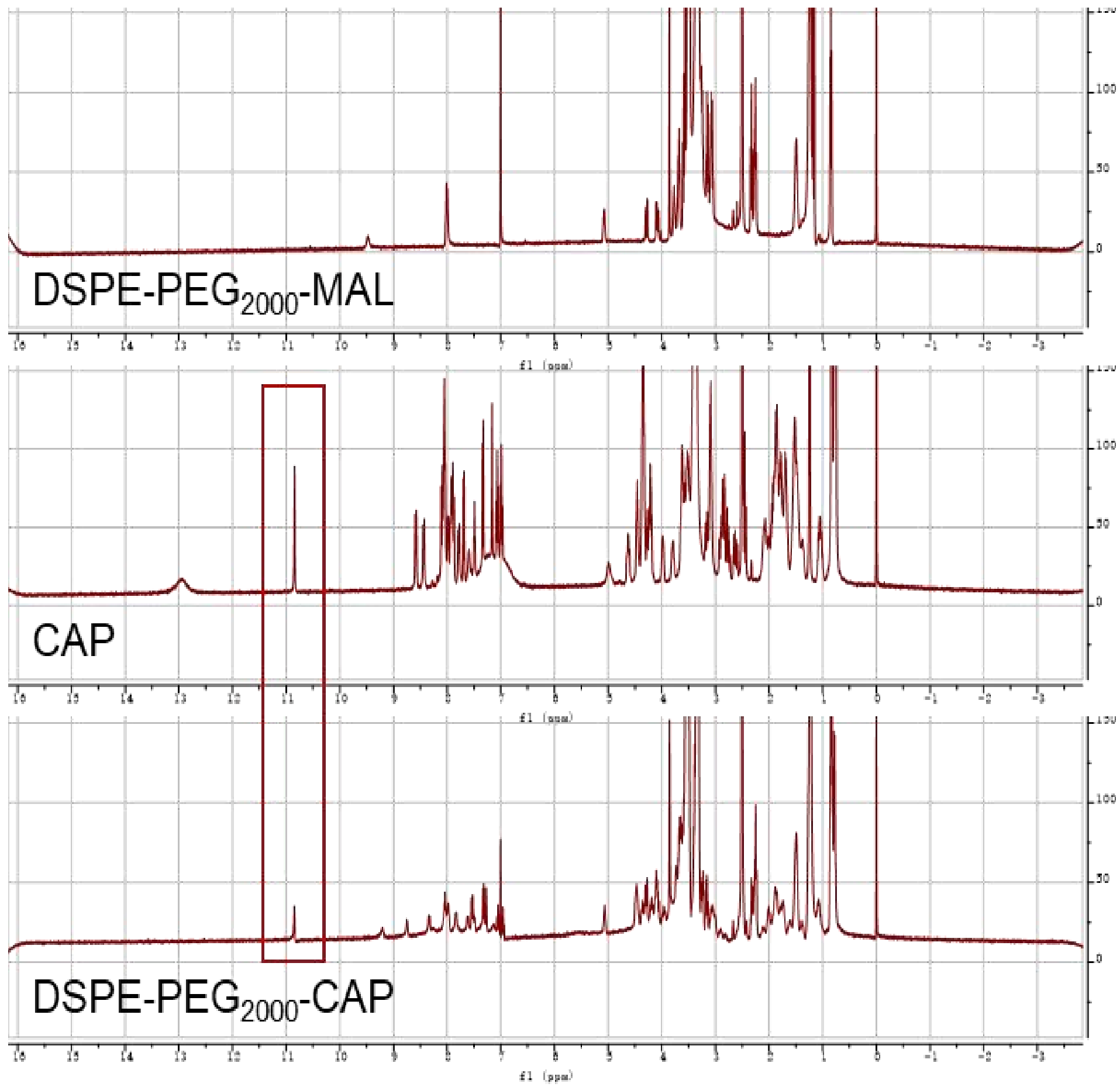

Supplementary Fig. 10 | FT-IR analysis of DSPE-PEG$_{2000}$-CAP. The spectrum shows characteristic absorption bands of CAP along with the preserved vibrational signatures of the DSPE-PEG$_{2000}$-MAL backbone, confirming successful conjugation. Data are representative of three independent experiments with similar results.

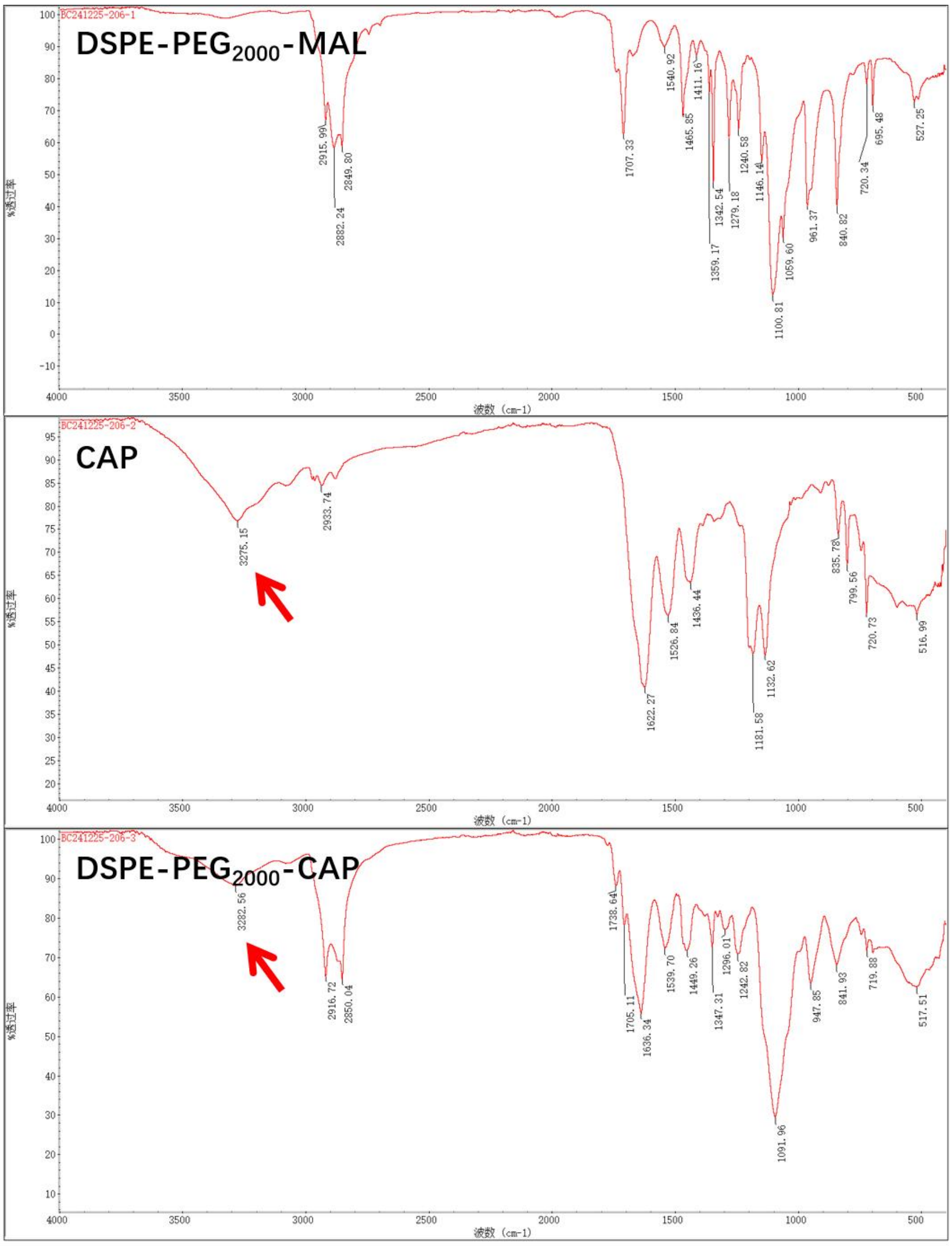

Supplementary Fig. 11 | Analysis of particle size and PDI for membrane fusion between exosomes using incubation, extrusion, and combined “incubation-extrusion” methods (n = 3, biological replicates per group). Data are presented as the mean ± SD. Source data are provided as a Source Data file.

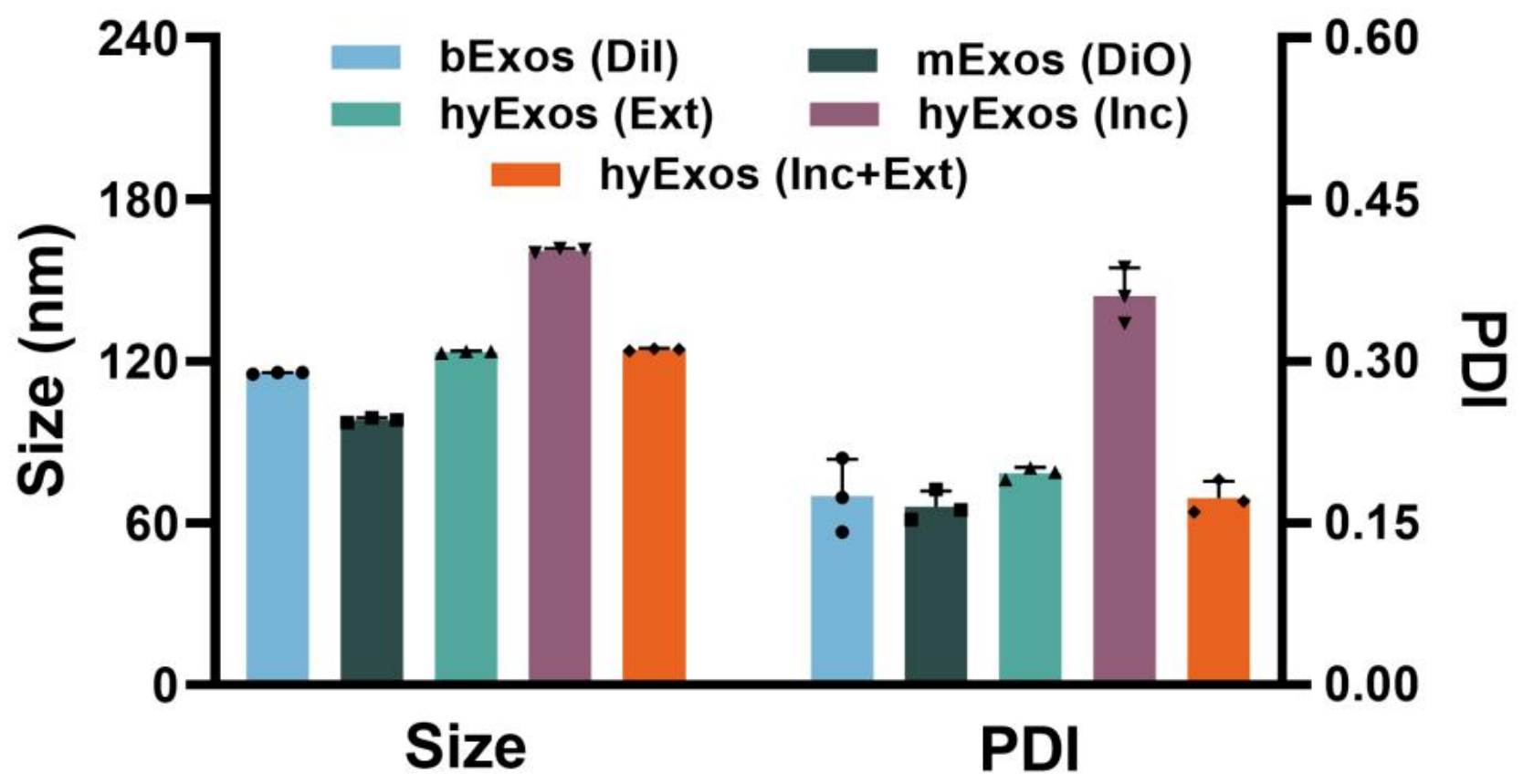

Supplementary Fig. 12 | SDS-PAGE analysis of protein profiles and compositional integrity of exosomal formulations. Data are representative of three independent experiments with similar results.

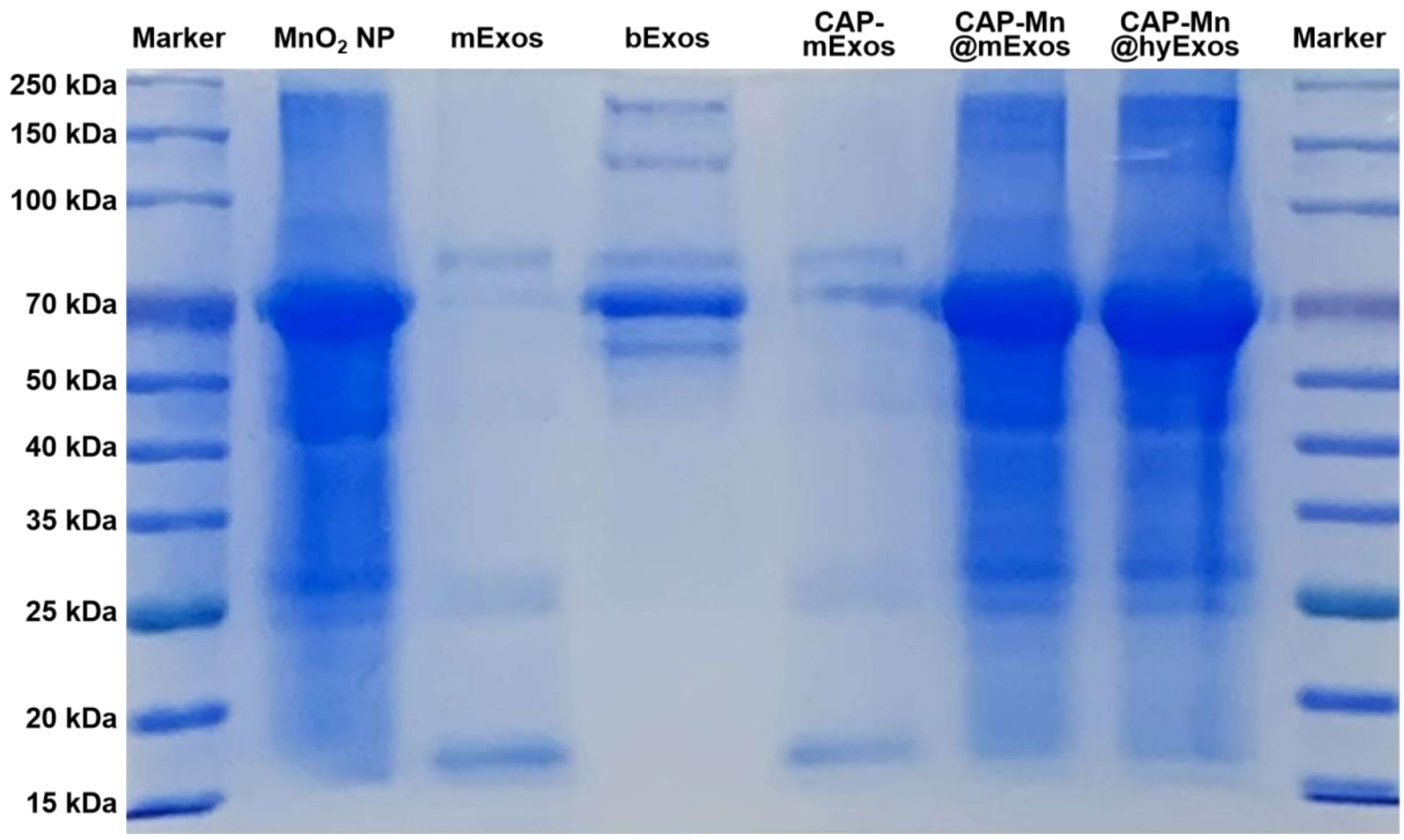

Supplementary Fig. 13 | Representative cryo-EM micrographs of MnO_2 NPs and various exosomal nanomotors, scale bar = 50 nm. Data are representative of three independent experiments with similar results.

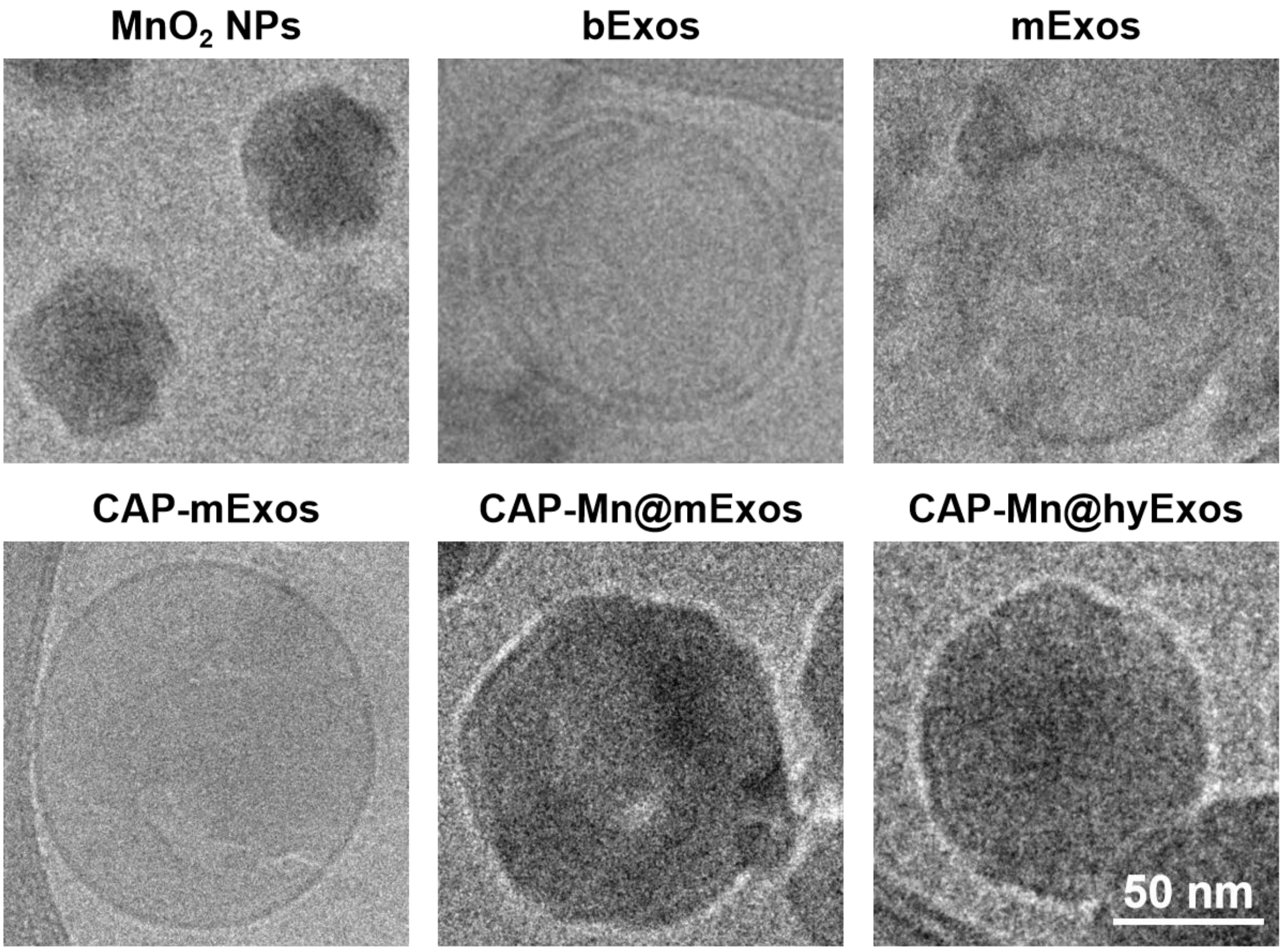

Supplementary Fig. 14 | Representative EDS mapping micrographs of CAP-hyExos and CAP-Mn@hyExos, scale bar = 50 nm. Data are representative of three independent experiments with similar results.

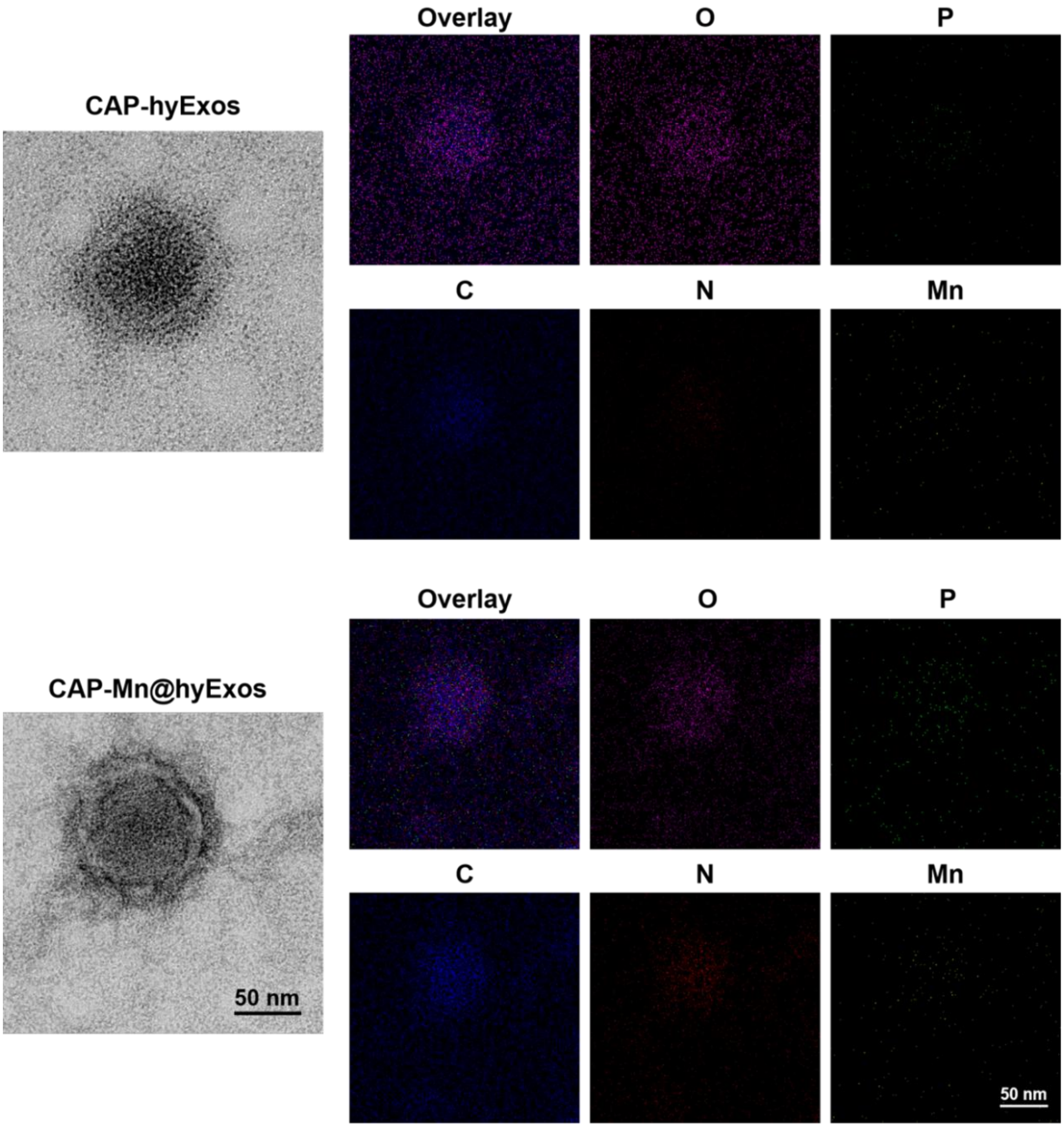

Supplementary Fig. 15 | DLS analysis of hydrodynamic size and PDI of exosomal formulations in simulated synovial fluid over 7 days (n = 3, biological replicates per group). Data are presented as the mean ± SD. Source data are provided as a Source Data file.

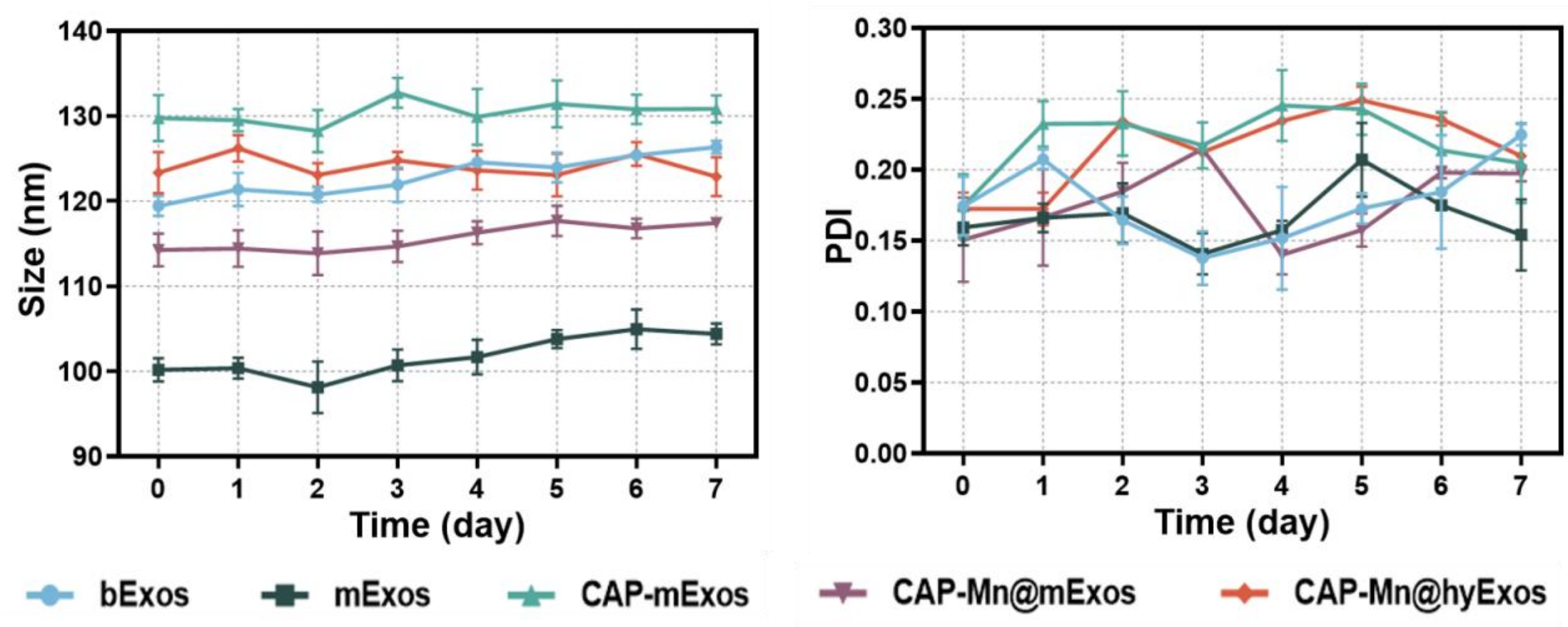

Supplementary Fig. 16 | DLS analysis of CAP-Mn@hyExos PDI over 7 days under OA-mimicking conditions (n = 3, biological replicates per group). Data are presented as the mean ± SD. Source data are provided as a Source Data file.

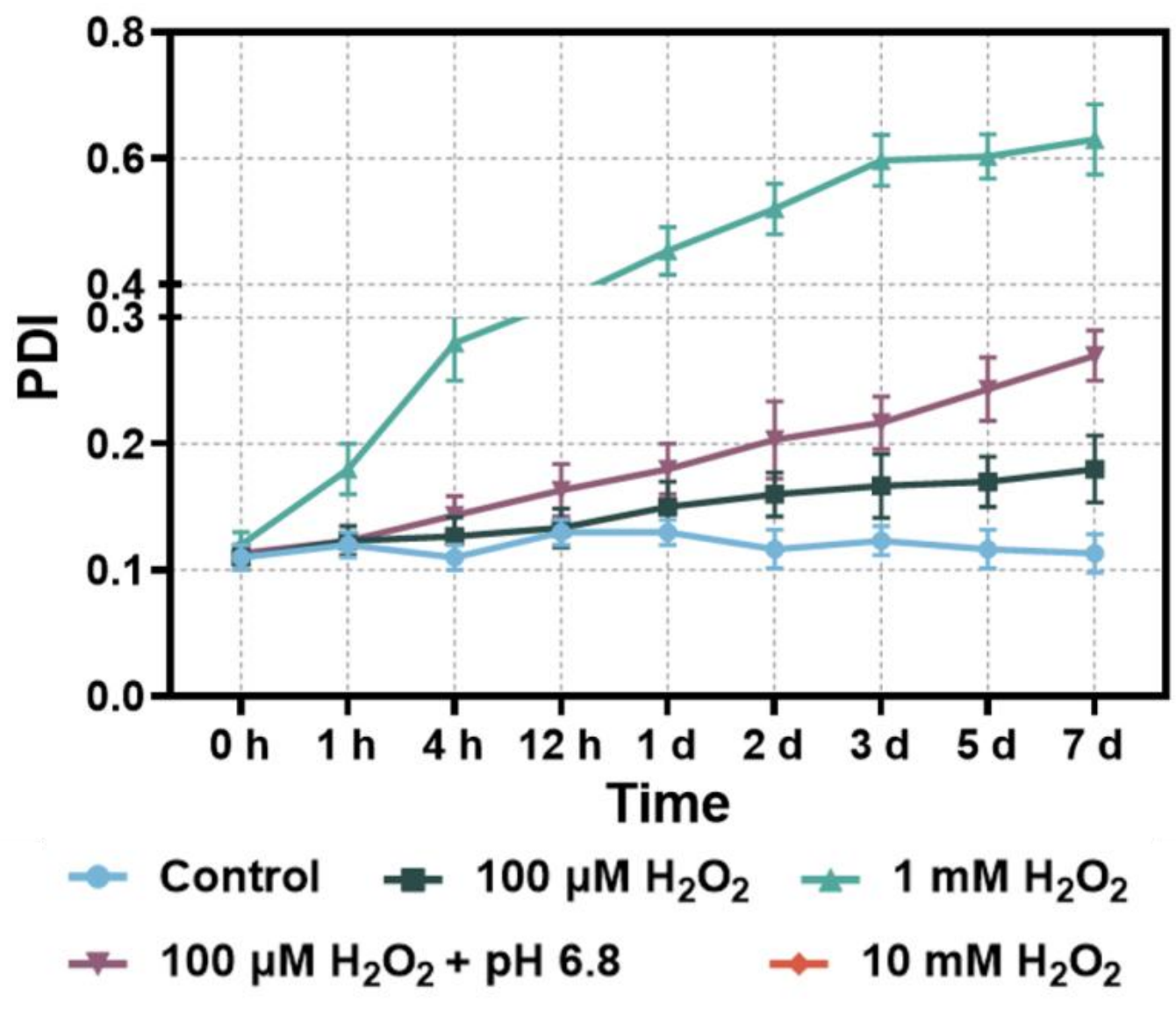

Supplementary Fig. 17 | Particle velocity distribution of exosomal formulations with or without H_2O_2 (n = 20, biological replicates per group). Data are presented as the mean ± SD. Source data are provided as a Source Data file.

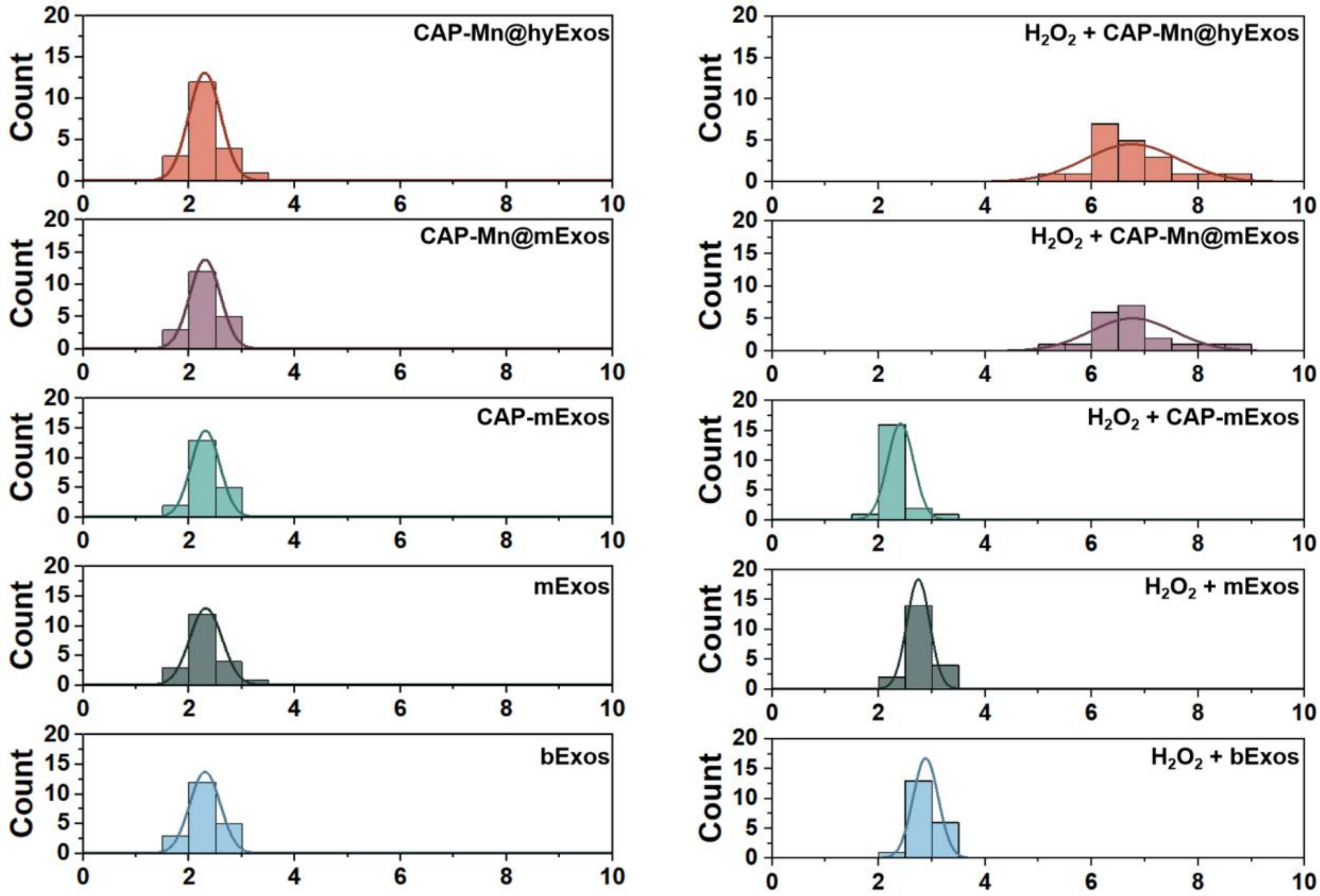

Supplementary Fig. 18 | Schematic illustration and confocal microscopy images showing the motion and migration of exosomal formulations under H_2O_2-enriched or control conditions in a Y-shaped microfluidic chip, scale bars = 5 μm. Created in Microsoft PowerPoint. Data are representative of three independent experiments with similar results.

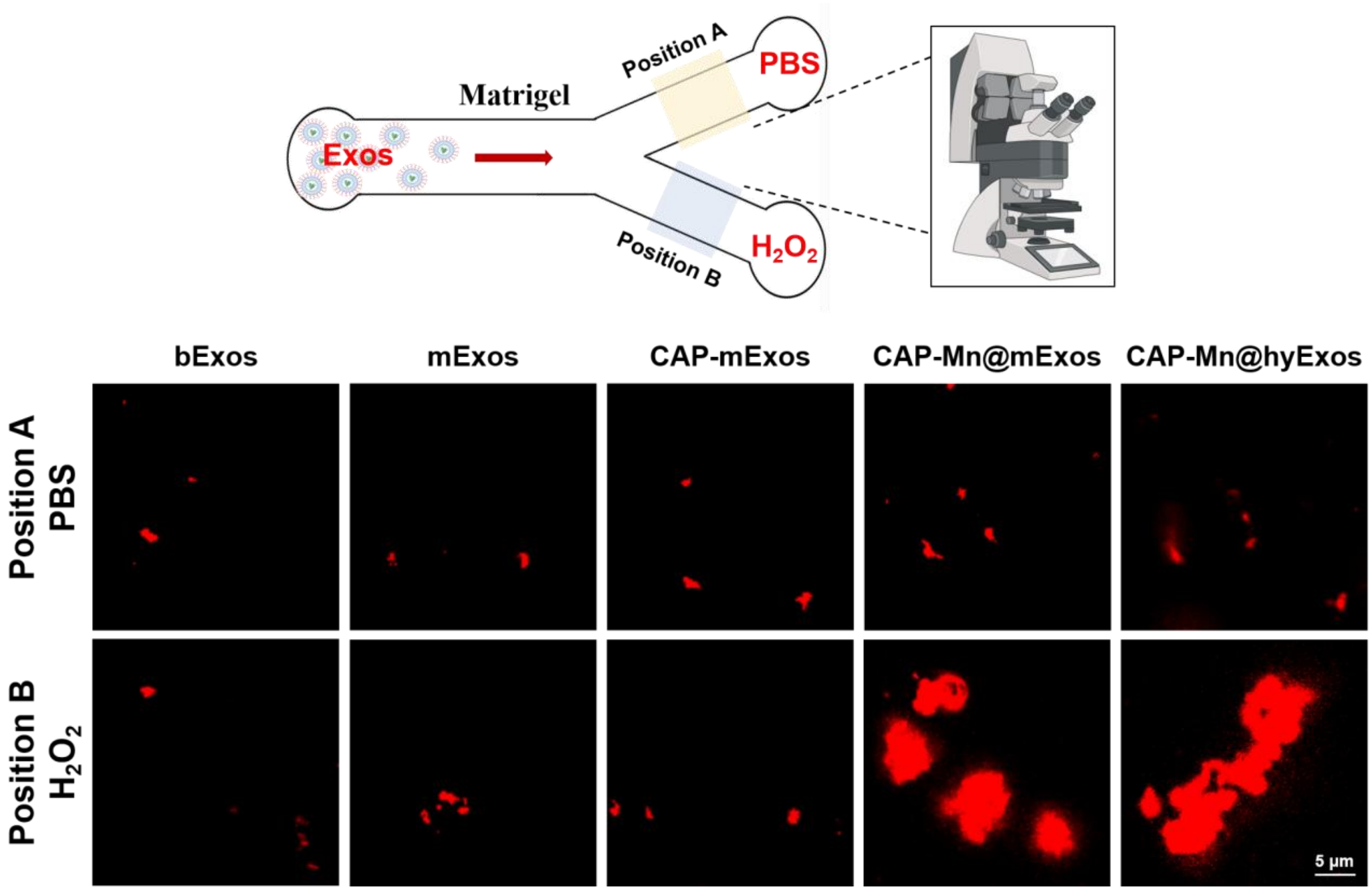

Supplementary Fig. 19 | Schematic illustration and confocal microscopy images showing the motion and migration of CAP-Mn@hyExos under various microenvironmental conditions, including PBS, urea, glucose, and ROS-related species (H_2O_2 and O_2^-), highlighting the stimulus-selective behavior of the nanomotors, scale bars = 5 μm. Created in Microsoft PowerPoint. Data are representative of three independent experiments with similar results.

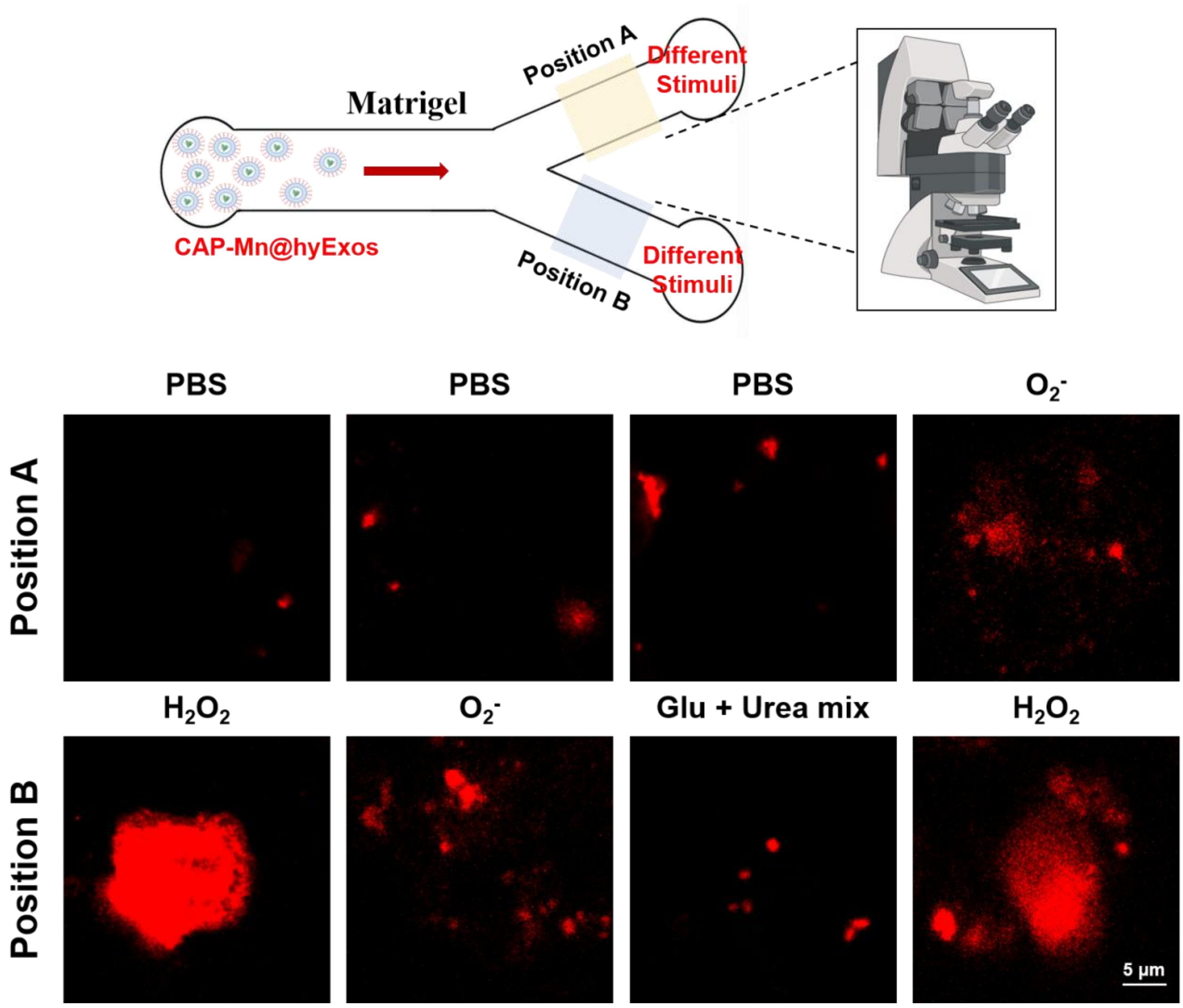

Supplementary Fig. 20 | Confocal microscopy of ex vivo cartilage sections following incubation with DiI-labeled Mn@hyExos and CAP-Mn@hyExos for 2, 4, and 6 days, scale bar = 50 μm. Data are representative of three independent experiments with similar results.

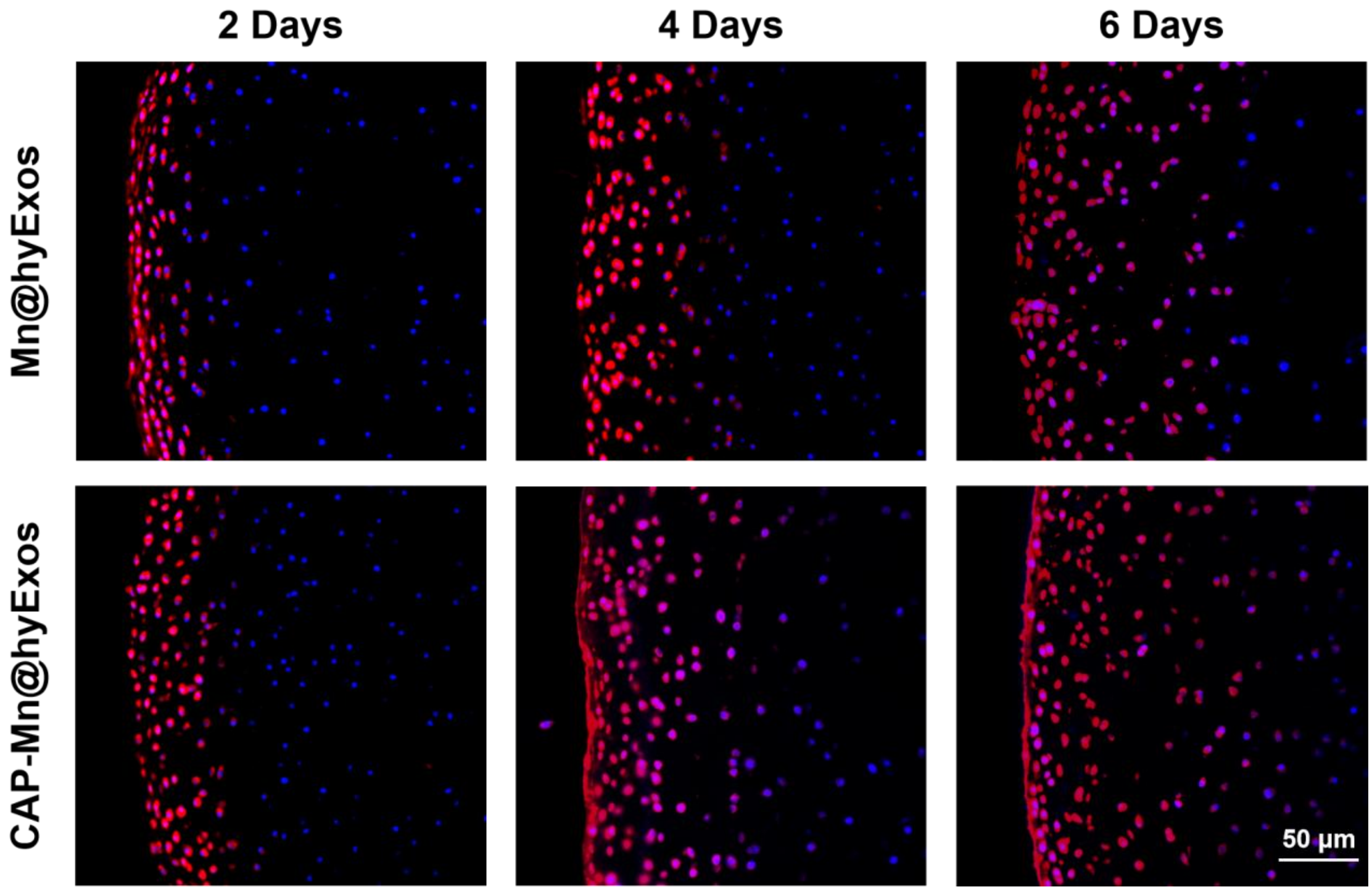

Supplementary Fig. 21 | Three-dimensional characterization of exosome localization in cartilage after ex vivo penetration. Representative two-dimensional confocal images, three-dimensional reconstructed images, and orthogonal views showing the spatial relationship between DiI-labeled CAP-Mn@mExos (red) and DAPI-stained nuclei (blue) following 6 days of incubation with cartilage explants. Enlarged views highlight the preferential localization of exosomes in cell-rich cartilage regions. scale bars = 50 μm. Data are representative of three independent experiments with similar results.

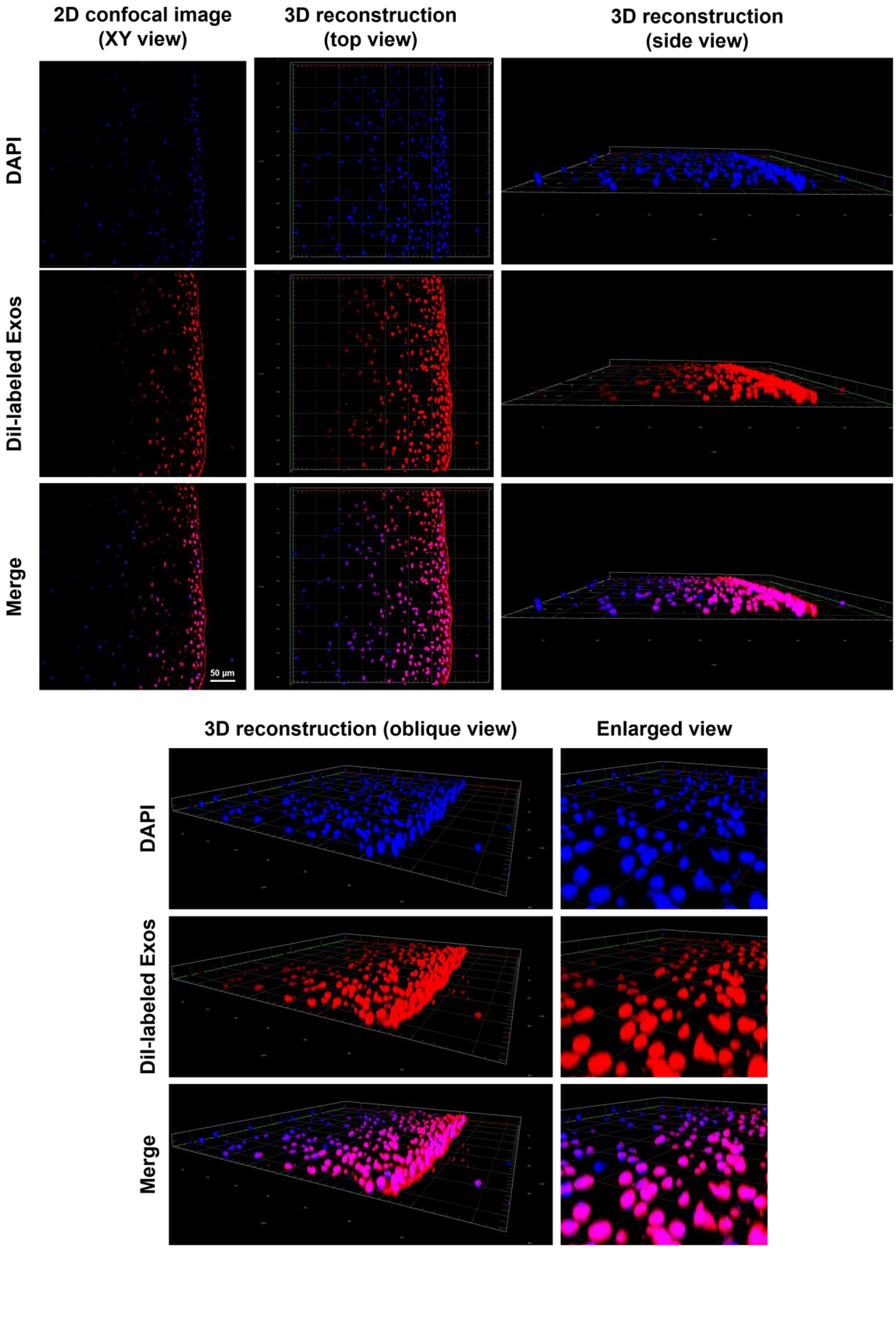

Supplementary Fig. 22 | Fluorescence imaging of ROS levels and spatial distribution in healthy and osteoarthritic knee cartilage, scale bars = 100 μm. Data are representative of three independent experiments with similar results.

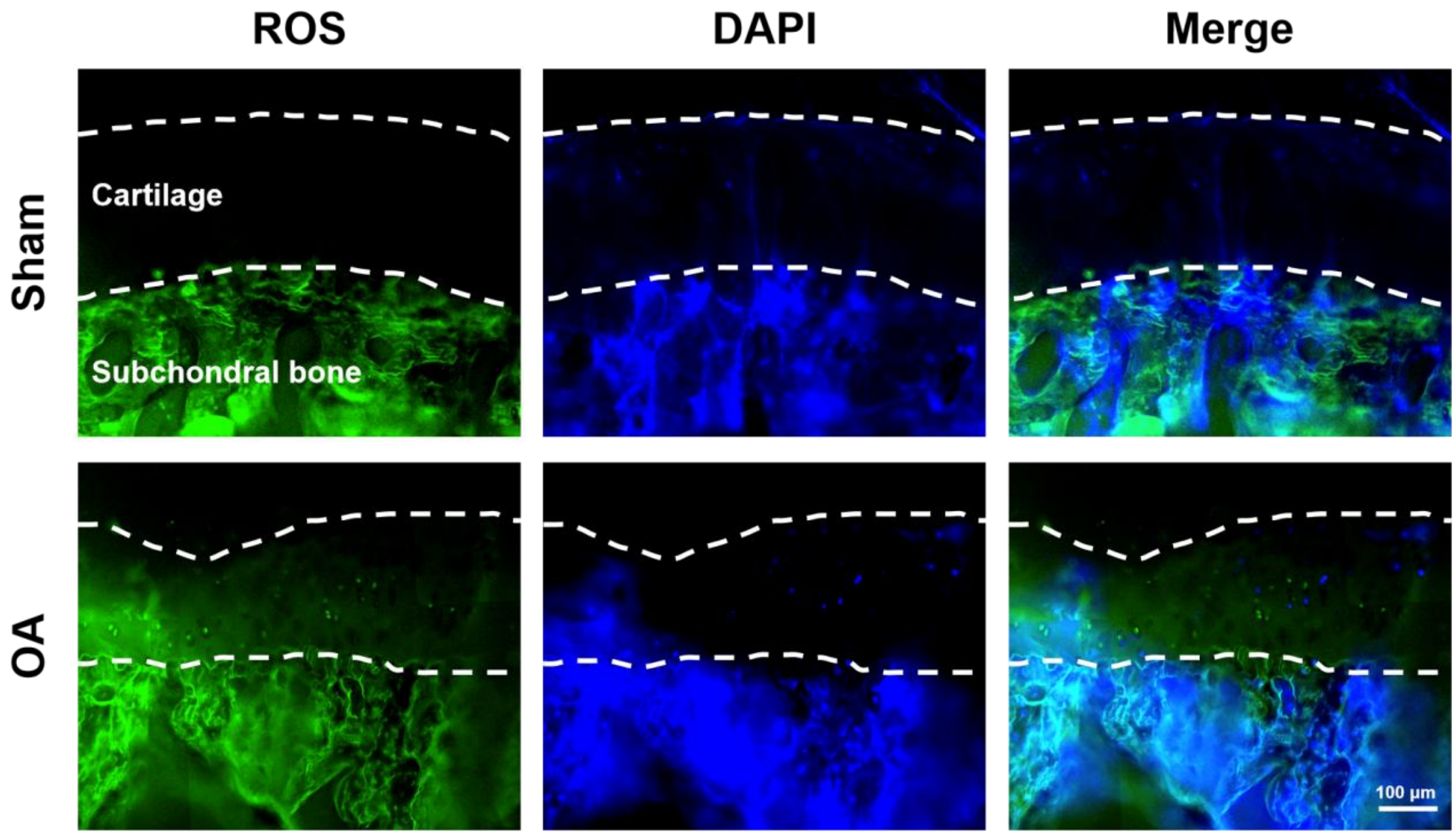

Supplementary Fig. 23 | Fluorescence images of cartilage from OA rat knees 2 days after intra-articular injection of DiI-labeled exosomal formulations, showing ROS-driven tissue penetration in vivo, scale bars = 50 μm. Data are representative of three independent experiments with similar results.

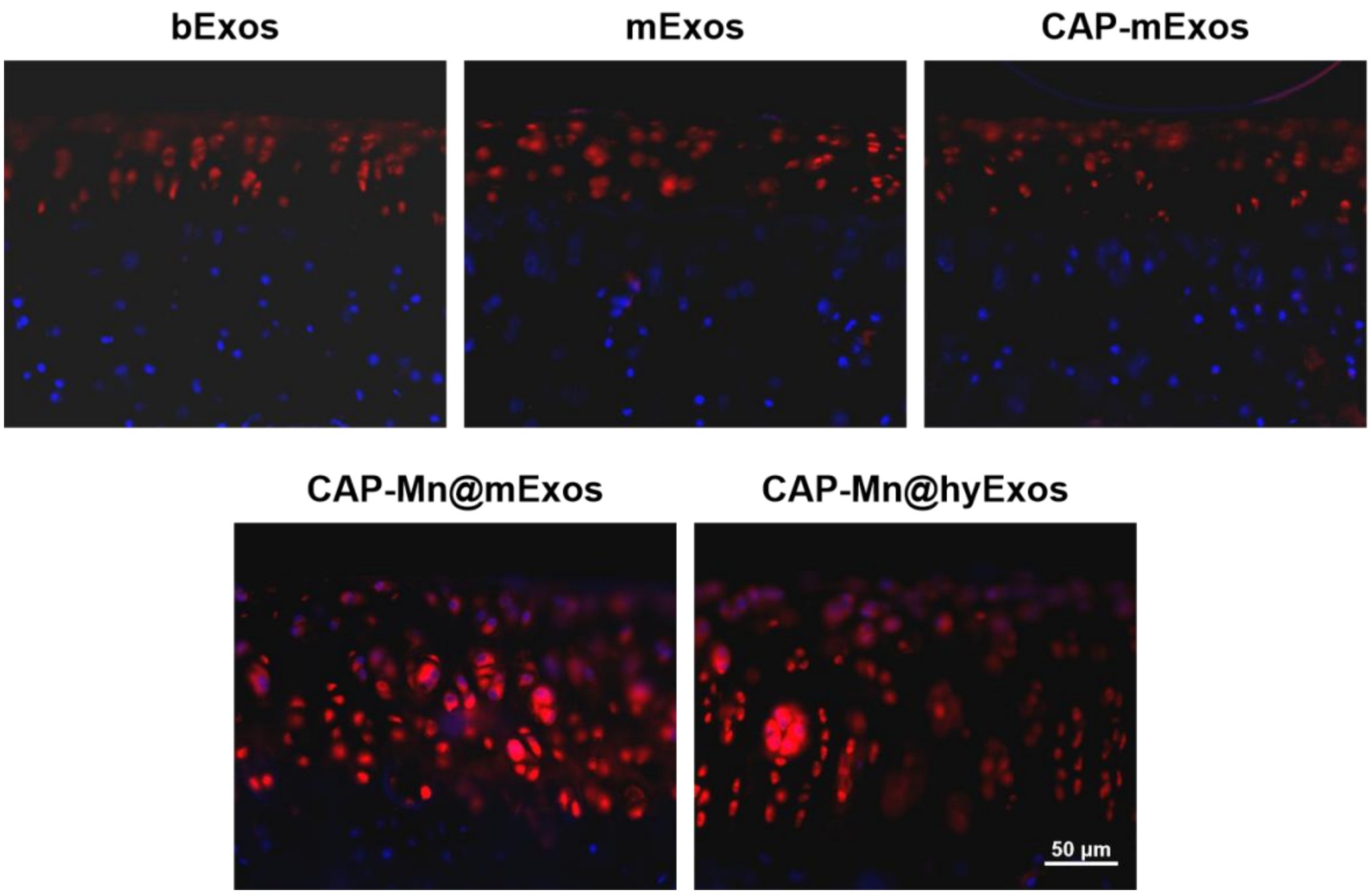

Supplementary Fig. 24 | In vivo imaging of CAP-Mn@hyExos retention in knee joints after intra-articular injection in OA and healthy rats (n = 3, biological replicates per group). Images are representative of three independent experiment with similar results. Data are presented as mean ± SD. Source data are provided as a Source Data file.

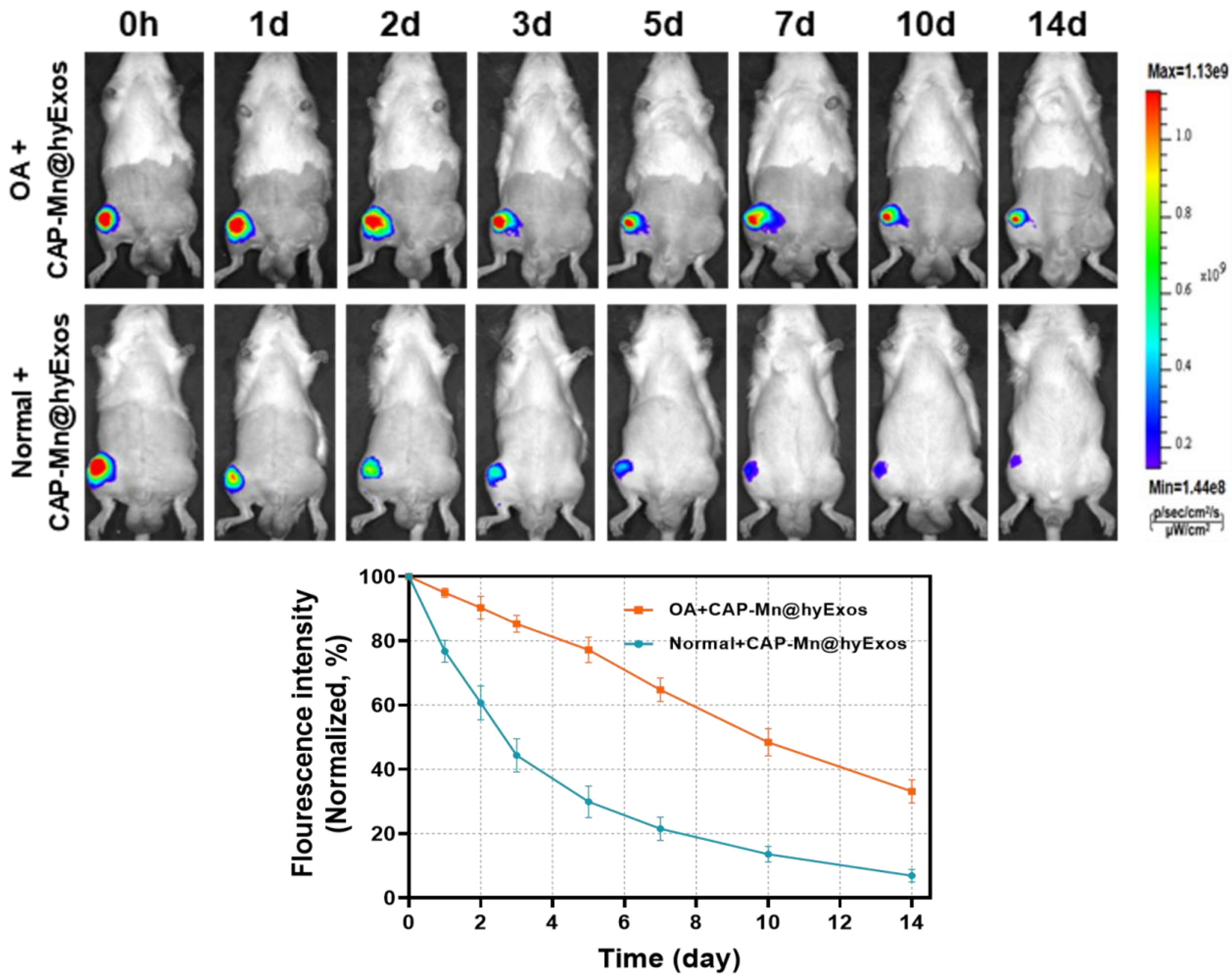

Supplementary Fig. 25 | In vivo imaging of major organs at 3 days, 2 weeks, and 8 weeks post intra-articular injection of exosomal formulations in OA rats to evaluate biodistribution and metabolic fate. Created in Microsoft PowerPoint. Data are representative of three independent experiments with similar results.

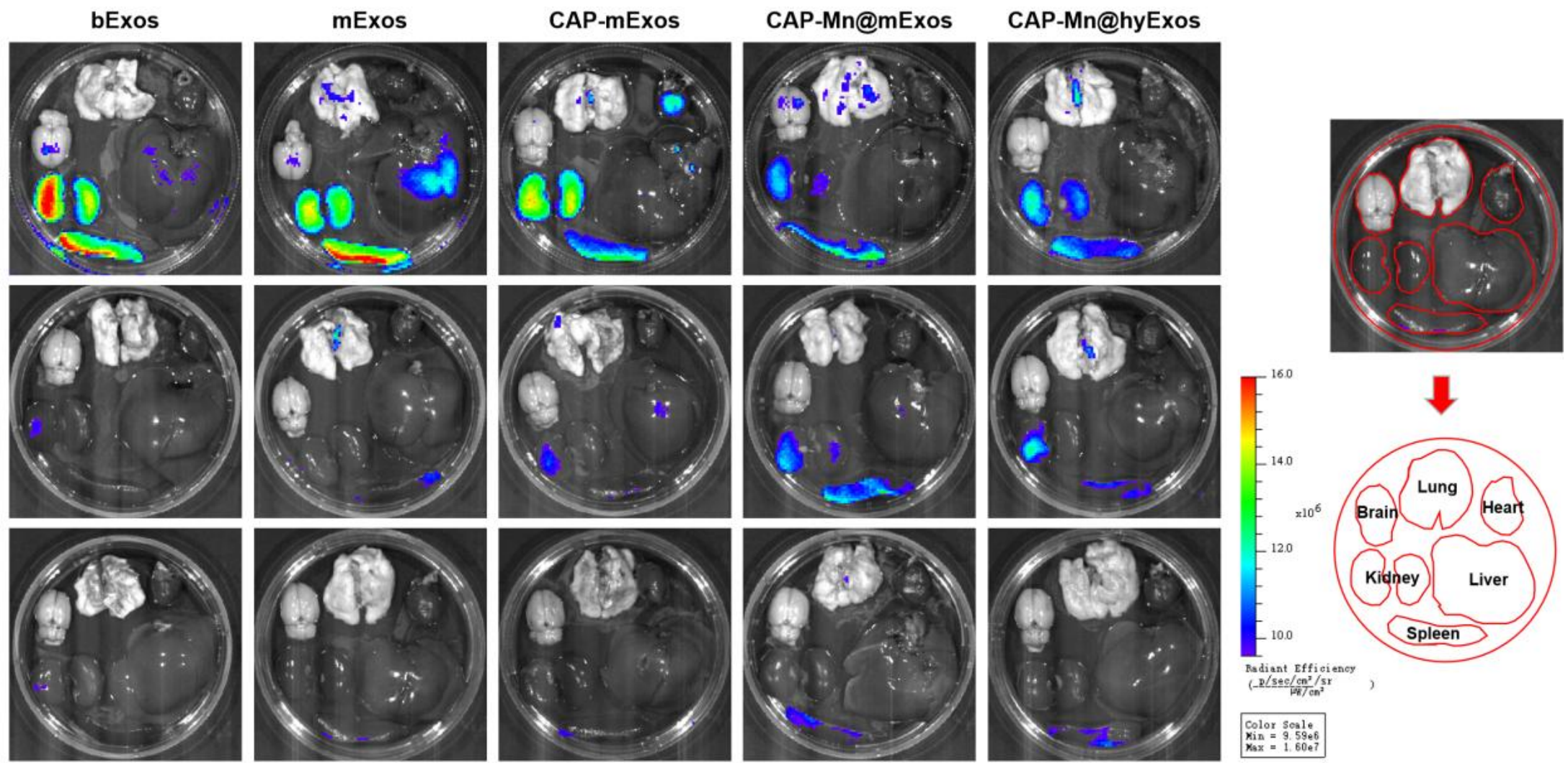

Supplementary Fig. 26 | ICP-OES quantification of manganese content in joint, spleen, kidney, liver, and brain at 3 days, 2 weeks, and 8 weeks post intra-articular injection of CAP-Mn@hyExos or PBS control in OA rats (n = 3, biological replicates per group). Data are presented as mean ± SD. *P* values are from paired two-tailed Student's t-test. Source data are provided as a Source Data file.

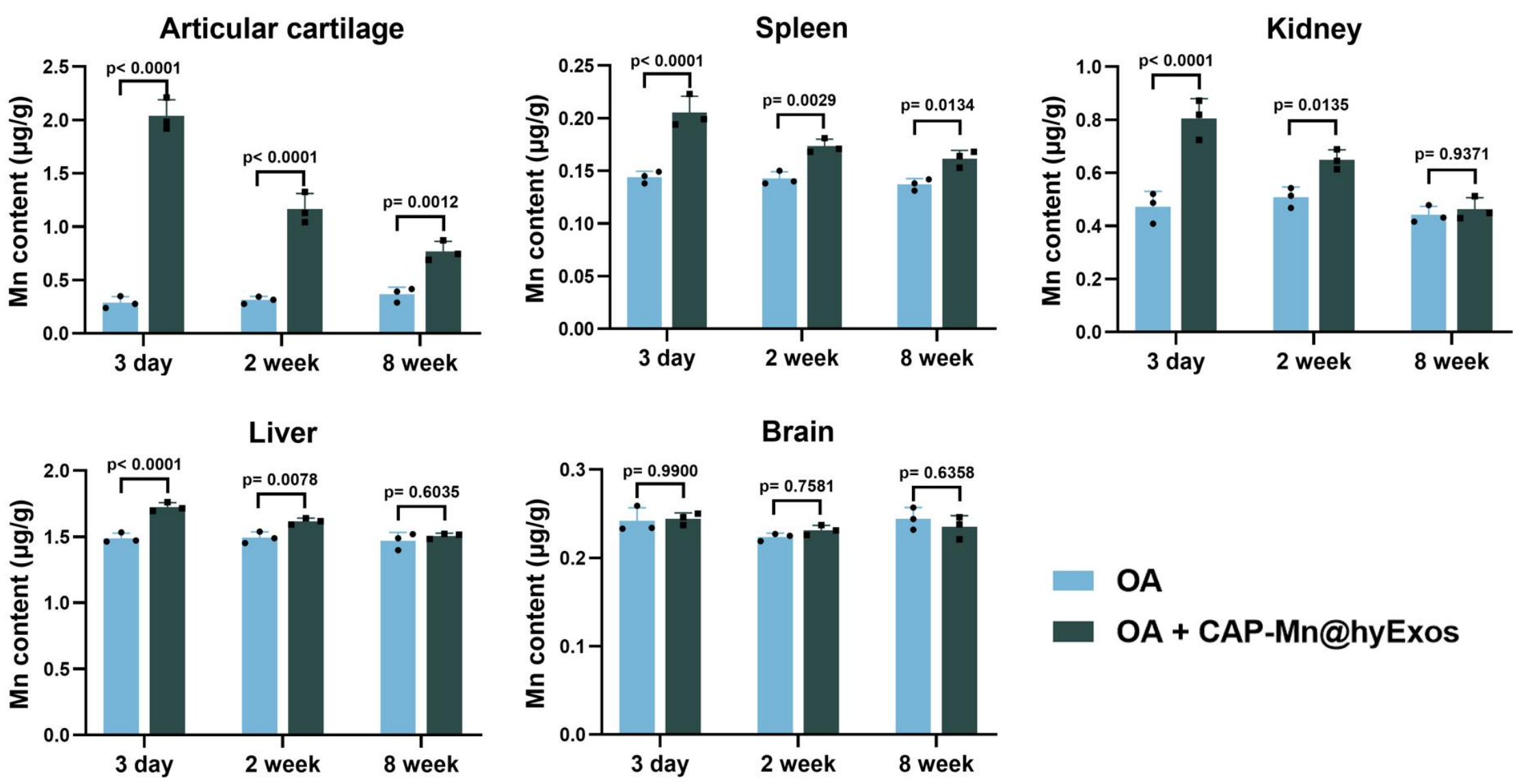

Supplementary Fig. 27 | Hematoxylin & Eosin staining of brain tissue at 8 weeks after intra-articular injection of exosomal formulations in OA rats to assess potential structural abnormalities, inflammatory infiltration, or pathological changes. Representative low-magnification images are shown in the upper panels, with corresponding higher-magnification views in the lower panels. Scale bars, 1000 μm (upper panels) and 200 μm (lower panels). Data are representative of three independent experiments with similar results.

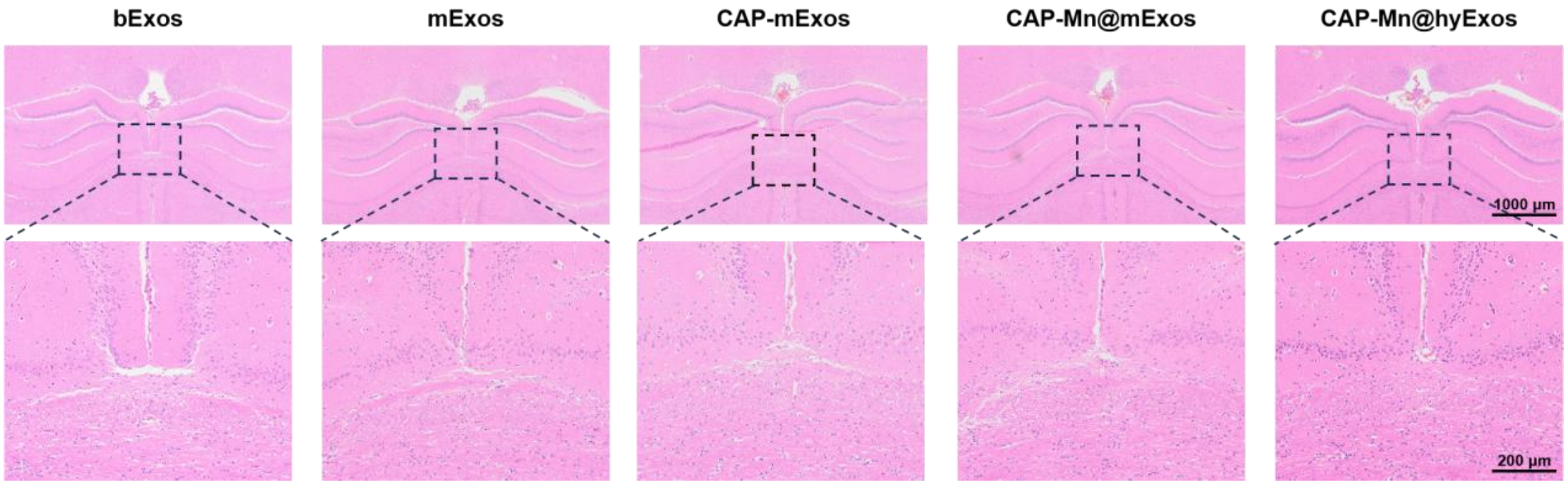

Supplementary Fig. 28 | Immunofluorescence images and related quantitative analysis of COL2A1, MMP13, and iNOS in OA chondrocytes treated with mExos, bExos, or 140@bExos. Images are representative of three independent experiment with similar results. Data are presented as the mean ± SD. *P* values are from one-way analysis of variance (ANOVA) with Tukey's multiple comparisons test. Source data are provided as a Source Data file.

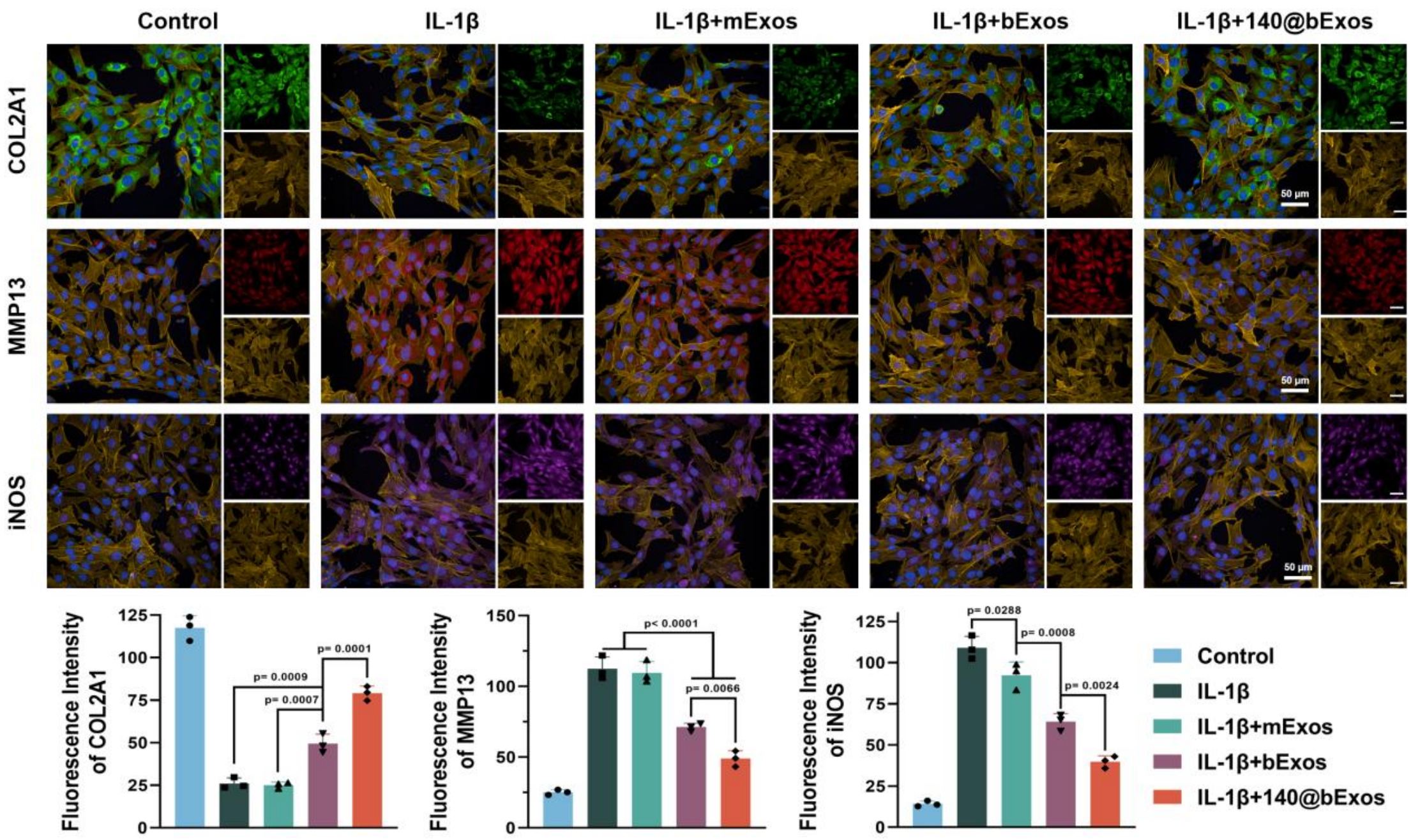

Supplementary Fig. 29 | Western blot analysis and related quantitative analysis of COL2A1, MMP13, and iNOS in OA chondrocytes treated with mExos, bExos, or 140@bExos. Images are representative of three independent experiment with similar results. Data are presented as the mean ± SD. *P* values are from one-way analysis of variance (ANOVA) with Tukey's multiple comparisons test. Source data are provided as a Source Data file.

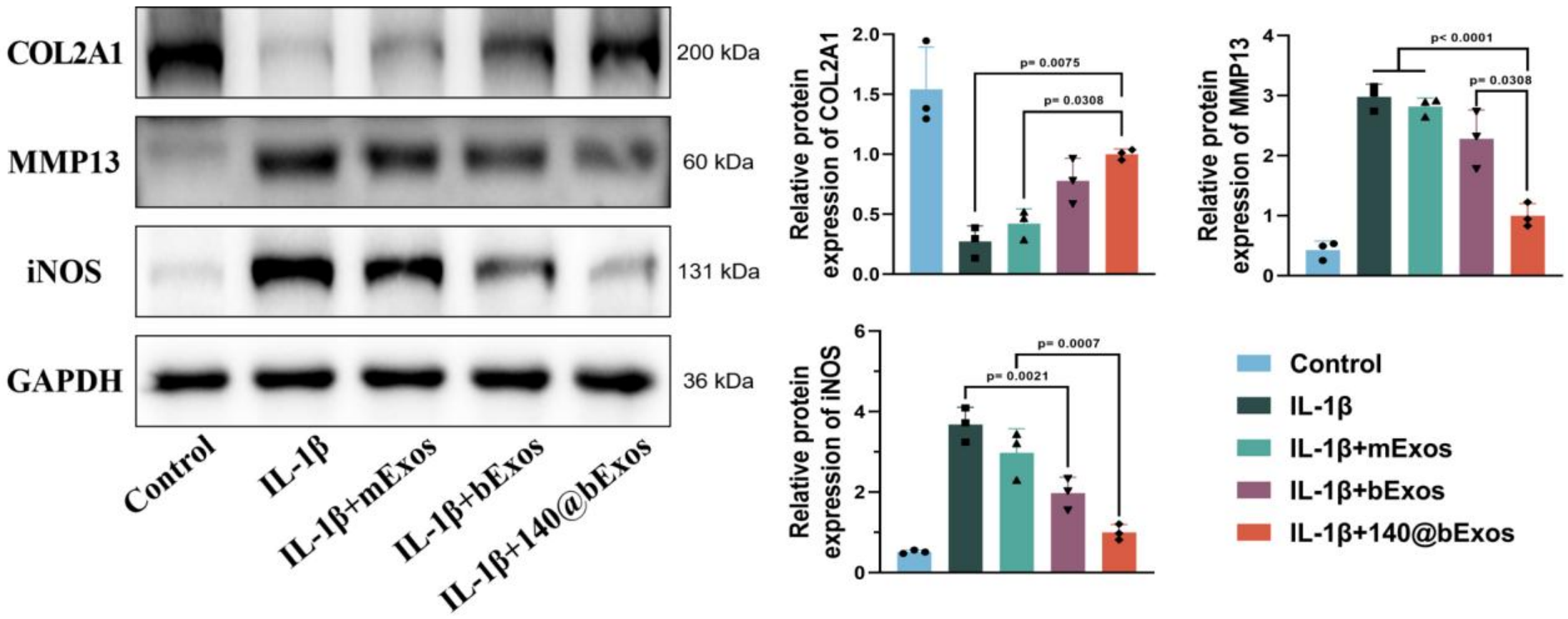

Supplementary Fig. 30 | qPCR analysis of *COL2a1*, *MMP13*, and *iNOS* in OA chondrocytes treated with mExos, bExos, or 140@bExos (n = 4, biological replicates per group). Data are presented as the mean ± SD. *P* values are from one-way analysis of variance (ANOVA) with Tukey's multiple comparisons test. Source data are provided as a Source Data file.

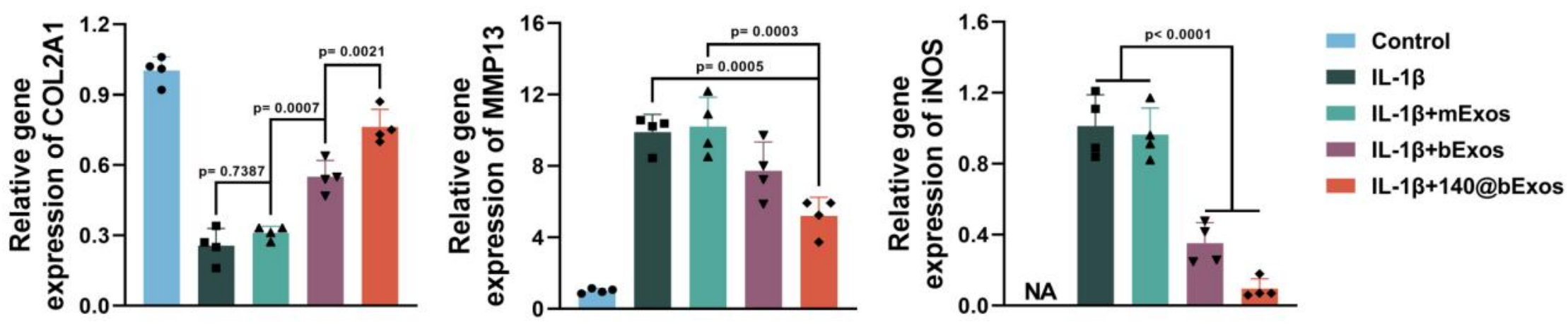

Supplementary Fig. 31 | Safranin O and Alcian Blue staining images and related quantitative analysis of COL2A1, MMP13, and iNOS in OA chondrocytes treated with mExos, bExos, or 140@bExos. Images are representative of three independent experiment with similar results. Data are presented as the mean ± SD. *P* values are from one-way analysis of variance (ANOVA) with Tukey's multiple comparisons test. Source data are provided as a Source Data file.

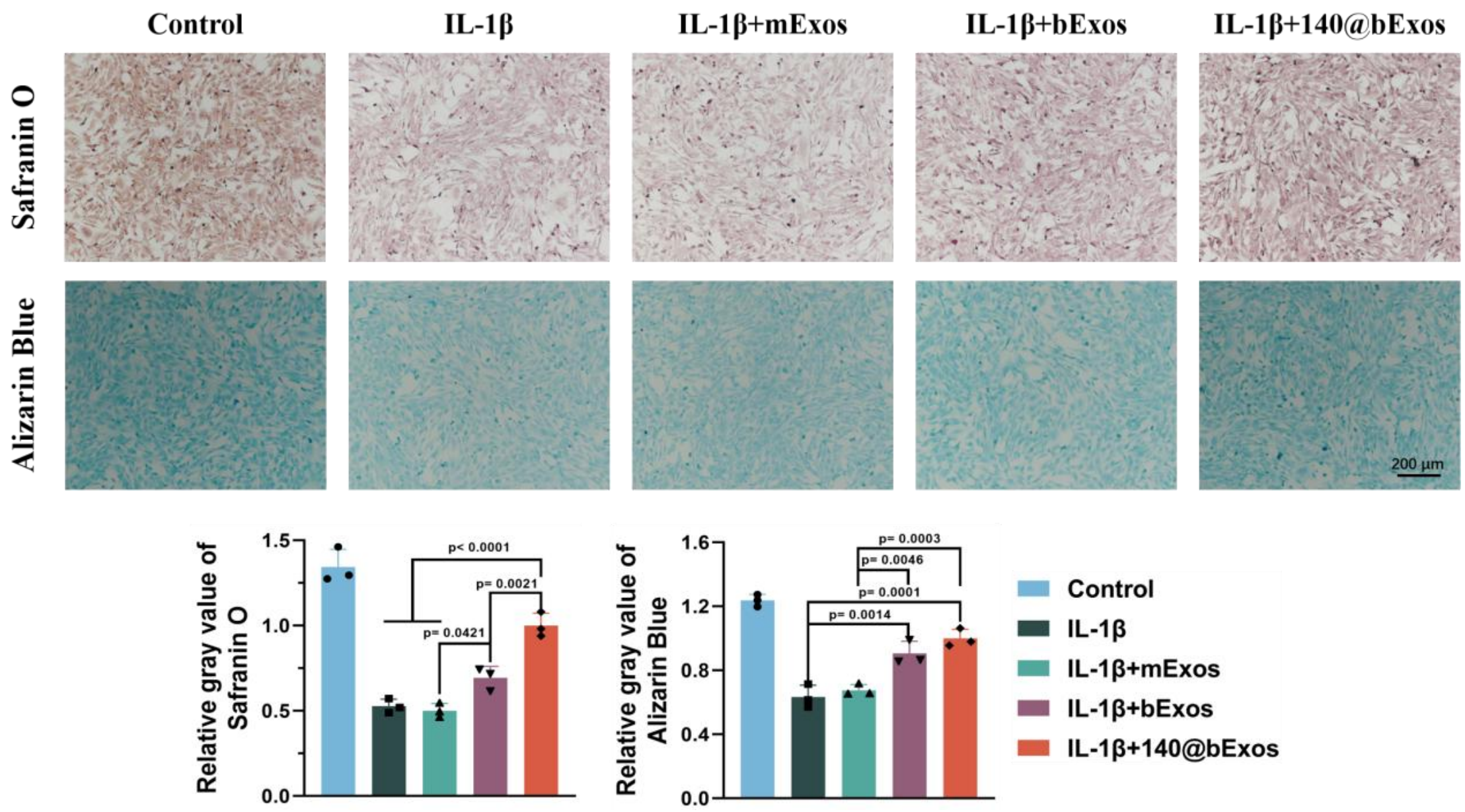

Supplementary Fig. 32 | Hemolysis assay with related quantitative analysis of various exosomal formulations to evaluate hemocompatibility. Images are representative of three independent experiment with similar results. Data are presented as the mean ± SD. Source data are provided as a Source Data file.

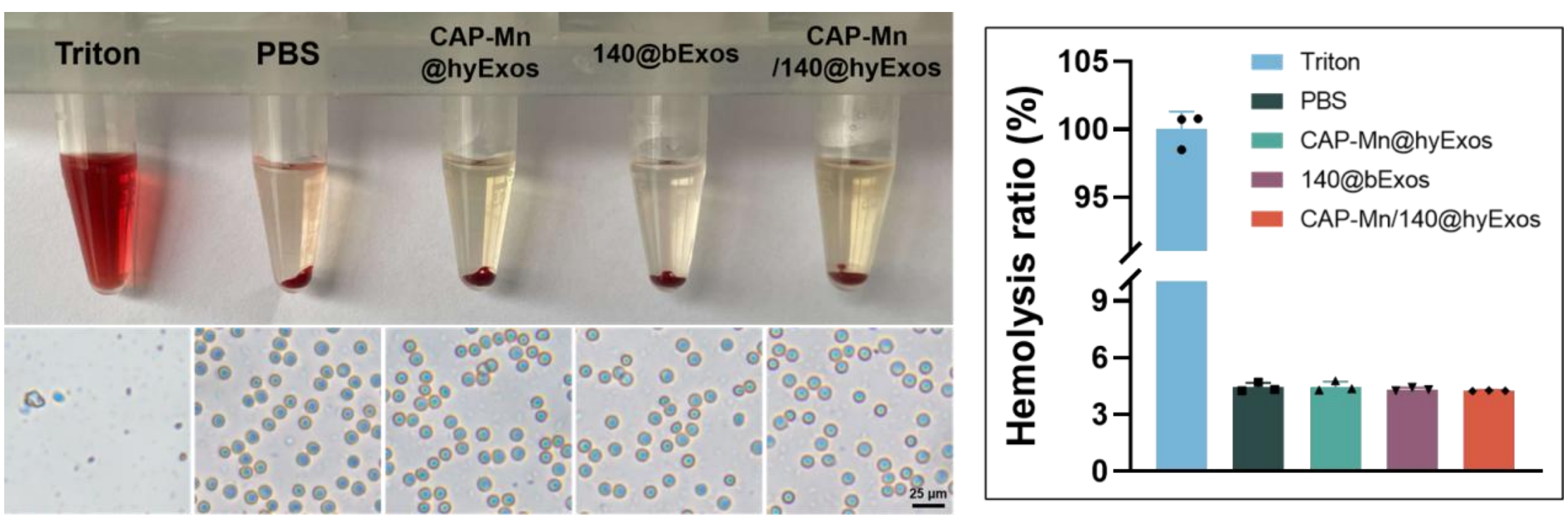

Supplementary Fig. 33 | IC_{50}-guided optimization of the H_2O_2 working concentration for establishment of the in vitro oxidative stress model (n = 5, biological replicates per group). Data are presented as the mean ± SD. Source data are provided as a Source Data file.

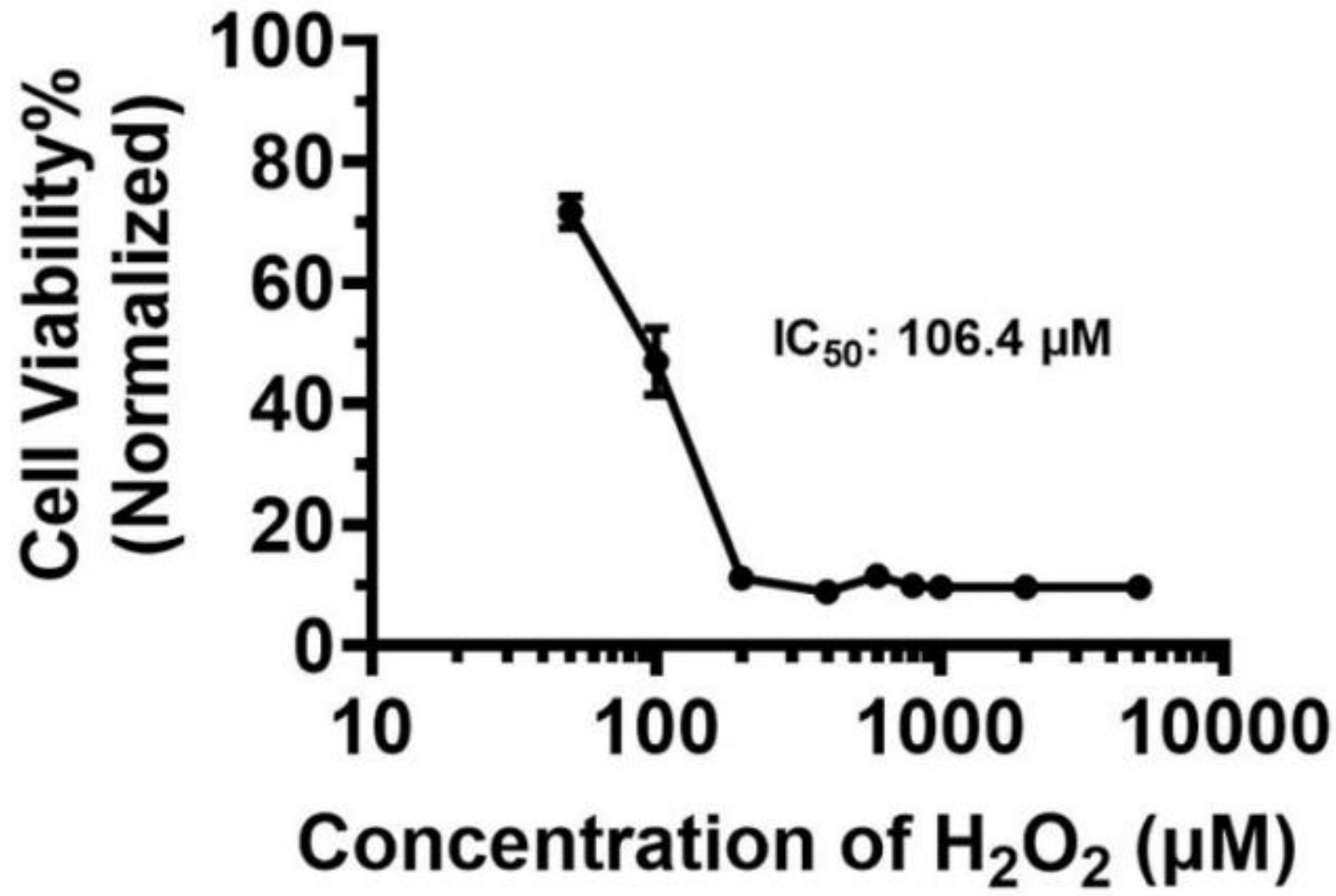

Supplementary Fig. 34 | Coronal (A) and sagittal (B) micro-CT images of tibial subchondral bone in OA rats at 4 and 8 weeks after treatment with different exosomal formulations. Data are representative of four independent experiments with similar results.

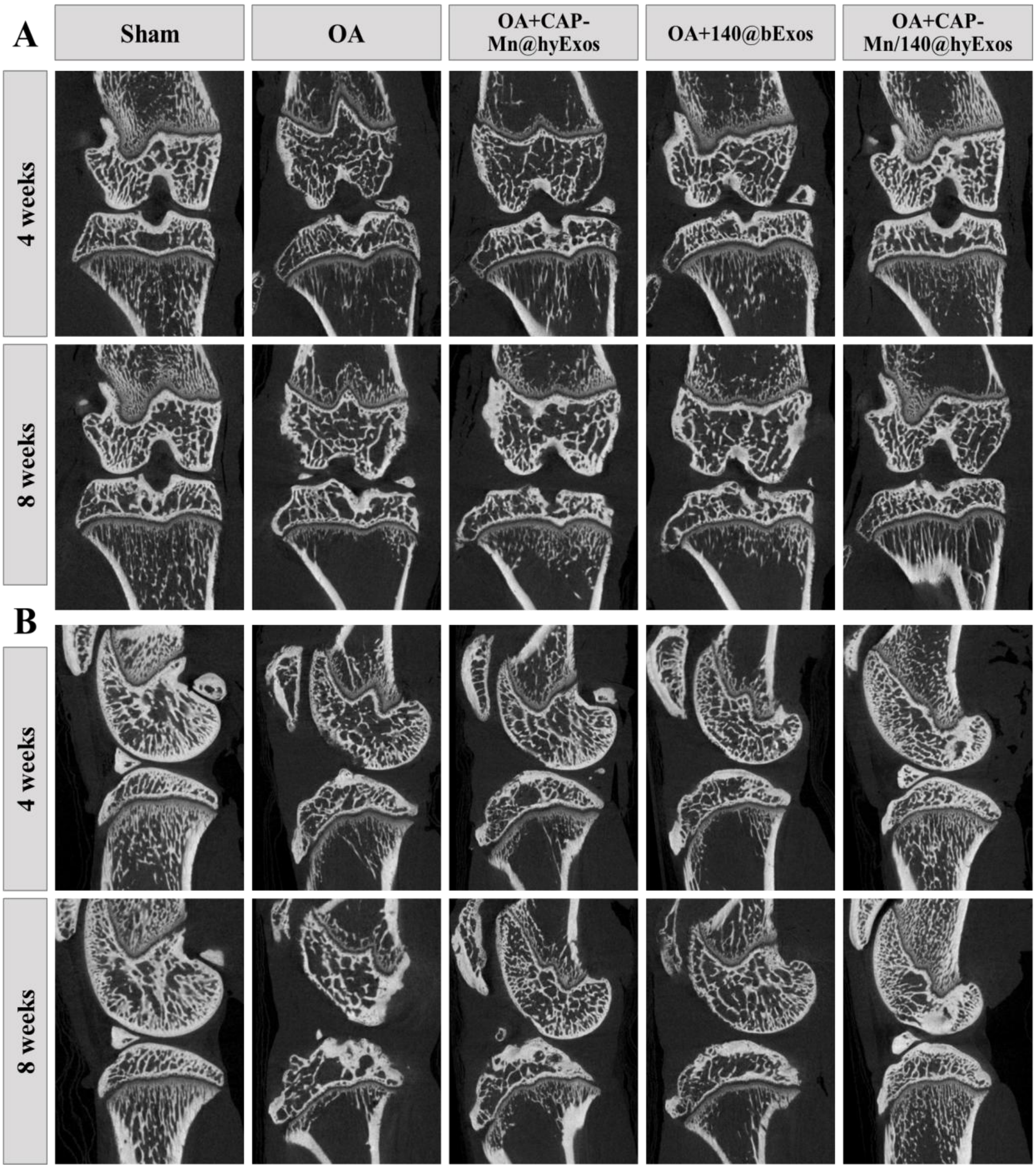

Supplementary Fig. 35 | Gross appearance of OA rat knee joints at 4 and 8 weeks after treatment with different exosomal formulations. Data are representative of four independent experiments with similar results.

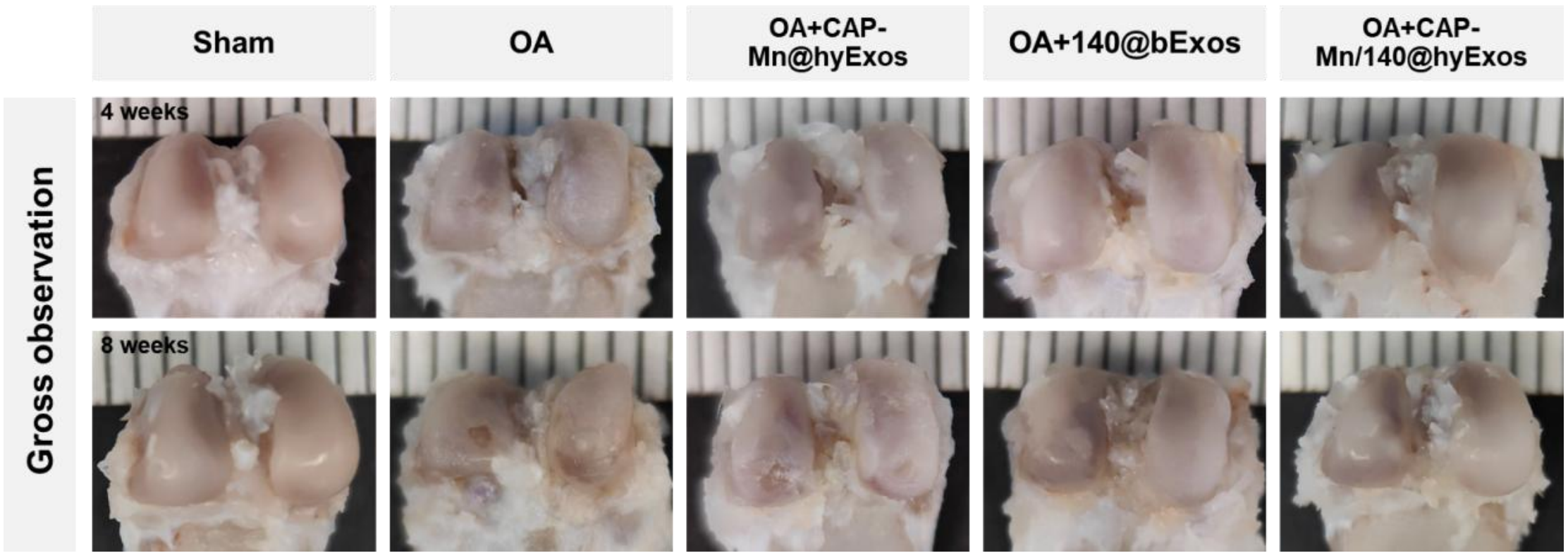

Supplementary Fig. 36 | Serum biochemical analysis of OA rats at 8 weeks after treatment with various exosomal formulations (n = 4, biological replicates per group). Data are presented as the mean ± SD. *P* values are from one-way analysis of variance (ANOVA) with Tukey's multiple comparisons test. Source data are provided as a Source Data file.

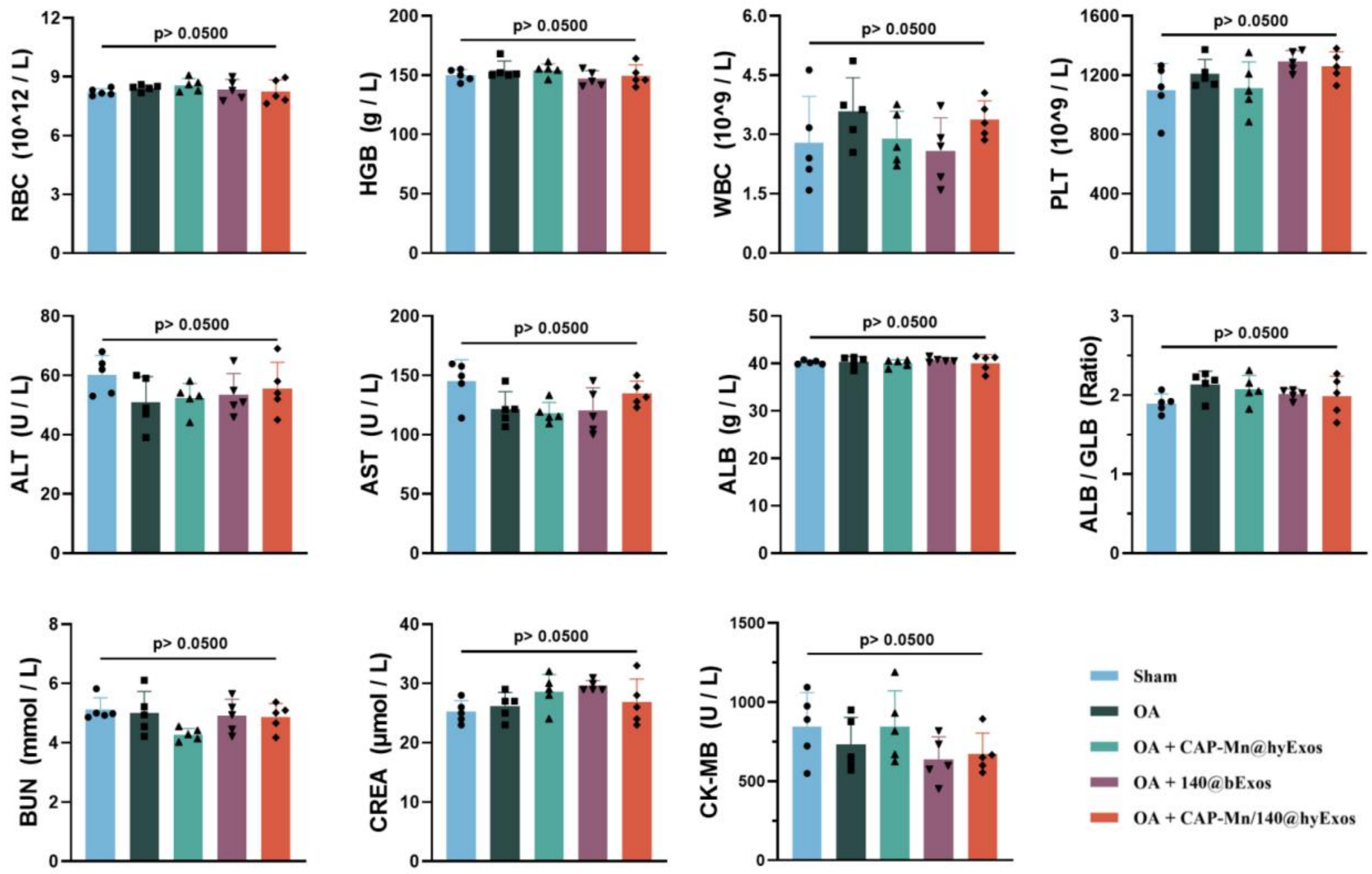

Supplementary Fig. 37 | Hematoxylin and eosin staining of major organs in OA rats at 8 weeks after treatment with various exosomal formulations, scale bar = 200 μm. Data are representative of four independent experiments with similar results.

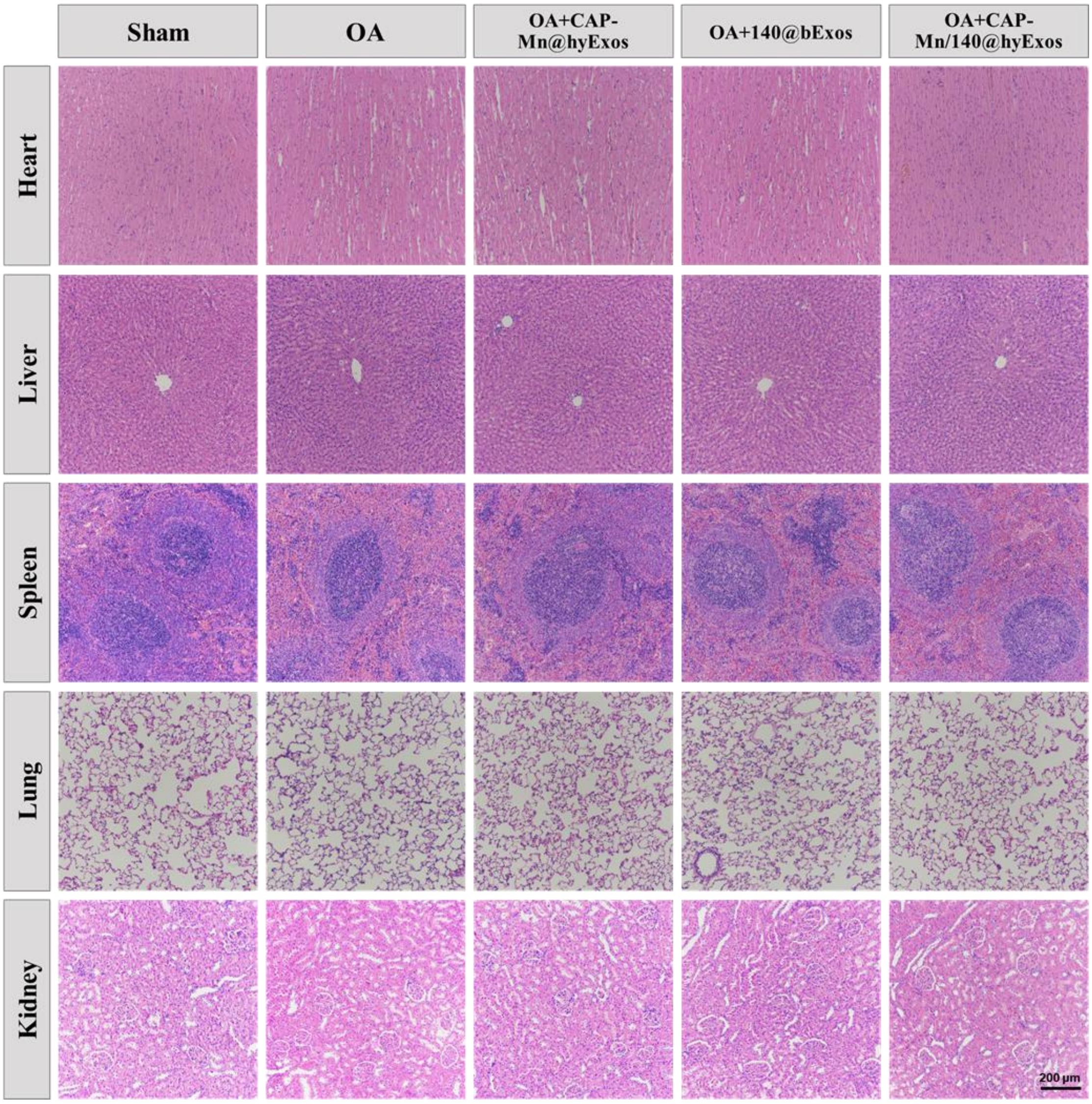

Supplementary Fig. 38 | Principal component analysis (PCA) of RNA-seq transcriptomic profiles from OA control and CAP-Mn/140@hyExos-treated groups (n = 3, biological replicates per group).

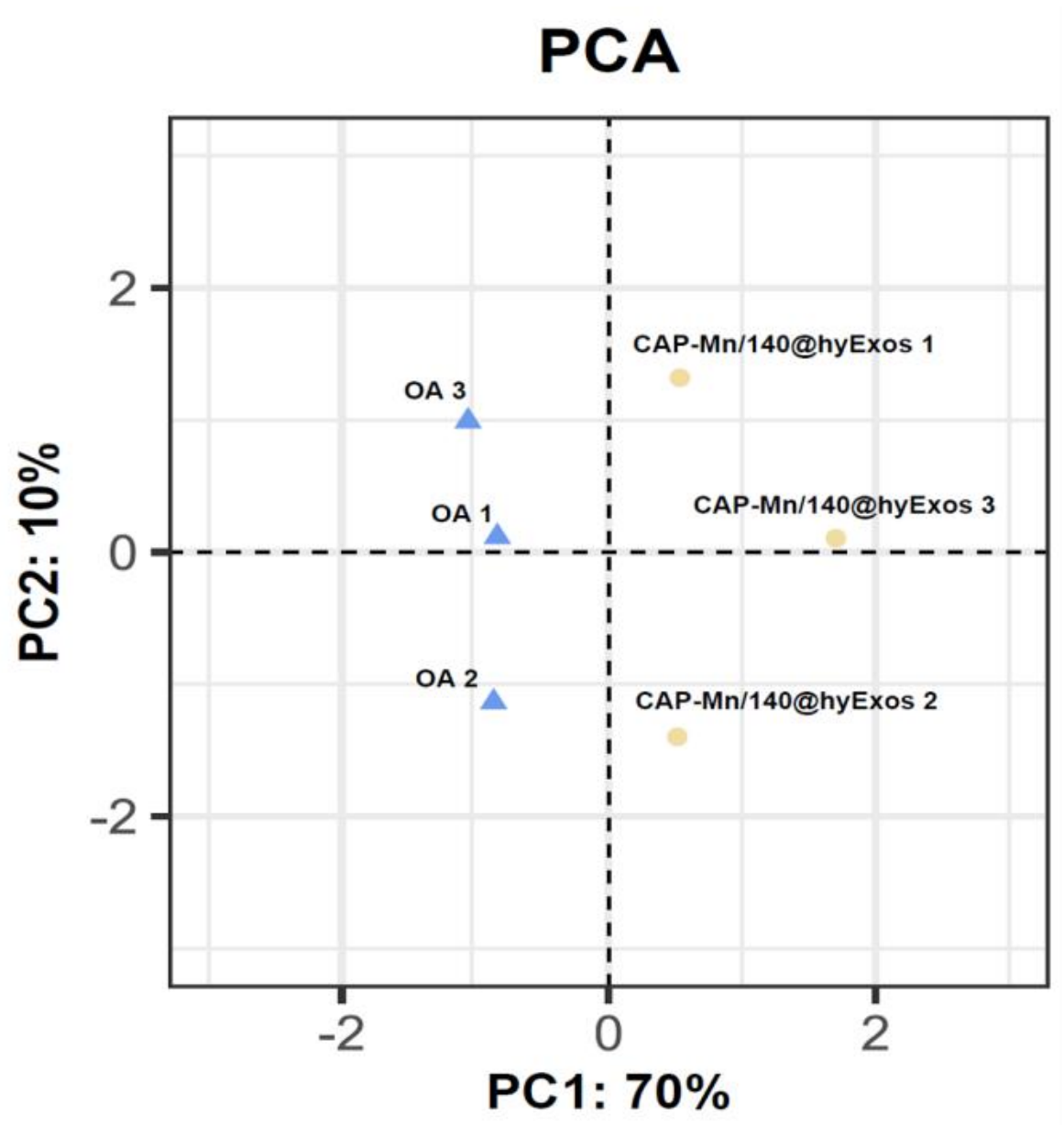

Supplementary Fig. 39 | Pearson correlation analysis of RNA-seq transcriptomic profiles from OA control and CAP-Mn/140@hyExos-treated groups (n = 3, biological replicates per group).

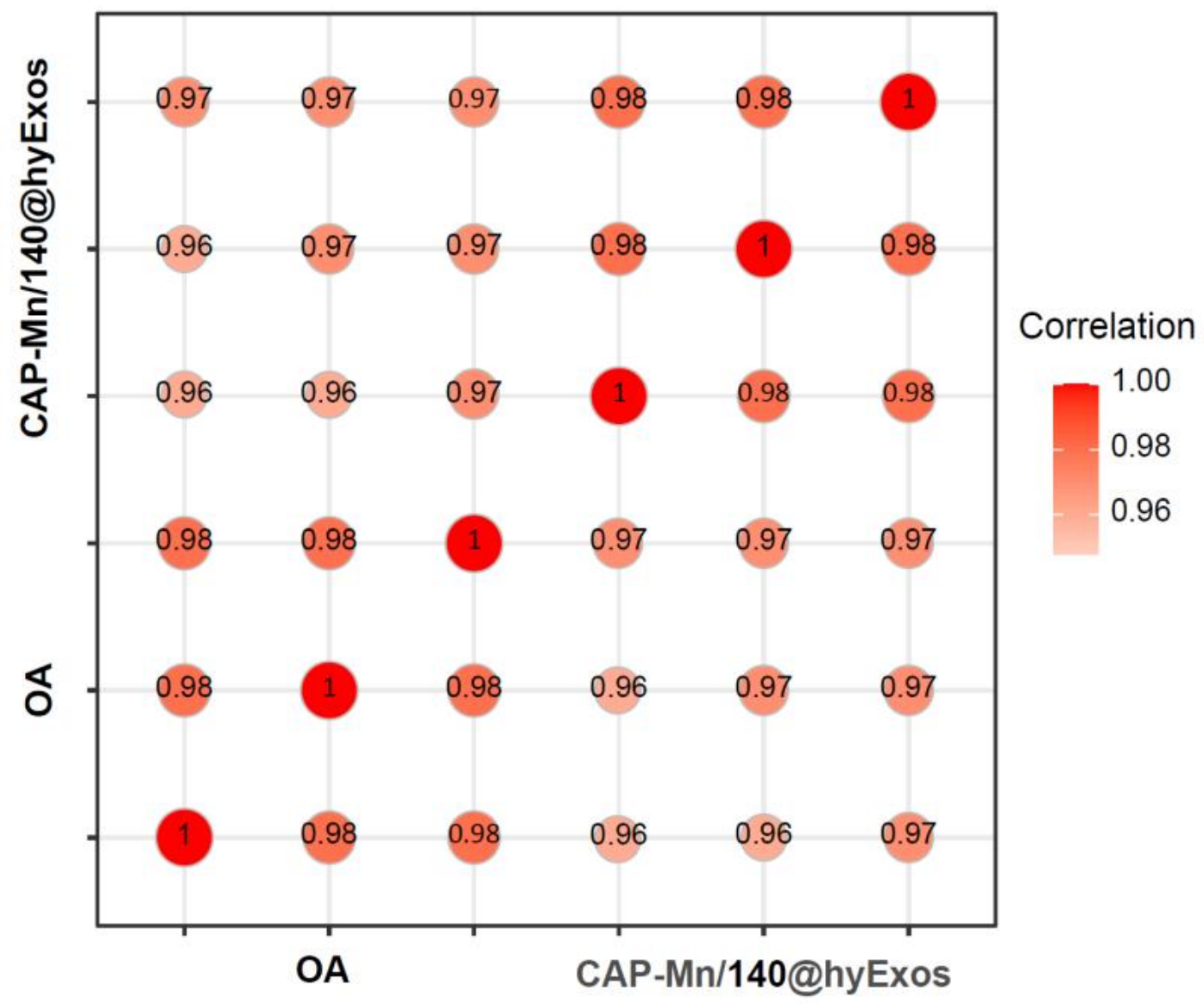

Supplementary Fig. 40 | Expression levels of key genes involved in oxidative detoxification, mitochondrial oxidative phosphorylation, and anti-inflammatory immune regulation across OA control and CAP-Mn/140@hyExos-treated groups (n = 3, biological replicates per group).

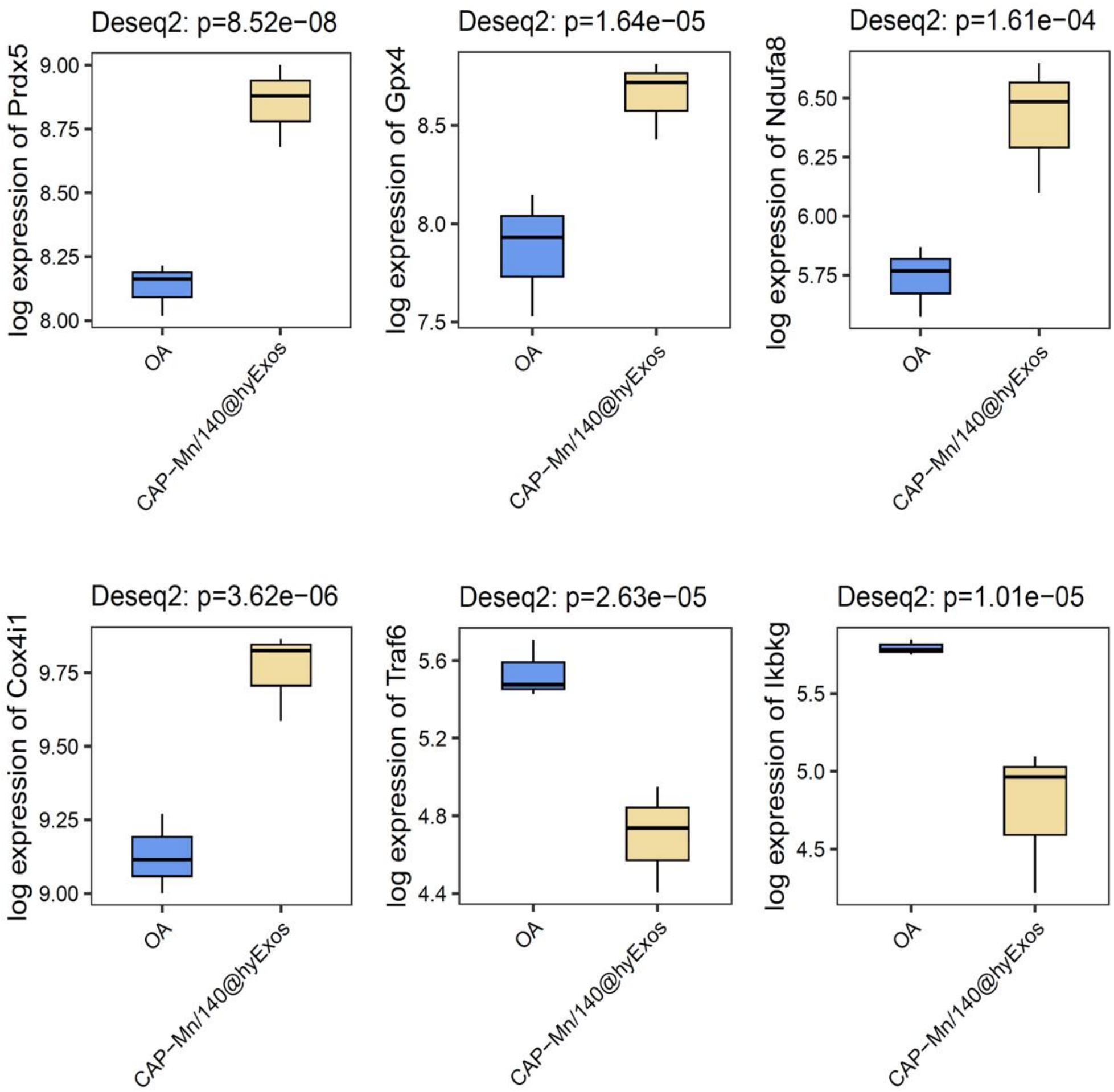

Supplementary Fig. 41 | Representative flow cytometry gating strategy for identifying the analyzed cell populations in the control group shown in Fig. 5F.

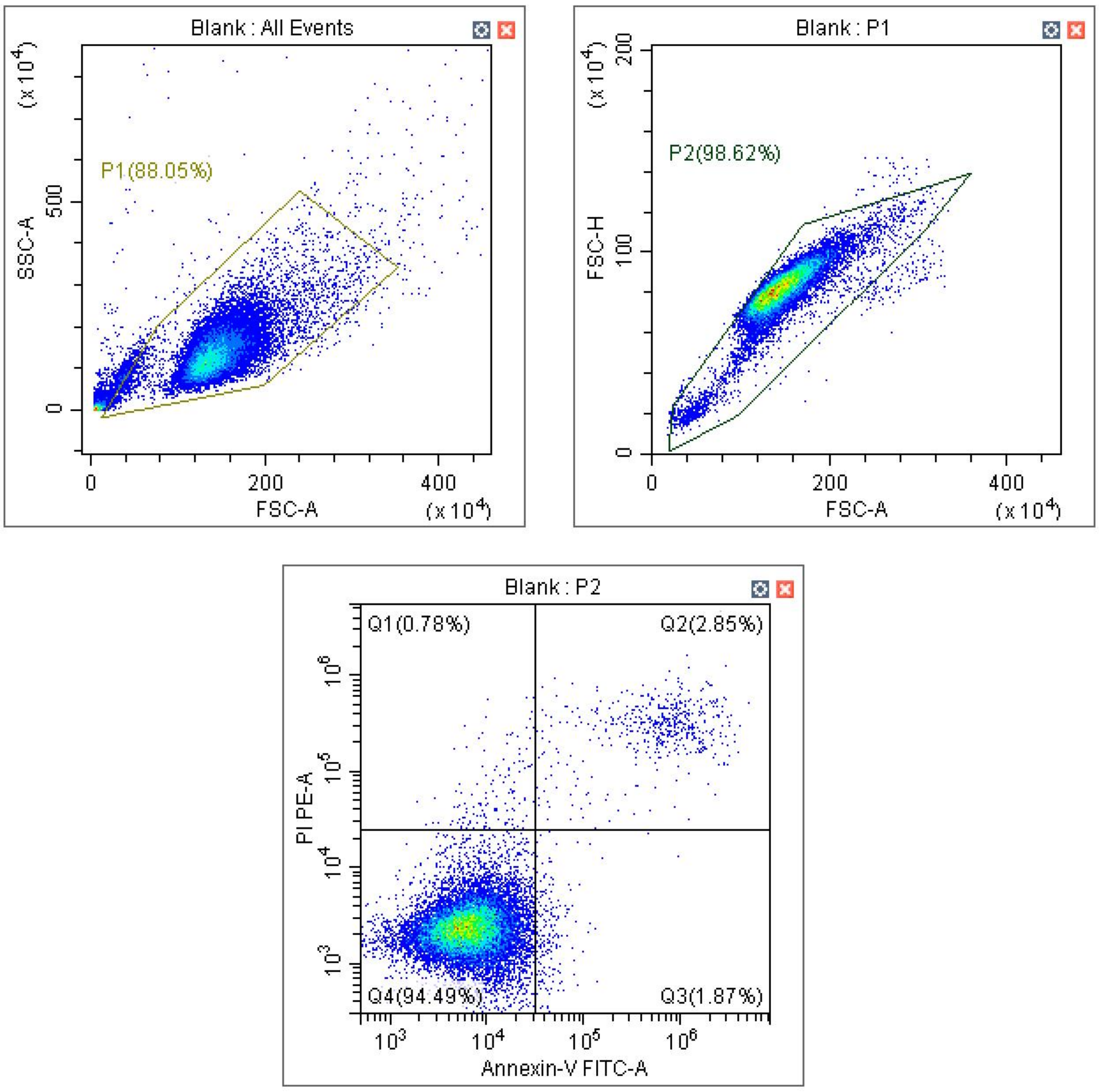

Supplement: Supplementary file 1 [file 41467_2026_76949_MOESM1_ESM.pdf]
