# Supplementary material for: Hybrid exosomal nanomotors convert pathological ROS into transport-driving cues to enhance cartilage accumulation in osteoarthritis
Source: Nat Commun. 2026 Aug 29;17:10310. doi: 10.1038/s41467-026-76949-8 (PMC13619634; doi:10.1038/s41467-026-76949-8)
Supplement: Supplementary file 2 [file 41467_2026_76949_MOESM2_ESM.pdf]

nature portfolio

Corresponding author(s): Huiqi Xie, Zhou Zhou, Bin Shen

Last updated by author(s): Jul 27, 2026

Reporting Summary

Nature Portfolio wishes to improve the reproducibility of the work that we publish. This form provides structure for consistency and transparency in reporting. For further information on Nature Portfolio policies, see our Editorial Policies and the Editorial Policy Checklist.

Statistics

For all statistical analyses, confirm that the following items are present in the figure legend, table legend, main text, or Methods section.

n/a	Confirmed	
☐	☒	The exact sample size (n) for each experimental group/condition, given as a discrete number and unit of measurement
☐	☒	A statement on whether measurements were taken from distinct samples or whether the same sample was measured repeatedly
☐	☒	The statistical test(s) used AND whether they are one- or two-sided<br>*Only common tests should be described solely by name; describe more complex techniques in the Methods section.*
☒	☐	A description of all covariates tested
☐	☒	A description of any assumptions or corrections, such as tests of normality and adjustment for multiple comparisons
☐	☒	A full description of the statistical parameters including central tendency (e.g. means) or other basic estimates (e.g. regression coefficient) AND variation (e.g. standard deviation) or associated estimates of uncertainty (e.g. confidence intervals)
☐	☒	For null hypothesis testing, the test statistic (e.g. F, t, r) with confidence intervals, effect sizes, degrees of freedom and P value noted<br>*Give P values as exact values whenever suitable.*
☒	☐	For Bayesian analysis, information on the choice of priors and Markov chain Monte Carlo settings
☐	☒	For hierarchical and complex designs, identification of the appropriate level for tests and full reporting of outcomes
☐	☒	Estimates of effect sizes (e.g. Cohen's d, Pearson's r), indicating how they were calculated

Our web collection on statistics for biologists contains articles on many of the points above.

Software and code

Policy information about availability of computer code

Data collection	Beckman Coulter Optima XPN-100 (Beckman Coulter); FEI Tecnai G2 Spirit (FEI Company); FEI Talos F200C (FEI Company); FEI Tecnai G2 F30 (FEI Company); Particle Metrix ZetaView PMX-230 (Particle Metrix); Zetasizer Nano ZS90 (Malvern Panalytical); Bioruptor Plus (Diagenode); D8 Advance (Bruker); L1600303 (PerkinElmer); Varioskan LUX (Thermo Fisher Scientific); CytoFLEX (Beckman Coulter); Lumina III (PerkinElmer); NexION 300D (PerkinElmer); K-Alpha (Thermo Fisher Scientific); LSM 980 (ZEISS); VS200 Slide Scanner (Olympus); Opera Phenix (PerkinElmer); Roche LightCycler 480 (Roche); SKYSCAN 1276 (Bruker); CatWalk XT (Noldus).
Data analysis	GraphPad Prism 8 (GraphPad Software); ImageJ v1.53 (NIH); CytExpert (Beckman Coulter); MestReNova v15.0 (Mestrelab Research); Tanon gel image analysis system (Tanon Technology); OlyVIA (Olympus); ZEISS ZEN software (ZEISS); Opera Phenix analysis software (PerkinElmer); CatWalk XT software (Noldus); ZetaView software (Particle Metrix); Zetasizer software (Malvern Panalytical).

For manuscripts utilizing custom algorithms or software that are central to the research but not yet described in published literature, software must be made available to editors and reviewers. We strongly encourage code deposition in a community repository (e.g. GitHub). See the Nature Portfolio guidelines for submitting code & software for further information.

Data

Policy information about availability of data

All manuscripts must include a data availability statement. This statement should provide the following information, where applicable:

- Accession codes, unique identifiers, or web links for publicly available datasets
- A description of any restrictions on data availability
- For clinical datasets or third party data, please ensure that the statement adheres to our policy

The transcriptome sequencing data generated in this study have been deposited in the Genome Sequence Archive (GSA) of the National Genomics Data Center (NGDC), China National Center for Bioinformation, under accession code CRA035408 (https://ngdc.cncb.ac.cn/gsa/browse/CRA035408). The data supporting the findings of this study are available within the Article, Supplementary Information, or Source Data file. The Source Data file contains the data underlying Figs. 2D-E, K-L; 3C, E, G, I, K; 4C-E, H, J; 5C, E, G, I, K; 6A, D, F, H; 7G-I; 8D-F, J-L; 9I; and Supplementary Figs. 11, 15, 16, 24, 26, 28–32, and 36.

Research involving human participants, their data, or biological material

Policy information about studies with human participants or human data. See also policy information about sex, gender (identity/presentation), and sexual orientation and race, ethnicity and racism.

Reporting on sex and gender	This study does not involve human participants.
Reporting on race, ethnicity, or other socially relevant groupings	This study does not involve human participants.
Population characteristics	This study does not involve human participants.
Recruitment	This study does not involve human participants.
Ethics oversight	This study does not involve human participants. All animal experimental procedures were approved by the Animal Ethics Committee of West China Hospital, Sichuan University (Approval No.: 20250923002) and were conducted in strict compliance with institutional guidelines for animal experimentation.

Note that full information on the approval of the study protocol must also be provided in the manuscript.

Field-specific reporting

Please select the one below that is the best fit for your research. If you are not sure, read the appropriate sections before making your selection.

☒ Life sciences ☐ Behavioural & social sciences ☐ Ecological, evolutionary & environmental sciences

For a reference copy of the document with all sections, see nature.com/documents/nr-reporting-summary-flat.pdf

Life sciences study design

All studies must disclose on these points even when the disclosure is negative.

Sample size	The sample size was selected to ensure sufficient statistical power while adhering to the 3Rs principle (Replacement, Reduction, and Refinement) for animal welfare. Unless otherwise stated, all experiments were performed using at least three independent biological replicates ($n \geq 3$), and exact sample sizes are provided in the figure legends.
Data exclusions	No data were excluded from the analyses.
Replication	All experiments were independently repeated at least three times ($n \geq 3$), and the detailed sample sizes are provided in the corresponding figure captions.
Randomization	Samples and animals were randomly grouped.
Blinding	The investigator performing data analysis was blinded to group allocation during the experiments.

Reporting for specific materials, systems and methods

We require information from authors about some types of materials, experimental systems and methods used in many studies. Here, indicate whether each material, system or method listed is relevant to your study. If you are not sure if a list item applies to your research, read the appropriate section before selecting a response.

Materials & experimental systems

n/a	Involved in the study	
☐	☒	Antibodies
☒	☐	Eukaryotic cell lines
☒	☐	Palaeontology and archaeology
☐	☒	Animals and other organisms
☒	☐	Clinical data
☒	☐	Dual use research of concern
☒	☐	Plants

Methods

n/a	Involved in the study	
☒	☐	ChIP-seq
☐	☒	Flow cytometry
☒	☐	MRI-based neuroimaging

Antibodies

Antibodies used	anti-CD90 Antibody (BioLegend, 206101; 1:100 for flow cytometry); anti-CD73 Antibody (BioLegend, 614951; 1:100 for flow cytometry); anti-CD34 Antibody (BioLegend, 378602; 1:100 for flow cytometry); anti-CD45 Antibody (BioLegend, 202201; 1:100 for flow cytometry); ALIX Rabbit mAb (ABclonal, A25326; 1:2000 for WB); TSG101 Rabbit mAb (ABclonal, A28611; 1:2000 for WB); CD81 Rabbit PolymAb (ABclonal, A22986PM; 1:1000 for WB); Calnexin Rabbit mAb (ABclonal, A27819; 1:5000 for WB); HRP-conjugated Goat anti-Rabbit IgG (H+L) (ABclonal, AS014; 1:5000 for WB); COL2A1 Rabbit mAb (ABclonal, A28346; 1:2000 for WB, 1:500 for IHC, 1:500 for IF); MMP13 Rabbit mAb (ABclonal, A11148; 1:1000 for WB, 1:200 for IHC, 1:200 for IF); iNOS Rabbit mAb (ABclonal, A3774; 1:1000 for WB, 1:100 for IHC, 1:100 for IF); GAPDH Rabbit mAb (High Dilution) (ABclonal, A19056; 1:50000 for WB); PRDX5 Rabbit mAb (ABclonal, A24731; 1:1000 for WB, 1:200 for IHC); GPX4 Rabbit mAb (ABclonal, A28048; 1:3000 for WB, 1:500 for IHC); NDUFA8 Rabbit pAb (ABclonal, A12118; 1:1000 for WB, 1:200 for IHC); COX IV Rabbit mAb (ABclonal, A28231; 1:3000 for WB, 1:500 for IHC); TRAF6 Rabbit mAb (ABclonal, A23385; 1:5000 for WB, 1:500 for IHC); Ikbkg Rabbit mAb (ABclonal, A28259; 1:3000 for WB, 1:200 for IHC); VDAC1 Rabbit mAb (ABclonal, A19707; 1:5000 for WB).
Validation	All antibodies were validated by the manufacturers for use in the indicated applications and species. Where necessary, antibody performance was further confirmed by titration or use of appropriate positive and negative controls prior to experiments.

Animals and other research organisms

Policy information about studies involving animals; ARRIVE guidelines recommended for reporting animal research, and Sex and Gender in Research

Laboratory animals	All in vivo experiments and primary chondrocyte isolations were performed using Sprague–Dawley rats.
Wild animals	No wild animals were used in this study.
Reporting on sex	Only male Sprague–Dawley rats were used in this study.
Field-collected samples	The study did not involve samples collected from the field.
Ethics oversight	All animal experimental procedures were approved by the Animal Ethics Committee of West China Hospital, Sichuan University (Approval No.: 20250923002) and conducted in strict compliance with the institutional guidelines for animal experimentation.

Note that full information on the approval of the study protocol must also be provided in the manuscript.

Plants

Seed stocks	This study does not involve plants.
Novel plant genotypes	This study does not involve plants.
Authentication	This study does not involve plants.

Flow Cytometry

Plots

Confirm that:

[x] The axis labels state the marker and fluorochrome used (e.g. CD4-FITC).

[x] The axis scales are clearly visible. Include numbers along axes only for bottom left plot of group (a 'group' is an analysis of identical markers).

[x] All plots are contour plots with outliers or pseudocolor plots.

[x] A numerical value for number of cells or percentage (with statistics) is provided.

Methodology

Sample preparation	Cells were collected, washed with PBS, stained with the indicated fluorescent probes or antibodies according to the experimental design, washed again, and resuspended in PBS for flow cytometric analysis.
Instrument	CytoFLEX (Beckman Coulter)
Software	CytExpert (Beckman Coulter)
Cell population abundance	Cell population abundance was quantified as the percentage of gated cells within the total cell population using flow cytometry data.
Gating strategy	Cells were first gated to exclude debris and dead cells based on forward scatter (FSC) and side scatter (SSC). Single cells were then identified using FSC-A versus FSC-H to exclude doublets. Unstained cells were used as negative controls to define gating thresholds for fluorescence signals.

[x] Tick this box to confirm that a figure exemplifying the gating strategy is provided in the Supplementary Information.
