# Supplementary material for: Hybrid exosomal nanomotors convert pathological ROS into transport-driving cues to enhance cartilage accumulation in osteoarthritis
Source: Nat Commun. 2026 Aug 29;17:10310. doi: 10.1038/s41467-026-76949-8 (PMC13619634; doi:10.1038/s41467-026-76949-8)
Supplement: Supplementary file 3 [file 41467_2026_76949_MOESM3_ESM.pdf]

nature portfolio

Peer Review File

Hybrid exosomal nanomotors convert pathological ROS into transport-driving cues to enhance cartilage accumulation in osteoarthritis

Corresponding Author: Professor Hui-Qi Xie

This file contains all reviewer reports in order by version, followed by all author rebuttals in order by version.

Version 0:

Reviewer comments:

Reviewer #1

(Remarks to the Author)
The authors report an interesting nanosystem incorporating miR-140 and MnO_2 nanoparticles, with surface modification by a cartilage-targeting peptide. The system is claimed to exhibit ROS-responsive self-propulsion behavior. The proposed concept is highly intriguing and is suggested to significantly enhance drug penetration depth compared with exosomes. Overall, this is an interesting study that may substantially advance therapeutic strategies for osteoarthritis (OA). However, several issues still need to be addressed.
1. The authors claim that the nanomotors generate propulsion by decomposing ROS to produce oxygen. My major concern is that ROS-mediated decomposition may lead to the disintegration of the nanoparticles themselves, potentially preventing effective delivery of the cargo into chondrocytes. The authors should carefully evaluate this possibility, as it may compromise the therapeutic efficacy.
2. In Fig. 3F–H, why does encapsulation of MnO_2 within nanovesicles enhance cellular uptake? What is the underlying mechanism responsible for this increased internalization?
3. In Fig. 3J–K, why do MnO_2-encapsulated nanoparticles exhibit slower degradation in vivo? According to the authors' design rationale, the nanoparticles are expected to undergo ROS-responsive cleavage and thus should degrade more rapidly. The authors should re-examine and rationalize these results.
4. The therapeutic role of microRNAs in OA, as well as the protective effects of MnO_2 against ROS-induced damage in chondrocytes, has been extensively reported in previous studies. The authors should discuss the novelty of the present work and further clarify the advantages of this combinational strategy in the Introduction to better highlight the innovation of the study.
5. In Fig. 9A, the PCA correlation analysis shows substantial intra-group variability in both the OA group and the nanomotor-treated group. Could this heterogeneity affect the accuracy or reliability of the results?
6. In Fig. 9G, the gene labels are too small to be clearly readable.
7. In Fig. 9J–K, did the authors analyze the expression levels of other components of the oxidative respiratory chain in addition to COX4?

Reviewer #2

(Remarks to the Author)
This work developed a membrane fusion-based cascade-chemotaxis hybrid exosome nanomotor system. By integrating manganese dioxide nanoparticles, cartilage-targeting peptide CAP, and therapeutic miR-140, the system achieved self-propulsion, deep cartilage penetration, and targeted drug delivery in the osteoarthritis microenvironment. Regarding the material construction and chemotaxis mechanism, solid experimental evidence is still lacking and requires in-depth consideration.
1. What is the chemotaxis mechanism of the nanomotor constructed in this study? Is it driven by released oxygen bubbles? Chemotaxis is a long-distance sensing process (gradual migration from low to high concentrations). If it is bubble-driven, how can movement occur under long-distance, low-concentration H_2O_2 conditions? This needs to be thoroughly investigated, as it is a crucial premise for the validity of this study.
2. The condition for chemotaxis is the presence of an H_2O_2 concentration gradient. The authors demonstrated the chemotactic penetration behavior of the nanomotor in ex vivo joints but should also provide evidence that an H_2O_2

concentration gradient exists in the ex vivo joint. Is this gradient sufficient to ensure chemotaxis?
3. Many reported chemotaxis systems are based on enzyme-substrate interactions due to the high affinity and specificity of enzymes for their substrates. The authors used the MnO_2 nanoparticle-H_2O_2 system. Does this system possess similar high affinity and specificity? Detailed experiments should be designed to verify this.
4. Material characterization needs to be strengthened. In particular, current evidence is insufficient to fully demonstrate that MnO_2 nanoparticles are encapsulated within the exosomes. TEM results are not clear, and more substantial evidence should be provided.
5. Under what conditions can exosomes release MnO_2 nanoparticles, and why? This point lacks sufficient experimental support. The process of release needs to be characterized in detail.
6. Chemotactic movement typically involves sensing the concentration gradient of an inducer over long distances. Therefore, the sensing components in nanomotors are generally exposed externally to sensitively detect inducer concentration gradients. In this work, MnO_2 nanoparticles are encapsulated inside the exosomes. How can chemotactic movement still occur under such conditions?
7. At least 20 particle trajectories should be provided to generate a motion velocity distribution plot.
8. Is the additional H_2O_2 added by the authors comparable to in vivo concentrations? This should be demonstrated.
9. The penetration capability of the nanomotor in vivo needs to be evaluated in detail (e.g., penetration depth, co-localization with chondrocytes, specific

Reviewer #3

(Remarks to the Author)
This research reports an innovative cascade chemotactic hybrid exosomal nanomotor (CAP-Mn/140@hyExos) for precision treatment of osteoarthritis (OA). Through integrated design, this work combines the catalytic self-propulsion capability of MnO_2 NPs, the precise recognition ability of CAP, and engineered exosomes overexpressing miR-140. This aims to synergistically address key bottlenecks in OA treatment, including short intra-articular drug retention time, difficulty in penetrating the cartilage matrix, and low chondrocyte targeting efficiency. The research forms a complete closed-loop, spanning the construction and characterisation of the nanocarrier to in vitro and in vivo functional validation and mechanism exploration. This work demonstrates considerable innovation and holds potential for clinical translation.

Major concerns:
1. Membrane fusion employed a sequential 'incubation-extrusion' method. FRET efficiency data for the final product were not presented. Clearly state in the methods or results that following sequential processing, the size distribution (PDI) of the final product.
2. In Figures 2F and 2G, supplementary detection of calnexin or GRP94 as negative markers should be performed to rule out endoplasmic reticulum contamination.
3. Section 5.15 of Materials and Methods mentions the use of the 'ACLT+DMM' surgical model, yet Section 5.10, 'Assessment of Joint Stiffness Capacity', employs a 'monoiodoacetate' injection model. These represent distinct OA models (post-traumatic OA vs chemically induced OA). Why were different models utilised across these experiments?
4. In vivo therapeutic experiments, specify the number of exosomal nanomotors or protein mass administered per injection.
5. The degradation and excretion pathways of MnO_2 within the joint cavity and systemic circulation were not discussed. Add long-term (>8 weeks) detection of Mn accumulation in joints and brain tissue.
6. Beyond immunohistochemical validation of Cox4i1, perform protein-level validation for 1–2 core genes within other key pathways identified by GSEA enrichment analysis (e.g., immune regulation-related pathways).
7. miRNAs typically suppress target gene expression by inhibiting RNA post- transcriptional translation. However, the key downstream gene Cox4i1 exhibited upregulation following miR-140 delivery. What might be the regulatory mechanism governing the interaction between miR-140 and Cox4i1?

Minor issues:
1. Throughout the text, the statistical symbol P should be capitalised and italicised.
2. Line 175: (i) Initially, MnO_2 nanoparticles were synthesised via biomineralisation using bovine serum albumin (BSA)-modified manganese chloride. Manganese chloride is not shown in Figure 2A.
3. Figures 3, 7, and 8 require specification of biological replicates (n values).
4. 'Disccussion' should be corrected to 'Discussion'.

Reviewer #4

(Remarks to the Author)
I co-reviewed this manuscript with one of the reviewers who provided the listed reports. This is part of the Nature Communications initiative to facilitate training in peer review and to provide appropriate recognition for Early Career Researchers who co-review manuscripts.

Version 1:

Reviewer comments:

Reviewer #1

(Remarks to the Author)

The authors has addressed all my concerns, I have no further comments.

Reviewer #2

(Remarks to the Author)
I have carefully read the authors' revisions in response to the reviewers' comments, particularly regarding material degradation/fate and in vivo ROS measurements. I would first like to acknowledge the considerable efforts made by the authors. However, several key issues have not been fully resolved, and I suggest that the authors address them in future investigations.

The authors used DiD fluorescence labeling to track nanoparticle organ distribution and clearance, observing that fluorescence signals nearly disappeared after 8 weeks, leading to the conclusion of "complete systemic clearance". However, attenuation of fluorescence signal does not directly equate to the metabolism or excretion of the material itself. DiD is a lipophilic dye that may undergo:Fluorescence quenching, Detachment from the carrier and transfer to other lipid structures, Dilution of the dye within tissues after carrier disintegration, even if the material remains. Therefore, more rigorous methods should be employed to investigate this issue, as it is crucial for assessing translational potential.
2. The authors used 100 μM H_2O_2 to establish an oxidative stress model in vitro and demonstrated elevated ROS signals in OA cartilage (Fig. S20). However, they did not actually measure the concentration of H_2O_2 or other ROS species in the joint fluid or cartilage of the animal model. This makes it impossible for readers to judge whether the 100 μM H_2O_2 used in vitro matches the actual in vivo oxidative levels, or whether the nanomotors operate within a "ROS-responsive" window in vivo. I recommend measuring H_2O_2 concentration in OA joint fluid (e.g., using Amplex Red or a chemiluminescence assay), or detecting hydroxyl radicals by microdialysis coupled with liquid chromatography. At the very least, provide in vivo reference values comparable to the in vitro concentration (e.g., reported ROS concentration ranges in similar OA models). Qualitative descriptions such as "elevated and heterogeneous" are insufficient.
3.The authors showed increased penetration depth (~140 μm) and DAPI costaining indicating signals near cell nuclei. However, it remains unclear whether the signals in deep cartilage layers are truly inside chondrocytes or simply retained in the pericellular matrix. The resolution of confocal microscopy is insufficient to distinguish intra versus extracellular localization. I suggest using 3D reconstructed confocal imaging or immunogold electron microscopy to localize nanoparticles relative to the plasma membrane/cytoplasm, or performing flow cytometry on enzymatically digested deeplayer chondrocytes to quantify fluorescence positivity.
4.The authors propose that "ROSresponsive motion" and "CAPmediated matrix binding" collectively enhance distribution, but no experiment was designed to decouple these two contributions. For example, in the absence of CAP modification (i.e., Mn@hyExos without CAP), would the enhanced motion alone be sufficient to achieve deep penetration under ROS conditions? Current data only compare CAPMn@hyExos with CAPmExos (lacking MnO_2). A comparison between CAPMn@hyExos and nontargeted Mn@hyExos (without CAP) is missing.

Reviewer #3

(Remarks to the Author)
The authors have addressed all of my questions.

Reviewer #4

(Remarks to the Author)
I co-reviewed this manuscript with one of the reviewers who provided the listed reports. This is part of the Nature Communications initiative to facilitate training in peer review and to provide appropriate recognition for Early Career Researchers who co-review manuscripts.

Version 2:

Reviewer comments:

Reviewer #2

(Remarks to the Author)
The authors have addressed the remaining concerns through additional experiments and data clarification. I recommend acceptance of this version for publication.

Open Access This Peer Review File is licensed under a Creative Commons Attribution 4.0 International License, which permits use, sharing, adaptation, distribution and reproduction in any medium or format, as long as you give appropriate credit to the original author(s) and the source, provide a link to the Creative Commons license, and indicate if changes were made.
In cases where reviewers are anonymous, credit should be given to 'Anonymous Referee' and the source.
The images or other third party material in this Peer Review File are included in the article's Creative Commons license, unless indicated otherwise in a credit line to the material. If material is not included in the article's Creative Commons license and your intended use is not permitted by statutory regulation or exceeds the permitted use, you will need to obtain permission directly from the copyright holder.
To view a copy of this license, visit https://creativecommons.org/licenses/by/4.0/

Point-by-point Response to Reviewers' Comments

We sincerely thank the editor and the reviewers for their careful evaluation of our manuscript and for the constructive comments.

We have carefully revised the manuscript in response to all comments. In particular, we have:

● revised the terminology describing the transport behavior to avoid potential ambiguity associated with "chemotaxis", and accordingly updated the manuscript title to more accurately reflect the ROS-responsive transport mechanism;

● substantially strengthened the mechanistic interpretation through additional experimental evidence, including microfluidic analyses, ROS imaging in osteoarthritic cartilage, and expanded ex vivo and in vivo cartilage penetration studies;

● reinforced the structural characterization and stability of the hybrid exosomal system using additional cryo-EM, elemental mapping, and microenvironment-dependent stability analyses;

● expanded the in vivo evaluation to include comprehensive biodistribution profiling, clearance kinetics, and long-term biosafety assessment up to 8 weeks, supported by histological and ICP-OES analyses;

● provided additional multi-level validation of the relationship between miR-140 and Cox4i1, including transcriptomic, protein-level, Western blot, and qPCR analyses.

All changes are clearly marked in the revised manuscript using track changes/highlighted text, and a detailed point-by-point response to each reviewer comment is provided below.

Collectively, these revisions have substantially improved the clarity, rigor, and mechanistic robustness of the manuscript. We hope that the revised version is now suitable for further consideration in ***Nature Communications***.

Reviewer #1 (Remarks to the Author):

The authors report an interesting nanosystem incorporating miR-140 and MnO_2 nanoparticles, with surface modification by a cartilage-targeting peptide. The system is claimed to exhibit ROS-responsive self-propulsion behavior. The proposed concept is highly intriguing and is suggested to significantly enhance drug penetration depth compared with exosomes. Overall, this is an interesting study that may substantially advance therapeutic strategies for osteoarthritis (OA). However, several issues still need to be addressed.

Response:

We sincerely thank the reviewer for the positive and constructive evaluation of our manuscript. We greatly appreciate the recognition of the novelty of our nanosystem integrating miR-140, MnO_2 nanoparticles, and a cartilage-targeting peptide, as well as the potential of this strategy for enhancing intra-cartilage drug delivery in osteoarthritis.

We also sincerely appreciate the reviewer's insightful and critical comments, which have helped us further improve the clarity and mechanistic interpretation of the study. In response, we have carefully revised the manuscript and performed additional analyses and clarifications to address all concerns in a point-by-point manner, as detailed below.

1. The authors claim that the nanomotors generate propulsion by decomposing ROS to produce oxygen. My major concern is that ROS-mediated decomposition may lead to the disintegration of the nanoparticles themselves, potentially preventing effective delivery of the cargo into chondrocytes. The authors should carefully evaluate this possibility, as it may compromise the therapeutic efficacy.

Response:

We sincerely thank the reviewer for this important concern regarding the potential structural instability of the nanomotors under ROS-enriched conditions and its possible impact on cargo delivery efficiency. We agree that excessive ROS-triggered decomposition could compromise carrier integrity and reduce therapeutic efficacy. Therefore, in the revised manuscript, we systematically evaluated the structural stability and cargo retention behavior of the nanomotor system under physiological, OA-relevant, and strongly oxidative conditions.

From a mechanistic perspective, MnO_2 exhibits environment-dependent redox activity. Under physiological and OA-relevant conditions (neutral to mildly acidic pH with moderate ROS levels), MnO_2 primarily functions as a catalytic component that reacts with H_2O_2 ($2H_2O_2 \rightarrow 2H_2O + O_2\uparrow$) while remaining sufficiently stable within the exosomal platform. In contrast, under strongly oxidative and acidic conditions, MnO_2 undergoes accelerated degradation and structural destabilization ($MnO_2 + H_2O_2 + 2H^+ \rightarrow Mn^{2+} + 2H_2O + O_2\uparrow$). Importantly, such extreme conditions are not representative of the OA joint microenvironment investigated in this study.

Experimentally, we first evaluated the colloidal stability of the nanomotor system in simulated synovial fluid. After 7 days of incubation, all exosomal formulations maintained stable hydrodynamic diameters (~110–130 nm) with low polydispersity indices (PDI: 0.10–0.25), without obvious aggregation or structural collapse, indicating favorable baseline stability under joint-relevant conditions (**Fig. S15**).

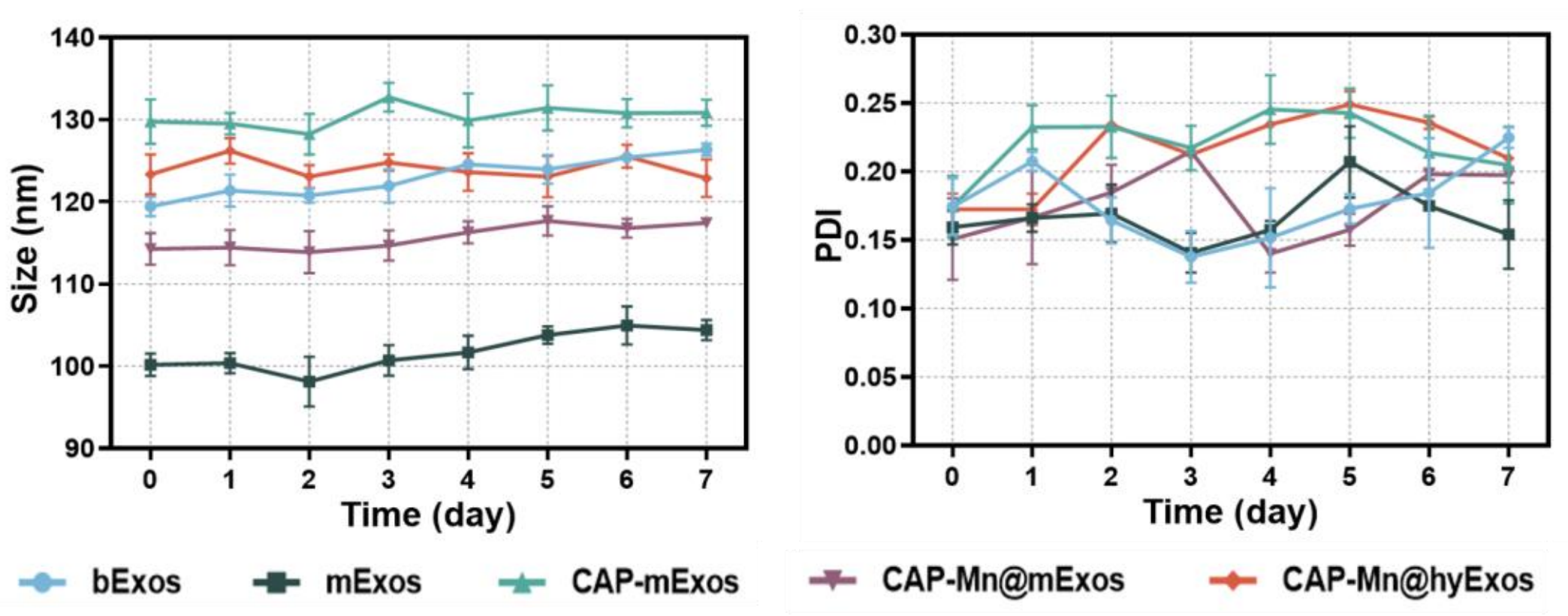

Fig. S15 | DLS analysis of hydrodynamic size and PDI of exosomal formulations in simulated synovial fluid over 7 days (n = 3). Data are presented as the mean ± SD. Source data are provided as a Source Data file.

To further assess cargo retention behavior, fluorescence release experiments were performed under different oxidative conditions. Only minimal fluorescence leakage was observed in simulated synovial fluid over 7 days. Under OA-relevant conditions (100 μM H_2O_2 or pH 6.8 + 100 μM H_2O_2), a slight increase in fluorescence signal was detected, while overall release remained low and gradual, indicating that the nanomotor system largely retained its structural integrity and cargo confinement capability. In contrast, under strongly oxidative conditions (≥1 mM H_2O_2), fluorescence intensity increased markedly, indicating substantial structural destabilization and accelerated release behavior (**Fig. 2K**).

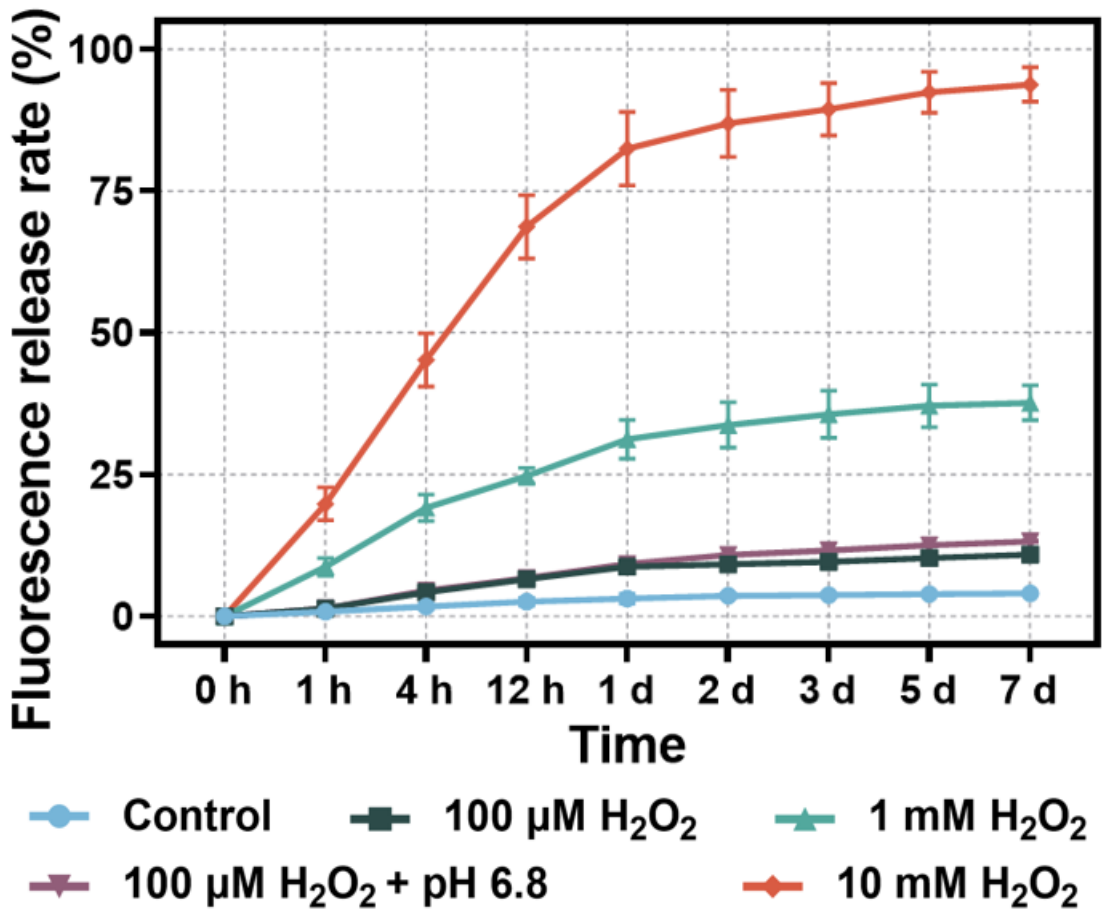

Fig. 2K | Fluorescence release of DiI-labeled CAP-Mn@hyExos over 7 days under OA-relevant microenvironmental conditions (n = 3). Data are presented as the mean ± SD. Source data are provided as a Source Data file.

Consistent with these observations, DLS analysis showed that particle size remained relatively stable under physiological and OA-like oxidative conditions (~120–140 nm, PDI < 0.30), while pronounced size enlargement and increased PDI were observed only under strongly oxidative conditions (**Figs. 2L & S16**), further supporting condition-dependent structural destabilization.

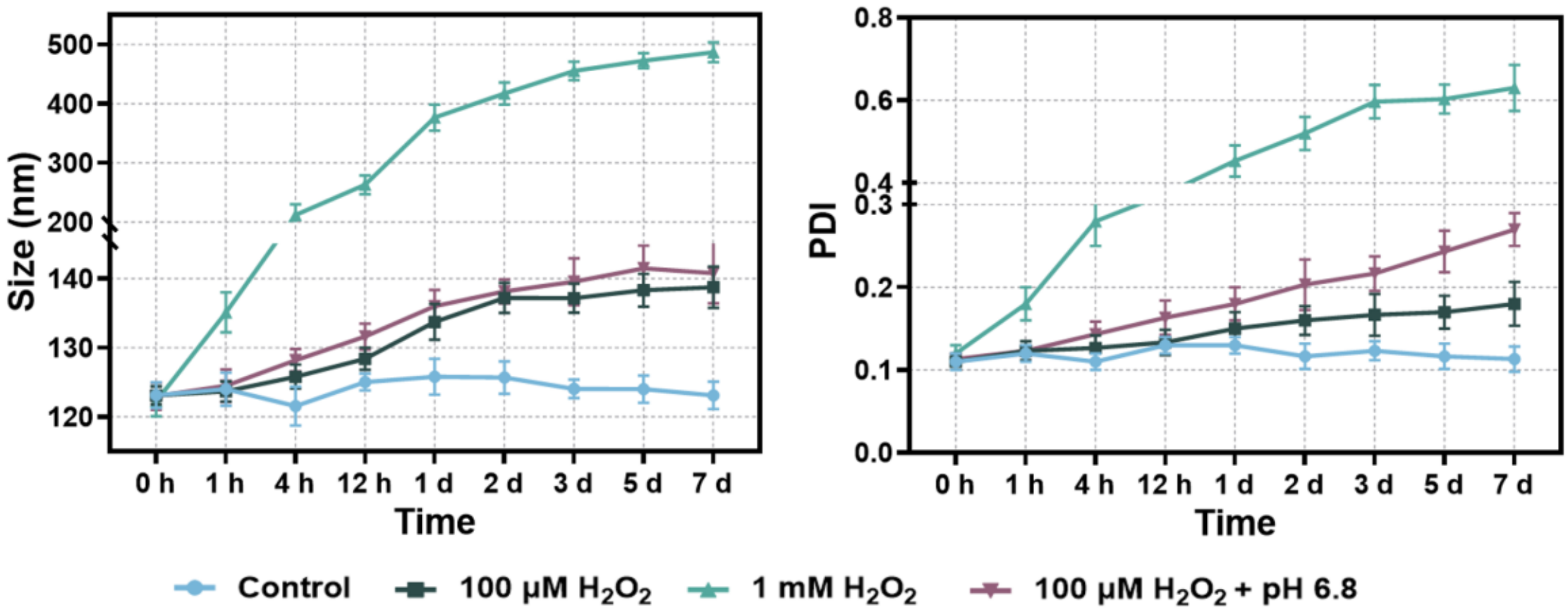

Figs. 2L & S16 | DLS analysis of size and PDI of CAP-Mn@hyExos over 7 days under OA-relevant microenvironmental conditions (n = 3). Data are presented as the mean ± SD. Source data are provided as a Source Data file.

Collectively, these results demonstrate that the nanomotor system remains sufficiently stable under physiological and OA-relevant oxidative conditions to preserve cargo retention and delivery capability, while significant structural disruption occurs only under non-physiological oxidative

extremes. These findings support that ROS-responsive transport behavior can be achieved without substantially compromising nanocarrier stability under therapeutically relevant conditions.

The above revisions and newly added data have been incorporated into the revised manuscript; please refer to lines 205–221 (page 6) and lines 741–755 (page 18) for detailed information.

2. In Fig. 3F–H, why does encapsulation of MnO_2 within nanovesicles enhance cellular uptake? What is the underlying mechanism responsible for this increased internalization?

Response:

We sincerely thank the reviewer for this important question regarding the mechanism underlying the enhanced cellular uptake observed after MnO_2 incorporation. We agree that the increased internalization behavior warrants careful mechanistic interpretation. Although the precise molecular basis remains to be further investigated, our current results suggest that the enhanced uptake is closely associated with the ROS-responsive transport behavior of the MnO_2-containing nanoplatform under osteoarthritic conditions.

Importantly, all cellular uptake experiments were performed in OA-like chondrocyte models characterized by elevated ROS levels. Under these conditions, MnO_2-containing nanoplatforms exhibited enhanced dynamic transport behavior compared with exosomal groups lacking MnO_2. We speculate that this ROS-responsive motility enhancement increases the frequency and duration of nanoplatform–cell interactions within the pericellular microenvironment, thereby facilitating subsequent endocytic internalization by chondrocytes.

Notably, the observed enhancement appears to arise from microenvironment-responsive transport behavior rather than from specific receptor-mediated targeting interactions. In this context, ROS-associated modulation of particle dynamics may promote more effective contact between nanoplatforms and chondrocytes under oxidative microenvironmental conditions.

In addition, the biological origin of the hybrid exosomal membrane may provide an auxiliary contribution to cellular internalization. Specifically, compared with mExos, bExos exhibited relatively higher uptake efficiency in chondrocytes, suggesting that membrane components derived from chondrocyte-related biological sources may facilitate membrane recognition and endocytic interactions to some extent. However, because this tendency was observed across both

MnO_2-containing and MnO_2-free groups, it is unlikely to solely account for the pronounced uptake enhancement induced by MnO_2 incorporation.

Collectively, these findings support that MnO_2 incorporation enhances cellular internalization primarily through ROS-responsive modulation of transport behavior, thereby increasing effective nanoplatform–cell interactions under osteoarthritic microenvironmental conditions.

The above revisions have been incorporated into the revised manuscript; please refer to lines 272-283 (pages 7-8) for detailed information.

3. In Fig. 3J–K, why do MnO_2-encapsulated nanoparticles exhibit slower degradation in vivo? According to the authors' design rationale, the nanoparticles are expected to undergo ROS-responsive cleavage and thus should degrade more rapidly. The authors should re-examine and rationalize these results.

Response：

We sincerely thank the reviewer for this insightful question regarding the apparently slower in vivo signal decay observed for MnO_2-loaded nanoparticles in Fig. 3J–K. We agree that ROS-responsive systems are generally expected to exhibit accelerated degradation under oxidative conditions, and we appreciate the opportunity to clarify this point.

Importantly, the fluorescence signal shown in Fig. 3J–K should not be interpreted as a direct measurement of intrinsic nanoparticle degradation kinetics. Instead, the observed signal persistence reflects the combined effects of tissue distribution, cartilage retention, and clearance behavior in vivo.

As demonstrated by our stability and release studies, MnO_2-containing nanoplatforms remain structurally stable under physiological and OA-relevant oxidative conditions, while significant structural breakdown occurs only under supraphysiological oxidative stress. This indicates that, under therapeutic conditions, MnO_2 primarily serves as a catalytic ROS-responsive component rather than being rapidly consumed.

In vivo, incorporation of MnO_2 enhances ROS-responsive transport behavior, while CAP modification promotes strong affinity to cartilage extracellular matrix. These two effects synergistically facilitate deep cartilage accumulation and prolonged intratissue retention. At the same time, reduced exposure to synovial fluid-mediated convection and lymphatic drainage

further contributes to slower apparent signal decay.

In addition, MnO_2-mediated modulation of the local oxidative microenvironment may also partially contribute to prolonged retention by attenuating inflammation-driven clearance within the joint cavity; however, this effect is considered secondary compared with the dominant contributions of enhanced cartilage accumulation and reduced clearance pathways.

Collectively, the slower in vivo signal decay arises from altered tissue distribution and clearance behavior rather than changes in intrinsic ROS responsiveness or material degradation, and is therefore consistent with the proposed design principle.

The above revisions have been incorporated into the revised manuscript; please refer to lines 284–294 (page 8) for detailed information.

4. The therapeutic role of microRNAs in OA, as well as the protective effects of MnO_2 against ROS-induced damage in chondrocytes, has been extensively reported in previous studies. The authors should discuss the novelty of the present work and further clarify the advantages of this combinational strategy in the Introduction to better highlight the innovation of the study.

Response：

We sincerely thank the reviewer for this constructive comment regarding the established therapeutic roles of miR-140 and MnO_2 in osteoarthritis. This suggestion has been valuable for further clarifying the conceptual positioning and innovation of the present study in the revised Introduction.

As correctly noted, both miR-140 and MnO_2 have been widely investigated in OA therapy, particularly for their roles in regulating chondrocyte gene expression and alleviating oxidative stress. Therefore, the objective of this work is not to re-establish the individual functions of these components or to present a simple additive combination.

Instead, the main novelty of this study lies in integrating pathological ROS into the design of the delivery system, where it regulates intratissue transport behavior within the cartilage microenvironment. In this context, ROS is leveraged as an endogenous microenvironmental factor that modulates nanoparticle motility and spatial distribution under tissue confinement conditions.

Within this framework, the system components are functionally coordinated rather than

independently acting. MnO_2 serves as a ROS-responsive element that links local oxidative conditions to changes in nanosystem transport behavior; the cartilage-targeting peptide (CAP) promotes matrix interaction and retention within the dense extracellular matrix; and miR-140 functions as the therapeutic cargo following enhanced tissue accumulation and cellular uptake.

Importantly, the innovation of this work is therefore centered on ROS-responsive regulation of intratissue transport behavior, where modulation of spatial distribution within the cartilage microenvironment contributes to improved delivery efficiency. This transport-centric perspective distinguishes the present study from conventional miRNA delivery systems and MnO_2-based antioxidant strategies that primarily focus on cargo function or reactive oxygen species scavenging alone.

Accordingly, the Introduction has been revised to better highlight this transport-centered design rationale and to clearly distinguish the present study from previous related approaches.

The above revisions have been incorporated into the revised manuscript; please refer to lines 65–119 (pages 3–4) for detailed information.

5. In Fig. 9A, the PCA correlation analysis shows substantial intra-group variability in both the OA group and the nanomotor-treated group. Could this heterogeneity affect the accuracy or reliability of the results?

Response:

We sincerely thank the reviewer for this important question regarding the intra-group variability observed in the PCA analysis shown in Fig. 9A.

First, the observed intra-group dispersion does not reflect experimental instability or reduced data quality. Instead, it primarily reflects the intrinsic biological heterogeneity of the osteoarthritis (OA) model. Given the progressive and spatially heterogeneous nature of OA, individual differences in cartilage degeneration severity, inflammatory status, and chondrocyte metabolic activity are expected, and can naturally contribute to transcriptomic variability within each group.

Importantly, this within-group heterogeneity does not affect the interpretation of the overall dataset structure. As shown in Fig. 9A (Fig. S35 in the revised manuscript), PC1 accounts for approximately 70% of the total variance, whereas PC2 explains only around 10%, indicating that the dominant source of variation is associated with treatment effects rather than intra-group

differences. This suggests that the primary separation in the transcriptomic landscape is driven by the experimental intervention.

Despite the presence of biological variability within each group, the OA and nanomotor-treated samples remain clearly separated in the principal component space, indicating a consistent and coordinated treatment-associated transcriptional response across biological replicates. Furthermore, the transcriptomic trends are strongly supported by independent validation at multiple levels, including histological staining, immunohistochemistry, and functional assays, all of which show consistent treatment-related changes. This multi-layer agreement further supports the robustness of the observed transcriptomic patterns.

Taken together, the intra-group variability observed in Fig. 9A reflects the inherent biological complexity of OA rather than technical inconsistency, and does not alter the conclusion that nanomotor treatment induces a dominant and coordinated transcriptional reprogramming at the system level.

6. In Fig. 9G, the gene labels are too small to be clearly readable.

Response:

We sincerely thank the reviewer for the careful comment regarding the readability of gene labels in **Fig. 9G (Fig. 9E in the revised manuscript)**.

In response, we have revised the figure to improve visual clarity. Specifically, the font size of gene labels has been increased, and the heatmap layout has been optimized to enhance overall readability while preserving the integrity of the dataset.

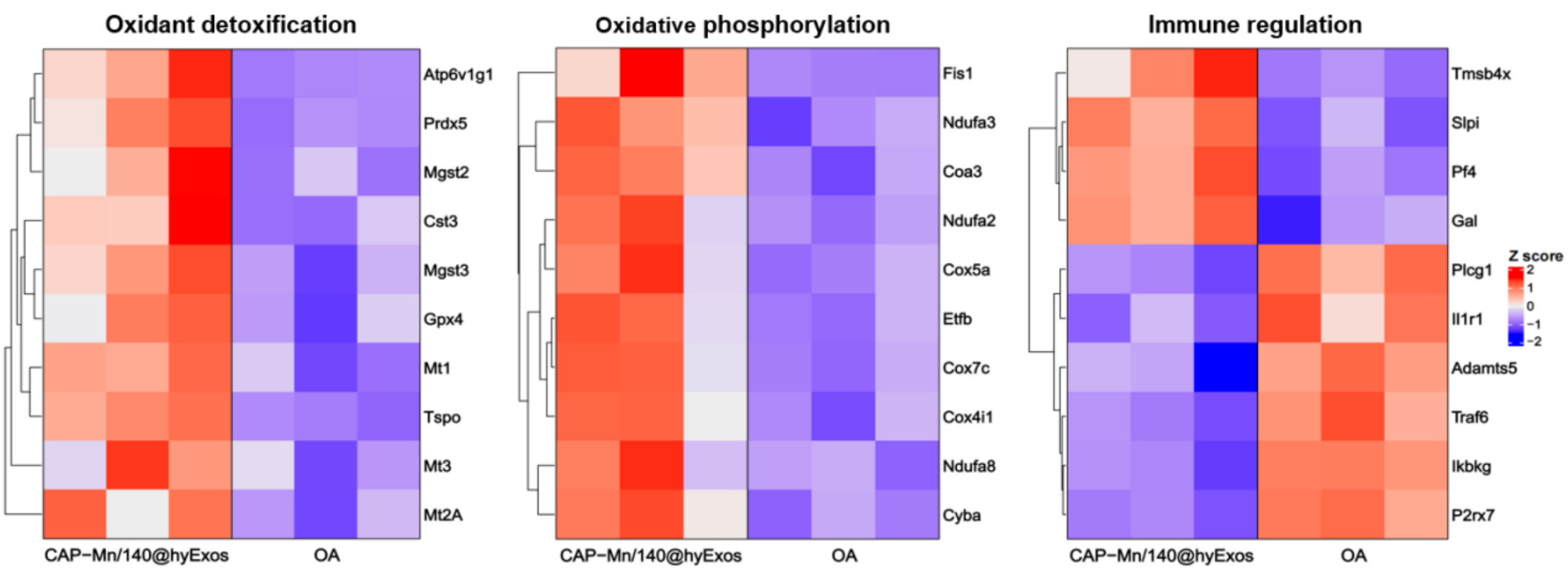

Fig. 9E | Heatmap of selected DEGs involved in pathways related to oxidative detoxification, oxidative phosphorylation, and immune modulation (n = 3).

The revised Fig. 9E (formerly Fig. 9G) now provides clearly legible gene annotations and improved visualization quality suitable for publication.

7. In Fig. 9J–K, did the authors analyze the expression levels of other components of the oxidative respiratory chain in addition to COX4?

Response:

We sincerely thank the reviewer for this important question regarding whether additional components of the oxidative respiratory chain were analyzed beyond COX4.

In response, we would like to clarify that the oxidative phosphorylation-related analysis in this study was not limited to Cox4i1 alone. In addition to Complex IV (Cox4i1), we also examined Ndufa8, a representative component of Complex I in the electron transport chain. The coordinated upregulation of these genes suggests a system-level enhancement of mitochondrial oxidative phosphorylation activity, rather than isolated regulation of a single respiratory complex.

Beyond oxidative phosphorylation, we further evaluated representative genes associated with related functional modules. Specifically, Prdx5 and Gpx4 were upregulated, indicating enhanced oxidative detoxification capacity, while Traf6 and Ikbkg were downregulated, suggesting suppression of inflammatory signaling (**Fig. S37**).

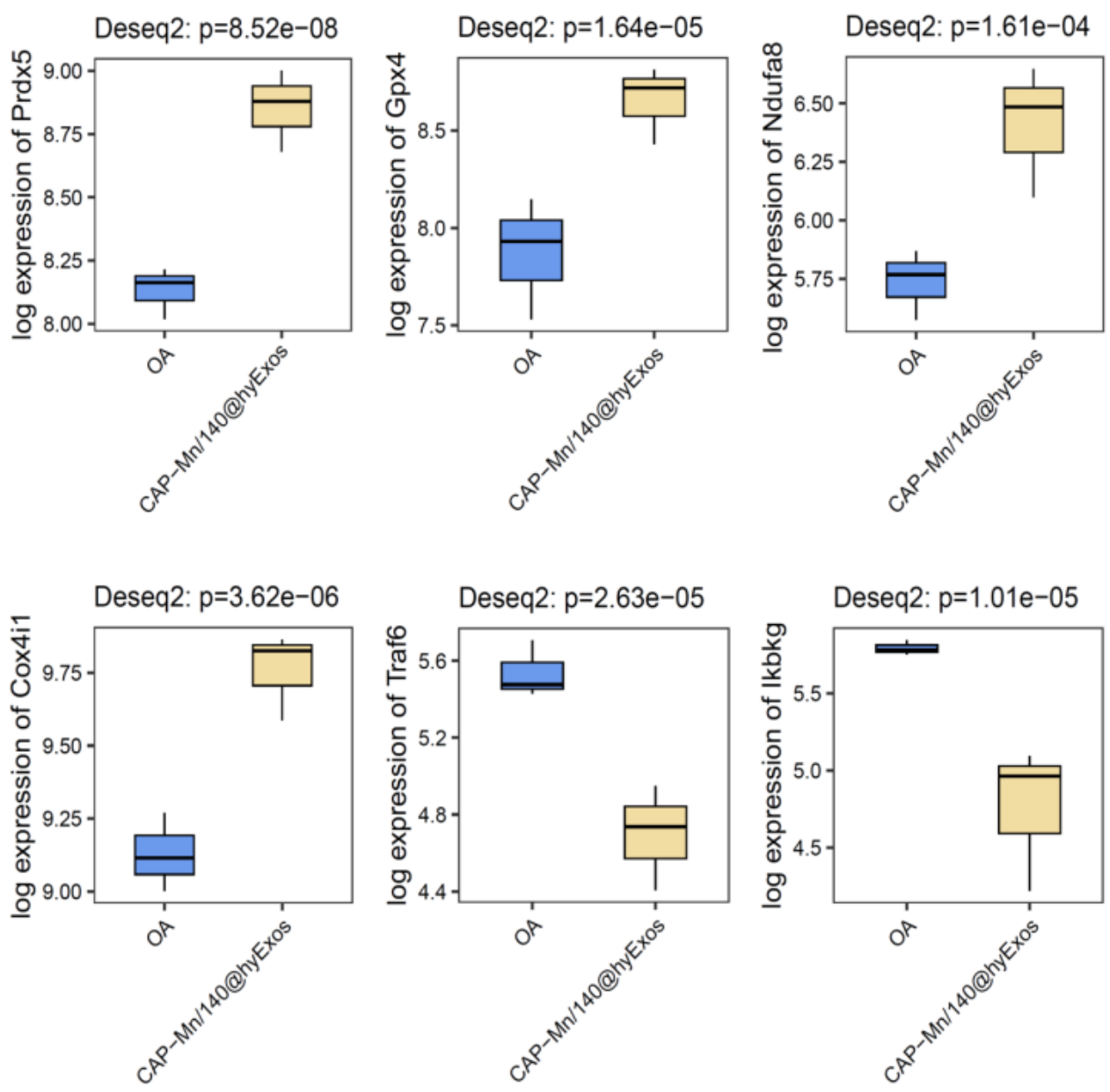

Fig. S37 | Expression levels of key genes involved in oxidative detoxification, mitochondrial oxidative phosphorylation, and anti-inflammatory immune regulation across OA control and CAP-Mn/140@hyExos-treated groups (n = 3).

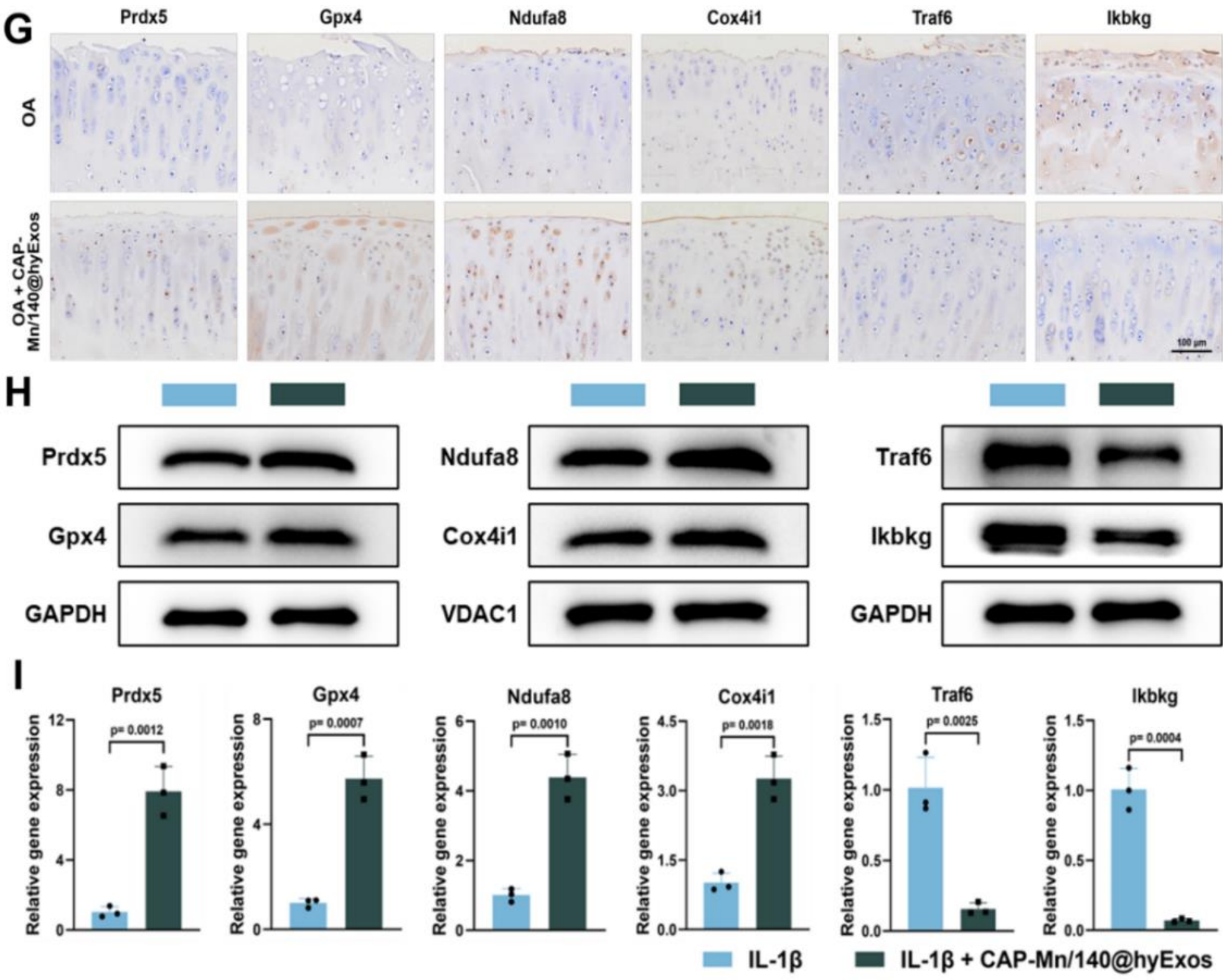

Figs. 9G-I | **G** Immunohistochemical analysis of proteins encoded by representative DEGs from major pathways in OA cartilage following CAP-Mn/140@hyExos treatment, scale bar = 100 μm. **H** Western blot analysis of the same proteins in chondrocyte models. **I** qPCR validation of the same genes in chondrocyte models (n = 3). Data are presented as mean ± SD. p values are from unpaired two-tailed Student's t-test (**I**). Data in (**G, H**) are representative of three independent experiments with similar results. Source data are provided as a Source Data file.

Importantly, these transcriptomic findings were consistently validated at multiple experimental levels, including immunohistochemistry in cartilage tissue and Western blotting and qPCR in chondrocyte models (**Figs. 9G–I**). These complementary results support coordinated regulation of mitochondrial function, redox homeostasis, and inflammatory signaling following CAP-Mn/140@hyExos treatment.

Taken together, these data indicate that the observed effects reflect coordinated modulation of multiple components of the oxidative phosphorylation pathway and associated cellular functional modules, rather than regulation of Cox4i1 alone.

The above revisions have been incorporated into the revised manuscript; please refer to lines 507–521 (page 13) and lines 960–964 (page 23) for detailed information.

Reviewer #2 (Remarks to the Author):

This work developed a membrane fusion-based cascade-chemotaxis hybrid exosome nanomotor system. By integrating manganese dioxide nanoparticles, cartilage-targeting peptide CAP, and therapeutic miR-140, the system achieved self-propulsion, deep cartilage penetration, and targeted drug delivery in the osteoarthritis microenvironment. Regarding the material construction and chemotaxis mechanism, solid experimental evidence is still lacking and requires in-depth consideration.

Response:

We sincerely thank the reviewer for the overall evaluation and constructive comments. We fully appreciate the concerns regarding both the chemotaxis-related interpretation and the material construction of the system.

Regarding the conceptual description of the system and the chemotaxis-related interpretation, we recognize that the original terminology "chemotaxis" may not fully capture the complexity of the transport behavior observed in this system and could potentially be misinterpreted in the context of classical chemotaxis. Importantly, the experimental observations do demonstrate a chemotaxis-like spatial redistribution behavior at the tissue scale; however, this behavior does not appear to rely on classical receptor-mediated long-range gradient sensing or active directional chemotactic signaling.

Based on additional mechanistic analyses and newly added microfluidic experiments, we now interpret the observed behavior more cautiously as a ROS-responsive transport process, in which pathological ROS modulates nanoparticle motility and contributes to biased spatial redistribution under cartilage matrix confinement conditions. Corresponding revisions have been made throughout the manuscript.

Regarding the material construction and structural characterization, we have strengthened the supporting evidence with additional experimental validation to further confirm the assembly and structural integrity of the exosome-based nanoplatform.

We trust that these revisions adequately address the reviewer's concerns and substantially improve the conceptual clarity and robustness of the manuscript.

1. What is the chemotaxis mechanism of the nanomotor constructed in this study? Is it driven by released oxygen bubbles? Chemotaxis is a long-distance sensing process (gradual migration from low to high concentrations). If it is bubble-driven, how can movement occur under long-distance, low-concentration H_2O_2 conditions? This needs to be thoroughly investigated, as it is a crucial premise for the validity of this study.

Response：

We sincerely thank the reviewer for this insightful and fundamental question regarding the transport mechanism of the CAP-Mn/140@hyExos nanomotor system. This comment is highly valuable for clarifying the physical basis underlying its intratissue redistribution behavior.

We would like to first clarify that the observed behavior exhibits a chemotaxis-like spatial redistribution pattern at the tissue scale, as demonstrated by enhanced accumulation of MnO_2-containing nanoplatforms in ROS-enriched regions in osteoarthritic microenvironments. This indicates a ROS-associated spatial bias in particle distribution. However, this behavior does not fully correspond to classical chemotaxis, which typically involves receptor-mediated long-range gradient sensing and active directional migration [1,2].

Instead, based on our mechanistic analyses and microfluidic experiments, we more cautiously interpret the observed phenomenon as a ROS-responsive transport process, in which spatially heterogeneous ROS conditions modulate the local motion behavior of nanoplatforms. This leads, over time, to enhanced accumulation in ROS-enriched regions under tissue microenvironmental conditions, as directly supported by Y-shaped microfluidic assays (Figs. S18), where MnO_2-containing groups show asymmetric accumulation only in ROS-containing channels, while control exosomes remain symmetrically distributed.

Regarding the propulsion-related mechanism, MnO_2 catalyzes the decomposition of H_2O_2 ($2H_2O_2 \rightarrow 2H_2O + O_2\uparrow$), and localized oxygen generation may occur at the nanoplatform interface. We acknowledge that such gas formation may introduce short-range perturbations in particle motion, including transient fluctuations in trajectory and local interfacial disturbances. However, under the relatively low H_2O_2 concentration used in this study (~100 μM), gas evolution is limited and does not support stable bubble nucleation, sustained growth, or directional bubble-driven propulsion as observed in classical bubble-based micromotors. Therefore, bubble formation is considered to contribute only to localized, transient modulation of motion behavior.

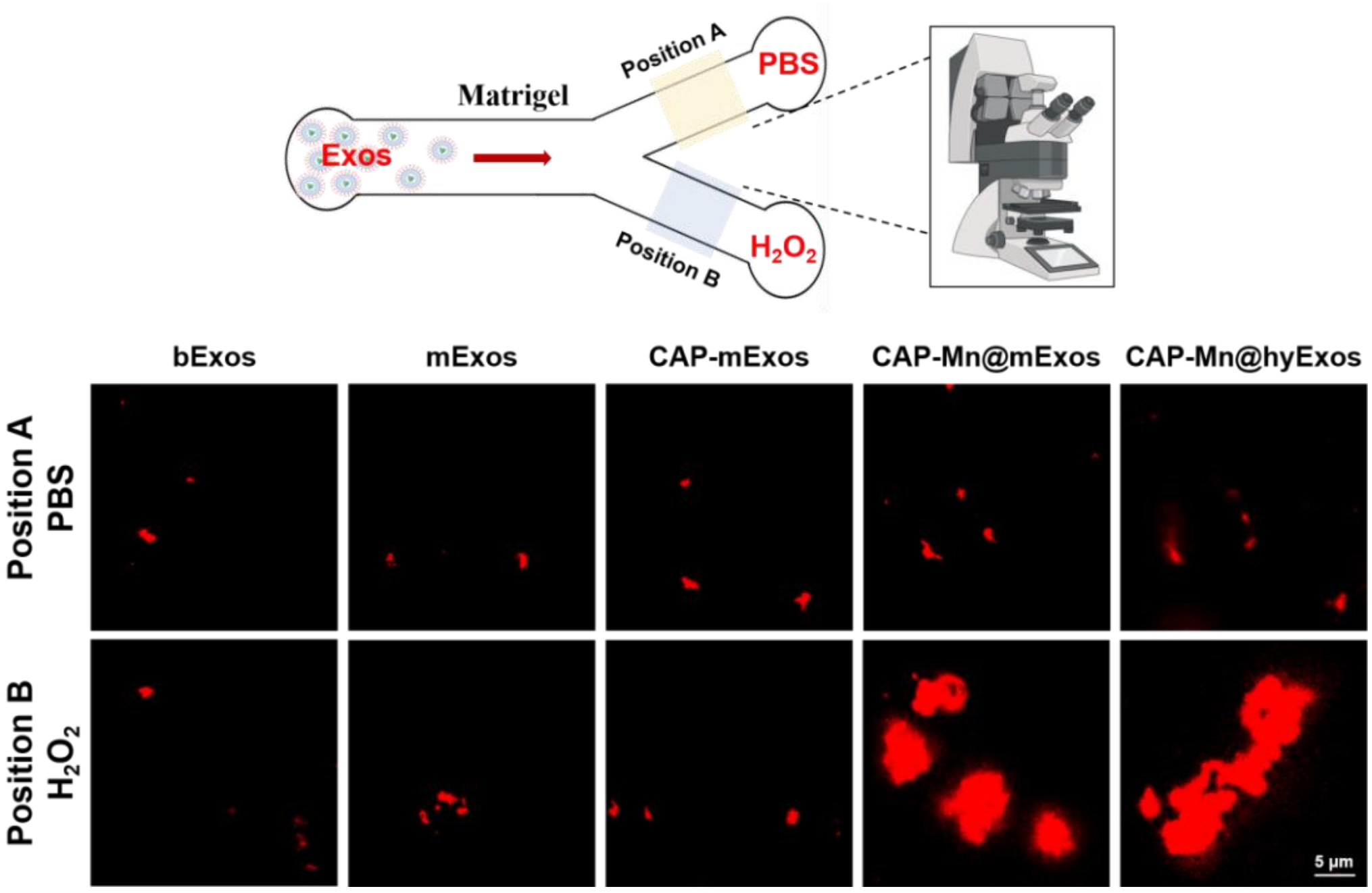

Fig. S18 | Schematic illustration and confocal microscopy images showing the motion and migration of exosomal formulations under H_2O_2-enriched or control conditions in a Y-shaped microfluidic chip, scale bars = 5 μm. Data are representative of three independent experiments with similar results.

Instead, the overall transport behavior arises from reaction-coupled modulation of particle motion under spatially heterogeneous ROS conditions. In particular, MnO_2-mediated redox reactions may modulate the local interfacial activity of individual nanoplatforms, which may lead to local variations in effective motility across ROS-rich and ROS-poor regions. Over time, this generates a statistically biased redistribution toward ROS-enriched areas.

At the tissue level, this process is further reinforced by CAP-mediated interactions with the cartilage extracellular matrix, which enhance matrix affinity and prolong local residence time, thereby stabilizing the ROS-induced redistribution pattern without introducing directional sensing elements.

In summary, the observed transport behavior should be understood as a chemotaxis-like, ROS-responsive redistribution process, rather than classical chemotaxis or bubble-propulsion-driven motion. This behavior emerges from the coupling between MnO_2-mediated ROS reactivity and spatial heterogeneity within the osteoarthritic microenvironment, together with matrix-mediated retention effects under tissue confinement conditions.

The above revisions have been incorporated into the revised manuscript; please refer to lines 234-247 (page 7) and lines 764-775 (page 19) for detailed information.

References

[1] Parent CA, Devreotes PN. A cell's sense of direction. Science. 1999 Apr 30;284(5415):765-70.

[2] Popescu MN, Uspal WE, Bechinger C, Fischer P. Chemotaxis of Active Janus Nanoparticles. Nano Lett. 2018 Sep 12;18(9):5345-5349.

2. The condition for chemotaxis is the presence of an H_2O_2 concentration gradient. The authors demonstrated the chemotactic penetration behavior of the nanomotor in ex vivo joints but should also provide evidence that an H_2O_2 concentration gradient exists in the ex vivo joint. Is this gradient sufficient to ensure chemotaxis?

Response:

We sincerely thank the reviewer for this important question regarding whether an H_2O_2/ROS concentration gradient is required to drive the observed transport behavior.

As clarified in our response to Question 1, the spatial redistribution observed in this system does not rely on classical chemotaxis, which requires long-range sensing of a stable and pre-established concentration gradient. Importantly, even in the presence of spatial variations in ROS levels, such conditions alone are not sufficient to constitute chemotactic migration. Instead, the present system operates through a ROS-responsive transport process and does not depend on a predefined gradient field as a driving requirement.

Mechanistically, the key determinant is not directional sensing, but ROS-responsive modulation of nanoparticle motion mediated by MnO_2. Through catalytic interactions with H_2O_2 and related ROS species, MnO_2 alters the local motion behavior of individual nanoplatforms. In a spatially heterogeneous ROS environment, this leads to position-dependent differences in motion behavior. When integrated over time, these differences result in a biased redistribution toward ROS-enriched regions.

To address the physiological relevance of ROS distribution, we further provided in vivo ROS imaging in an OA rat model, which demonstrates elevated and spatially heterogeneous ROS distribution within cartilage rather than a stable gradient field (**Fig. S20**). This supports the existence of a non-uniform redox landscape in the OA joint microenvironment.

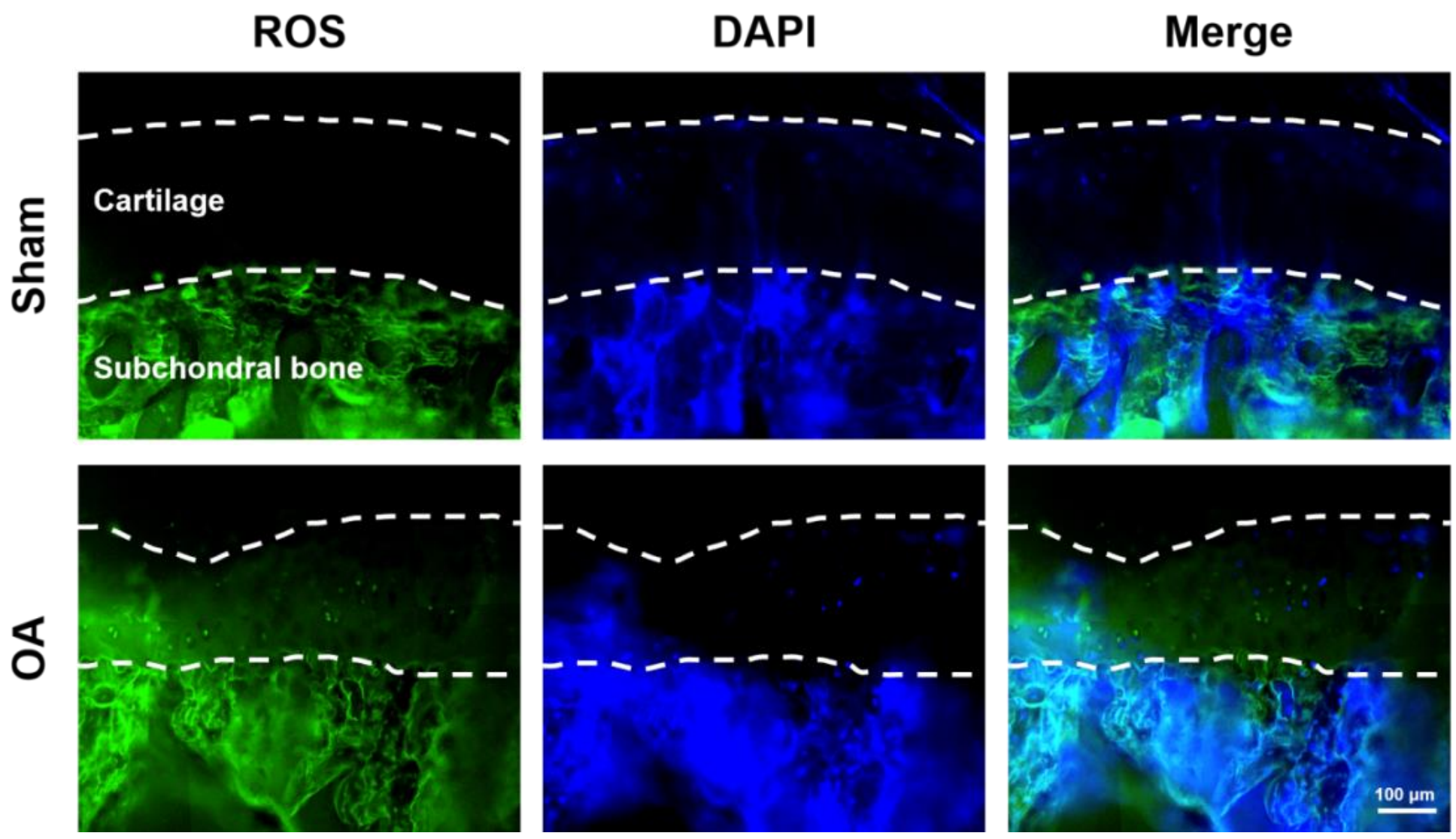

Fig. S20 | Fluorescence imaging of ROS levels and spatial distribution in healthy and osteoarthritic knee cartilage, scale bars = 100 μm. Data are representative of three independent experiments with similar results.

Building on this, both ex vivo and in vivo results consistently validate the transport behavior. In the ex vivo model, H_2O_2 pretreatment established a ROS-enriched microenvironment, in which MnO_2-containing nanoplatforms exhibited significantly enhanced cartilage penetration compared with controls (Figs. 3D–E). Importantly, in vivo experiments further confirmed that, in native OA joints without artificial gradient construction, MnO_2-containing nanomotors still achieved deep cartilage distribution, whereas control exosomes remained largely confined to superficial regions (**Fig. S21**).

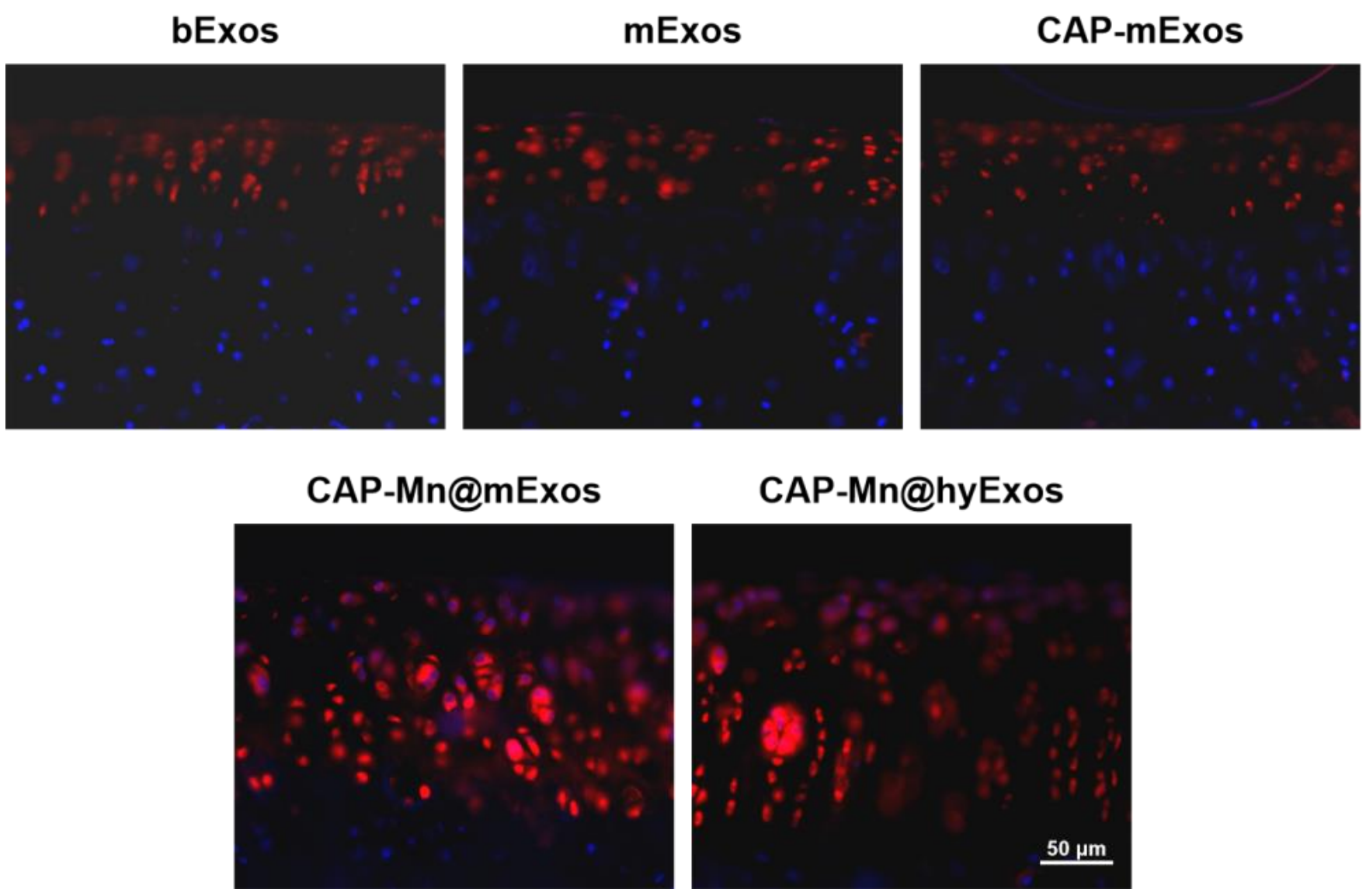

Fig. S21 | Fluorescence images of cartilage from OA rat knees 2 days after intra-articular injection of DiI-labeled exosomal formulations, showing ROS-driven tissue penetration in vivo, scale bars = 50 μm. Data are representative of three independent experiments with similar results.

Taken together, these findings indicate that the observed behavior arises from ROS heterogeneity–driven modulation of nanoparticle motion, rather than gradient-guided chemotaxis. Accordingly, the behavior should be understood as a ROS-responsive transport process governed by spatial microenvironmental differences within osteoarthritic cartilage.

The above revisions have been incorporated into the revised manuscript; please refer to lines 256-269 (page 7) and lines 782-792 (page 19) for detailed information.

3. Many reported chemotaxis systems are based on enzyme-substrate interactions due to the high affinity and specificity of enzymes for their substrates. The authors used the MnO_2 nanoparticle-H_2O_2 system. Does this system possess similar high affinity and specificity? Detailed experiments should be designed to verify this.

Response:

We sincerely thank the reviewer for this important question regarding whether the MnO_2–H_2O_2 system exhibits enzyme–substrate-like affinity and specificity.

We would like to clarify that the MnO_2–H_2O_2 system does not operate through enzyme–substrate-type molecular recognition. In classical enzymatic systems, specificity arises from structural complementarity and binding affinity between enzyme and substrate. In contrast, the interaction between MnO_2 and H_2O_2 is a redox catalytic process governed by electron transfer and reaction kinetics, rather than molecular binding. Therefore, this system does not exhibit enzyme-like affinity or recognition specificity.

Although it does not rely on binding-based interactions, the system shows distinct responsiveness toward chemically different species. To evaluate this, we performed Y-shaped microfluidic experiments under various microenvironmental conditions. The nanoplatform exhibited negligible redistribution under non-redox-active conditions (glucose and urea), showing symmetric diffusion-dominated behavior. In contrast, pronounced redistribution was observed under ROS-related conditions (H_2O_2 and O_2^-), with a stronger response to H_2O_2 (**Fig. S19**).

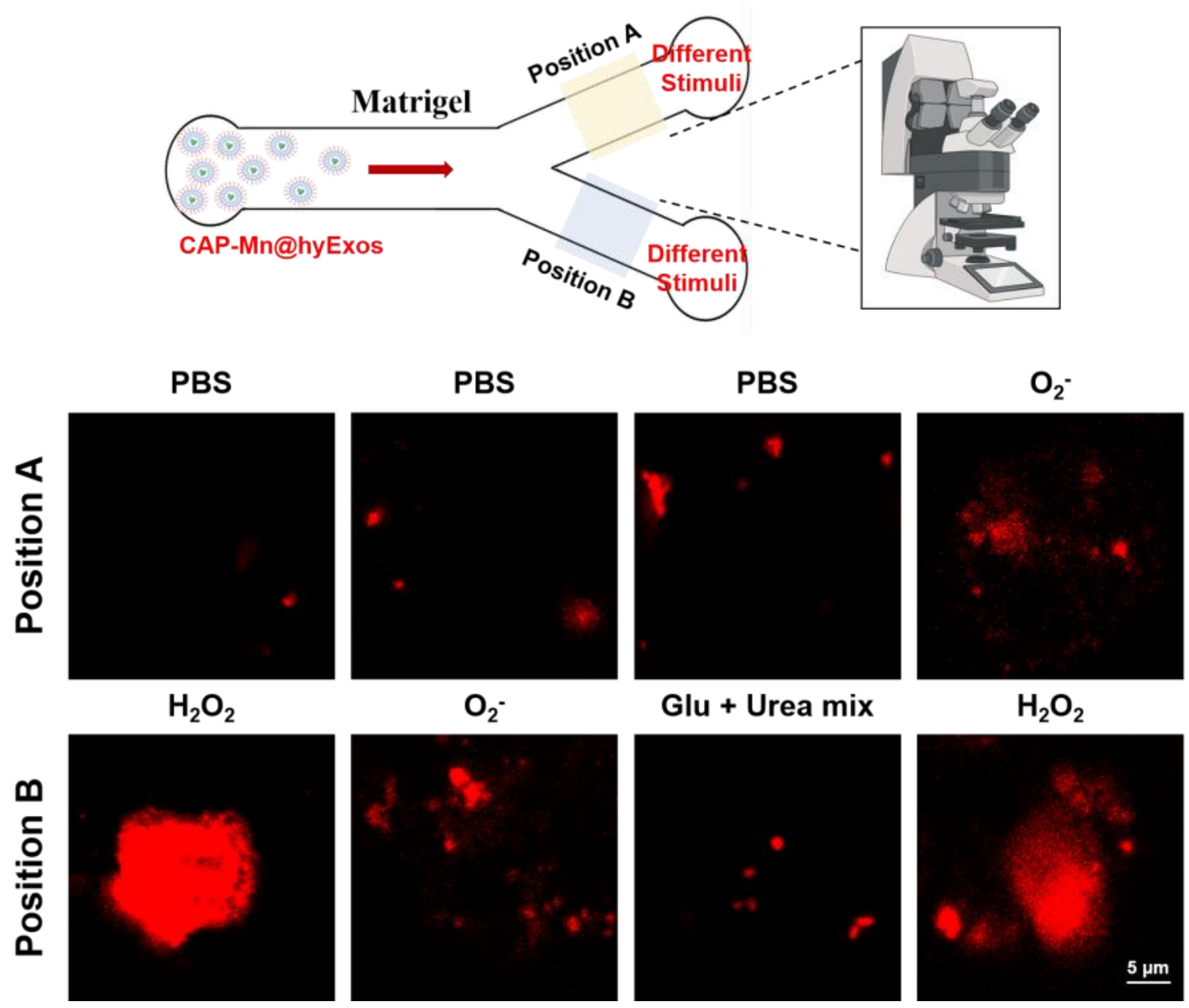

Fig. S19 | Schematic illustration and confocal microscopy images showing the motion and migration of CAP-Mn@hyExos under various microenvironmental conditions, including PBS, urea, glucose, and ROS-related species (H_2O_2 and O_2^-), highlighting the stimulus-selective behavior of the nanomotors, scale bars = 5 μm. Data are representative of three independent experiments with similar results.

This behavior reflects the intrinsic redox reactivity of MnO_2 toward different chemical species. ROS-related molecules such as H_2O_2 can undergo catalytic reactions on the MnO_2 surface, leading to continuous interfacial redox turnover, whereas non-redox-active molecules such as glucose and urea do not participate in such reactions. As a result, MnO_2-containing nanoplatforms exhibit condition-dependent differences in motion behavior under different microenvironmental settings, leading to a clear response hierarchy: $H_2O_2 > O_2^- >>$ glucose/urea.

Taken together, these results demonstrate that the observed behavior arises from differences in redox reactivity and reaction kinetics rather than molecular recognition or enzyme–substrate-like interactions. In this context, the MnO_2-based system should be understood as a ROS-responsive transport process, in which surface catalytic reactions modulate the motion behavior of nanoplatforms in different chemical environments.

The above revisions have been incorporated into the revised manuscript; please refer to lines 240-247 (page 7) and lines 770-775 (page 19) for detailed information.

4. Material characterization needs to be strengthened. In particular, current evidence is insufficient to fully demonstrate that MnO_2 nanoparticles are encapsulated within the exosomes. TEM results are not clear, and more substantial evidence should be provided.

Response:

We sincerely thank the reviewer for the valuable comment regarding the structural evidence supporting MnO_2 incorporation within the exosomal system. In response, we have strengthened the manuscript by adding cryo-electron microscopy (cryo-EM) and EDS elemental mapping, together with previously provided XPS, ICP-OES, and functional data to establish a multi-level validation framework.

To directly examine the spatial localization characteristics of MnO_2 within the exosomal system, cryo-EM was employed. MnO_2 nanoparticles alone exhibited strong electron-dense contrast with well-defined morphology. In control exosomes (bExos, mExos, and CAP-mExos), intact vesicular structures with uniform low electron density were observed, without internal high-density features. In contrast, both CAP-Mn@mExos and CAP-Mn@hyExos consistently displayed discrete electron-dense domains that were confined within vesicle-like boundaries rather than freely dispersed outside the membrane structure. Importantly, these high-density signals were spatially associated with intact exosomal vesicles, while preserving membrane morphology, suggesting that MnO_2-related components are physically associated with the exosomal vesicular compartment rather than existing as free external aggregates (**Fig. S13**).

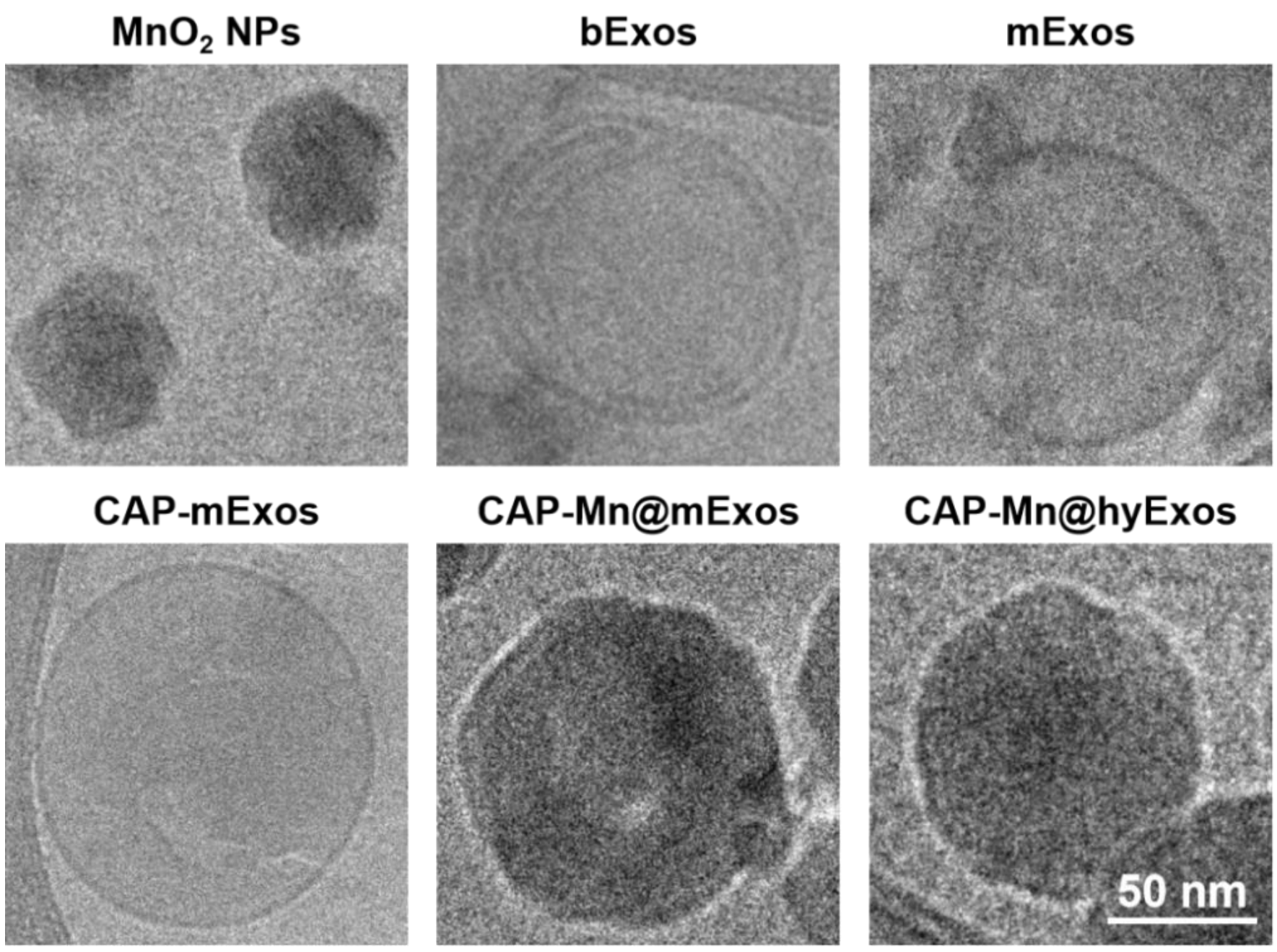

Fig. S13 | Representative cryo-EM micrographs of MnO_2 NPs and various exosomal nanomotors, scale bar = 50 nm. Data are representative of three independent experiments with similar results.

To further support compositional evidence, EDS elemental mapping was performed. Due to the nanoscale size of exosomes, low MnO_2 loading (~4 wt%), and attenuation effects from the organic membrane matrix, Mn signals were not reliably distinguishable from background noise and therefore were not used for spatial localization. However, a consistent increase in nitrogen signal was observed in CAP-Mn@hyExos, consistent with incorporation of BSA-modified MnO_2 components (**Fig. S14**).

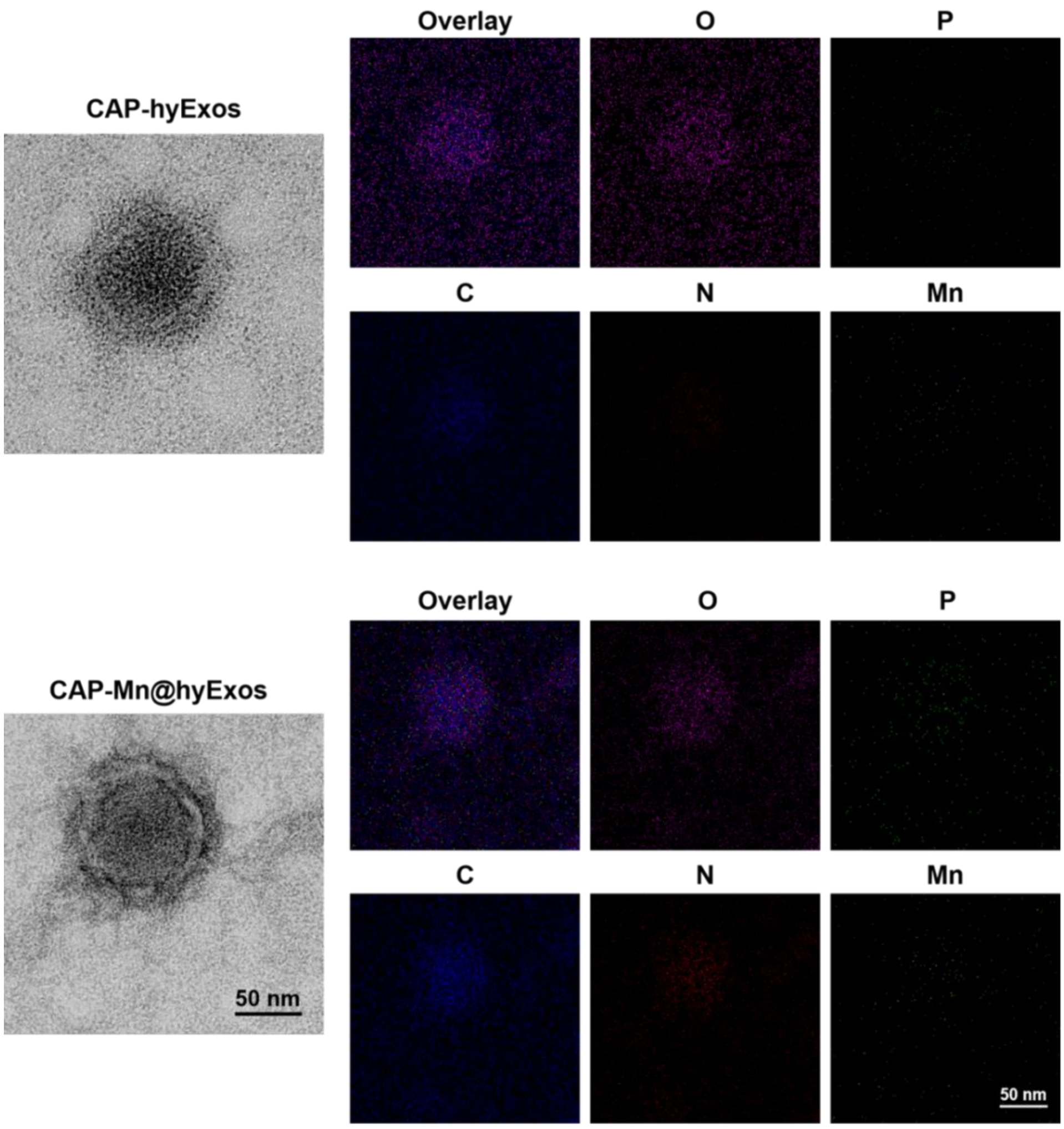

Fig. S14 | Representative EDS mapping micrographs of CAP-hyExos and CAP-Mn@hyExos, scale bar = 50 nm. Data are representative of three independent experiments with similar results.

In addition, XPS confirmed the MnO_2 oxidation state, excluding free ionic manganese species, while ICP-OES quantitatively verified stable Mn loading. Functionally, only

MnO_2-containing formulations exhibited ROS-responsive motility enhancement and improved cartilage penetration, whereas control exosomes did not show such behavior.

Taken together, these results provide convergent structural and functional evidence supporting that MnO_2 is associated with and incorporated into the exosomal vesicular nanoplatform rather than existing as free or externally detached nanoparticles.

The above revisions have been incorporated into the revised manuscript; please refer to lines 186-204 (pages 5-6) and lines 736-741 (page 18) for detailed information.

5. Under what conditions can exosomes release MnO_2 nanoparticles, and why? This point lacks sufficient experimental support. The process of release needs to be characterized in detail.

Response:

We sincerely thank the reviewer for this important question regarding the environment-dependent stability and transformation behavior of MnO_2 within the exosomal nanomotor system. This point is critical for clarifying the structural status and fate of MnO_2 under physiological and pathological conditions, and is closely related to the structural characterization addressed in Question 4.

From a structural perspective, as supported by cryo-EM and complementary characterization in response to Question 4, MnO_2 nanoparticles are physically associated with the exosomal vesicular architecture within the hybrid nanoplatform. Specifically, MnO_2 is distributed in close association with the vesicle structure rather than existing as free, detached, or independently dispersible particles. Therefore, MnO_2 should be understood as an integrated structural component of the hybrid exosomal system rather than a separable payload.

Under physiological and osteoarthritis (OA)-relevant microenvironments (neutral to mildly acidic pH with low to moderate ROS levels), MnO_2 primarily functions as a catalase-mimicking nanozyme, catalyzing the decomposition of H_2O_2 into water and oxygen ($2H_2O_2 \rightarrow 2H_2O + O_2\uparrow$). In this regime, MnO_2 maintains its structural association within the exosomal platform through reversible surface redox cycling. Therefore, no effective MnO_2 detachment or release is observed under therapeutic conditions.

In contrast, under non-physiological conditions characterized by high oxidative stress and

acidic environments (high H_2O_2 concentration and low pH), MnO_2 may undergo redox-driven structural conversion ($MnO_2 + H_2O_2 + 2H^+ \rightarrow Mn^{2+} + 2H_2O + O_2\uparrow$), resulting in degradation into soluble manganese species. This process represents an environment-triggered structural transformation of the MnO_2 component within the hybrid system, rather than a physiological release event during intra-articular administration.

To experimentally validate the structural stability and conditional transformation behavior, we systematically evaluated the system from multiple complementary perspectives.

First, after 7 days of incubation in simulated synovial fluid, all exosomal formulations maintained stable hydrodynamic diameters within 110–130 nm, with PDI values consistently ranging from 0.10 to 0.25. No obvious aggregation or structural disruption was observed, indicating excellent structural stability under physiological joint conditions (**Fig. S15**).

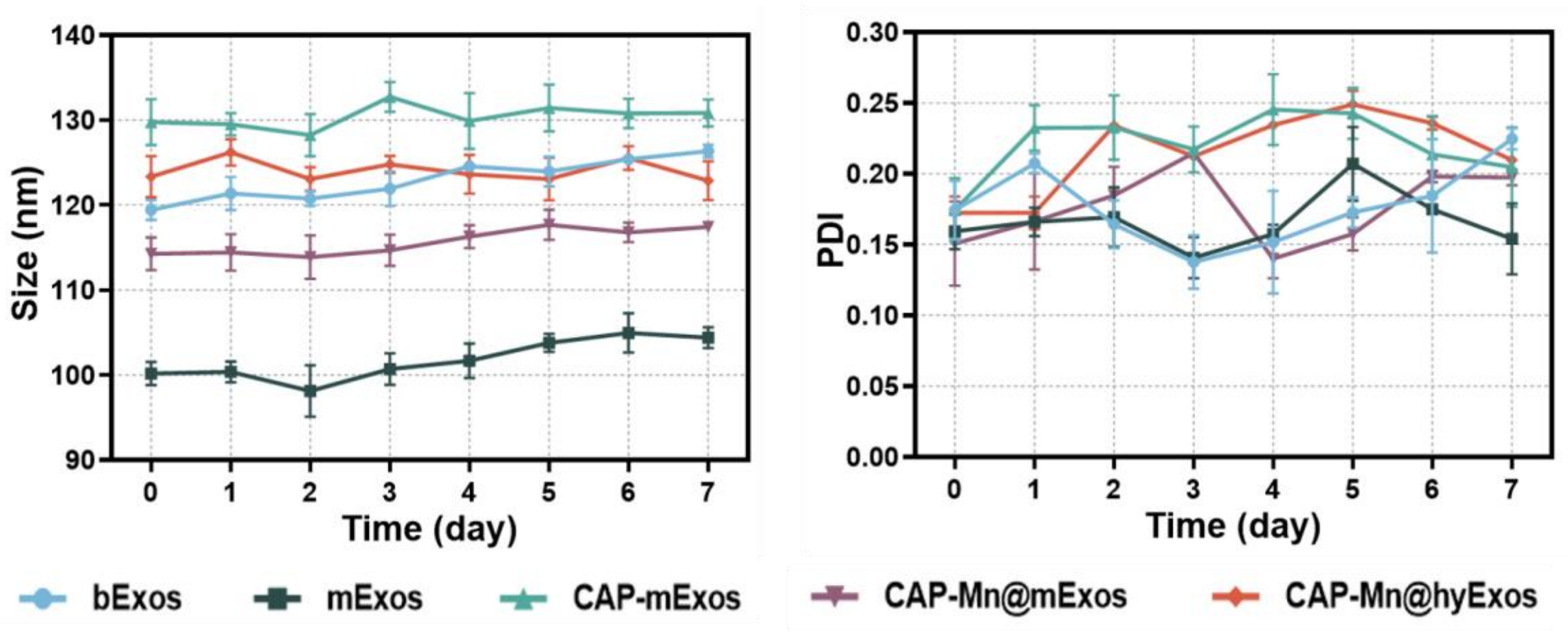

Fig. S15 | DLS analysis of hydrodynamic size and PDI of exosomal formulations in simulated synovial fluid over 7 days (n = 3). Data are presented as the mean ± SD. Source data are provided as a Source Data file.

Second, fluorescence-based stability assays showed only minimal signal variations under simulated synovial fluid. Slight increases were observed under 100 μM H_2O_2 and OA-mimicking conditions (pH 6.8 + 100 μM H_2O_2), while the overall signal remained low and stable. In contrast, under strongly oxidative conditions (≥1 mM H_2O_2), a pronounced increase in fluorescence intensity was detected, indicating significant structural destabilization and environment-induced transformation (**Fig. 2K**).

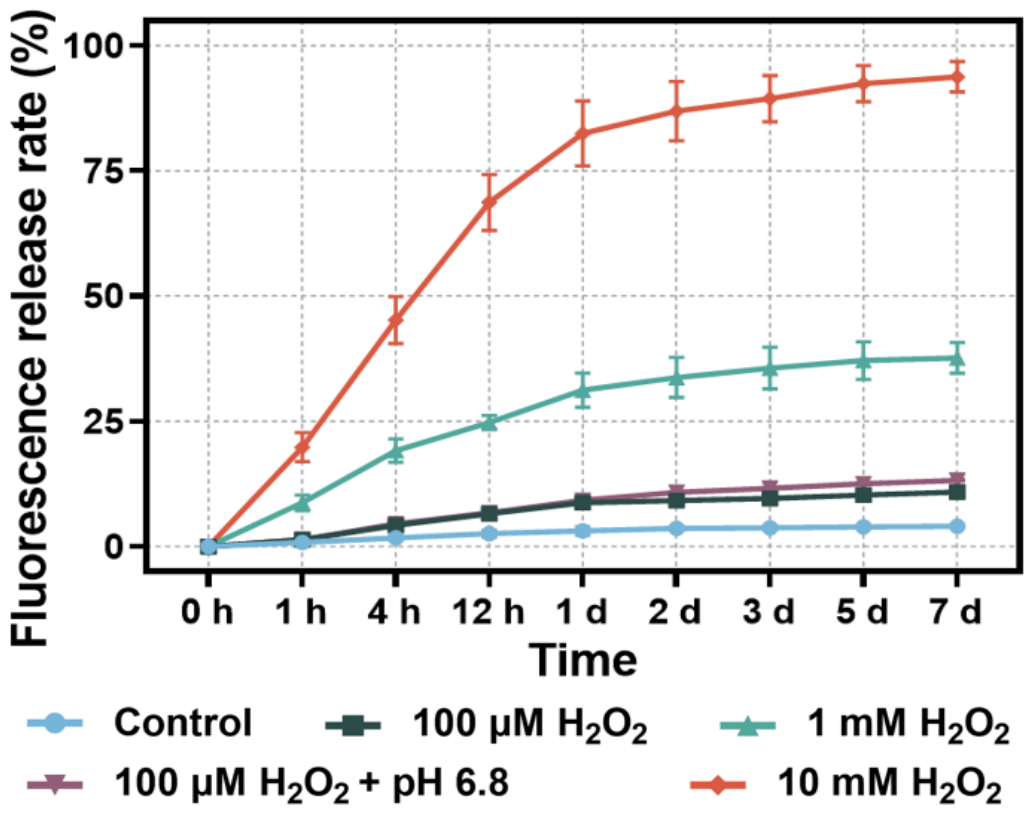

Fig. 2K | Fluorescence release of DiI-labeled CAP-Mn@hyExos over 7 days under OA-relevant microenvironmental conditions (n = 3). Data are presented as the mean ± SD. Source data are provided as a Source Data file.

Furthermore, DLS analysis provided consistent evidence supporting this behavior. Under physiological conditions, particle size remained stable (120–125 nm, PDI 0.10–0.15). Only minor fluctuations were observed under 100 μM H_2O_2 and OA-like conditions (~140 nm, PDI 0.10–0.27), with no indication of aggregation or structural collapse. However, under strongly oxidative conditions, particle size increased markedly to 370–490 nm with elevated PDI (>0.60), indicating pronounced structural disruption **(Figs. 2L & S16**).

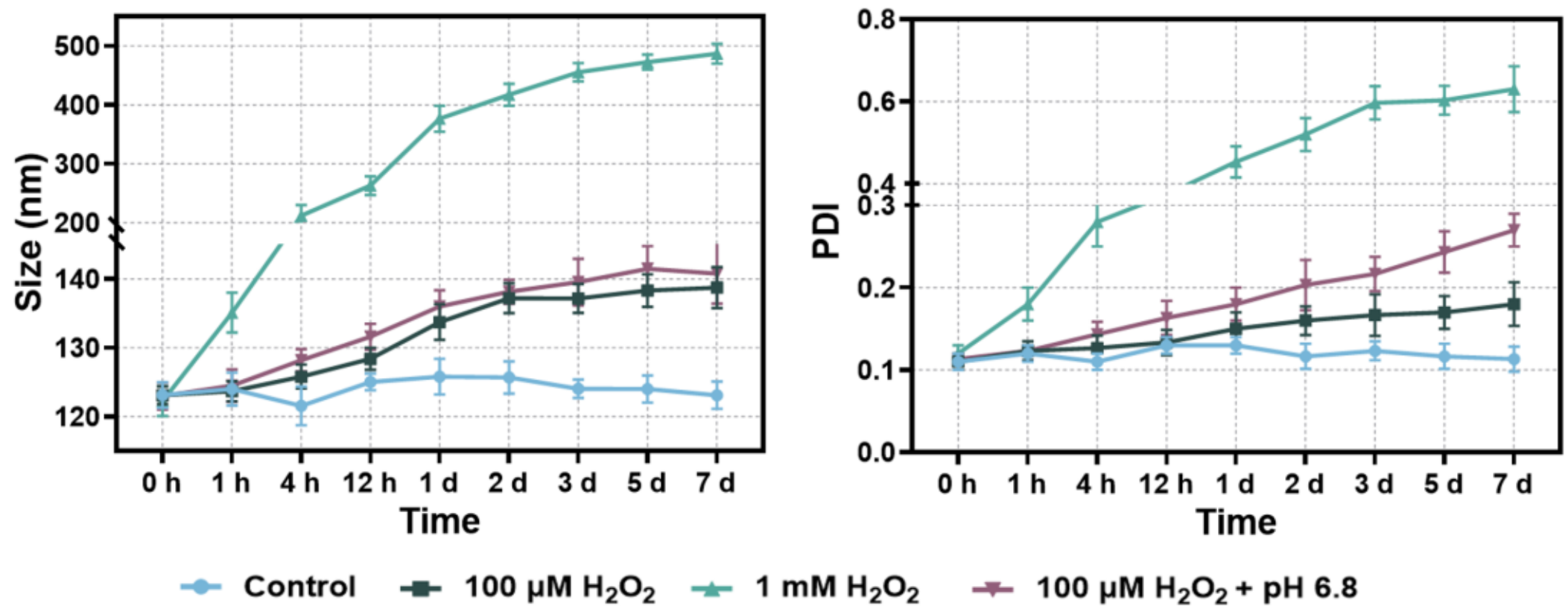

Figs. 2L & S16 | DLS analysis of size and PDI of CAP-Mn@hyExos over 7 days under OA-relevant microenvironmental conditions (n = 3). Data are presented as the mean ± SD. Source data are provided as a Source Data file.

Collectively, these independent datasets consistently demonstrate that the exosomal nanomotor system maintains structural integrity under physiological and OA-relevant microenvironments, while MnO_2 remains structurally associated within the hybrid vesicular architecture. Only under extreme oxidative stress conditions does MnO_2 undergo

environment-triggered structural conversion.

Accordingly, the term “release” does not refer to a physical detachment process under physiological conditions in this system. Instead, MnO_2 is stably integrated within the hybrid nanoplatform during intra-articular administration and participates in catalytic function in an associated state. Environment-induced changes observed under non-physiological conditions correspond to structural transformation rather than conventional release behavior.

The above revisions have been incorporated into the revised manuscript; please refer to lines 205-221 (page 6) and lines 741-755 (page 18) for detailed information.

6. Chemotactic movement typically involves sensing the concentration gradient of an inducer over long distances. Therefore, the sensing components in nanomotors are generally exposed externally to sensitively detect inducer concentration gradients. In this work, MnO_2 nanoparticles are encapsulated inside the exosomes. How can chemotactic movement still occur under such conditions?

Response:

We sincerely thank the reviewer for this insightful question regarding how chemotaxis-like behavior can arise when MnO_2 is encapsulated within exosomes without externally exposed sensing components. This clarification is essential for correctly interpreting the transport mechanism in this study.

We would like to first clarify that the nanomotor system does not operate via classical chemotaxis. It does not rely on receptor-mediated recognition, externally exposed sensing elements, or long-range sensing of a pre-existing concentration gradient. Instead, the observed spatial redistribution behavior arises from a ROS-responsive transport process, as consistently described in our responses to previous comments.

Mechanistically, although MnO_2 is encapsulated within the exosomal structure, low-molecular-weight ROS such as H_2O_2 can diffuse through biological matrices and access the MnO_2-containing domain of the hybrid nanoplatform. This enables localized redox reactions ($2H_2O_2 \rightarrow 2H_2O + O_2\uparrow$) to occur at the particle–microenvironment interface.

Importantly, these reactions do not generate directional propulsion or any long-range sensing behavior. Instead, they locally regulate the motion behavior of individual nanoplatforms by

altering their interfacial reaction activity. In practical terms, this reaction activity influences how frequently and how strongly individual particles undergo local motion fluctuations, including changes in trajectory persistence and effective diffusion behavior.

Because ROS levels are spatially heterogeneous in osteoarthritic tissue, nanoplatforms located in ROS-enriched regions experience more frequent catalytic reactions than those in ROS-poor regions. This leads to position-dependent differences in their stochastic motion behavior over time. Although each individual particle moves randomly, these local differences accumulate at the population level, resulting in a statistically biased redistribution toward ROS-enriched regions.

In this framework, it does not rely on external sensing architecture. Spatial information is instead encoded by intrinsic ROS heterogeneity, translated into local differences in reaction frequency via MnO_2 catalysis, and then converted into macroscopic redistribution through cumulative stochastic motion.

At the tissue level, CAP further enhances this process by increasing interactions with the extracellular matrix, which prolongs local residence time and reduces random back-diffusion. Importantly, CAP does not provide directional guidance, but instead helps maintain the ROS-induced redistribution bias once formed.

Therefore, the observed behavior should be understood as a ROS-responsive transport process governed by spatially heterogeneous reaction-dependent modulation of stochastic motion, rather than classical chemotaxis. The term “chemotaxis-like” refers only to the emergent spatial accumulation pattern at the tissue scale, not to receptor-mediated gradient sensing.

The above revisions have been incorporated into the revised manuscript; please refer to lines 547-563 (page 14) for detailed information.

7. At least 20 particle trajectories should be provided to generate a motion velocity distribution plot.

Response:

We sincerely thank the reviewer for the constructive suggestion regarding the statistical characterization of nanomotor motion. As correctly pointed out, population-level trajectory analysis is essential for accurately capturing the overall motility behavior of the nanomotors.

In response, we have supplemented the revised manuscript with independent trajectories of 20 individual particles per group and constructed velocity distribution profiles to more comprehensively characterize population-level motion behavior. In the revised **Figs. 3C & S17**, both velocity distribution histograms and trajectory-based scatter plots are provided, complementing the average velocity analysis.

The results show that, under 100 μM H_2O_2 stimulation, the velocity distribution of MnO_2-loaded nanomotors exhibits a clear rightward shift accompanied by a broader distribution profile. Specifically, pristine exosomes without MnO_2 are predominantly confined to a low-velocity range (~2–3 μm/s), consistent with Brownian diffusion-dominated motion. In contrast, MnO_2-loaded nanomotors display enhanced motility, characterized by an overall shift toward higher velocities and a measurable fraction of particles exhibiting velocities above 8 μm/s.

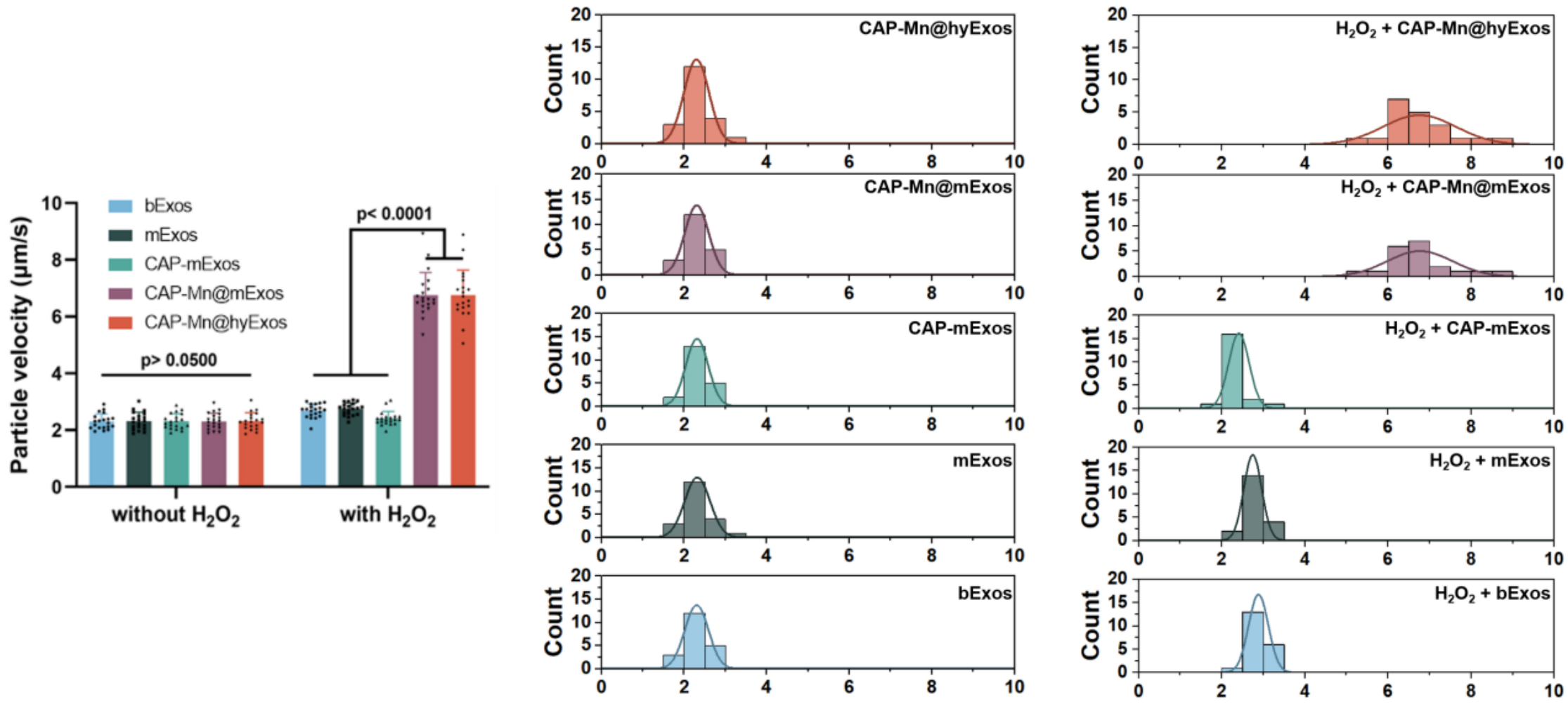

Figs. 3C & S17 | Particle velocity quantitative evaluation and distribution of exosomal formulations with or without H_2O_2 (n = 20). Data are presented as the mean ± SD. Source data are provided as a Source Data file.

Importantly, this increase is not driven by a small subset of outlier trajectories, but reflects a systematic shift across the particle population. This distribution pattern is consistently observed across independent experimental repeats, supporting its statistical robustness as a population-level phenomenon rather than stochastic variation.

In summary, velocity distribution analysis based on 20 individual particle trajectories provides statistically supported evidence for enhanced motility of the nanomotors, offering robust quantitative validation of MnO_2-mediated catalytic modulation of particle dynamics.

8. Is the additional H_2O_2 added by the authors comparable to in vivo concentrations? This should be demonstrated.

Response:

We sincerely thank the reviewer for this important question regarding whether the added H_2O_2 is comparable to physiological in vivo levels. This is an important point for clarifying the physiological relevance of the experimental design.

Previous studies have shown that ROS levels are significantly elevated in osteoarthritic (OA) cartilage and exhibit pronounced spatial heterogeneity with local enrichment. Due to the highly dynamic nature of H_2O_2 in vivo and its rapid turnover and clearance, its absolute concentration within the joint microenvironment is difficult to directly quantify. Accordingly, most related studies evaluate H_2O_2 in terms of functional oxidative stress levels and biological responses rather than relying on fixed absolute concentration values.

Based on this rationale, we adopted a functional oxidative stress modeling strategy in vitro to recapitulate a pathologically relevant OA-like microenvironment. Specifically, chondrocytes were co-incubated with H_2O_2, and the oxidative stress window was defined based on the half-maximal inhibitory concentration (IC_{50}) derived from cell viability assays (Fig. S38). On this basis, 100 µM H_2O_2 was selected, which maintains cell viability while inducing a stable and reproducible oxidative stress state, thereby providing a physiologically relevant condition for evaluating ROS-responsive behavior of the nanomotors.

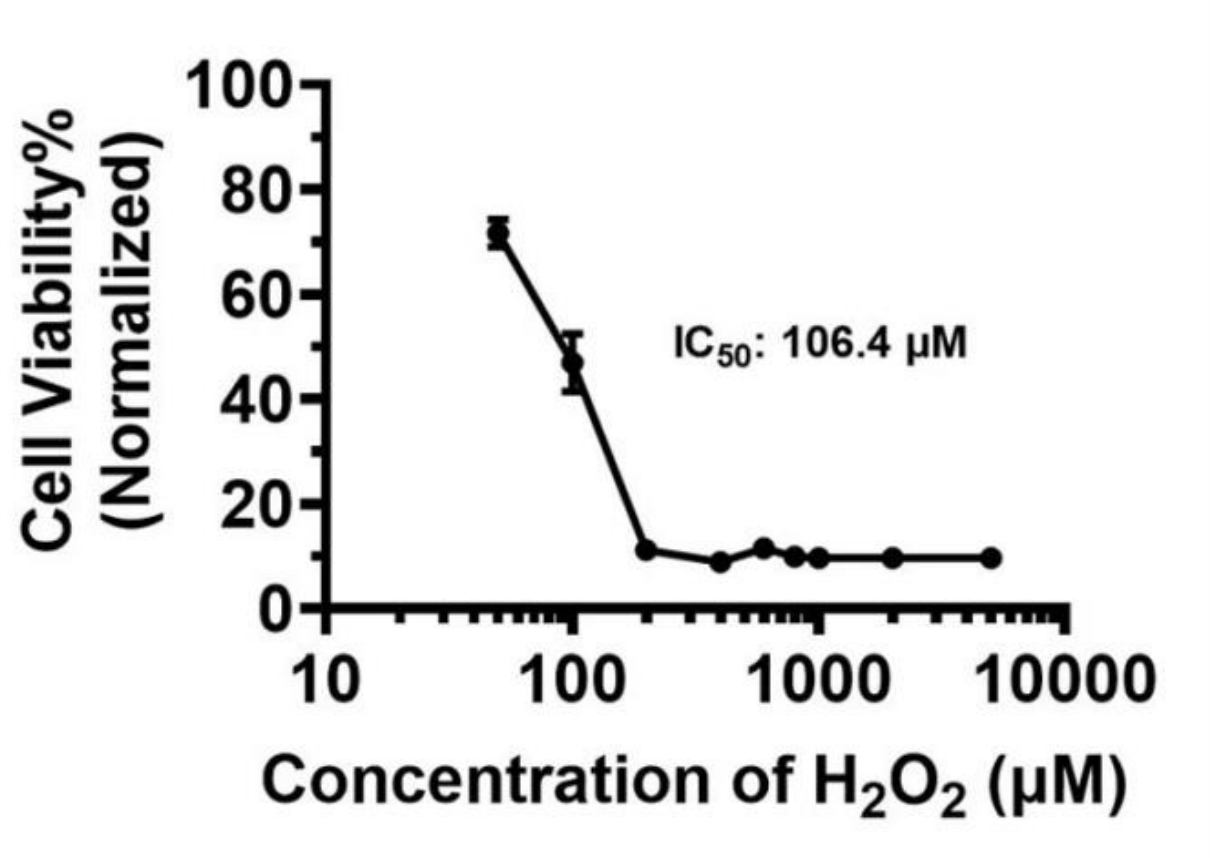

Fig. S38 | Determination of the oxidative stress working window of H_2O_2 in chondrocytes based on IC_{50}-guided viability analysis (n = 3). Data are presented as the mean ± SD. Source data are provided as a Source Data file.

Furthermore, in the established OA rat model, ROS imaging using fluorescent probes demonstrated that, compared with healthy cartilage, OA cartilage exhibited markedly elevated ROS levels accompanied by pronounced spatial heterogeneity and localized enrichment **(Fig. S20)**. This in vivo observation is consistent with the oxidative stress conditions modeled in vitro, thereby supporting the pathological relevance of our experimental design.

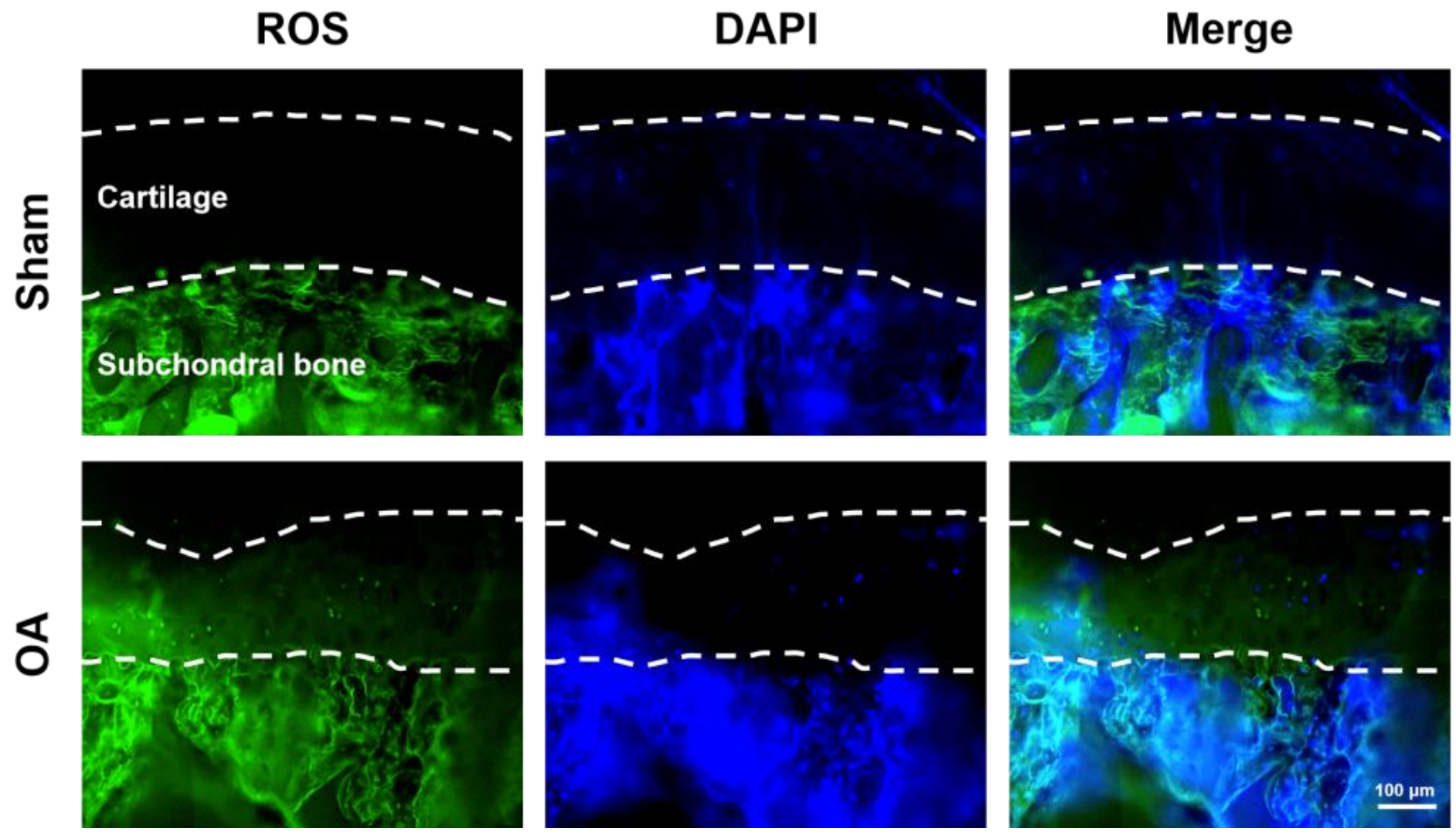

Fig. S20 | Fluorescence imaging of ROS levels and spatial distribution in healthy and osteoarthritic knee cartilage, scale bars = 100 μm. Data are representative of three independent experiments with similar results.

In summary, this study does not aim to directly match a single absolute in vivo H_2O_2 concentration. Instead, by integrating IC_{50}-guided definition of a cytocompatible oxidative stress window with in vivo validation of elevated and spatially heterogeneous ROS distribution in osteoarthritic cartilage, we establish a functionally consistent oxidative stress framework across in vitro and in vivo settings. Accordingly, the selected H_2O_2 concentration should be interpreted as a functionally relevant oxidative stress condition that recapitulates key pathological features of OA cartilage, rather than a strict quantitative surrogate of in vivo H_2O_2 levels.

The above revisions have been incorporated into the revised manuscript; please refer to lines 256-259 (page 7) and lines 782-787 (page 19) for detailed information.

9. The penetration capability of the nanomotor in vivo needs to be evaluated in detail (e.g., penetration depth, co-localization with chondrocytes, specific

Response:

We sincerely thank the reviewer for this constructive suggestion regarding the in vivo penetration capability of the nanomotors. In response, we systematically evaluated cartilage penetration by integrating ex vivo cartilage penetration analysis with in vivo intra-articular distribution studies in an osteoarthritic rat model.

In the ex vivo femoral head cartilage model, H_2O_2 pretreatment was used to simulate an osteoarthritic oxidative microenvironment, followed by incubation with DiI-labeled exosomal formulations for penetration assessment. The results showed that control groups (mExos, bExos, and CAP-mExos) were largely restricted to the superficial cartilage region, indicating limited matrix penetration. In contrast, MnO_2-containing nanomotors (CAP-Mn@mExos and CAP-Mn@hyExos) exhibited time-dependent migration into deeper cartilage layers, with detectable fluorescence signals observed in the deep cartilage zone after 6 days of incubation. Quantitative analysis indicated a penetration depth of approximately 140 μm, representing an approximately four-fold increase compared with control groups (Figs. 3D–E), demonstrating significantly enhanced cartilage matrix traversal capability.

To further validate these findings in vivo, an osteoarthritic rat model was established. ROS imaging confirmed that OA cartilage exhibits elevated and spatially heterogeneous ROS distribution, providing a pathological microenvironmental basis for evaluating ROS-responsive transport behavior (**Fig. S20**).

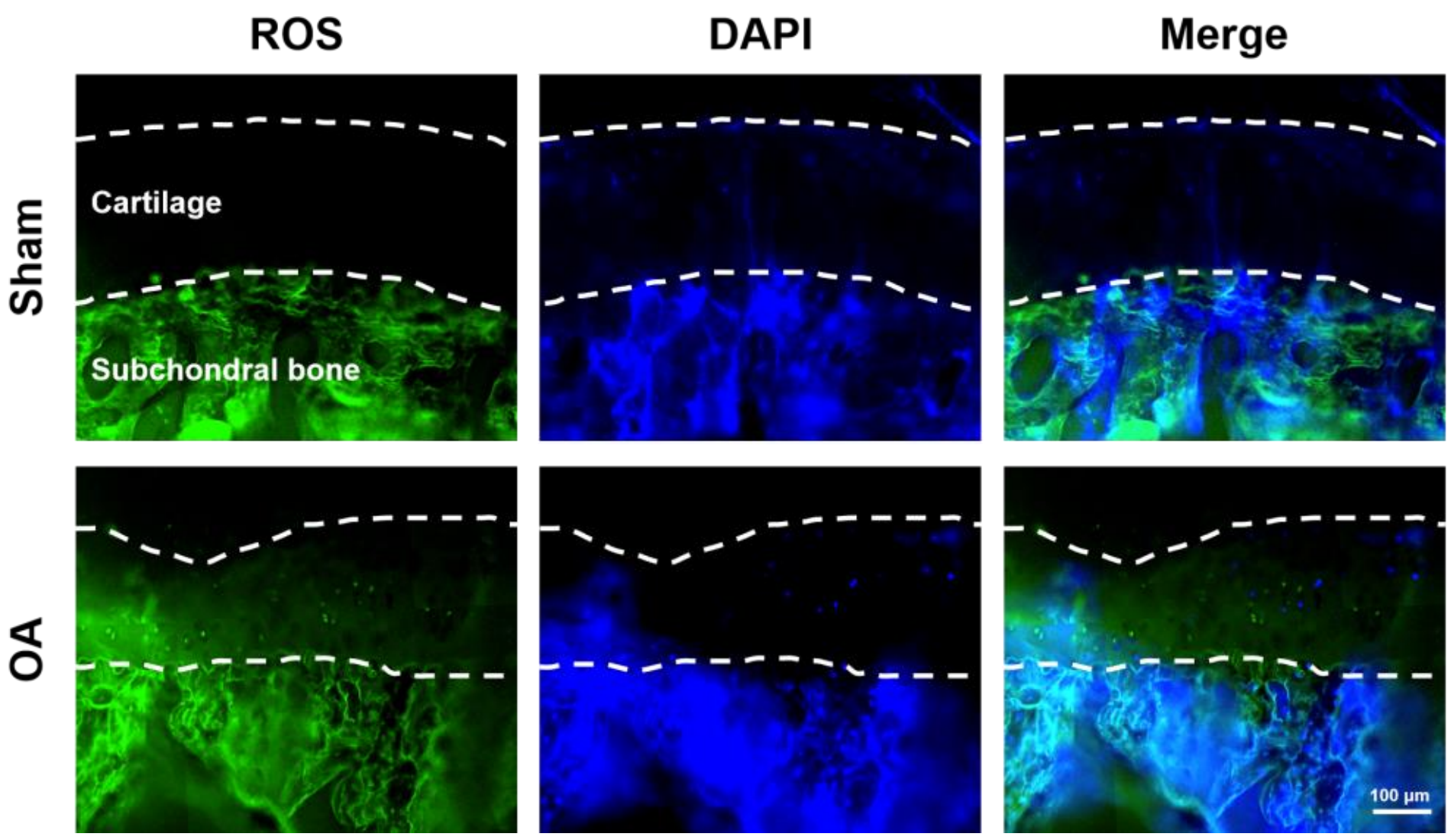

Fig. S20 | Fluorescence imaging of ROS levels and spatial distribution in healthy and osteoarthritic knee cartilage, scale bars = 100 μm.

Following intra-articular injection of DiI-labeled formulations, in vivo fluorescence imaging at 2 days post-administration showed that control exosomes remained primarily confined to the superficial cartilage layer, whereas CAP-Mn@mExos and CAP-Mn@hyExos exhibited markedly enhanced distribution within deeper cartilage regions, indicating improved intra-cartilage infiltration in the osteoarthritic joint environment (**Fig. S21**). Importantly, co-staining with DAPI (nuclear marker) further revealed that fluorescence signals from MnO_2-containing nanomotors were distributed in regions containing abundant cell nuclei within cartilage tissue sections, indicating their localization within cell-populated cartilage microregions and close spatial association with chondrocyte lacunae.

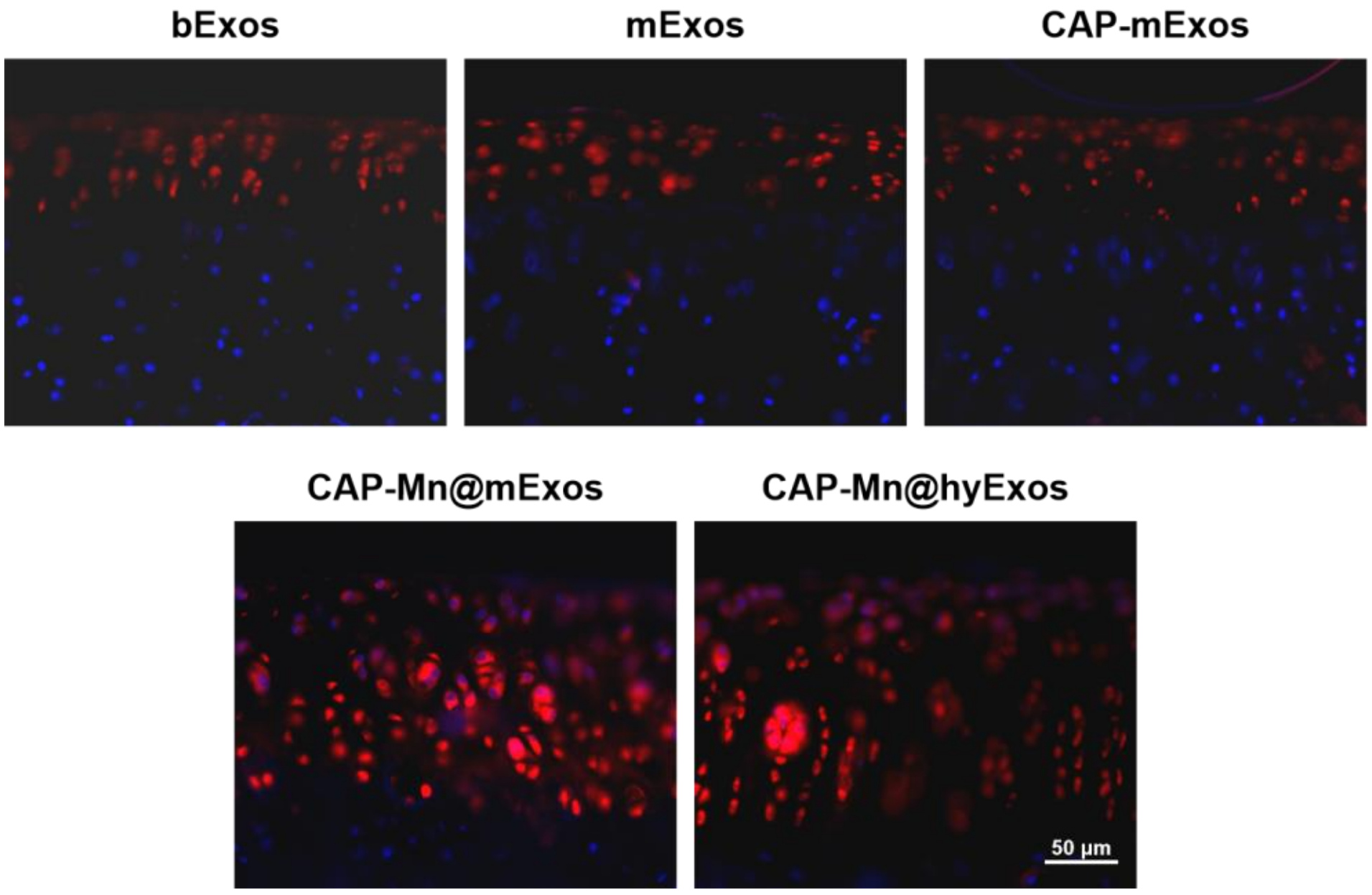

Fig. S21 | Fluorescence images of cartilage from OA rat knees 2 days after intra-articular injection of DiI-labeled exosomal formulations, showing ROS-driven tissue penetration in vivo, scale bars = 50 μm. Data are representative of three independent experiments with similar results.

Collectively, the ex vivo and in vivo results are consistent and demonstrate significantly enhanced cartilage penetration capability of the MnO_2-containing nanomotor systems. These findings support that the incorporation of MnO_2, together with enhanced tissue retention characteristics, enables improved intra-cartilage distribution within osteoarthritic joints.

The above revisions have been incorporated into the revised manuscript; please refer to lines 256-269 (page 7) and lines 782-792 (page 19) for detailed information.

Reviewer #3 (Remarks to the Author):

This research reports an innovative cascade chemotactic hybrid exosomal nanomotor (CAP-Mn/140@hyExos) for precision treatment of osteoarthritis (OA). Through integrated design, this work combines the catalytic self-propulsion capability of MnO_2 NPs, the precise recognition ability of CAP, and engineered exosomes overexpressing miR-140. This aims to synergistically address key bottlenecks in OA treatment, including short intra-articular drug retention time, difficulty in penetrating the cartilage matrix, and low chondrocyte targeting efficiency. The research forms a complete closed-loop, spanning the construction and characterisation of the nanocarrier to in vitro and in vivo functional validation and mechanism exploration. This work demonstrates considerable innovation and holds potential for clinical translation.

Response:

We sincerely thank the reviewer for the positive evaluation and constructive comments. We greatly appreciate the reviewer's recognition of the innovative design and translational potential of our hybrid exosomal nanomotor system for osteoarthritis therapy.

In response to the reviewer's suggestions, we have carefully revised the manuscript and further strengthened the study through additional experimental characterizations, methodological clarifications, and supplementary analyses, including material characterization, OA model description, dosing information, biosafety evaluation, and mechanistic validation, as detailed in our point-by-point responses below.

In addition, in view of the conceptual concerns raised during the review process, we have further refined the mechanistic description throughout the revised manuscript. Specifically, the term "chemotactic" has been removed to avoid potential ambiguity with classical chemotaxis, and the system is now consistently described as a ROS-responsive transport process regulated by spatially heterogeneous microenvironmental conditions and coupled with tissue–matrix interactions.

We believe that these revisions have significantly improved the clarity, mechanistic consistency, and rigor of the manuscript, and have further strengthened its overall quality.

Major concerns:

1. Membrane fusion employed a sequential ‘incubation-extrusion’ method. FRET efficiency data for the final product were not presented. Clearly state in the methods or results that following sequential processing, the size distribution (PDI) of the final product.

Response:

We sincerely thank the reviewer for this valuable suggestion regarding the validation of membrane fusion efficiency and the physicochemical characterization of the final hybrid exosomes. In response, we have now included the previously missing FRET analysis of the final product and the size distribution characterization of the prepared hybrid exosomes in the revised manuscript.

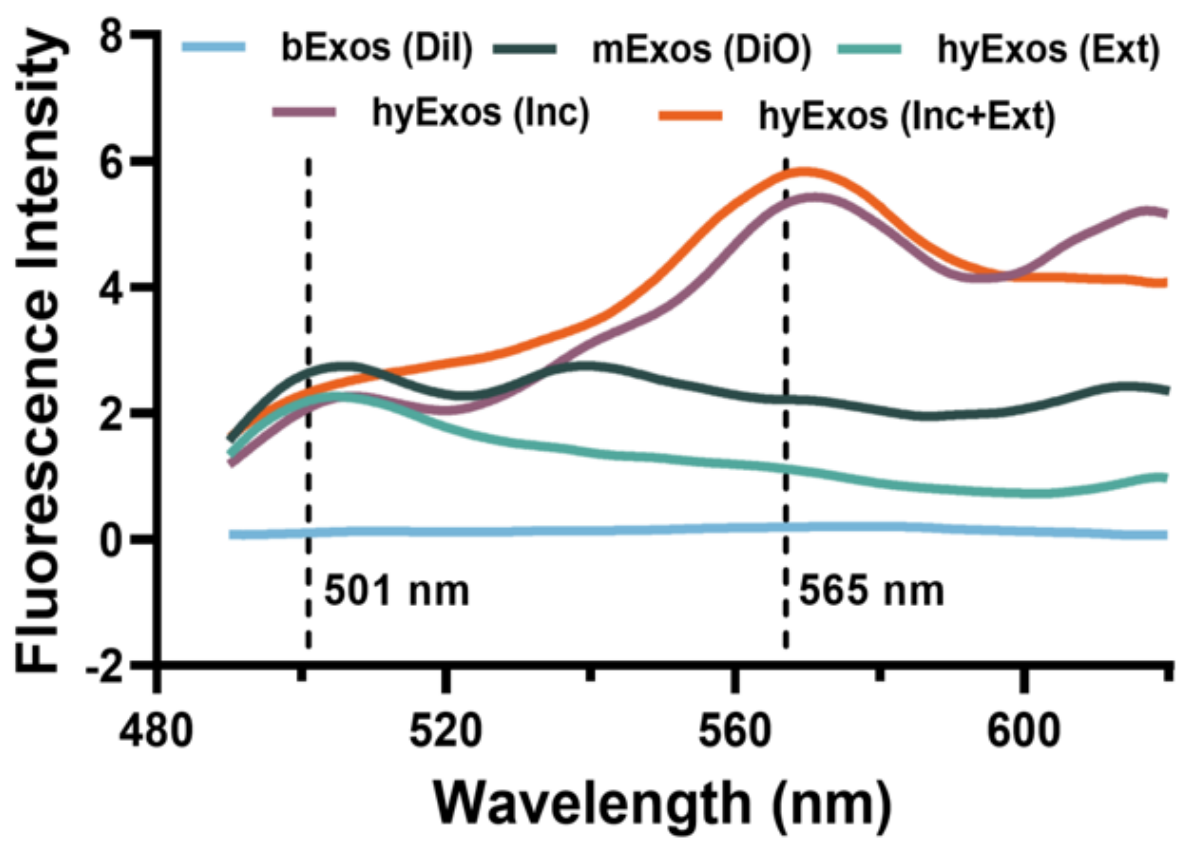

Fig. 2D | FRET-based analysis of membrane fusion between exosomes using incubation, extrusion, and combined “incubation-extrusion” methods. Data are presented as the mean ± SD. Source data are provided as a Source Data file.

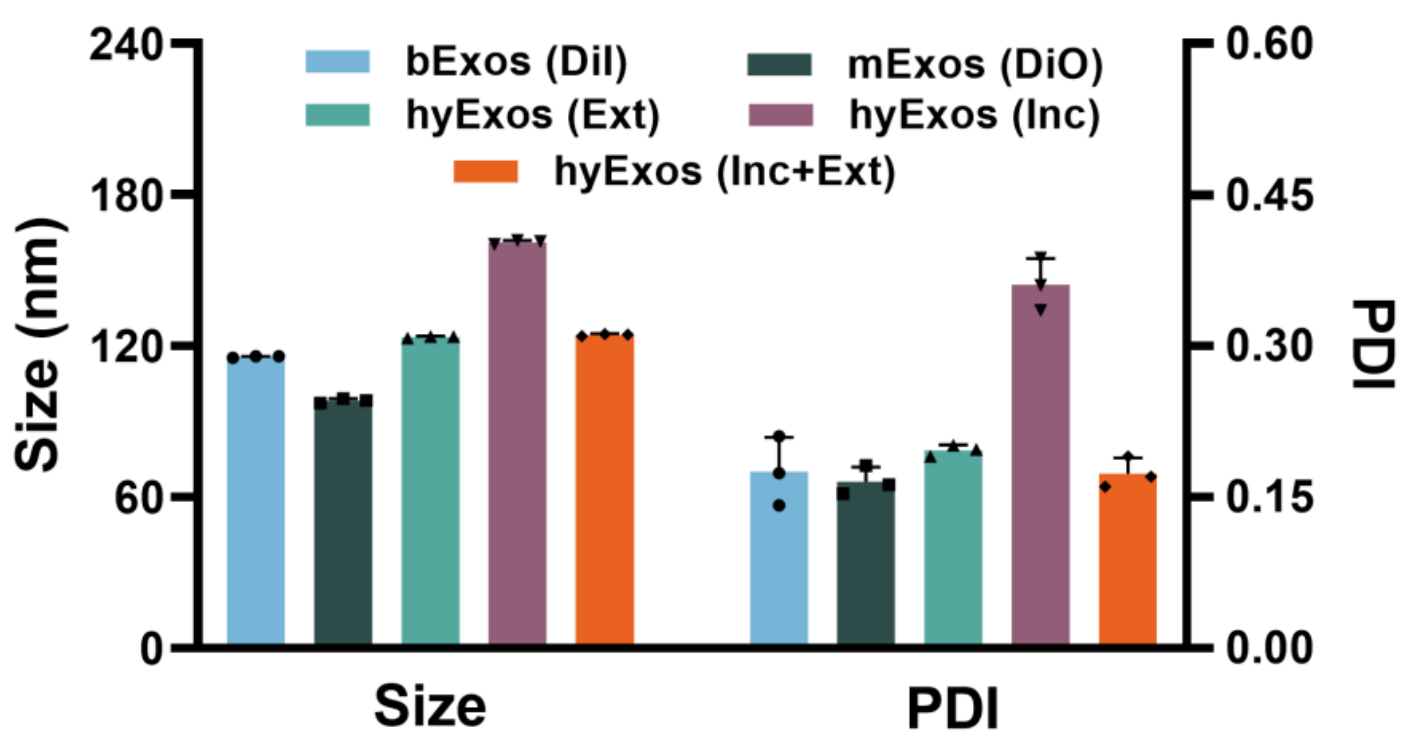

Fig. S11 | Analysis of particle size and PDI for membrane fusion between exosomes using incubation, extrusion, and combined “incubation-extrusion” methods (n = 3). Data are presented as the mean ± SD. Source data are provided as a Source Data file.

Specifically, FRET analysis was performed to evaluate membrane fusion efficiency during the construction of hybrid exosomes. The sequential "incubation–extrusion" strategy was designed to balance fusion efficiency and structural homogeneity. While incubation promotes efficient membrane fusion, extrusion improves particle uniformity, and the combined process enables simultaneous optimization of both parameters (**Figs. 2D & S11**).

Importantly, FRET analysis of the final CAP-Mn/140@hyExos confirms successful membrane fusion, with a fusion efficiency of approximately 70%, indicating effective integration of the exosomal membranes after sequential processing. In addition, nanoparticle tracking analysis (NTA) of the final hybrid exosomes has now been explicitly included in the revised manuscript. The CAP-Mn/140@hyExos exhibit a uniform hydrodynamic diameter with a narrow size distribution and a low polydispersity index (PDI < 0.2), demonstrating good colloidal homogeneity and structural stability of the final product **(Figs. 2E & S11)**.

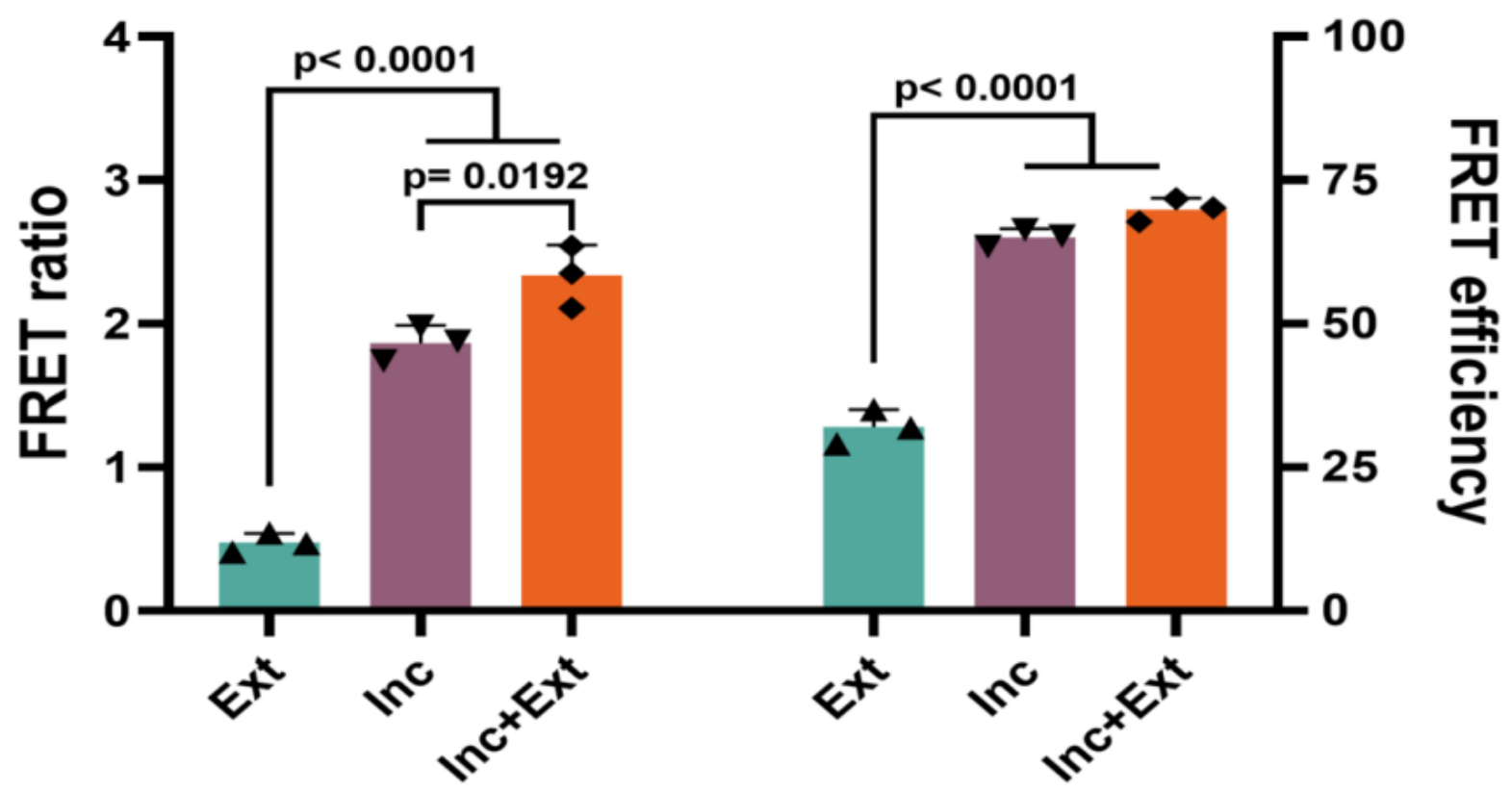

Fig. 2E | Quantitative evaluation of the FRET ratio and FRET efficiency (n = 3). Data are presented as the mean ± SD. p values are from one-way analysis of variance (ANOVA) with Tukey's multiple comparisons test. Source data are provided as a Source Data file.

Collectively, these results confirm that the sequential "incubation–extrusion" strategy yields a structurally uniform hybrid exosomal platform with verified membrane fusion efficiency, providing a stable and reliable foundation for subsequent nanomotor construction and functional studies.

The above revisions have been incorporated into the revised manuscript; please refer to lines 165-177 (page 5) and lines 714-721 (pages 17-18) for detailed information.

2. In Figures 2F and 2G, supplementary detection of calnexin or GRP94 as negative markers should be performed to rule out endoplasmic reticulum contamination.

Response:

We sincerely thank the reviewer for this important suggestion regarding exosome purity and the potential concern of endoplasmic reticulum (ER) contamination.

In response, we performed additional Western blot analysis using Calnexin as a canonical ER-resident negative marker, and the corresponding results have now been included in the revised manuscript (**Figs. 2F–G**). As shown, both bExos and mExos exhibit clear expression of classical exosomal markers, including ALIX, TSG101, and CD81, whereas no detectable Calnexin signal was observed in either sample, supporting the absence of detectable ER contamination in the isolated vesicle preparations.

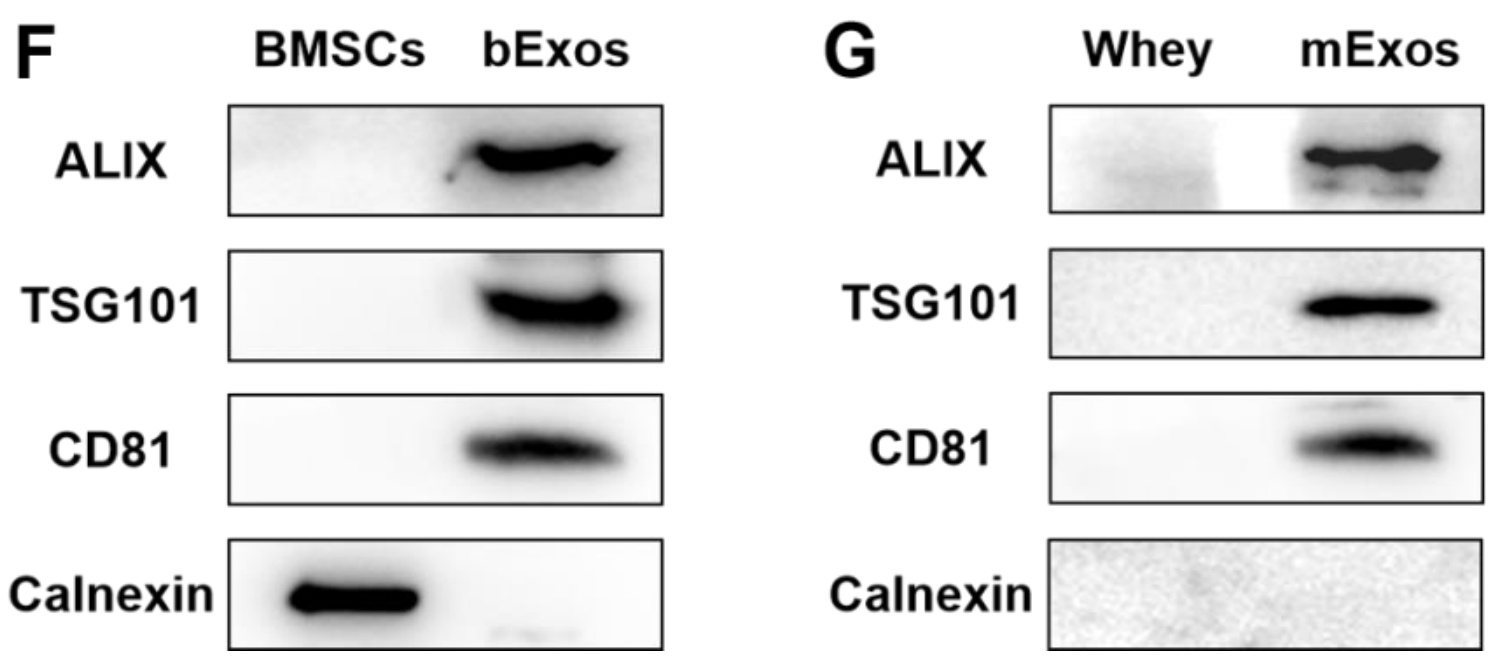

Figs. 2F-G | Western blot analysis of exosomal marker proteins ALIX, TSG101, CD81, and Calnexin. Data are representative of three independent experiments with similar results.

Importantly, the inclusion of positive exosomal markers together with a negative ER marker is consistent with the minimal characterization criteria recommended by the International Society for Extracellular Vesicles (ISEV, MISEV2018 guidelines), further supporting the purity and identity of the isolated exosomal vesicles used in this study.

The above revisions have been incorporated into the revised manuscript; please refer to lines 179–182 (page 5) and lines 683–685 (page 17) for detailed information.

3. Section 5.15 of Materials and Methods mentions the use of the 'ACLT+DMM' surgical model, yet Section 5.10, 'Assessment of Joint Stiffness Capacity', employs a 'monoiodoacetate' injection model. These represent distinct OA models (post-traumatic OA vs chemically induced OA). Why were different models utilised across these experiments?

Response:

We sincerely thank the reviewer for this important question regarding the use of different OA models across experimental sections. In this study, the ACLT+DMM and monosodium iodoacetate (MIA) models were intentionally selected to address different experimental objectives rather than representing inconsistency in the experimental design.

Specifically, the ACLT+DMM model is a well-established post-traumatic OA model that induces progressive cartilage degeneration and joint remodeling through surgical destabilization. This model closely recapitulates the chronic pathological progression of human OA and was therefore used to evaluate the long-term therapeutic efficacy and cartilage-protective effects of CAP-Mn/140@hyExos.

In contrast, the MIA model is a chemically induced OA model that does not require surgical destabilization of the joint or disruption of the joint capsule, thereby minimizing potential interference from surgically induced structural damage during short-term intra-articular distribution and retention studies. In addition, the MIA model rapidly establishes an OA-like oxidative microenvironment characterized by elevated ROS levels, which is consistent with the ROS-responsive transport mechanism investigated in this study. Therefore, this model was used for in vivo evaluation of joint retention, biodistribution, and metabolic behavior of the exosomal nanomotor system.

Taken together, the combined use of these two complementary OA models enabled comprehensive evaluation of the nanomotor platform, including both long-term therapeutic efficacy in a structurally progressive OA model (ACLT+DMM) and short-term intra-articular transport and retention behavior under relatively controlled joint conditions (MIA).

We have clarified this rationale in the revised manuscript to avoid potential misunderstanding; please refer to lines 819-830 (page 20) for detailed information.

4. In vivo therapeutic experiments, specify the number of exosomal nanomotors or protein mass administered per injection.

Response:

We sincerely thank the reviewer for this important comment regarding the clarification of dosing in the in vivo therapeutic experiments.

In this study, all formulations were normalized based on total exosomal protein to ensure strict comparability across experimental groups. Specifically, the 140@bExos group was administered at a concentration of 1 μg μL^{-1}, consistent with our previous reports [1,2]. The hybrid nanomotor systems (CAP-Mn@hyExos and CAP-Mn/140@hyExos) were prepared via membrane fusion and adjusted to the same total exosomal protein concentration (1 μg μL^{-1}). Each intra-articular injection was performed at a fixed volume of 100 μL, corresponding to a total dose of 100 μg exosomal protein per joint.

Accordingly, the administered dose is expressed as total exosomal protein rather than particle number. This is because protein-based quantification is widely adopted in extracellular vesicle studies and provides higher reproducibility across isolation batches and hybridization processes, whereas particle-based quantification (e.g., NTA counts) may introduce variability for hybrid exosomal systems with altered scattering properties.

It is noted that due to the hybrid composition, the miR-140 loading in CAP-Mn/140@hyExos is lower than that in 140@bExos. This design was not intended to match miR-140 dosage alone, but to evaluate the system-level therapeutic contribution of ROS-responsive motility and CAP-mediated cartilage targeting under an equivalent total exosome dose.

Therefore, the comparison focuses on the overall functional performance of the delivery system rather than isolated miRNA dosage equivalence, allowing a fair assessment of transport efficiency, retention behavior, and therapeutic efficacy.

We have clarified this rationale in the revised manuscript to avoid potential misunderstanding; please refer to lines 910-914 (page 22) for detailed information.

References

[1] Liu Y, Huang K, Zhou SL, Li S, Si HB, Zeng Y, Xie HQ, Shen B. Engineered exosomal miR140 modulates mitophagy of chondrocytes through targeting CAPN1 to alleviate osteoarthritis. Sci China Life Sci. 2025 Sep;68(9):2617-2634.

[2] Liu Y, Zeng Y, Si HB, Tang L, Xie HQ, Shen B. Exosomes Derived From Human Urine-Derived Stem Cells Overexpressing miR-140-5p Alleviate Knee Osteoarthritis Through Downregulation of VEGFA in a Rat Model. Am J Sports Med. 2022 Mar;50(4):1088-1105.

5. The degradation and excretion pathways of MnO_2 within the joint cavity and systemic circulation were not discussed. Add long-term (>8 weeks) detection of Mn accumulation in joints and brain tissue.

Response:

We sincerely thank the reviewer for raising this important question regarding the degradation, biodistribution, and potential long-term accumulation of MnO_2 within the joint cavity and systemic circulation. In response, we have added comprehensive in vivo analyses in the revised manuscript, including time-dependent biodistribution imaging, quantitative elemental analysis, and histological evaluation up to 8 weeks.

Whole-body IVIS imaging showed that at 3 days post-injection, fluorescence signals were primarily distributed in major metabolic organs, including the spleen and kidneys. By 2 weeks, signal intensity markedly decreased in most groups, while MnO_2-containing formulations (CAP-Mn@mExos and CAP-Mn@hyExos) still exhibited detectable but reduced signals. At 8 weeks, no obvious fluorescence signals were observed in any major organs, indicating progressive clearance over time (**Fig. S23**).

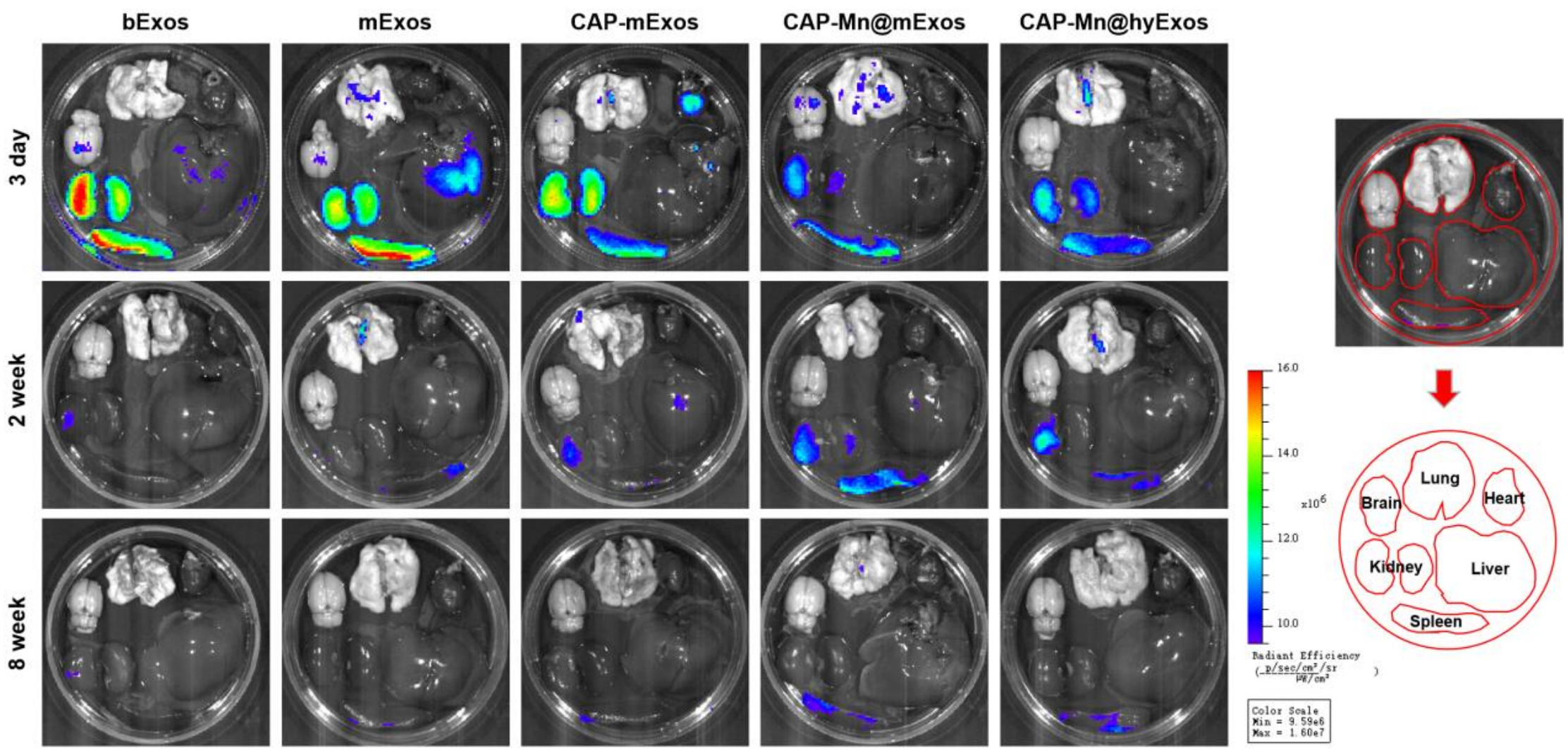

Fig. S23 | In vivo imaging of major organs at 3 days, 2 weeks, and 8 weeks post intra-articular injection of exosomal formulations in OA rats to evaluate biodistribution and metabolic fate (n = 3). Data are representative of three independent experiments with similar results.

Consistently, ICP-OES analysis was performed to quantify total manganese content in major tissues, including joint, liver, spleen, kidney, and brain. The results showed that Mn levels

gradually decreased over time in all examined organs, with no abnormal enrichment observed in the brain at any time point, including 8 weeks (**Fig. S24**). It is important to note that ICP-OES measures total manganese content rather than distinguishing between MnO_2 and its reduced Mn species (e.g., Mn^{2+}), thus reflecting overall systemic manganese burden rather than specific chemical states.

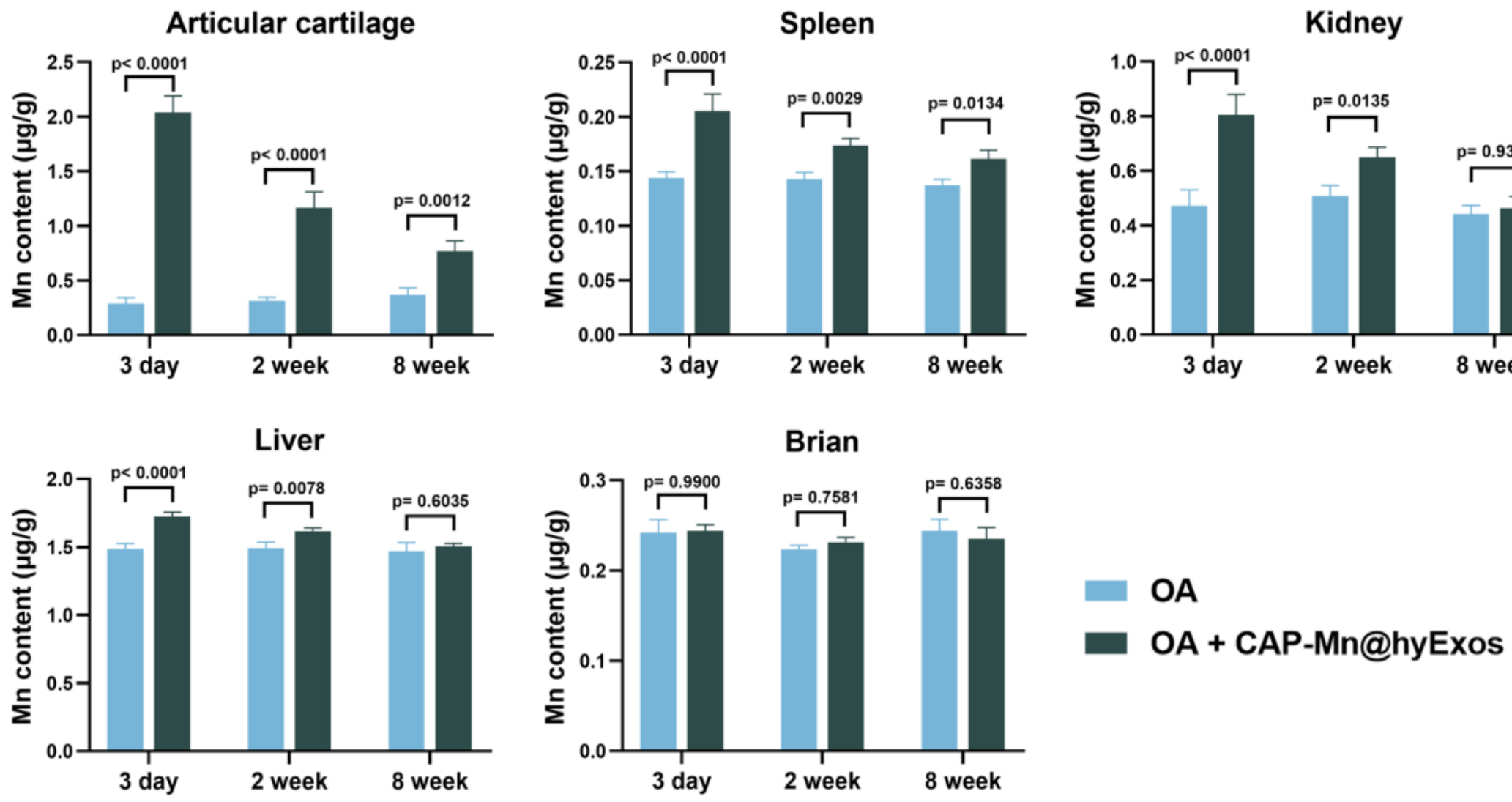

Fig. S24 | ICP-OES quantification of manganese content in joint, spleen, kidney, liver, and brain at 3 days, 2 weeks, and 8 weeks post intra-articular injection of CAP-Mn@hyExos or PBS control in OA rats (n = 3). Data are presented as mean ± SD. p values are from paired two-tailed Student's t-test. Source data are provided as a Source Data file.

Importantly, brain tissue showed no evidence of Mn accumulation by either IVIS or ICP-OES at 8 weeks. In addition, histological evaluation revealed no observable structural abnormalities, inflammatory infiltration, or morphological changes in brain sections (**Fig. S25**), supporting the absence of detectable neurotoxicity under the studied conditions.

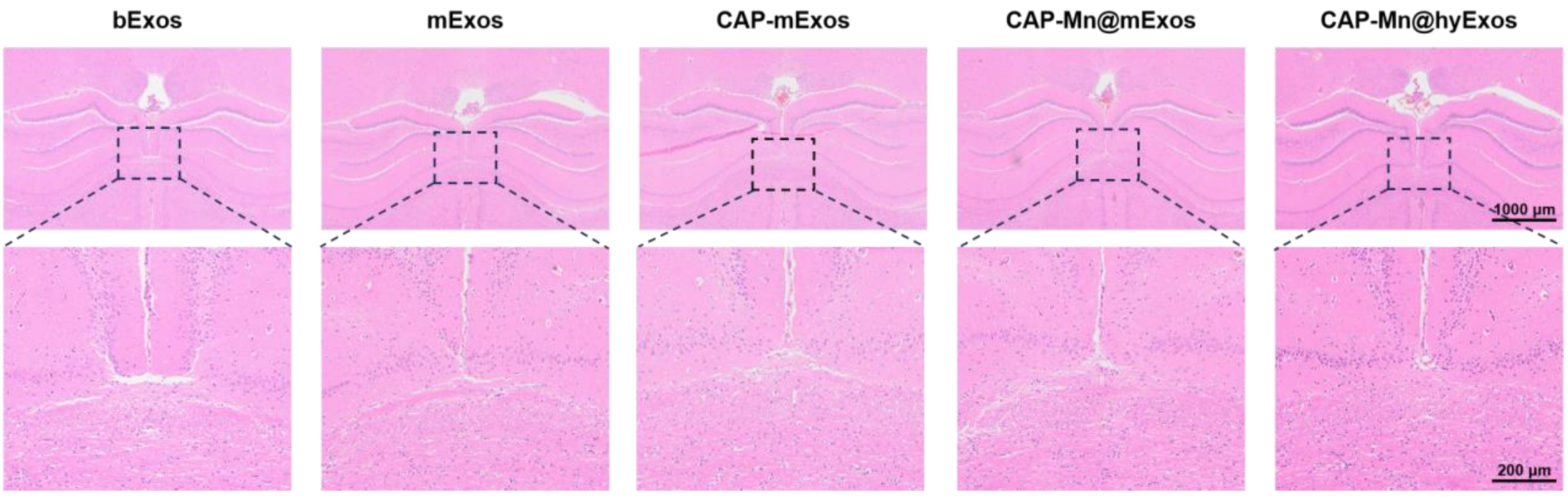

Fig. S25 | Hematoxylin & Eosin staining of brain tissue at 8 weeks after intra-articular injection of exosomal formulations in OA rats to assess potential structural abnormalities, inflammatory infiltration, or pathological changes, scale bars = 1000/ 200 μm. Data are representative of three independent experiments with similar results.

Taken together, these multi-modal results consistently demonstrate a time-dependent decline of Mn-related signals across all evaluated tissues, accompanied by efficient systemic clearance and no evidence of abnormal brain accumulation within the 8-week observation period. While this duration represents the longest time point assessed in the present study, the continuously decreasing trend and lack of detectable residual signals at 8 weeks collectively suggest that delayed accumulation beyond the experimental window is unlikely. Overall, these findings support efficient in vivo clearance of the MnO_2-based nanoplatform following intra-articular administration, without evidence of long-term retention in major organs, including the brain.

The above revisions have been incorporated into the revised manuscript; please refer to lines 295-313 (page 8) and lines 819-830 (page 20) for detailed information.

6. Beyond immunohistochemical validation of Cox4i1, perform protein-level validation for 1–2 core genes within other key pathways identified by GSEA enrichment analysis (e.g., immune regulation-related pathways).

Response:

We sincerely thank the reviewer for this constructive suggestion regarding protein-level validation of key pathways identified by transcriptomic analysis. In response, we have further strengthened the revised manuscript by extending experimental validation from mitochondrial function to additional oxidative stress and immune regulatory pathways, thereby providing a more comprehensive multi-axis mechanistic confirmation of osteoarthritis progression and therapeutic response.

Based on transcriptomic profiling, functional enrichment was performed using KEGG and GO pathway analyses. Representative genes were selected as hub or highly enriched genes within each significantly regulated pathway, taking into account both pathway enrichment significance and gene-level relevance within the corresponding biological modules. This selection strategy

ensures that the validated targets are biologically representative of the dominant pathway activities rather than isolated gene-level fluctuations（**Fig. S37**）.

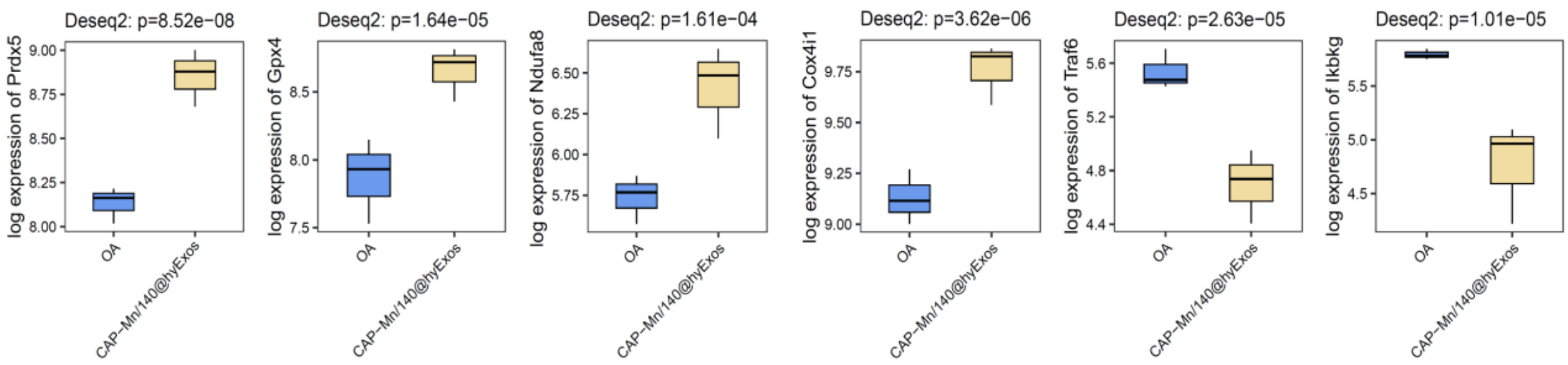

Fig. S37 | Expression levels of key genes involved in oxidative detoxification, mitochondrial oxidative phosphorylation, and anti-inflammatory immune regulation across OA control and CAP-Mn/140@hyExos-treated groups (n = 3).

Specifically, three interconnected functional axes were defined, including oxidative stress regulation, mitochondrial oxidative phosphorylation, and inflammatory immune signaling. These pathways are functionally interdependent and collectively reflect the disruption and restoration of cellular homeostasis in osteoarthritic cartilage.

For oxidative stress regulation, Prdx5 and Gpx4 were selected as representative antioxidant defense proteins, both of which are key regulators of ROS detoxification and lipid peroxidation balance. Immunohistochemical analysis demonstrated that CAP-Mn/140@hyExos treatment significantly upregulated their expression, indicating enhanced antioxidative capacity and attenuation of oxidative injury (**Fig. 9G**).

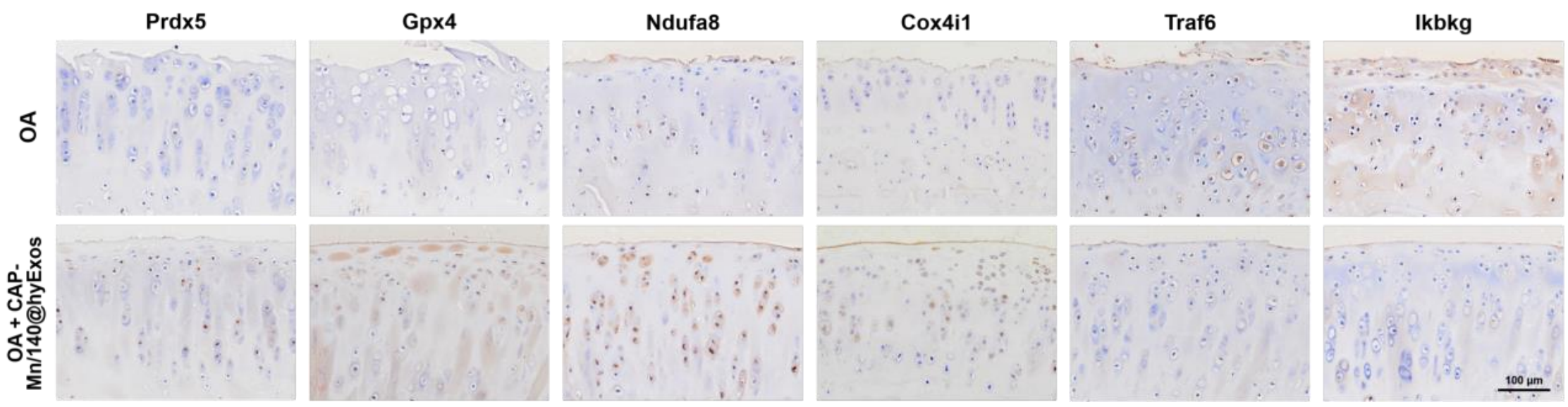

Fig. 9G | Immunohistochemical analysis of proteins encoded by representative DEGs from major pathways in OA cartilage following CAP-Mn/140@hyExos treatment, scale bar = 100 μm. Data are representative of three independent experiments with similar results.

For mitochondrial function, Ndufa8 and Cox4i1 were selected as core components of the electron transport chain complexes I and IV, respectively. Their restored expression at both protein and transcript levels indicates improved mitochondrial respiratory chain activity and recovery of

oxidative phosphorylation function following treatment (**Fig. 9G**).

For inflammatory signaling, Traf6 and Ikbkg were chosen as representative regulatory nodes within the NF-κB signaling cascade. Their downregulation at the protein level suggests suppression of upstream inflammatory signal transduction and attenuation of NF-κB–mediated inflammatory activation in osteoarthritic cartilage (**Fig. 9G**).

Importantly, these protein-level changes observed in tissue sections were further supported by Western blot and qPCR analyses in chondrocyte models, which showed consistent trends across molecular and cellular levels, confirming the robustness of the regulatory effects across different experimental systems (**Figs. 9H-I**)..

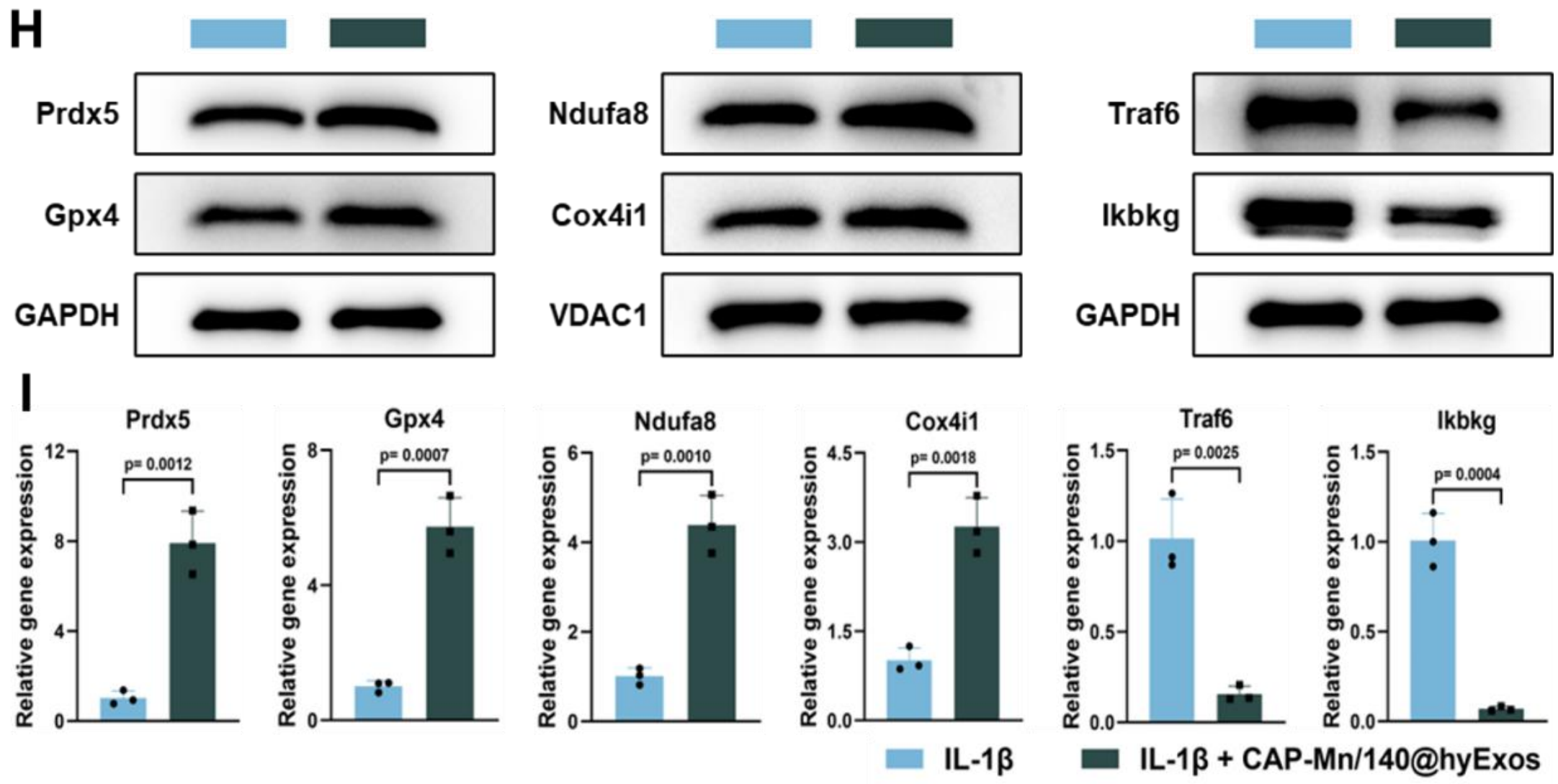

Figs. 9H-I | **H** Western blot analysis of proteins encoded by representative DEGs from major pathways in chondrocyte models. **I** qPCR validation of the same genes in chondrocyte models (n = 3). Data are presented as mean ± SD. p values in **I** are from unpaired two-tailed Student's t-test. Data in **H** are representative of three independent experiments with similar results. Source data are provided as a Source Data file.

Collectively, these multi-level validation results demonstrate coordinated regulation across oxidative stress, mitochondrial dysfunction, and inflammatory signaling pathways, supporting a tightly linked pathological network in osteoarthritis and the multi-target therapeutic effects of CAP-Mn/140@hyExos.

The above revisions have been incorporated into the revised manuscript; please refer to lines 507-521 (page 13) and lines 960-964 (page 23) for detailed information.

7. miRNAs typically suppress target gene expression by inhibiting RNA post- transcriptional translation. However, the key downstream gene Cox4i1 exhibited upregulation following miR-140 delivery. What might be the regulatory mechanism governing the interaction between miR-140 and Cox4i1?

Response:

We sincerely thank the reviewer for this insightful question regarding the apparent upregulation of Cox4i1 following miR-140 delivery, which may seem inconsistent with the canonical gene-silencing function of miRNAs.

First, we would like to clarify that miR-140 does not regulate Cox4i1 through a direct one-to-one targeting relationship. As miRNAs generally exert multi-target, network-level regulatory effects, expression changes in individual non-target genes often arise indirectly as a consequence of broader cellular state transitions rather than direct transcriptional regulation.

In the present study, the observed upregulation of Cox4i1 is not attributed to direct targeting by miR-140. Bioinformatic target prediction did not identify Cox4i1 as a direct binding target of miR-140, further supporting this interpretation. Instead, the change in Cox4i1 reflects a downstream consequence of CAP-Mn/140@hyExos-mediated restoration of osteoarthritic cellular homeostasis.

In osteoarthritic chondrocytes, persistent oxidative stress and inflammatory signaling (particularly ROS- and NF-κB–associated pathways) are known to suppress mitochondrial oxidative phosphorylation activity, thereby contributing to the downregulation of key OXPHOS genes such as Cox4i1. Following treatment, miR-140-mediated regulatory effects together with CAP-Mn–driven ROS modulation attenuate these pathological stress signals, relieving inflammation- and oxidative stress–associated suppression of mitochondrial function and enabling recovery of oxidative phosphorylation-related gene expression.

Within this context, the increase in Cox4i1 should be understood as a functional readout of restored mitochondrial respiratory capacity and improved cellular energetic status, rather than a direct transcriptional target of miR-140. Accordingly, Cox4i1 serves as an indirect but sensitive indicator of mitochondrial functional recovery following system-level reprogramming of the osteoarthritic microenvironment.

Collectively, these findings support a network-level regulatory model in which

CAP-Mn/140@hyExos alleviates oxidative stress and inflammatory signaling, thereby indirectly restoring mitochondrial function and normalizing OXPHOS-related gene expression, including Cox4i1. This interpretation is consistent with the systems-level nature of miRNA-mediated regulation in complex disease microenvironments.

We have clarified this rationale in the revised manuscript to avoid potential misunderstanding; please refer to lines 564-578 (page 14) for detailed information.

Minor issues:

1. Throughout the text, the statistical symbol P should be capitalised and italicised.

2. Line 175: (i) Initially, MnO_2 nanoparticles were synthesised via biomineralisation using bovine serum albumin (BSA)-modified manganese chloride. Manganese chloride is not shown in Figure 2A.

3. Figures 3, 7, and 8 require specification of biological replicates (n values).

4. 'Disccussion' should be corrected to 'Discussion'.

Response:

We sincerely thank the reviewer for the careful review and constructive suggestions. All comments have been addressed as follows:

1. We have revised the manuscript to ensure that the statistical symbol P is consistently capitalised and italicised throughout the text, in full compliance with journal statistical reporting standards.

2. Regarding Line 175, we acknowledge the reviewer's comment and have revised the corresponding description accordingly. In the updated **Fig. 2A**, manganese chloride has been explicitly included and labelled to ensure consistency between the schematic illustration and the experimental description, thereby avoiding potential ambiguity.

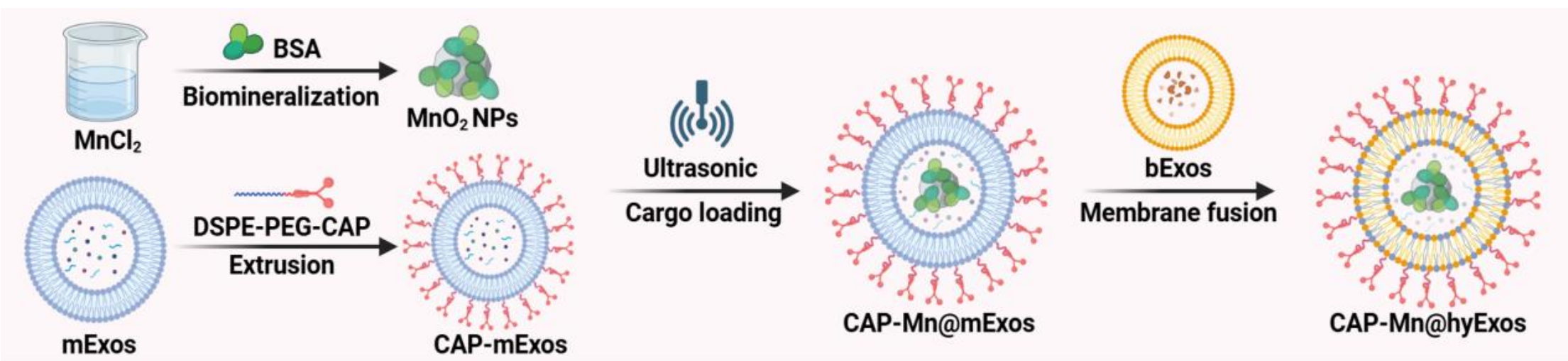

Fig. 2A | Schematic illustration of the fabrication procedure of exosomal nanomotors.

3. We have carefully reviewed all figures involving quantitative analyses and ensured that the number of biological replicates (n values) is explicitly stated in each corresponding figure legend. All reported n values are now clearly specified, improving transparency and rigor in data reporting.

4. The typographical error "Disccussion" has been corrected to "Discussion" throughout the manuscript to ensure typographical accuracy.

Reviewer #4 (Remarks to the Author):

I co-reviewed this manuscript with one of the reviewers who provided the listed reports. This is part of the Nature Communications initiative to facilitate training in peer review and to provide appropriate recognition for Early Career Researchers who co-review manuscripts.

Response：

We sincerely thank Reviewer #4 for their contribution to the peer-review process and for participating in the Nature Communications co-review initiative. We greatly appreciate the time and effort dedicated to the evaluation of our manuscript and the valuable role played in supporting rigorous and constructive peer review.

Point-by-point Response to Reviewers' Comments

We sincerely thank you for the careful evaluation of our revised manuscript and for providing us with the opportunity to further improve our work.

We are grateful for the constructive comments from both the editor and the reviewers, which have significantly enhanced the rigor, clarity, and overall quality of the manuscript.

We fully appreciate the editorial assessment that, although the reviewers recognized the substantial improvements made in the previous revision, several important concerns raised by Reviewer #2 require further clarification to ensure the robustness of the conclusions.

In response, we have addressed all remaining comments through additional experiments, quantitative analyses, and corresponding revisions to the manuscript. These new data further clarify the metabolic fate of the nanomotors, the physiological relevance of the oxidative stress model, the spatial localization within deep cartilage, and the respective roles of ROS-responsive motion and CAP-mediated matrix binding in cartilage delivery.

We believe that these additional results have strengthened the overall rigor and mechanistic interpretation of the study. We hope that the revised manuscript will now be suitable for publication in ***Nature Communications***.

Reviewer #1 (Remarks to the Author):

The authors has addressed all my concerns, I have no further comments.

Response:

We sincerely thank the reviewer for the careful evaluation of our manuscript and for the positive assessment of our revisions. We greatly appreciate the reviewer's recognition that the concerns raised during the previous round have been satisfactorily addressed. The reviewer's constructive comments have been invaluable in improving the quality and rigor of our manuscript.

Reviewer #2 (Remarks to the Author):

I have carefully read the authors' revisions in response to the reviewers' comments, particularly regarding material degradation/fate and in vivo ROS measurements. I would first like to acknowledge the considerable efforts made by the authors. However, several key issues have not been fully resolved, and I suggest that the authors address them in future investigations.

Response:

We sincerely thank the reviewer for the careful evaluation of our revised manuscript and for acknowledging the efforts made in the previous revision. We greatly appreciate the reviewer's constructive comments, which have further helped us improve the rigor and clarity of the study.

We fully recognize the importance of the remaining concerns regarding material degradation and fate, in vivo ROS-related considerations, and mechanistic interpretation. In response, we have addressed all comments through additional experiments, quantitative analyses, and corresponding revisions to the manuscript.

Specifically, we have (i) performed long-term ICP-OES quantification and in vivo biodistribution analysis to further evaluate the metabolic fate and clearance of the nanomotors; (ii) refined the in vitro oxidative stress model by incorporating literature-reported ROS levels in OA and IC_{50}-guided optimization of H_2O_2 concentration; (iii) conducted three-dimensional Z-stack reconstruction and orthogonal-view imaging to better characterize the spatial distribution of nanomotors within deep cartilage, with revised terminology to avoid overinterpretation; and (iv) performed additional comparative experiments using Mn@hyExos to decouple the contributions of ROS-responsive motion and CAP-mediated matrix binding.

We hope that these additional data and revisions adequately address the reviewer's concerns. We are grateful for the reviewer's insightful comments, which have contributed to improving the clarity and robustness of the manuscript

1. The authors used DiI fluorescence labeling to track nanoparticle organ distribution and clearance, observing that fluorescence signals nearly disappeared after 8 weeks, leading to the conclusion of "complete systemic clearance". However, attenuation of fluorescence signal does not directly equate to the metabolism or excretion of the material itself. DiI is a

lipophilic dye that may undergo:Fluorescence quenching, Detachment from the carrier and transfer to other lipid structures, Dilution of the dye within tissues after carrier disintegration, even if the material remains. Therefore, more rigorous methods should be employed to investigate this issue, as it is crucial for assessing translational potential.

Response:

We sincerely thank the reviewer for this valuable comment. We fully agree that attenuation of DiI fluorescence alone cannot be regarded as definitive evidence of material metabolism or systemic clearance, as DiI signals may be influenced by fluorescence quenching, dye dissociation and transfer to other lipid structures, or signal dilution following carrier degradation. Therefore, fluorescence imaging alone has inherent limitations in determining the in vivo fate of the nanomotors.

In the original manuscript, we employed longitudinal in vivo IVIS to dynamically monitor the biodistribution of DiI-labeled exosomal nanomotors following intra-articular injection. Whole-body imaging showed that fluorescence signals were predominantly detected in major metabolic organs, including the spleen and kidneys, at 3 days after administration. By 2 weeks, fluorescence intensity had markedly decreased in all groups, while MnO_2-containing formulations (CAP-Mn@mExos and CAP-Mn@hyExos) still exhibited weak but detectable signals. At 8 weeks, fluorescence signals were minimal to undetectable in any major organs (**Fig. S25**). These longitudinal imaging results demonstrated a progressive reduction of fluorescence signals over time without evidence of persistent fluorescence retention in vivo.

To further address this limitation, we additionally performed ICP-OES to quantify manganese content in major organs at corresponding time points (**Fig. S26**). The results showed a time-dependent decrease in manganese levels across all examined tissues, which gradually approached background levels, with no abnormal accumulation detected in the brain throughout the observation period. The temporal profile from ICP-OES was consistent with the IVIS findings, providing quantitative support for a progressive reduction of nanomaterial signals over time.

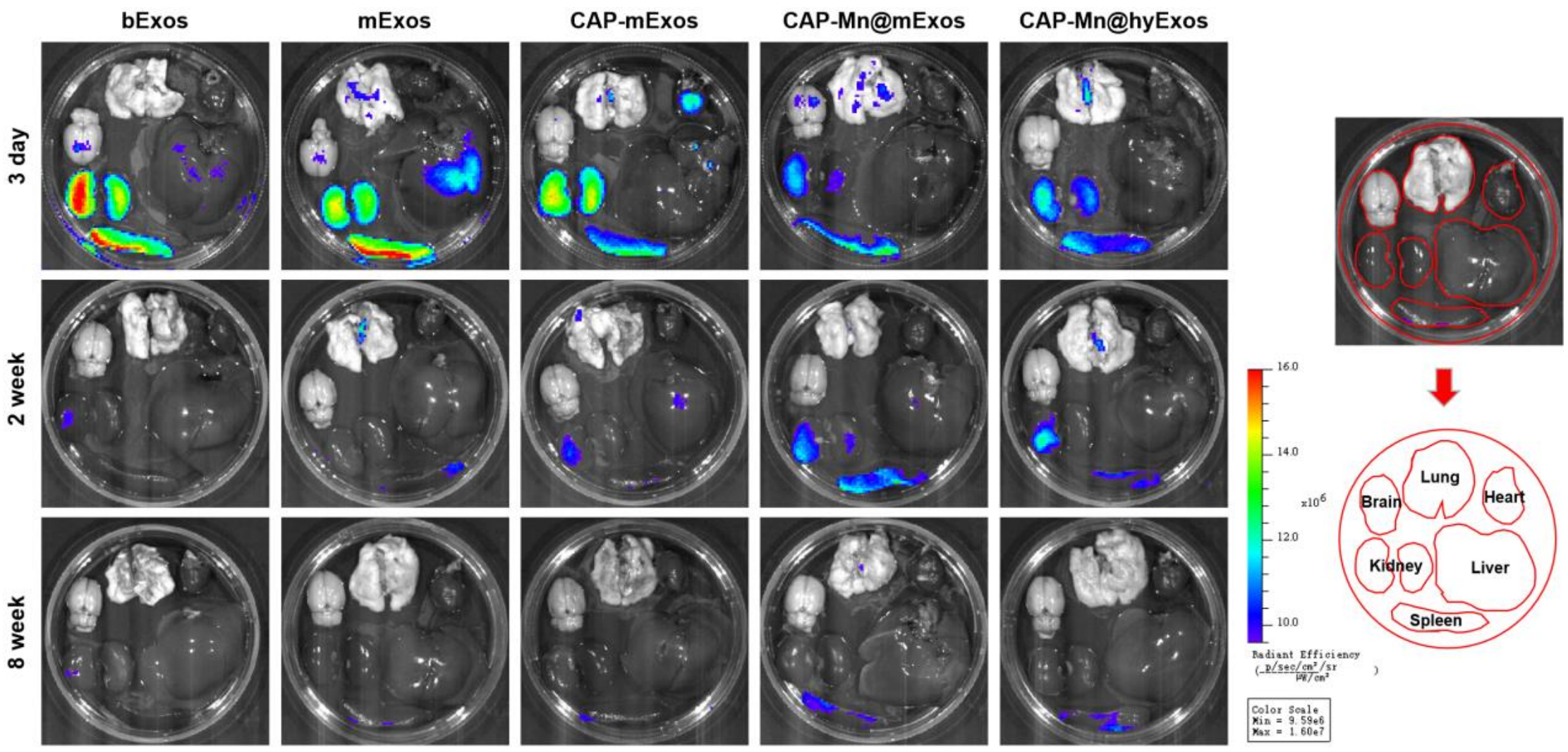

Fig. S25 | In vivo imaging of major organs at 3 days, 2 weeks, and 8 weeks post intra-articular injection of exosomal formulations in OA rats to evaluate biodistribution and metabolic fate. Data are representative of three independent experiments with similar results.

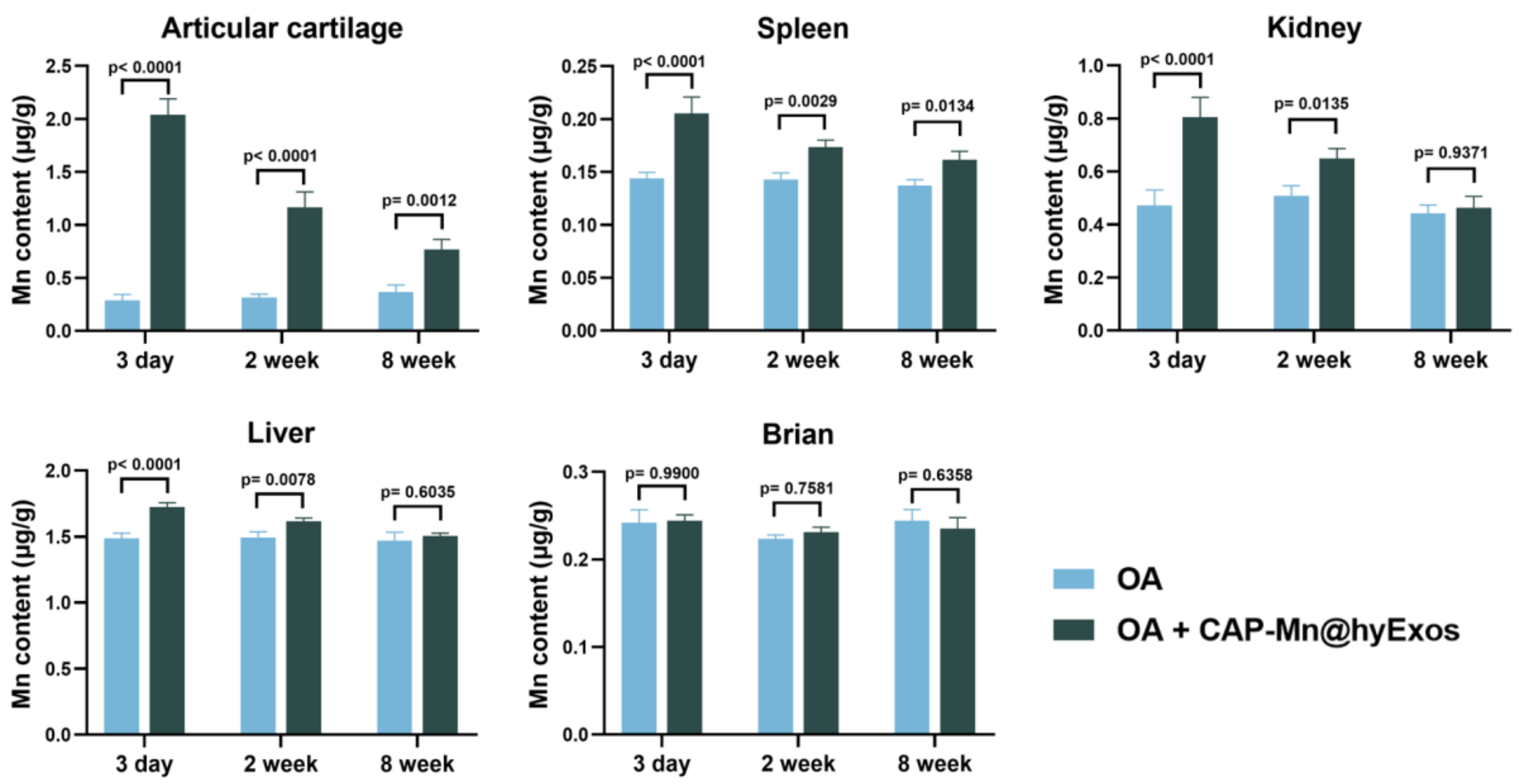

Fig. S26 | ICP-OES quantification of manganese content in joint, spleen, kidney, liver, and brain at 3 days, 2 weeks, and 8 weeks post intra-articular injection of CAP-Mn@hyExos or PBS control in OA rats (n = 3). Data are presented as mean ± SD. p values are from paired two-tailed Student's t-test. Source data are provided as a Source Data file.

These findings are consistent with the reported degradation behavior of MnO_2-based nanomaterials, which can be reduced under physiological conditions to soluble Mn^{2+} species that are subsequently processed through endogenous metal ion metabolic pathways[1]. Although the exact excretion route was not directly traced, the combined IVIS and ICP-OES results consistently

support progressive degradation and systemic clearance of the nanomotor system without evidence of long-term tissue accumulation.

To further evaluate long-term biosafety, we additionally performed histological examination of major organs together with serum biochemical analyses. No obvious tissue injury or organ dysfunction was observed throughout the entire observation period, further supporting the favorable long-term biocompatibility of the nanomotor system (**Figs. S25, S33 & S34**).

Accordingly, we have revised the description of "complete systemic clearance" to "progressive systemic clearance" throughout the manuscript to more accurately reflect the level of evidence provided by the experimental data. **The corresponding changes have been incorporated into the revised manuscript; please refer to lines 317-335 (pages 8-9) and lines 842-863 (pages 20-21) for detailed.**

References

[1] Wang H, Zhao G, Pumera M. Beyond platinum: bubble-propelled micromotors based on Ag and MnO2 catalysts. J Am Chem Soc. 2014 Feb 19;136(7):2719-22.

2. The authors used 100 µM H_2O_2 to establish an oxidative stress model in vitro and demonstrated elevated ROS signals in OA cartilage (Fig. S20). However, they did not actually measure the concentration of H_2O_2 or other ROS species in the joint fluid or cartilage of the animal model. This makes it impossible for readers to judge whether the 100 µM H_2O_2 used in vitro matches the actual in vivo oxidative levels, or whether the nanomotors operate within a "ROS-responsive" window in vivo. I recommend measuring H_2O_2 concentration in OA joint fluid (e.g., using Amplex Red or a chemiluminescence assay), or detecting hydroxyl radicals by microdialysis coupled with liquid chromatography. At the very least, provide in vivo reference values comparable to the in vitro concentration (e.g., reported ROS concentration ranges in similar OA models). Qualitative descriptions such as "elevated and heterogeneous" are insufficient.

Response：

We sincerely thank the reviewer for this insightful comment. We agree that correlating the in vitro oxidative stress condition with the pathological ROS environment in vivo is important for evaluating the physiological relevance of ROS-responsive nanomotors.

Direct quantification of ROS levels in vivo remains technically challenging due to their extremely short half-life, high reactivity, and pronounced spatial–temporal heterogeneity. As a result, reliable absolute measurements in OA joints or synovial fluid are still limited in the current literature.

Nevertheless, previous studies and review articles have reported that ROS/H_2O_2 levels in OA pathological microenvironments may reach approximately 50–100 μM, which is considered sufficient to activate ROS-responsive systems[1–3]. In addition, 100–200 μM H_2O_2 is widely used in OA-related in vitro models to simulate oxidative stress conditions for evaluating ROS-responsive therapeutic strategies[4–7].

Importantly, the selection of 100 μM H_2O_2 in this study was based on both literature evidence and an IC_{50}-guided optimization of chondrocyte viability (**Fig. S33**). This concentration maintains acceptable cell viability while inducing a stable oxidative stress state, providing a reproducible condition for evaluating ROS-responsive behavior.

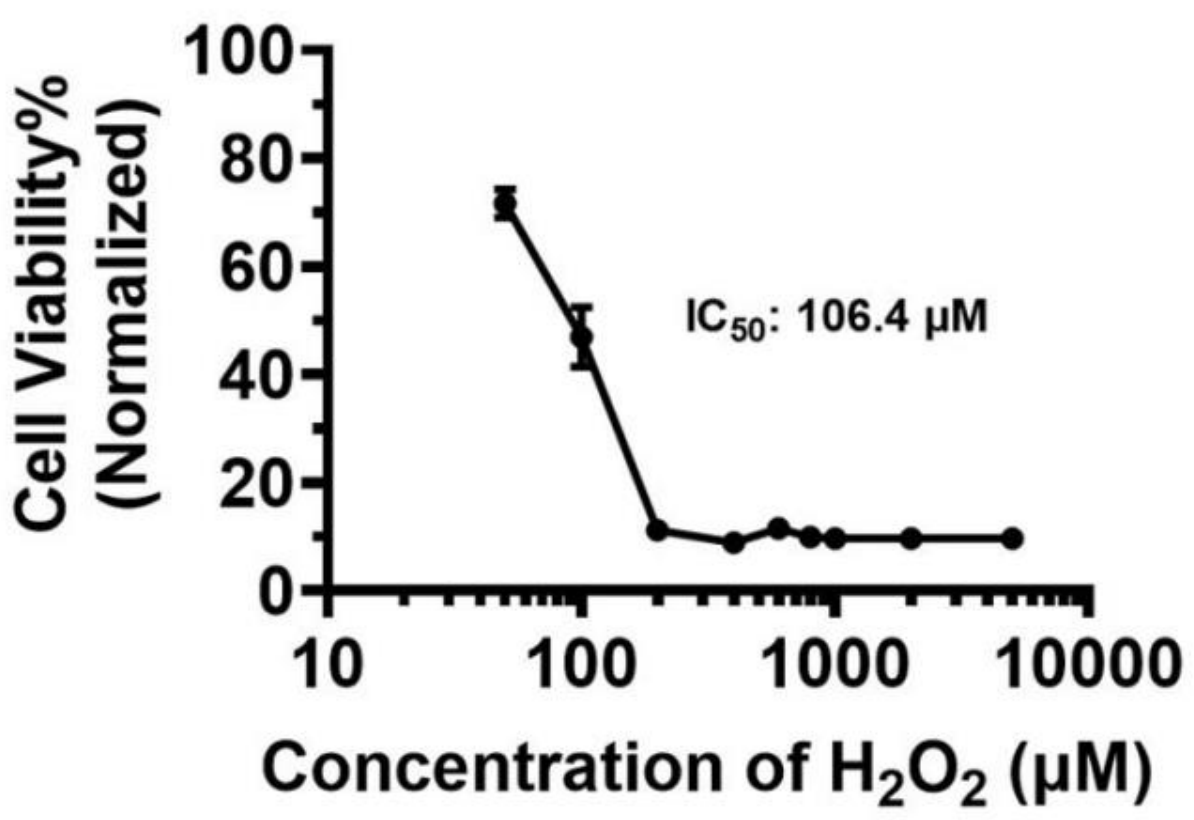

Fig. S33 | IC_{50}-guided optimization of the H_2O_2 working concentration for establishment of the in vitro oxidative stress model (n = 3). Data are presented as the mean ± SD. Source data are provided as a Source Data file.

This rationale is further supported by experimental observations showing elevated ROS levels in OA cartilage (**Fig. S22**), confirming a sustained oxidative stress microenvironment in our model. Consistently, enhanced motility, increased cartilage penetration ex vivo, and improved in vivo accumulation were all observed under this condition, indicating that endogenous ROS levels in OA joints are sufficient to activate the ROS-responsive nanomotor system.

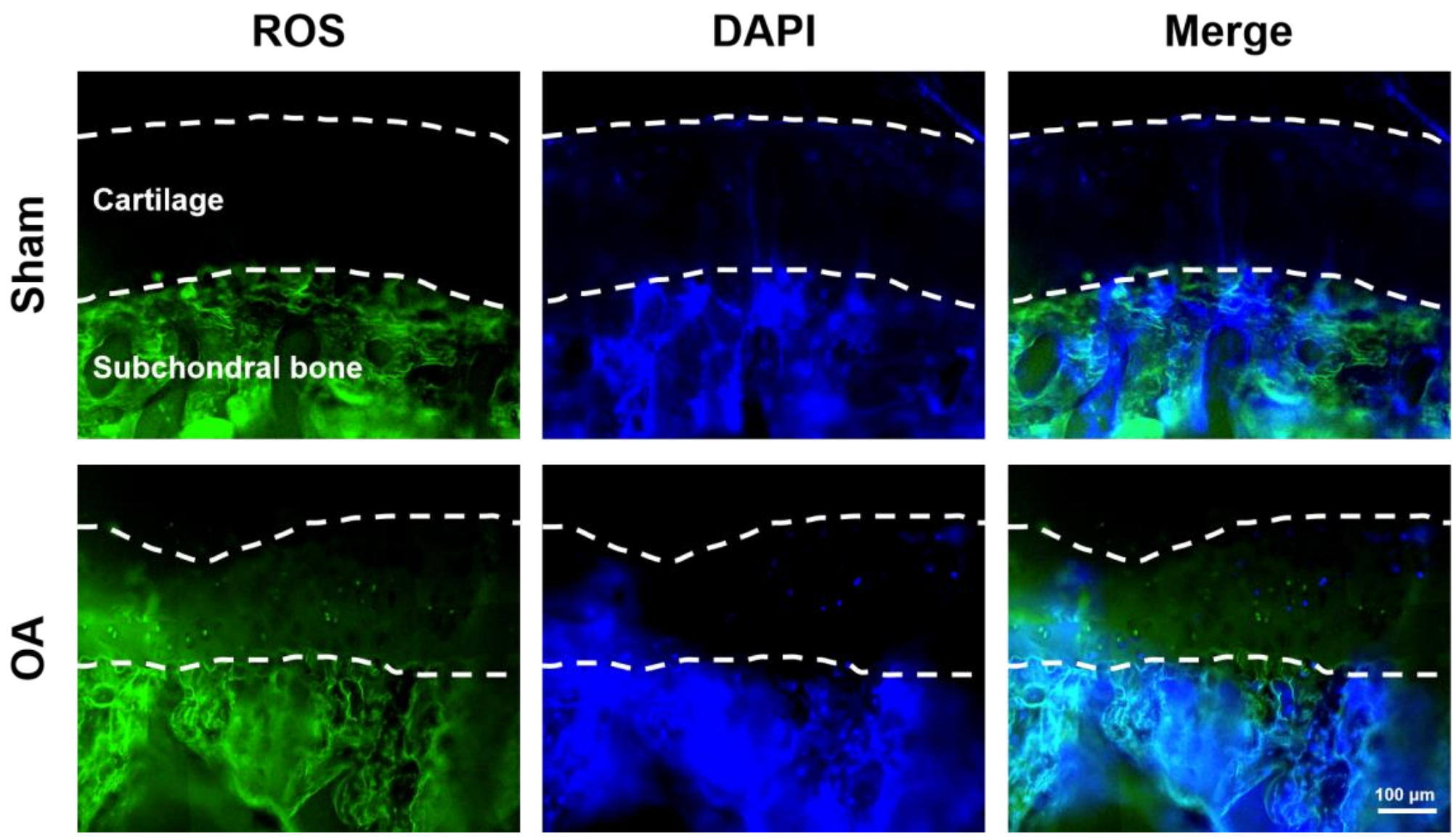

Fig. S22 | Fluorescence imaging of ROS levels and spatial distribution in healthy and osteoarthritic knee cartilage, scale bars = 100 μm. Data are representative of three independent experiments with similar results.

Taken together, the use of 100 μM H_2O_2 is supported by (i) reported ROS levels in OA-related studies, (ii) IC_{50}-guided experimental optimization, and (iii) consistent in vitro and in vivo functional validation. Therefore, this condition was used to establish a functionally relevant oxidative stress model rather than to replicate an exact in vivo ROS concentration.

Following the reviewer's suggestion, the above clarifications have been incorporated into the revised manuscript. Please refer to lines 386-397 (page 10) and lines 904-921 (page 22) for details.

References

[1] Shen C, Gao M, Chen H, Zhan Y, Lan Q, Li Z, Xiong W, Qin Z, Zheng L, Zhao J. Reactive oxygen species (ROS)-responsive nanoprobe for bioimaging and targeting therapy of osteoarthritis. J Nanobiotechnology. 2021 Nov 27;19(1):395.

[2] de Gracia Lux C, Joshi-Barr S, Nguyen T, Mahmoud E, Schopf E, Fomina N, Almutairi A. Biocompatible polymeric nanoparticles degrade and release cargo in response to biologically relevant levels of hydrogen peroxide. J Am Chem Soc. 2012 Sep 26;134(38):15758-64.

[3] Yao Y, Zhang H, Wang Z, Ding J, Wang S, Huang B, Ke S, Gao C. Reactive oxygen species (ROS)-responsive biomaterials mediate tissue microenvironments and tissue regeneration. J Mater Chem B. 2019 Aug 21;7(33):5019-5037.

[4] Gong H, Zhang J, Fan Y, Tian M, Chen C, Tan S, Li C, Du J, He S. Ultrasound-Propelled Antioxidant Nanobowls for the Treatment of Osteoarthritis. Adv Funct Mater. 2026 Mar 1;25(36):e23507.

[5] Wang Q, Feng K, Wan G, Liao W, Jin J, Wang P, Sun X, Wang W, Jiang Q. A ROS-responsive hydrogel encapsulated with matrix metalloproteinase-13 siRNA nanocarriers to attenuate osteoarthritis progression. J Nanobiotechnology. 2025 Jan 16;23(1):18.
[6] Chen M, Liu Y, Liu Q, Deng S, Liu Y, Chen J, Zhou Y, Cui X, Liang J, Zhang X, Fan Y, Wang Q, Shen B. Nanoengineered cargo with targeted in vivo Foxo3 gene editing modulated mitophagy of chondrocytes to alleviate osteoarthritis. Acta Pharm Sin B. 2025 Jan;15(1):571-591.
[7] Zhang Y, Liu W, Liu Z, Liu Y. Daurisoline attenuates H2O2-induced chondrocyte autophagy by activating the PI3K/Akt/mTOR signaling pathway. J Orthop Surg Res. 2023 Mar 27;18(1):248.

3. The authors showed increased penetration depth (~140 μm) and DAPI co-staining indicating signals near cell nuclei. However, it remains unclear whether the signals in deep cartilage layers are truly inside chondrocytes or simply retained in the pericellular matrix. The resolution of confocal microscopy is insufficient to distinguish intra-versus extracellular localization. I suggest using 3D reconstructed confocal imaging or immunogold electron microscopy to localize nanoparticles relative to the plasma membrane/cytoplasm, or performing flow cytometry on enzymatically digested deep-layer chondrocytes to quantify fluorescence positivity.

Response:

We sincerely thank the reviewer for this valuable comment. We agree that conventional confocal microscopy alone cannot definitively distinguish intracellular from extracellular localization within deep cartilage tissue. Therefore, a more cautious interpretation of the localization data is warranted.

First, the intracellular uptake capability of the exosomal nanomotors has been systematically evaluated in our in vitro studies using two complementary approaches. Confocal laser scanning microscopy (CLSM) demonstrated efficient internalization of the nanomotors by chondrocytes, while flow cytometry quantitatively confirmed cellular uptake (**Figs. 3F–I**). Together, these results indicate that chondrocytes are capable of internalizing the exosomal nanomotors.

Second, in the ex vivo cartilage penetration assay, fluorescence signals were observed throughout the cartilage depth and appeared spatially associated with DAPI-positive nuclei. In deeper cartilage regions, where cell density is lower, signals were mainly distributed around DAPI-positive cells rather than in nucleus-free extracellular matrix areas (**Fig. 3D**).

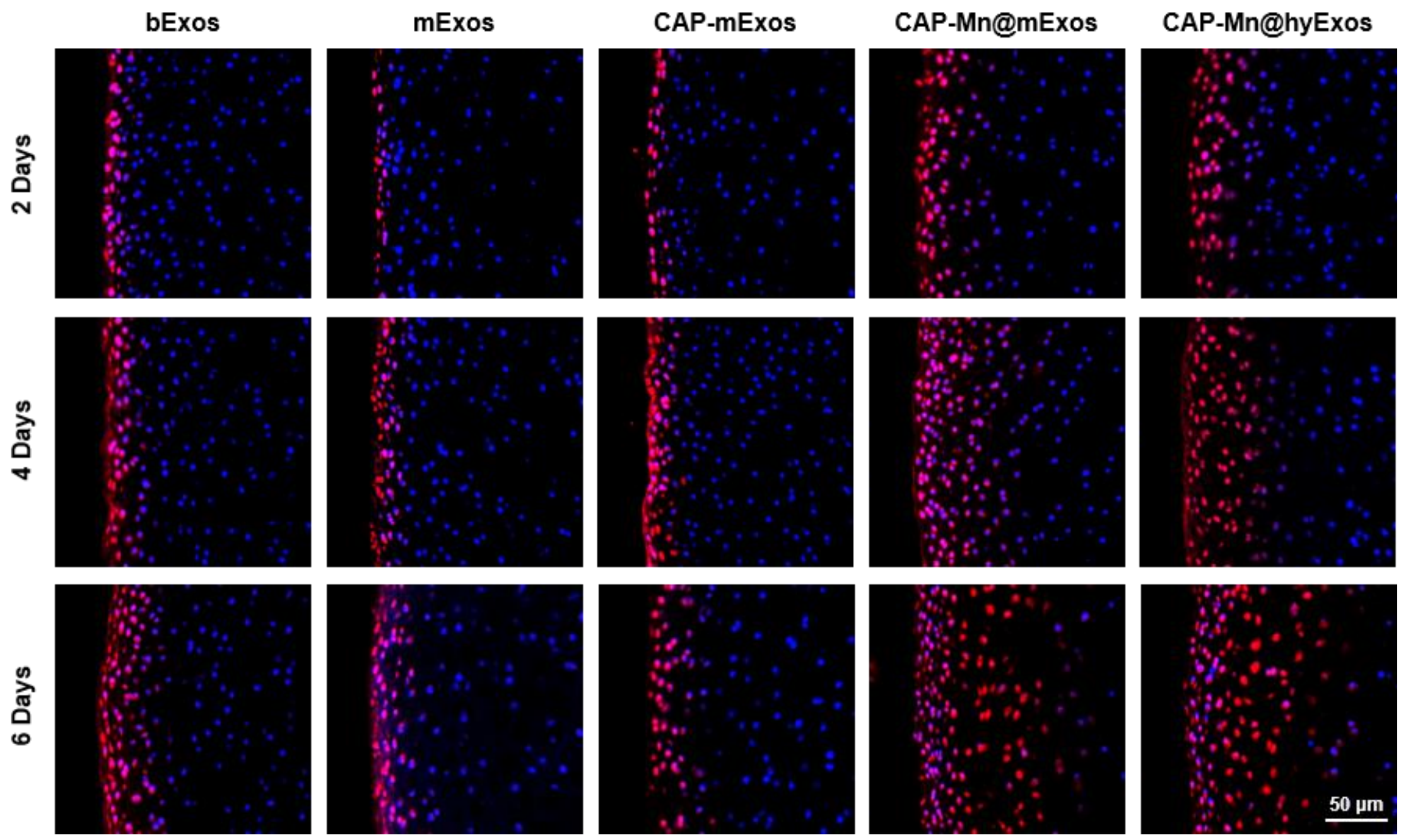

Fig. 3D | D-E Confocal microscopy of ex vivo cartilage sections following incubation with DiI-labeled exosomal formulations for 2, 4, and 6 days, scale bar = 50 μm. Data are representative of three independent experiments with similar results.

Following the reviewer's suggestion, we further performed Z-stack-based 3D reconstruction and orthogonal view analysis (**Fig. S21**). Across optical sections, fluorescence signals remained spatially associated with DAPI-positive nuclei throughout the cartilage depth, while only limited signal was observed in nucleus-free extracellular matrix regions. These results do not indicate widespread extracellular matrix retention and are consistent with close association between exosomes and chondrocyte-rich regions.

Taken together, the in vitro uptake data confirm efficient cellular internalization capability of the exosomal nanomotors, while the ex vivo penetration results together with 3D reconstruction suggest their distribution within cell-rich cartilage regions after tissue penetration. We acknowledge that the spatial resolution of confocal microscopy does not allow definitive determination of intracellular versus pericellular localization at the ultrastructural level.

Accordingly, we have revised the manuscript to describe these findings as "chondrocyte-associated localization" rather than definitive "intracellular localization", thereby providing a more accurate interpretation of the imaging data. **Please refer to lines 249–291 (pages 7-8) and lines 802–815 (pages 19-20) for details.**

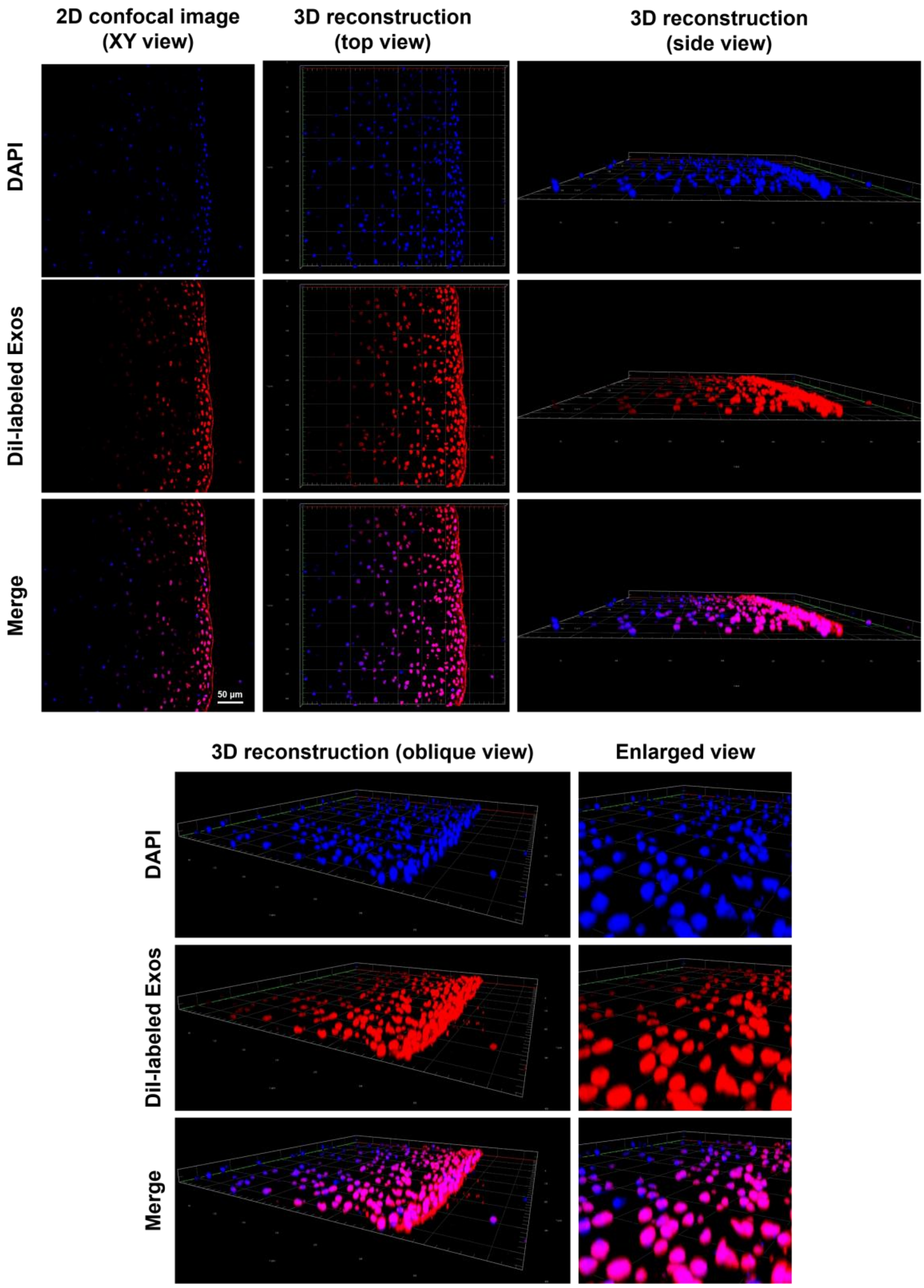

Fig. S21 | Three-dimensional characterization of exosome localization in cartilage after ex vivo penetration. Representative two-dimensional confocal images, three-dimensional reconstructed images, and orthogonal views showing the spatial relationship between DiI-labeled CAP-Mn@mExos (red) and DAPI-stained nuclei (blue) following 6 days of incubation with cartilage explants. Enlarged views highlight the preferential localization of exosomes in cell-rich cartilage regions. scale bars = 50 μm. Data are representative of three independent experiments with similar results.

4. The authors propose that "ROS-responsive motion" and "CAP-mediated matrix binding" collectively enhance distribution, but no experiment was designed to decouple these two contributions. For example, in the absence of CAP modification (i.e., Mn@hyExos without CAP), would the enhanced motion alone be sufficient to achieve deep penetration under ROS conditions? Current data only compare CAP-Mn@hyExos with CAP-mExos (lacking MnO_2). A comparison between CAP-Mn@hyExos and non-targeted Mn@hyExos (without CAP) is missing.

Response:

We sincerely thank the reviewer for this valuable comment. We fully agree that both ROS-responsive motion and CAP-mediated matrix binding may contribute to the enhanced cartilage delivery of the exosomal nanomotors. Therefore, decoupling their respective contributions is essential for elucidating the mechanism underlying the improved cartilage penetration observed in this study.

In the original manuscript, CAP-mExos and CAP-Mn@mExos possess identical CAP modification, while CAP-Mn@mExos additionally contains the MnO_2 propulsion module. Compared with CAP-mExos, CAP-Mn@mExos exhibited significantly enhanced cartilage penetration, suggesting that ROS-responsive motion plays a major role in facilitating active transport and deep cartilage penetration. Notably, CAP-mExos exhibited a penetration depth comparable to that of mExos, indicating that CAP modification alone is insufficient to substantially enhance deep cartilage penetration (**Fig. 3D**).

Following the reviewer's suggestion, we further constructed Mn@hyExos without CAP modification and directly compared them with CAP-Mn@hyExos. These two formulations share the same hybrid membrane structure and MnO_2 propulsion module, with CAP modification as the only variable. This design therefore enables the specific contribution of CAP-mediated matrix binding to be evaluated under comparable ROS-responsive propulsion conditions.

Compared with Mn@hyExos, CAP-Mn@hyExos exhibited increased cartilage accumulation and deeper penetration. These results suggest that CAP modification alone has limited impact on initial penetration, but may enhance nanoparticle interaction with cartilage tissue after ROS-driven transport has occurred (**Fig. S20**).

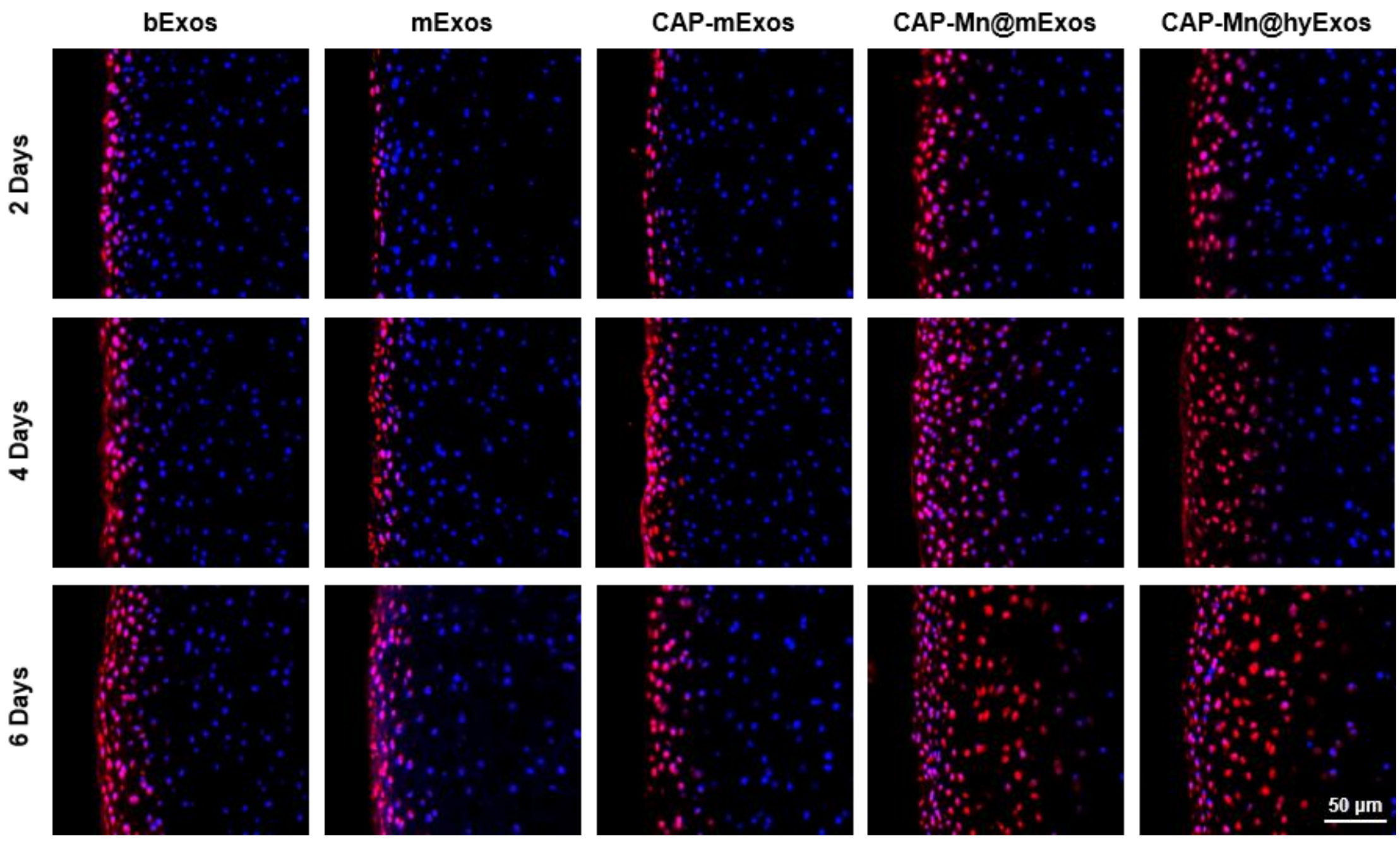

Fig. 3D | D-E Confocal microscopy of ex vivo cartilage sections following incubation with DiI-labeled exosomal formulations for 2, 4, and 6 days, scale bar = 50 µm. Data are representative of three independent experiments with similar results.

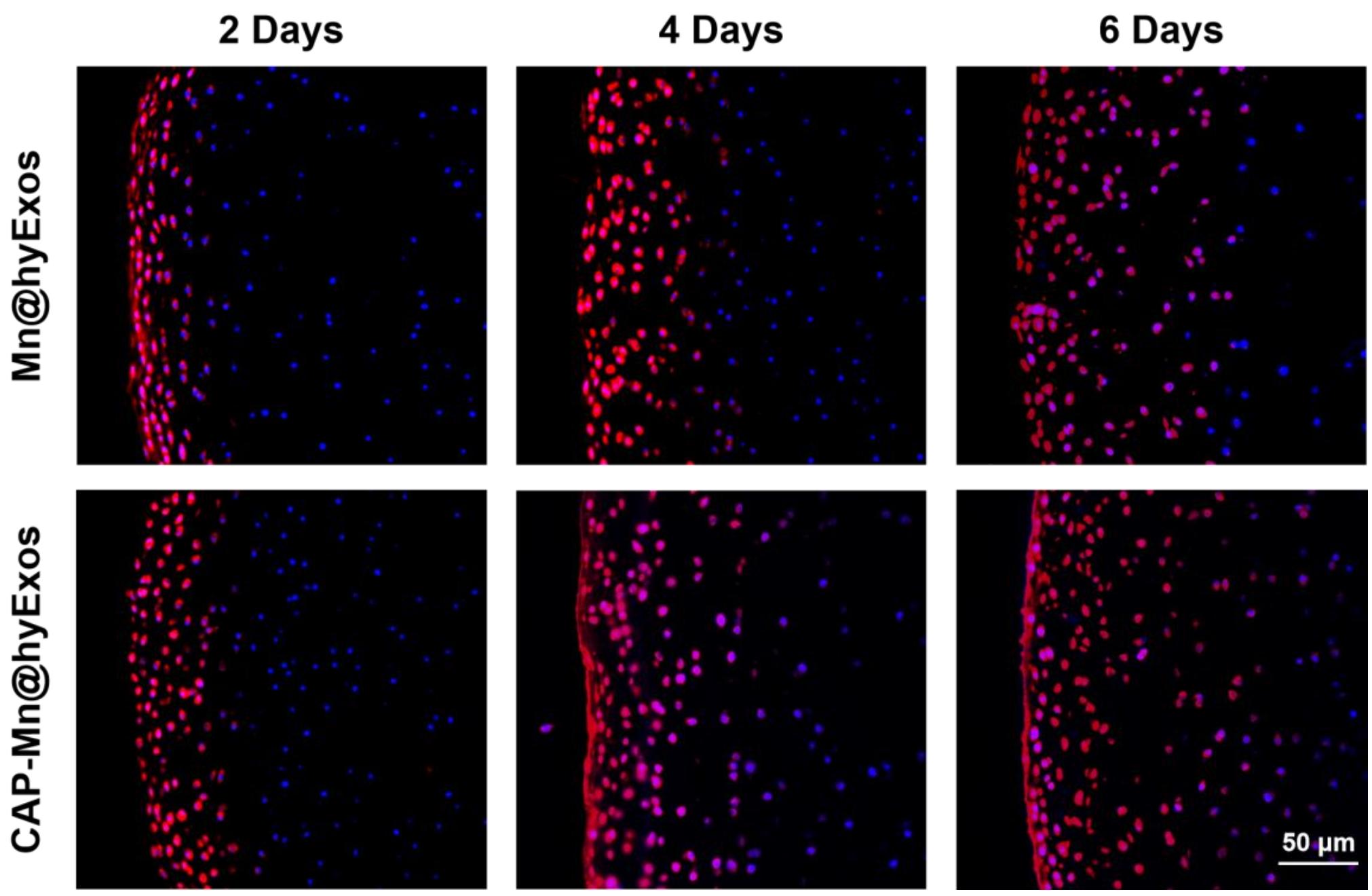

Fig. S20 | Confocal microscopy of ex vivo cartilage sections following incubation with DiI-labeled Mn@hyExos and CAP-Mn@hyExos for 2, 4, and 6 days, scale bar = 50 µm. Data are representative of three independent experiments with similar results.

Rather than acting as an independent penetration mechanism, CAP is more likely to contribute to tissue interaction and local retention, thereby further improving cartilage accumulation and spatial distribution. This observation addresses the reviewer's concern regarding the decoupling of the two mechanisms.

Collectively, these findings indicate that ROS-responsive motion and CAP-mediated matrix binding play distinct but complementary roles in cartilage delivery. ROS-responsive motion primarily facilitates active transport into cartilage, while CAP-mediated binding contributes to enhanced tissue interaction and retention within the cartilage matrix. Accordingly, the improved performance of CAP-Mn@hyExos is likely driven by the combined effects of ROS-responsive transport and CAP-mediated matrix interaction, leading to enhanced cartilage accumulation and distribution.

We have revised the manuscript accordingly to clarify the respective contributions of these two mechanisms. **Please refer to lines 249–291 (pages 7-8) and lines 802–815 (pages 19-20) for details.**

Reviewer #3 (Remarks to the Author):

The authors have addressed all of my questions.

Response:

We sincerely thank the reviewer for the careful evaluation of our manuscript and for the positive assessment of our revisions. We greatly appreciate the reviewer's recognition that all of the questions raised during the previous round have been satisfactorily addressed. The reviewer's constructive comments have been invaluable in improving the quality and clarity of our manuscript.

Reviewer #4 (Remarks to the Author):

I co-reviewed this manuscript with one of the reviewers who provided the listed reports. This is part of the Nature Communications initiative to facilitate training in peer review and to provide appropriate recognition for Early Career Researchers who co-review manuscripts.

Response:

We sincerely thank Reviewer #4 for their contribution to the peer-review process and for participating in the Nature Communications co-review initiative. We greatly appreciate the time and effort dedicated to the evaluation of our manuscript and the valuable role played in supporting rigorous and constructive peer review.
