# Supplementary material for: Hybrid exosomal nanomotors convert pathological ROS into transport-driving cues to enhance cartilage accumulation in osteoarthritis
Source: Nat Commun. 2026 Aug 29;17:10310. doi: 10.1038/s41467-026-76949-8 (PMC13619634; doi:10.1038/s41467-026-76949-8)
Supplement: Supplementary file 4 [file 41467_2026_76949_MOESM5_ESM.docx]

**Figure 1** | Original full-length Western blot membranes for **Supplementary Fig. 6**, showing continuous lanes with preserved molecular weight markers.

**Figure 2** | Original full-length Western blot membranes for **Figs. 2F-G**, showing continuous lanes with preserved molecular weight markers.

**Figure 3** | Original full-length Western blot membranes for **Supplementary Fig. 27**, showing continuous lanes with preserved molecular weight markers.

**Figure 4** | Original full-length Western blot membranes for **Fig. 6E**, showing continuous lanes with preserved molecular weight markers.

**Figure 5** | Original full-length Western blot membranes for **Fig. 9H**, showing continuous lanes with preserved molecular weight markers.
